# Synthesis and biological evaluation of novel hydrazone derivatives for the treatment of Alzheimer's disease

**DOI:** 10.1039/d5ra05755h

**Published:** 2025-11-21

**Authors:** Sazan Haji Ali, Derya Osmaniye, Zafer Asım Kaplancıklı

**Affiliations:** a Department of Pharmaceutical Chemistry, College of Pharmacy, Hawler Medical University Erbil 44000 Iraq sazan.hajiali@hmu.edu.krd; b Department of Pharmaceutical Chemistry, Faculty of Pharmacy, Anadolu University Eskişehir 26470 Turkey; c Central Research Laboratory, Faculty of Pharmacy, Anadolu University Eskişehir 26470 Turkey

## Abstract

In recent years, Alzheimer's disease has emerged as a silent epidemic neurodegenerative disorder. Due to its complex pathophysiology, there has been significant scientific interest in developing effective treatments that go beyond symptomatic relief. The main aim is to improve patients' quality of life and lower the death rate associated with Alzheimer's disease. Since this has not yet been achieved, continued research on Alzheimer's disease remains a global priority. In this study, a total of 27 hybrid molecules (D1a–D1i, D2a–D2i, and D3a–D3i) were designed based on the molecular scaffold of donepezil, a well-known acetylcholinesterase inhibitor (AChEI). These hybrids incorporate dihydrothiazolyl hydrazone and phenyl piperidine moieties. All compounds were synthesized and characterized using IR, NMR, and HRMS spectroscopy, and subsequently evaluated for acetylcholinesterase (AChE) and butyrylcholinesterase (BChE) inhibition using the *in vitro* Ellman method. Evaluation of biological activity revealed that compound D1f exhibited the highest inhibitory activity against the AChE enzyme, with an IC_50_ of (0.039 ± 0.001 Mm). In contrast, none of the compounds showed significant inhibitory activity against the BChE enzyme. Cytotoxicity testing of compound D1f on NIH3T3 fibroblast cells demonstrated non-cytotoxic effects (IC_50_ = 3.324 ± 0.155 µM) and the highest selectivity index (SI = 85.231), respectively. Molecular docking and molecular dynamics simulations verified the stable binding affinity and favorable interactions of compound D1f within the active site of acetylcholinesterase (AChE). The results further demonstrated that the AChE enzyme preserved its structural integrity and compactness throughout its interaction with D1f. Collectively, these observations highlight D1f as a promising lead molecule for subsequent optimization and development of novel anti-Alzheimer's therapeutic agents.

## Abstract 2

This study delivers structurally refined dihydrothiazolyl hydrazone hybrids, leveraging coordinated synthetic design, SAR guidance, and molecular modeling to achieve precise dual-site modulation of acetylcholinesterase enzyme for the treatment of Alzheimer's disease.

## Graphical abstract

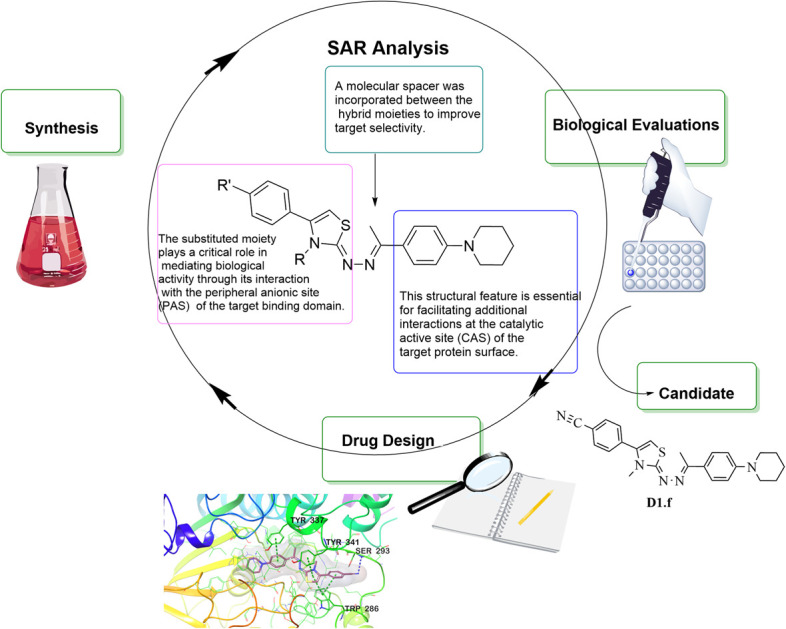

## 1. Introduction

The National Institutes of Health (NIH) describes neurodegenerative disorders as a group of diseases in which specific cells in a particular area of the central nervous system either die or stop functioning.^1^ The 2021 World Health Organization WHO global status report indicates that the frequency of Alzheimer's disease (AD) and projected dementia cases will surge significantly, rising from 55 million in 2019 to 139 million by 2050.^2^ Neurodegenerative disorders tend to progress gradually over time and often have no cure.^3,4^ Among individuals aged 65 and older, AD is the most common form of dementia.^5^ AD remains a complex health issue and is considered an incurable condition in modern medicine.^6^ The mystery surrounding the disease's causes, both from health, social and economic perspectives, makes it one of the most challenging issues today.^7^ The unclear underlying mechanisms further add to the complexity and difficulty of understanding and treating the disease.^5,8^ The primary symptoms of AD include mood and behaviour changes, memory loss, cognitive decline, difficulty performing familiar tasks, and impairments in physical activities.^9–12^ The disease duration, characterized by the patient's struggle with daily activities culminating in memory loss and immobility, is prolonged, lasting approximately 8 to 10 years.^13^ A key neuronal abnormality associated with AD is a reduction in cholinergic neurotransmitter levels, including acetylcholine (ACh), in the cortex and hippocampus.^14,15^

According to research on cholinergic neurons, severe damage and early death of cholinergic neurons in the basal forebrain area can be seen as the disease progresses.^16^ Therefore, the main approach in treating AD is to inhibit the acetylcholinesterase (AChE) enzyme to prevent the breakdown of ACh.^17,18^ In general, an effective AChE inhibitor is typically characterized by three key structural features. First, a central ring system capable of interacting with the enzyme's peripheral anionic site (PAS) is essential. Second, a basic moiety is required to engage the catalytic active site (CAS) of AChE. Third, a linker group such as oxygen, methylene (CH_2_), amide (CONH), substituted amide (CONH(CH_2_)_*n*_), or hydrazone connects the central ring system to the basic center, ensuring proper spatial orientation for optimal enzyme binding.^19^

Based on its chemical structure, acotiamide hydrochloride functions as a selective and reversible AChE inhibitor. Its thiazole and benzamide moieties are key contributors to this inhibitory activity, enhancing ACh availability at neuromuscular junctions and promoting gastric motility.^20^ This advancement prompted the investigation of thiazole-based structures as potential AChE inhibitors for the treatment of AD.^21–25^ The discovery of 2-aminothiazole derivatives as AChEI for the treatment of AD and other neurodegenerative disorders highlighted the utility of the thiazole moiety scaffold for AChE inhibition.^26^ Several studies focused on designing AChE inhibitors based on a phenylpiperidine scaffold as analogs of donepezil. For example, Tok *et al.* (2024) reported the design, synthesis, and molecular modeling of novel donepezil derivatives incorporating a phenylpiperidine moiety with improved AChE inhibitory activity.^19^ These investigations demonstrate phenylpiperidine-based compounds as promising candidates in the development of AChE inhibitors, providing valuable insights into structural optimization for AD therapy.

**Fig. 1:**
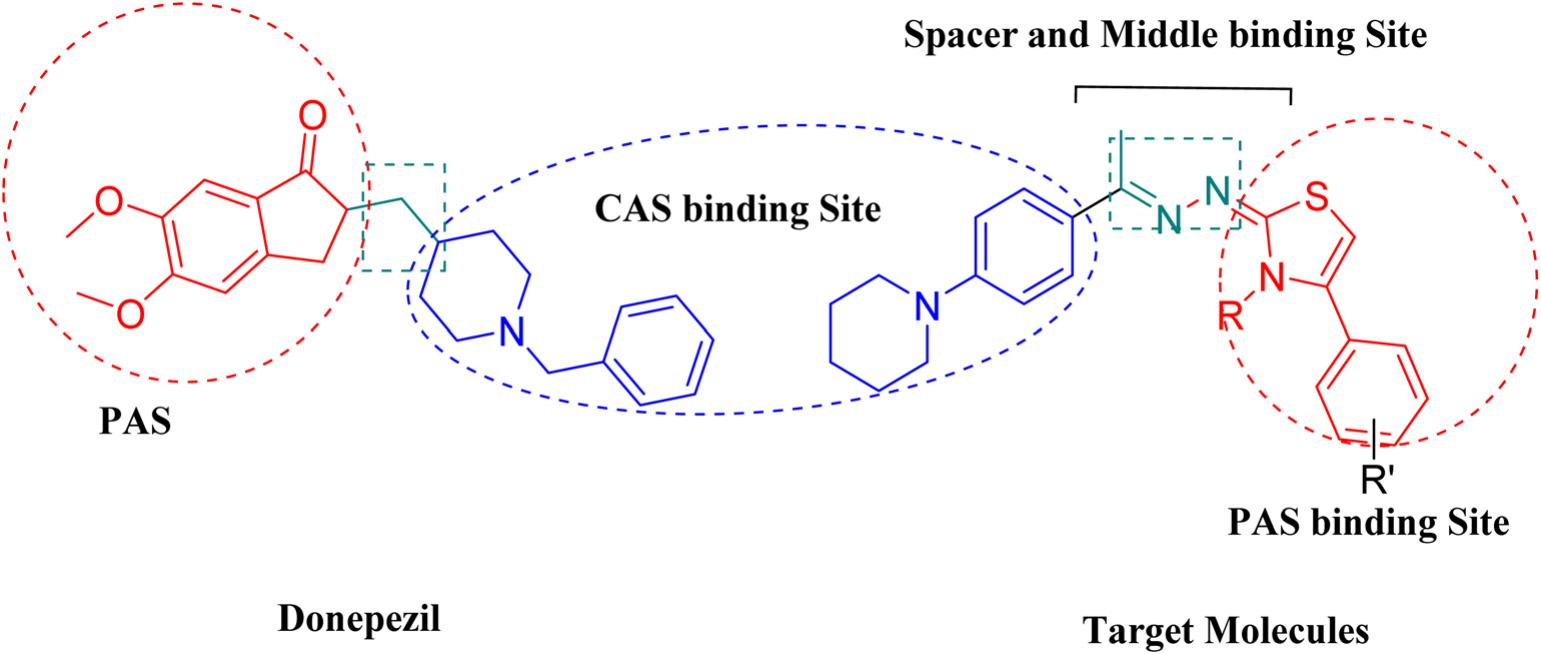
Structural comparison between donepezil and the designed derivatives, highlighting key pharmacophoric modifications.

Building on previous studies, a dihydrothiazolyl hydrazone pharmacophore was designed to target core binding sites on the enzyme. The present work focuses on the design, synthesis, biological evaluation, and molecular modeling of three distinct series of dihydrothiazolyl hydrazone derivatives, developed with consideration of the chemical structure of donepezil as a reference AChE inhibitor (Fig. 1).

## 2. Result and discussion

### 2.1. Chemistry

**Scheme 1:**
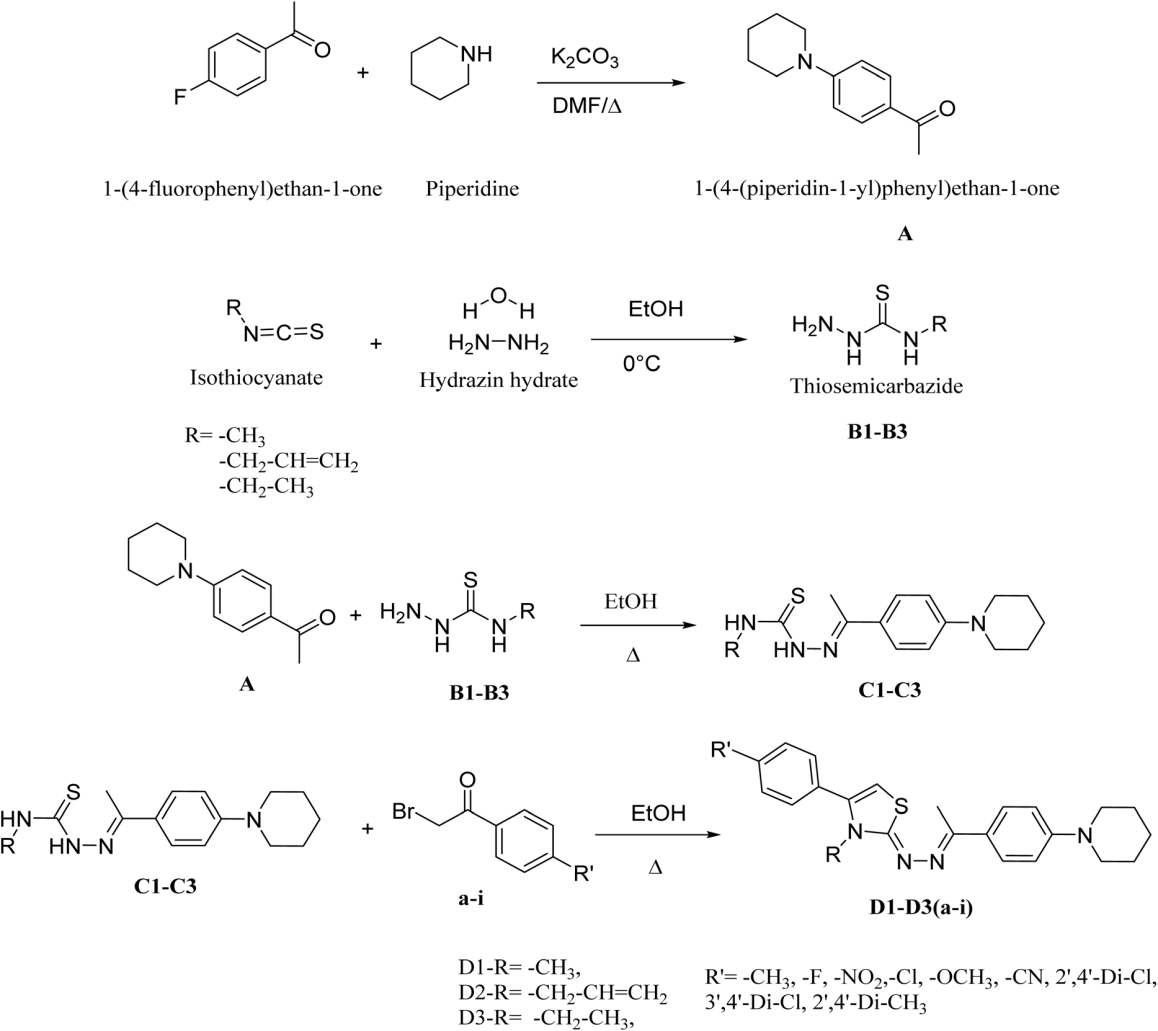
Synthetic mechanism of the targeted compounds.

The synthesis of compounds (D1a–D1i, D2a–D2i, and D3a–D3i) was carried out in several stages, as depicted in Scheme 1. The synthesis began with the reaction of piperidine refluxed in dimethylformamide DMF with 1-(4-fluorophenyl)ethan-1-one for 24 hours at ∼160 °C to obtain compound A. In parallel, the thiosemicarbazide intermediates B1–B3 were prepared by reacting hydrazine hydrate with methyl, ethyl, and allyl isothiocyanates under continuous stirring in an ice bath (0–5 °C) condition for approximately ∼2 hours, affording white crystalline precipitates that were isolated by filtration. The third step involved a condensation reaction between thiosemicarbazide B1–B3 and the obtained compound A to form C1–C3. Specifically, the thiosemicarbazide intermediates (B1–B3) were reacted with compound A in ethanol under reflux conditions (∼78 °C) overnight, using a few drops of concentrated hydrochloric acid as a catalyst, to yield the corresponding intermediates C1–C3. Finally, the target compounds D1a–D1i, D2a–D2i, and D3a–D3i were synthesized *via* a Hantzsch condensation reaction by refluxing the intermediates C1–C3 with the corresponding bromoacetophenone derivatives (a–i) in ethanol overnight (∼12–16 h) at ∼78 °C, affording the desired thiazole derivatives as crystalline solids.^27^ The precipitated products were filtered and recrystallized from hot ethanol to afford pure, crystalline derivatives. The structures of the obtained compounds were confirmed using spectroscopic techniques, including IR, 1H-NMR, 13C-NMR, and HRMS (SI data).

#### 2.1.1. 2D-NMR analyses

The molecular structure and stereochemistry of the target compound 4-(-3-methyl-2-((-1-(4-(piperidin-1-yl)phenyl)ethylidene)hydrazineylidene)-2,3-dihydrothiazol-4-yl)benzonitrile D1f were confirmed through detailed 2D NMR analyses in DMSO-d_6_, including HSQC, HMBC, and NOESY experiments (Table 1). The direct correlation between protonated carbon atoms observed in the HSQC spectra allowed unambiguous assignment of aliphatic and aromatic CH groups throughout the structure with certainty.

**Table 1:** 2D ^1^H NMR and ^13^C NMR analyses of D1f compound

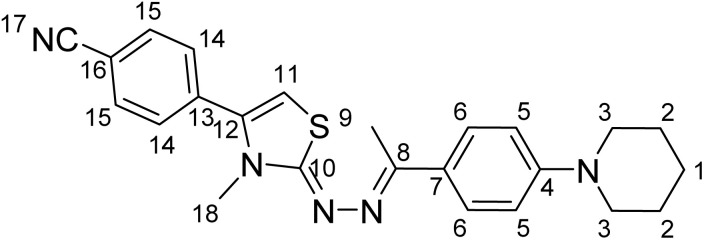		
#	^1^H value (ppm)	^13^C value (ppm)
1	1.60	24.5
2	1.60	25.5
3	3.20	49.3
4	—	152.2
5	6.93	115.1
6	7.68	127.0
7	—	128.5
8	—	155.2
9	2.34	14.5
10	—	167.9
11	6.53	102.9
12	—	139.5
13	—	135.6
14	7.73	129.6
15	7.96	133.2
16	—	119.0
17	—	111.9
18	3.3	34.2

Clear one-bond correlations were observed for the piperidine ring (H-1 to C-1, H-2 to C-2), aromatic protons on the phenyl rings (H-5 to C-5, H-6 to C-6), and the methyl group at position 18 (H-18 to C-18), confirming their chemical environments. The HMBC spectrum revealed long-range (^2^*J* and ^3^*J*) heteronuclear correlations crucial for determining carbon connectivity within the thiazole and hydrazone core. Notably, key HMBC cross-peaks from the methyl group (H-18) to (C-10) supported its placement at the 3-position of the thiazoline ring. The benzonitrile ring exhibited strong correlations from the aromatic protons (H-13, H-14) to quaternary carbon (C-12) and the nitrile-bearing carbon (C-17), consistent with para-substitution. The NOESY spectrum showed through-space proton–proton interactions that helped clarify stereochemical relationships. A distinct NOE cross-peak between H-18 (methyl group) and the nearby hydrazone NH proton supported a (*Z*)-configuration across the thiazoline double bond. Additionally, NOE correlations between the hydrazone NH and the aromatic protons of the adjacent phenyl ring supported the (*E*)-geometry around the imine double bond. These interactions played a crucial role in determining the relative spatial orientation of the substituents, thereby confirming both the regiochemistry and stereochemistry of the compound. The proposed molecular structure and stereochemistry of the thiazoline–hydrazone scaffold were fully confirmed by the combined 2D NMR data, which also verified the *Z*/*E* geometry assignments and the substitution pattern (see SI data).

#### 2.1.2. Structure activity relationship (SAR) of the target molecules

**Fig. 2:**
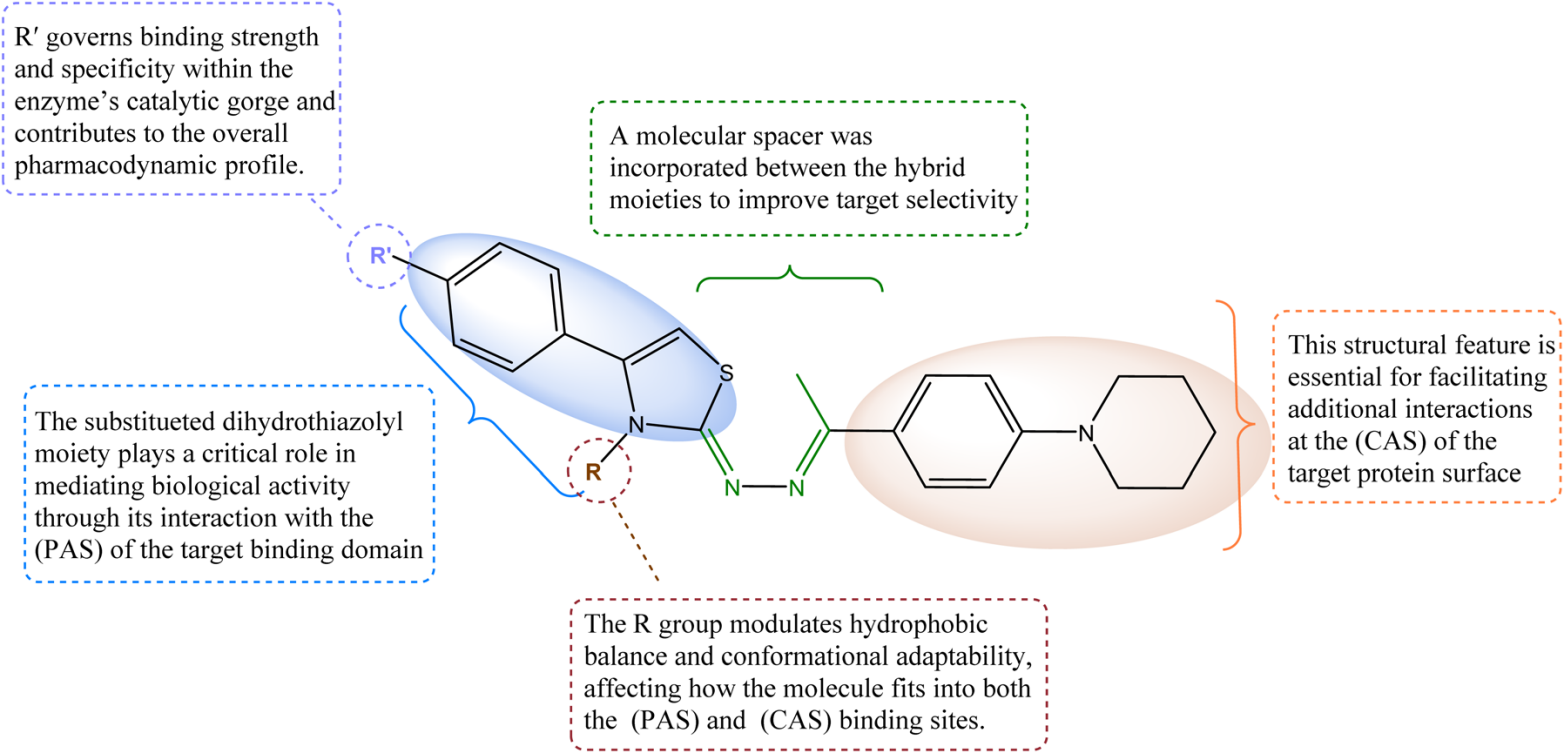
Structure activity relationship (SAR) of the designed molecules as AChEI.

The designed dihydrothiazolyl hydrazone derivatives were rationally developed to fulfill the essential structural requirements of potent acetylcholinesterase inhibitors (AChEIs), integrating key pharmacophoric elements known to engage both the CAS and the PAS of AChE^19^ (Fig. 2). The thiazole ring, serving as the central heterocyclic core, provides an aromatic surface capable of π–π interactions with PAS residues such as Trp286 and Tyr341, similar to the interaction pattern observed in thiazole-based inhibitors like acotiamide.^20^ The hydrazone linkage (–CH=NNH–) functions as a flexible connector, maintaining optimal spatial orientation between the two active regions of the enzyme while contributing additional hydrogen-bonding capacity. The piperidinyl-phenyl moiety acts as the basic center, mimicking the benzylpiperidine fragment of donepezil, and facilitates hydrophobic and cation–π interactions with CAS residues including Tyr337 and Phe338, as demonstrated in molecular docking studies.

Furthermore, the R substituent (methyl, allyl and ethyl) on the thiazole base modulates lipophilicity and steric adaptability, influencing overall binding conformation and affinity. The R′ substituents on the aromatic ring of the bromoacetophenone derivatives critically determine electronic effects and binding strength; electron-withdrawing groups such as –CN enhance hydrogen bonding and π–π stacking within the enzyme's gorge as observed in docking analyses. Among the synthesized derivatives, compound D1f, bearing a methyl group (R = CH_3_) and a *para*-cyano substituent (R′ = –CN), exhibited the most potent AChE inhibitory activity due to its optimal hydrophobic balance and strong dual-site interactions. Collectively, these structural features confirm that the dihydrothiazolyl hydrazone pharmacophore effectively integrates thiazole's PAS affinity, hydrazone's flexibility, and phenyl piperidine's CAS anchoring capacity, making it a promising scaffold for further optimization in AChE inhibitor design for AD therapy.

### 2.2. Cholinesterase enzyme inhibition assay

**Fig. 3:**
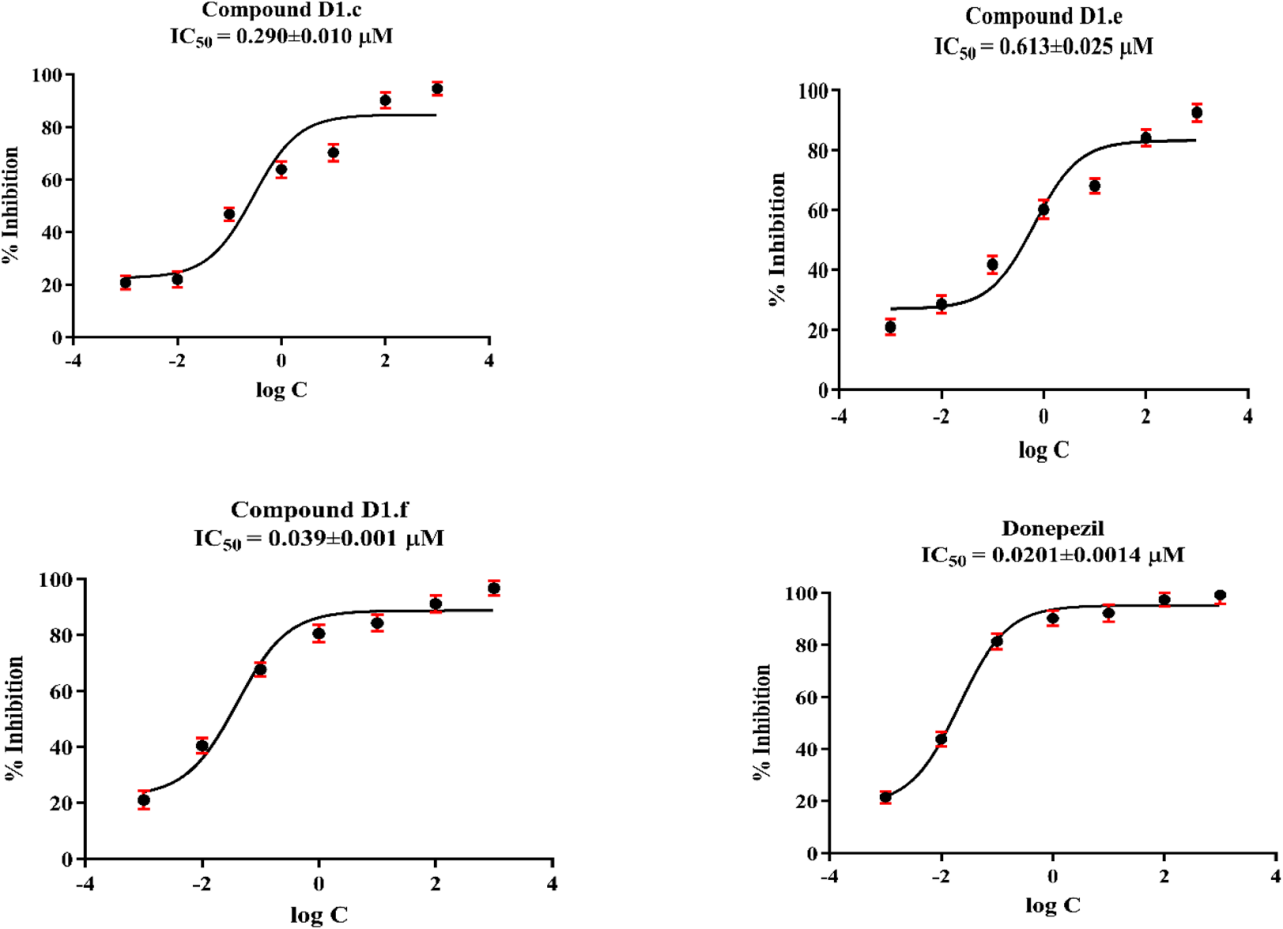
IC_50_ graphs of compounds D1c, D1e and D1f and donepezil on AChE enzyme (the graphs were formed using GraphPad Prism Version 6 *via* regression analyses).

The inhibitory activities of all the obtained hydrazone derivatives (D1a–D1i, D2a–D2i, and D3a–D3i) against cholinesterase enzymes were evaluated using the previously described *in vitro* modified Ellman's spectrophotometric method^28–34^ (Table 2). The assay was completed in two steps. The first step was achieved by means of all the hydrazone derivatives and reference agents, namely donepezil and tacrine, at concentrations of 1000 and 100 µM. The enzyme activity results of the first step are presented in Table 2. Next, the selected compounds (D1c, D1e, and D1f) that displayed more than 50% inhibitory activity at concentrations of 1000 and 100 µM were further tested, along with the reference agents, at concentrations of 10 to 0.001 µM. The IC_50_ values of the test compounds and reference agents are presented in Fig. 3.

**Table 2:** The % inhibition of AChE and BChE enzymes at 10^−3^ and 10^−4^ M concentrations of different bile acids and IC_50_ (µM)

Compounds	AChE% inhibition		AChE IC_50_ (µM)	BChE% inhibition		BChE IC_50_ (µM)
	10^−3^ M	10^−4^ M		10^−3^ M	10^−4^ M	
D1a	40.512 ± 1.923	36.464 ± 1.723	>1000	32.523 ± 1.062	20.422 ± 0.984	>1000
D1b	37.163 ± 1.541	31.058 ± 1.364	>1000	30.154 ± 0.936	19.564 ± 0.864	>1000
D1c	**94.621 ± 2.351**	**90.326 ± 1.526**	**0.290 ± 0.010**	24.401 ± 0.941	21.613 ± 0.852	>1000
D1d	**55.668 ± 2.079**	29.637 ± 1.227	>100	22.954 ± 0.725	15.167 ± 0.734	>1000
D1e	**92.584 ± 1.802**	**84.089 ± 2.150**	**0.613 ± 0.025**	27.636 ± 1.084	17.884 ± 0.710	>1000
D1f	**96.755 ± 2.087**	**91.204 ± 2.427**	**0.039 ± 0.001**	29.020 ± 1.241	16.959 ± 0.784	>1000
D1g	**63.485 ± 1.964**	16.862 ± 0.778	>100	36.345 ± 1.556	31.062 ± 1.067	>1000
D1h	27.564 ± 1.178	14.719 ± 0.646	>1000	31.487 ± 1.310	24.303 ± 0.925	>1000
D1i	**59.330 ± 1.294**	13.658 ± 0.520	>100	40.859 ± 1.864	29.474 ± 0.846	>1000
D2a	38.574 ± 1.354	30.141 ± 1.445	>1000	30.761 ± 1.068	20.898 ± 0.802	>1000
D2b	47.195 ± 2.168	41.667 ± 1.961	>1000	38.232 ± 1.662	27.411 ± 1.248	>1000
D2c	**67.012 ± 1.849**	31.449 ± 0.932	>100	35.010 ± 1.141	30.664 ± 1.151	>1000
D2d	31.205 ± 1.323	28.025 ± 0.884	>1000	30.541 ± 1.028	20.020 ± 0.926	>1000
D2e	39.638 ± 1.757	25.338 ± 1.057	>1000	25.667 ± 0.963	17.235 ± 0.634	>1000
D2f	**75.967 ± 2.281**	29.766 ± 1.264	>100	21.928 ± 0.855	16.489 ± 0.578	>1000
D2g	39.737 ± 1.879	31.619 ± 1.474	>1000	29.330 ± 1.352	23.967 ± 1.074	>1000
D2h	31.520 ± 1.040	20.337 ± 0.989	>1000	36.163 ± 1.361	31.484 ± 0.963	>1000
D2i	28.097 ± 1.251	20.484 ± 0.862	>1000	42.142 ± 2.024	28.710 ± 1.121	>1000
D3a	**56.234 ± 1.751**	37.536 ± 0.921	>100	45.448 ± 2.087	25.320 ± 1.004	>1000
D3b	40.158 ± 1.848	35.848 ± 1.246	>1000	32.557 ± 1.459	22.635 ± 0.964	>1000
D3c	42.798 ± 2.036	38.191 ± 1.738	>1000	33.895 ± 1.545	27.269 ± 1.158	>1000
D3d	32.320 ± 1.441	23.258 ± 1.053	>1000	37.461 ± 1.462	21.348 ± 1.066	>1000
D3e	34.146 ± 1.568	21.564 ± 0.926	>1000	36.305 ± 1.422	30.251 ± 0.956	>1000
D3f	**57.418 ± 1.721**	25.366 ± 0.856	>100	30.246 ± 1.048	21.085 ± 0.947	>1000
D3g	26.655 ± 1.152	20.930 ± 0.884	>1000	33.528 ± 1.264	26.787 ± 1.062	>1000
D3h	28.384 ± 1.250	23.661 ± 0.969	>1000	38.664 ± 1.320	32.469 ± 1.131	>1000
D3i	40.874 ± 1.587	22.148 ± 0.962	>1000	31.537 ± 1.036	24.145 ± 0.858	>1000
Donepezil	99.156 ± 1.302	97.395 ± 1.255	0.0201 ± 0.0014	—	—	—
Tacrine	—	—	—	99.827 ± 1.378	98.651 ± 1.402	0.0064 ± 0.0002

It was observed that all compounds were more effective against AChE. None of the compounds exhibited greater than 50% inhibitory activity against BChE. However, compounds with an aromatic ring bearing strong electron-withdrawing substituents on a methylated thiazole moiety showed greater inhibitory activity among other derivatives and comparable to that of donepezil against AChE. Compounds D1c, D1e, and D1f were selected for the second step of the enzyme activity assay, and their IC_50_ values were calculated by performing an enzyme inhibition study at concentrations of 10 to 0.001 µM. The AChE % inhibition values of compounds D1c, D1e, and D1f were determined to be 96.755 ± 2.087, 94.621 ± 2.351, and 92.584 ± 1.802 µM, respectively. Based on the findings, it can be concluded that the presence of a substituent at the para position of the phenyl thiazole moiety results in improved inhibitory activity and is crucial for biological activity. Furthermore, a shorter *N*-alkyl chain on the thiazole ring was found to enhance the activity. To elucidate the underlying mechanism, molecular docking studies were conducted. The results revealed that strong AChE binding was associated with increased surface hydrophobicity, particularly in derivatives bearing *para*-substituted nitrile, nitro, and methoxy groups.

### 2.3. Evaluation of cytotoxicity effect studies

Compounds D1f and D1c exhibited potent AChE inhibition profiles and were further tested for toxicity using the MTT assay in the NIH/3T3 cell line.^29,35^ The IC_50_ values of the compounds were determined by nonlinear regression analysis, allowing the cytotoxic properties of the compounds to be interpreted. The molecules D1f and D1c were active against AChE enzymes. The inhibition potential (IC_50_ values) of these derivatives on the relevant enzymes ranged from 0.039 ± 0.001 to 0.290 ± 0.010 µM, and their IC_50_ values in the NIH3T3 fibroblast cell line ranged from 3.324 ± 0.155 to 2.410 ± 0.109 µM, as shown in Table 3. This result suggests that the compounds did not show cytotoxic activity. Based on the definition of SI compound D1f can be selected as an ideal compound among all the synthesized compounds, since it exhibits a high selective index, indicating a high relative toxic concentration compared with its low active concentration as an AChE inhibitor. In conclusion, it was determined that compound D1f is not toxic at the IC_50_ concentrations at which it is active against the relevant enzymes.

**Table 3:** Evaluation of IC_50_ values of candidate compounds against AChE enzyme and NIH3T3 cells by MTT cytotoxicity test

Molecule	AChE enzyme	NIH3T3 cell line	Selectivity index^a^
D1c	0.290 ± 0.010	2.410 ± 0.109	8.310
D1f	0.039 ± 0.001	3.324 ± 0.155	**85.231**

a Selectivity index = IC_50_ (NIH3T3)/IC_50_ (AChE).

### 2.4. Docking study

**Fig. 4:**
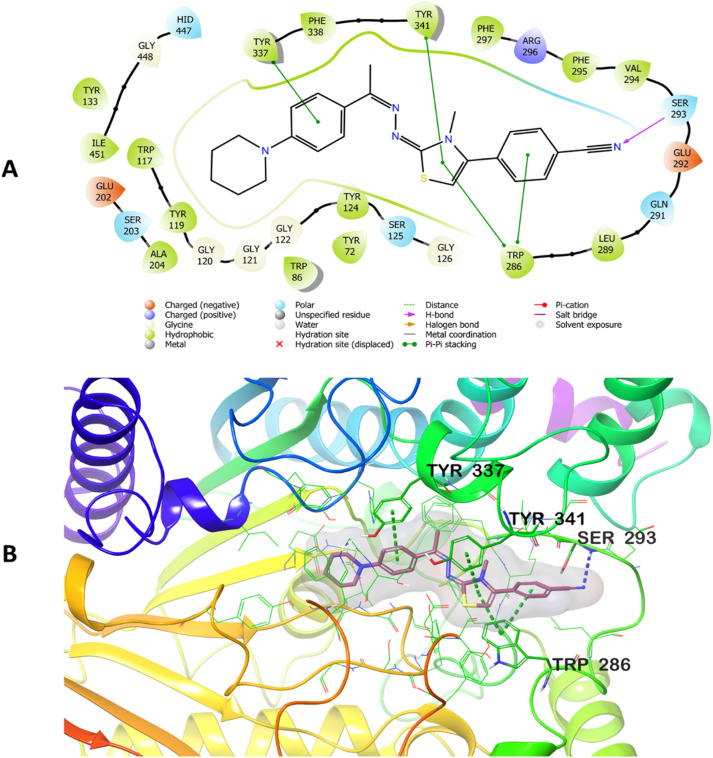
Two-dimensional (A) and three-dimensional (B) view of the interaction of compound D1f with the AChE enzyme active site.

To investigate the potential interactions of D1f, the compound synthesized as part of this thesis with the highest AChE antagonistic activity, its interactions with the enzyme's catalytic core (PDB code: 4EY7)^36^ were analyzed. Using the Glide program,^37^ docking investigations were carried out on the crystal structure. The Glide Score SP method was used to predict the most likely poses. The docking poses that were acquired by working with the AChE enzyme are shown in Fig. 4. The functional moieties of compound D1f, such as 4-cyanophenyl and thiazole rings, establish two π-π interactions with the amino acid Trp286's indole ring, as seen in Fig. 4. Tyr341's phenyl group interacts through π–π stacking with the thiazole ring as well. These interactions show that compound D1f significantly localizes to the PAS region of the AChE enzyme. Additionally, a π–π interaction was observed between Tyr337's phenyl ring and the phenyl ring next to the hydrazone group in the structure's center. The interaction observed with the amino acid Tyr337, compound D1f was found to bind very strongly and effectively to the CAS region of the AChE enzyme active site. The cyano group in the cyanophenyl ring forms a hydrogen bond with the amino group of Ser293. All these findings collectively elucidate the potent blocking activity of compound D1f on the enzyme *in vitro*.

### 2.5. Molecular dynamics simulation study

**Fig. 5:**
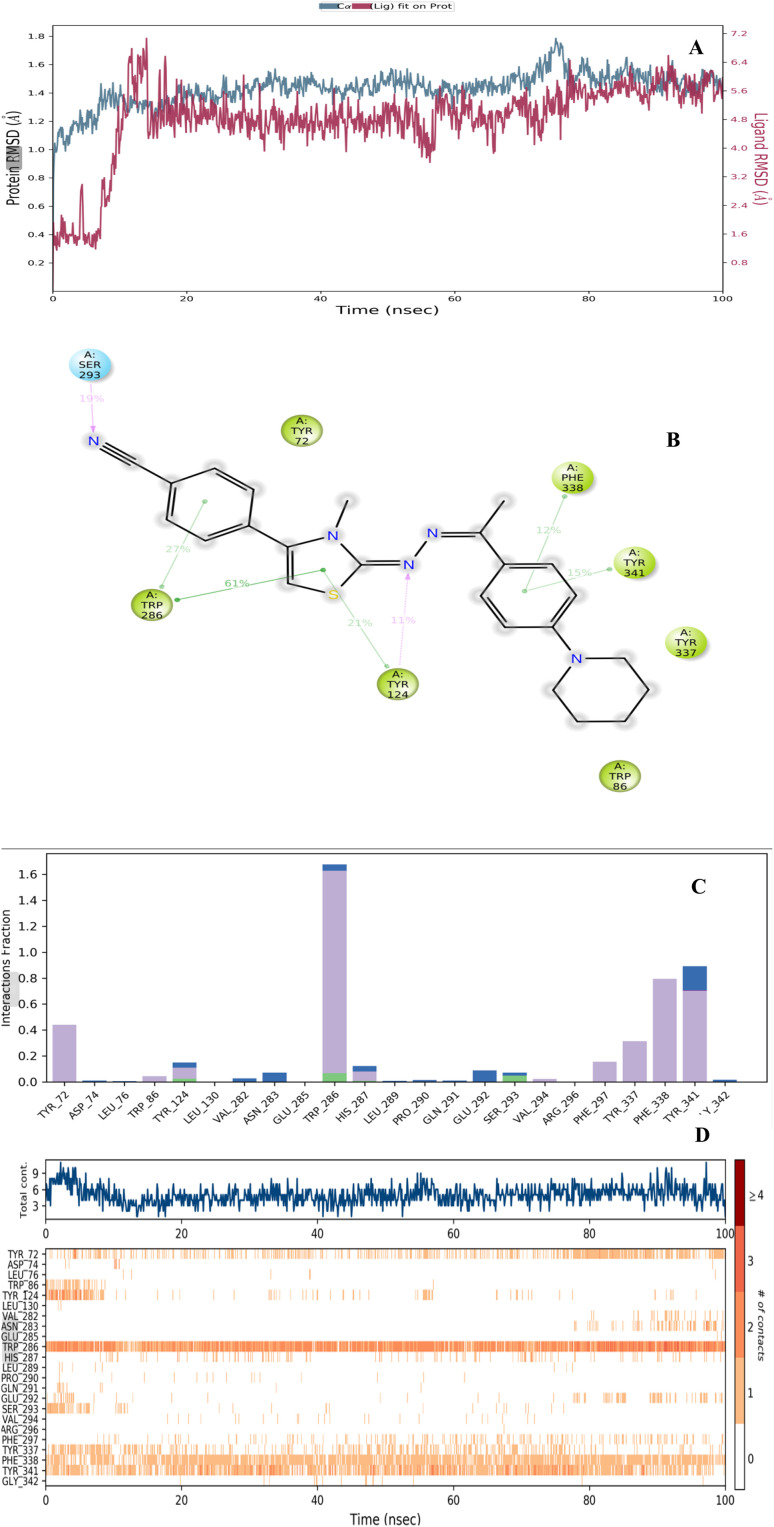
Presents the results of the MD simulation conducted with the compound D1f–AChE complex. (A) displays the RMSD plot plotted against simulation time, (B) highlights the amino acids that interact with the catalytic domain of the enzyme, (C) the interaction fractions by complex throughout the simulation (D) the interaction count *versus* residue plot reveals continuous interactions of compound D1f with residues.

The molecular dynamics (MD) simulation technique is a powerful computational tool for exploring the dynamic stability and interaction profiles of protein-ligand complexes. In this study, a 100 ns MD simulation was performed under an explicit solvent environment to evaluate the stability of the docking complex formed between the potential inhibitor D1f and the AChE enzyme (PDB ID: 4EY7) (Fig. 5).^36^ As shown in Fig. 5A, the root mean square deviation (RMSD) values consistently falling within the specified range of 1–3 Å and the D1f–AChE complex remained within a range of 1.0–1.8 Å throughout the 100 ns simulation. This minimal fluctuation indicates that the complex retained structural stability with no major conformational deviations. The RMSD trajectory showed a slight rise during the initial 11 ns of equilibration but stabilized after approximately 14 ns, confirming that the system had reached equilibrium and the complex maintained a stable conformation during the remainder of the simulation.

Interaction analysis (Fig. 5B–D) provided insights into the key residues contributing to the binding stability of D1f within the enzyme's active site. Residues exhibiting interaction fractions exceeding 10% of the total simulation time were considered significant. The π–π stacking interactions were prominently observed with Trp286 (27%, 61%), Tyr124 (21%), Phe338 (12%), and Tyr341 (15%), while hydrogen bonds were maintained with Tyr124 (11%) and Ser293 (19%). As depicted in Fig. 5C, the color-coded interaction fractions show blue for water-mediated hydrogen bonds, green for conventional hydrogen bonds, and purple for hydrophobic contacts. Hydrogen bonding predominantly involved Tyr124, Trp286, His287, and Ser293, whereas water-mediated hydrogen bonds were detected with Asp74, Tyr124, Trp286, Ser293, and Tyr341. Hydrophobic interactions were further identified with Tyr72, Trp86, Tyr124, Trp286, His287, Val294, Phe297, Tyr337, Phe338, and Tyr341.

The interaction count over time (Fig. 5D) revealed that residues Trp286, Tyr337, Phe338, and Tyr341 maintained persistent contacts throughout the simulation, while interactions with Tyr72 and Phe297 appeared after approximately 25 ns and 43 ns, respectively. These persistent interactions play crucial roles in stabilizing the D1f–AChE complex within the enzyme's core region. Additionally, the MD analysis confirmed aromatic hydrogen bonding with Tyr72, Tyr124, Trp286, Leu289, Glu292, Arg296, Phe338, and Tyr341. These interactions involved the hydroxyl groups of Tyr72, Tyr124, and Tyr341; the carbonyl groups of Trp286, Arg296, Phe338, and Leu289; and the amide group of Glu292, collectively reinforcing the stability and inhibitory potential of D1f.

Overall, the MD simulation findings are consistent with the molecular docking results, demonstrating that compound D1f maintains strong and stable interactions with critical residues in the catalytic domain of AChE. The persistence of π–π stacking and hydrogen-bonding interactions, particularly with Trp286, Tyr337, Phe338, and Tyr341, underscores the compound's favorable conformational stability and high binding affinity. These results strongly support the potential of D1f as a promising AChE inhibitor candidate.

### 2.6. Pharmacokinetic studies

An optimal pharmacokinetic profile with high bioactivity and low toxicity is essential for drug development. To minimize time and resource demands during early drug discovery, computational tools like pkCSM and SwissADME were employed to predict pharmacokinetic parameters (absorption, distribution, metabolism, excretion, and toxicity, ADMET), based on molecular structure.^38,39^ The pharmacokinetic and physicochemical properties of the synthesized compounds were analyzed, as summarized in Table 4–6.

**Table 4:** Physicochemical parameters of active compounds

Molecule	H-Bond acceptors	H-Bond donors	Mr	Tpsa	*I* log *p*	Consensus log *p*	Silicos-it log *p*	Esol log *s*	Surfaces area
D1c	4	0	131.68	106.95	4.29	5.12	6.49	−5.87	184.703
D1e	3	0	129.35	70.36	4.46	4.8	6.13	−5.78	181.528
D1f	3	0	127.58	84.92	4.06	4.58	6.11	−5.66	180.807

**Table 5:** Pharmacokinetic parameters of the active compounds

Compounds	Absorption			Distribution			Excretion	Toxicity	
	Water solubility	Caco-2 permeability	VDss (human)	Intestinal absorption (human)	CNS permeability	BBB permeability	Total clearance	Hepatotoxicity	Max. tolerated dose (human)
D1c	−5.853	0.545	0.561	94.541	−0.755	0.032	−0.064	No	−0.064
D1e	−5.949	1.041	0.717	97.348	−1.43	0.387	−0.051	No	−0.522
D1f	−5.735	1.027	0.551	97.46	−1.475	0.263	−0.002	No	−0.704

**Table 6:** Influence of active compounds on drug-metabolizing enzymes

Molecule	Pgp substrate	CYP1A2 inhibitor	CYP2C19 inhibitor	CYP2C9 inhibitor	CYP2D6 inhibitor	CYP3A4 inhibitor
D1c	No	No	Yes	Yes	No	Yes
D1e	No	No	Yes	Yes	No	Yes
D1f	No	No	Yes	Yes	No	Yes

SwissADME predictions revealed that structural modifications across the D1 series notably influenced key physicochemical parameters, including solubility, lipophilicity, and drug-likeness (Table 4). All compounds **D1c–D1f** possessed 2–4 hydrogen bond acceptors and no hydrogen bond donors, which likely enhance binding affinity and specificity toward AChE through optimal interaction with hydrophobic residues in the active site.

The *I* log *P* values (4.06–4.46) and consensus log *P* values (4.58–5.12) indicate moderate lipophilicity, supporting efficient membrane permeability. The Silicos-it log *P* (6.11–6.49) further suggests enhanced hydrophobic interactions, which aligns with the experimentally observed enhancement in receptor binding affinity. However, the low Esol log *S* values (−5.66 to 5.87) reflect poor aqueous solubility, which may limit oral bioavailability. These effects can be attributed to the introduction of hydrophobic substituents in the modified derivatives, which improve lipophilicity but reduce solubility.

Regarding pharmacokinetic parameters (Table 5), the candidate compounds demonstrated high Caco-2 permeability, indicating efficient gastrointestinal absorption and with 97% intestinal absorption well above the 30% threshold for poor absorption. Distribution volumes ranged from 0.551 to 0.717, which falls within the optimal range for efficient tissue distribution.

Metabolically, the compounds do not inhibit CYP2D6 but act as effective CYP3A4 inhibitors (Table 6), implicating a role in xenobiotic metabolism. Clearance values indicate efficient drug elimination, minimizing accumulation and potential toxicity, and predicted hepatotoxicity was absent in most compounds, suggesting a favorable safety profile.

Overall, physicochemical studies and ADMET analysis confirm the potential of D1c, D1e and D1f as drug candidates. The candidate derivatives exhibit favorable pharmacokinetic profiles, with strong absorption, effective distribution, acceptable metabolism, and low toxicity. Although low solubility may limit bioavailability, this could be addressed through formulation strategies. All the findings collectively support their suitability for further pharmacological evaluation.

## 3. Conclusion

Alzheimer's disease (AD) poses a considerable public health challenge due to its increasing incidence and significant mortality rates. The rising prevalence and high mortality associated with AD underscore the urgency and importance of advancing medical research to find effective therapies.^7^ The most crucial approach in treating AD is to suppress the AChE enzyme in order to stop ACh from being hydrolyzed.^14,40^

In this study, 27 new hydrazinyl di-hydrothiazole derivatives were synthesized as potential candidates for treating AD. The hydrazinyl di-hydrothiazole moiety was identified as a critical pharmacophore, essential for engaging the catalytic domain of AChE enzyme.

The assessment of the synthesized chemicals provides key insights into their potential as inhibitors of AChE and BChE. The synthesized compounds, particularly D1f, D1c, and D1e, demonstrate significant inhibitory activity and low IC_50_ values, comparable to the reference drug donepezil. Their lower activity against BChE suggests a selective inhibition profile, which is beneficial for targeting AChE specifically in therapeutic applications. These findings highlight these compounds as selective AChE inhibitors, offering promise in the treatment of diseases such as Alzheimer's. Cytotoxicity and SI data further establish compound D1f as a superior candidate and stands out as a promising lead for future studies. Compound D1f displayed significant binding interactions with key residues within the enzymatic core of AChE. The combination of docking and MD simulation results demonstrate that compound D1f binds effectively and stably to active regions of AChE, making it a promising candidate for further studies.

In summary, the findings particularly highlight D1f as a promising drug candidate, warranting further investigation to optimize its solubility and evaluate its therapeutic efficacy and safety in preclinical and clinical studies.

## 4. Experimental section

### 4.1. Chemistry

#### 4.1.1. General

All reagents were obtained from commercial sources and were used without any further purification. Melting points (m.p.) were determined using the Mettler Toledo-MP90 Melting Point System, with results reported as uncorrected. For nuclear magnetic resonance (NMR) analysis, a ^13^C-NMR Bruker DPX 75 MHz spectrometer and an ^1^H NMR Bruker DPX 300 spectrometer (Bruker Bioscience, Billerica, MA, USA), were utilized. A sample consisting of 10 mg of the compound was dissolved in 600 µL of DMSO-d6 for the NMR measurements. Mass spectra were recorded using an LCMS-IT-TOF (Shimadzu, Kyoto, Japan) with electrospray ionization (ESI). For high-resolution mass spectrometry (HRMS), 10 mg of each compound were dissolved in 1500 µL of MeOH.

#### 4.1.2. Synthesis of 1-(4-(piperidin-1-yl)phenyl)ethan-1-one (starting material A)

Starting material A, 1-(4-(piperidin-1-yl)phenyl)ethan-1-one, was synthesized by refluxing piperidine (117.64 mmol) with 1-(4-fluorophenyl)ethan-1-one (101.38 mmol) for 24 hours. After cooling, the solid formed was filtered, obtained as yellow powder, and recrystallized from ethanol. Reaction progress was monitored by thin-layer chromatography (TLC).

#### 4.1.3. Synthesis of three different series of thiosemicarbazides (B1–B3)

The compounds B1–B3 were synthesized by reacting methyl, ethyl, and allyl isothiocyanates (1 eq.) with hydrazine hydrate (4 eq.) added dropwise in an ice bath. After 2 hours of stirring, white precipitates formed, which were washed with cold ethanol, dried, and used for the next synthetic step.

#### 4.1.4. Synthesis of *N*-(R)-2-(1-(4-(piperidin-1 yl)phenyl)ethylidene)hydrazine carbothioamide derivatives

The intermediate compound C1–C3 was synthesized from A and B1–B3. This step involved dissolving (5 g) of compound A in ethanol. Subsequently, different amounts (1 eq.) of compounds B1–B3 were added to the flask and refluxed overnight. A few drops of hydrochloric acid were used as a catalyst to accelerate the reaction. The reaction was monitored by TLC (PE : EA 3 : 1). The intermediate C1–C3 was obtained as an off-white powder and purified for use in the synthesis of the target compounds.

#### 4.1.5. Synthesis of the 3-R-2-((-1-(4-(piperidin-1 yl)phenyl)ethylidene)hydrazineylidene)-4-(R)-2,3-dihydrothiazole derivatives (D1a–D1i, D2a–D2i, and D3a–D3i)

Thiazole derivatives were synthesized *via* Hantzsch's method (Hantzsch & Weber, 1887; Ibrahim & Rizk) by refluxing purified intermediates C1–C3 (1 eq.) with (1 eq.) of bromoacetophenone derivatives (Scheme 1) in ethanol overnight. The reaction progress was monitored using TLC (PE : EA 3 : 1). The resulting precipitates were filtered then recrystallized from ethanol to obtain pure crystals of the desired products (Scheme 1).

##### 4.1.5.1 3-Methyl-2-((1-(4-(piperidin-1-yl)phenyl)ethylidene)hydrazineylidene)-4-(*p*-tolyl)-2,3-dihydrothiazole (D1a)

Yield: 75%, m.p.: 223 °C, IR (cm^−1^): 2931 (C=C–H stretching band), 2812 (C–H stretching band), 1589 (C=N stretching band), 1556 (C=C band), 1234 (C–N band), 812 (4-substituted phenyl out-of-plane deformation band). ^1^H-NMR (300 MHz, DMSO-*d*_6_): 1.58 (6H, s, piperidine-H), 2.33–2.36 (6H, s, methyl-H), 3.21 (4H, s, piperidine-H), 3.30 (3H, s, methyl-H), 6.25 (1H, s, dihydrothiazole-H), 6.91–6.94 (2H, d, *J* = 8.06 Hz, Ar–H), 7.29–7.40 (4H, m, Ar–H), 7.67–7.69 (2H, d, *J* = 8.26 Hz, Ar–H). ^13^C-NMR (75 MHz, DMSO-*d*_6_): *δ* = 14.38, 21.34, 24.43, 25.48, 33.83, 49.35, 99.21, 115.11, 127.22, 128.35, 128.71, 128.95, 129.78, 139.08, 140.91, 152.09, 154.57, 168.08. HRMS (*m*/*z*): [M + H]^+^ calcd for C_24_ H_28_ N_4_ S [M + H]^+^: 405.2107; found: 405.2091.

##### 4.1.5.2 4-(4-Fluorophenyl)-3-methyl-2-((1-(4-(piperidin-1 yl)phenyl)ethylidene)hydrazineylidene)-2,3-dihydrothiazole (D1b)

Yield: 86%, m.p.: 233.2 °C, IR (cm^−1^): 3109 (C=C–H stretching band), 2937 (C–H stretching band), 1585 (C=N stretching band), 1504 (C=C voltage band), 1240 (C–N band), 813 (4-substituted phenyl out-of-plane deformation band). ^1^H-NMR (300 MHz, DMSO-*d*_6_): 1.57 (6H, s, piperidine-H), 2.33 (3H, s, methyl-H), 3.21 (4H, s, piperidine-H), 3.30 (3H, s, methyl-H), 6.37 (1H, s, dihydrothiazole-H), 6.91–6.94 (2H, d, *J* = 8.14 Hz, Ar–H), 7.54–7.55 (4H, q, *J* = 5.30 Hz, Ar–H), 7.67–7.70 (2H, d, *J* = 8.69 Hz, Ar–H). ^13^C-NMR (75 MHz, DMSO-*d*_6_): *δ* = 14.15, 24.37, 25.64, 29.72, 33.43, 50.02, 99.61, 115.32, 115.71, 115.99, 127.18, 127.50, 129.61, 130.55, 130.66, 139.62, 152.30, 155.83, 161.39, 164.69. HRMS (*m*/*z*): [M + H]^+^ calcd for C_23_ H_25_ N_4_ FS [M + H]^+^: 409.1857; found: 409.1837.

##### 4.1.5.3 3-Methyl-4-(4-nitrophenyl)-2-((1-(4-(piperidin-1-yl)phenyl)ethylidene)hydrazineylidene)-2,3-dihydrothiazole (D1c)

Yield: 90%, m.p.: 257–259.4 °C, IR (cm^−1^): 3103 (C=C–H stretching band), 2933 (C–H stretching band), 1587 (C=N stretching band), 1516 (C=C band), 1240 (C–N stretching band), 823 (4-substituted phenyl out-of-plane deformation band). ^1^H-NMR (300 MHz, DMSO-*d*_6_): 1.58 (6H, s, piperidine-H), 2.33 (3H, s, methyl-H), 3.21 (4H, s, piperidine-H), 3.30 (3H, s, methyl-H), 6.35 (1H, s, dihydrothiazole-H), 6.90–6.93 (2H, d, *J* = 8.56 Hz, Ar–H), 7.54 (4H, s, Ar–H), 7.67–7.84 (2H, *J* = 8.63 Hz, Ar–H). HRMS (*m*/*z*): [M + H]^+^ calcd for C_23_H_25_N_5_ O_2_S [M + H]^+^: 436.1802; found: 436.1795.

##### 4.1.5.4 4-(4-Chlorophenyl)-3-methyl-2-((1-(4-(piperidin-1-yl)phenyl)ethylidene)hydrazineylidene)-2,3-dihydrothiazole (D1d)

Yield: 80%, m.p.: 216.6 °C, IR (cm^−1^): 3103 (C=C–H stretching band), 2937 (C–H stretching band), 1593 (C=N stretching band), 1537 (C=C band), 1236 (C–N stretching band), 827 (4-substituted phenyl out-of-plane deformation band).

^1^H-NMR (300 MHz, DMSO-*d*_6_): 1.57 (6H, s, piperidine-H), 2.33 (3H, s, methyl-H), 3.21 (4H, s, piperidine-H), 3.30 (3H, s, methyl-H), 6.37 (1H, s, dihydrothiazole-H), 6.91–6.94 (2H, d, *J* = 8.87 Hz, Ar–H), 7.54–7.55 (4H, m, Ar–H), 7.67–7.70 (2H, d, *J* = 8.90 Hz, Ar–H). ^13^C-NMR (75 MHz, DMSO-*d*_6_): *δ* = 14.41, 24.41, 25.46, 33.88, 49.36, 100.56, 113.37, 115.13, 127.26, 128.16, 129.28, 129.73, 130.01, 130.84, 134.24, 139.69, 154.82, 168.10. HRMS (*m*/*z*): [M + H]^+^ calcd for C_23_H_25_N_4_ S Cl [M + H]^+^: 425.1561; found: 425.1544.

##### 4.1.5.5 4-(4-Methoxyphenyl)-3-methyl-2-((1-(4-(piperidin-1-yl)phenyl)ethylidene)hydrazineylidene)-2,3-dihydrothiazole (D1e)

Yield: 65%, m.p.: 217.6 °C, IR (cm^−1^): 2943 (C=C–H stretching band), 2918 (C–H stretching band), 1591 (C=N stretching band), 1537 (C=C band), 1236 (C–N stretching band), 819 (4-substituted phenyl out-of-plane deformation band). ^1^H-NMR (300 MHz, DMSO-*d*_6_): 1.59 (6H, s, piperidine-H), 2.32 (3H, s, methyl-H), 3.20 (4H, s, piperidine-H), 3.28 (3H, s, methyl-H), 3.80 (3H, s, methoxy-H), 6.17 (1H, s, dihydrothiazole), 6.90–6.92 (2H, d, *J* = 7.52 Hz, Ar–H), 7.01–7.03 (2H, d, *J* = 7.29 Hz, Ar–H). 7.39–7.41 (2H, d, *J* = 7.26 Hz, Ar–H), 7.67–7.70 (2H, d, *J* = 7.52 Hz, Ar–H). ^13^C-NMR (75 MHz, DMSO-*d*_6_): *δ* = 14.13, 24.38, 25.65, 33.47, 50.02, 55.42, 98.68, 115.13, 115.40, 123.73, 129.84, 130.10, 140.52, 152.19, 155.43, 160.10, 168.70. HRMS (*m*/*z*): [M + H]^+^ calcd for C_24_H_28_N_4_O S [M + H]^+^: 421.2057; found: 421.2043.

##### 4.1.5.6 4-(-3-Methyl-2-((1-(4-(piperidin-1-yl)phenyl)ethylidene)hydrazineylidene)-2,3-dihydrothiazol-4-yl)benzonitrile (D1f)

Yield: 85%, m.p.: 201.1 °C, IR (cm^−1^): 3107 (C=C–H tension band), 2935 (C–H stretching band), 2229 (nitrile-CN), 1604 (C=N tension band), 1585 (C=C voltage band), 1238 (C–N voltage band), 815 (4-substituted phenyl out-of-plane deformation band). ^1^H-NMR (300 MHz, DMSO-*d*_6_): 1.56 (6H, s, piperidine-H), 2.33 (3H, s, methyl-H), 3.20 (4H, s, piperidine-H), 3.32 (3H, s, methyl-H), 6.51 (1H, s, dihydrothiazole-H), 6.89–6.92 (2H, d, *J* = 8.97 Hz, Ar–H), 7.66–7.72 (4H, t, *J* = 16.35 Hz, Ar–H), 7.92–7.95 (2H, d, *J* = 8.35 Hz, Ar–H). ^13^C-NMR (75 MHz, DMSO-*d*_6_): *δ* = 14.43, 14.47, 24.43, 25.48, 34.16, 49.30, 102.60, 111.84, 115.06, 118.98, 127.29, 128.51, 129.60, 133.14, 135.56, 139.45, 152.16, 155.14, 167.91. HRMS (*m*/*z*): [M + H]^+^ calcd for C_24_H_25_N_5_ S [M + H]^+^: 416.1903; found: 416.1891.

##### 4.1.5.7 4-(2,4-Dichlorophenyl)-3-methyl-2-((1-(4-(piperidin-1-yl)phenyl)ethylidene)hydrazineylidene)-2,3-dihydrothiazole (D1g)

Yield: 75%, m.p.: 179.4 °C, IR (cm^−1^): 3100 (C=C–H stretching band), 2929 (C–H stretching band), 1591 (C=N stretching band), 1562 (C=C band), 1238 (C–N voltage band), 815 (4-substituted phenyl out-of-plane deformation band). ^1^H-NMR (300 MHz, DMSO-*d*_6_): 2.26 (6H, s, piperidine-H), 2.99 (3H, s, methyl-H), 3.32 (3H, s, methyl-H), 3.86–3.90 (4H, m, piperidine-H), 7.59–7.62 (2H, d, *J* = 8.86 Hz, Ar–H), 7.90 (1H, s, dihydrothiazole-H), 8.21–8.25 (1H, m, Ar–H), 8.34–8.37 (3H, m, Ar–H), 8.59–8.62 (1H, d, *J* = 8.59 Hz, Ar–H). ^13^C-NMR (75 MHz, DMSO-*d*_6_): *δ* = 14.39, 24.43, 25.50, 29.86, 48.25, 49.28, 93.08, 115.02, 127.27, 127.84, 128.57, 131.08, 131.67, 132.85, 134.53, 137.88, 152.18, 155.72, 166.26. HRMS (*m*/*z*): [M + H]^+^ calcd for C_23_H_24_N_4_ S Cl_2_ [M + H]^+^:459.1171; found: 459.1164.

##### 4.1.5.8 4-(3,4-Dichlorophenyl)-3-methyl-2-((1-(4-(piperidin-1-yl) phenyl)ethylidene)hydrazineylidene)-2,3-dihydrothiazole (D1h)

Yield: 70%, m.p.: 175 °C, IR (cm^−1^): 3093 (C=C–H tension band), 2931 (C–H stretching band), 1589 (C=N stretching band), 1556 (C=C band), 1234 (C–N stretching band), 821 (3,4-substituted phenyl out-of-plane deformation band). ^1^H-NMR (300 MHz, DMSO-*d*_6_): 1.57 (6H, s, piperidine-H), 2.33 (3H, s, methyl-H), 3.21 (4H, m, piperidine-H), 3.33 (3H, s, methyl-H), 6.46 (1H, s, dihydrothiazole-H), 6.90–6.93 (2H, d, *J* = 8.61 Hz, Ar–H), 7.50–7.53 (1H, d, *J*_1_ & *J*_2_ = 8.31 Hz, Ar–H), 7.66–7.69 (2H, d, *J* = 8.47, Hz, Ar–H), 7.73–7.76 (1H, d, *J* = 8.27 Hz, Ar–H), 7.82 (1H, s, Ar–H). ^13^C-NMR (75 MHz, DMSO-*d*_6_): *δ* = 14.42, 24.43, 25.48, 33.89, 49.31, 101.65, 115.07, 127.27, 129.22, 131.33, 131.68, 132.02, 138.48, 152.13, 154.98, 167.80. HRMS (*m*/*z*): [M + H]^+^ calcd for C_23_H_24_N_4_ S Cl_2_ [M + H]^+^:459.1171; found: 459.1166.

##### 4.1.5.9 4-(2,4-Dimethylphenyl)-3-methyl-2-((1-(4-(piperidin-1-yl)phenyl)ethylidene)hydrazineylidene)-2,3-dihydrothiazole (D1i)

Yield: 60%, m.p.: 175 °C, IR (cm^−1^): 2935 (C=C–H stretching band), 2918 (C–H stretching band), 1587 (C=N stretching band), 1562 (C=C band), 1236 (C–N stretching band), 833 (2,4-disubstituted benzene out-of-plane deformation band).

^1^H-NMR (300 MHz, DMSO-*d*_6_): 1.57 (6H, s, piperidine-H), 2.16 (3H, s, methyl-H), 2.32 (6H, s, methyl-H), 3.04 (3H, s, methyl-H), 3.18–3.20 (4H, m, piperidine-H), 6.15 (1H, s, dihydrothiazole-H), 6.90–6.93 (2H, d, *J* = 8.94 Hz, Ar–H), 7.09–7.18 (3H, m, Ar–H), 7.66–7.69 (2H, d, *J* = 8.89 Hz, Ar–H). ^13^C-NMR (75 MHz, DMSO-*d*_6_): *δ* = 13.51, 15.21, 18.77, 23.85, 25.50, 31.64, 33.49, 49.35, 51.14, 100.27, 114.05, 116.20, 126.10, 128.02, 128.28, 128.76, 129.72, 131.84, 132.34, 137.59, 139.42, 139.50, 152.05, 154.40, 167.48. HRMS (*m*/*z*): [M + H]^+^ calcd for C_25_H_30_N_4_ S [M + H]^+^: 419.2264, found: 419.2255.

##### 4.1.5.10 3-Allyl-2-((1-(4-(piperidin-1-yl)phenyl)ethylidene)hydrazineylidene)-4-(*p*-tolyl)-2,3-dihydrothiazole (D2a)

Yield: 78%, m.p.: 129.4 °C, IR (cm^−1^): 2933 (C=C–H stretching band), 2787 (C–H stretching band), 1591 (C=N stretching band), 1504 (C=C band), 1234 (C–N stretching band), 815 (4-substituted benzene out-of-plane deformation band). ^1^H-NMR (300 MHz, DMSO-*d*_6_): 1.57 (6H, s, piperidine-H), 2.29 (3H, s, methyl-H), 2.34 (3H, s, methyl-H), 3.20 (4H, m, piperidine-H), 4.38 (2H, s, allyl-H), 4.90–4.96 (1H, d, *J* = 17.64 Hz, allyl-H), 5.08–5.12 (1H, d, *J* = 10.36 Hz, allyl-H), 5.76–5.87 (1H, m, allyl-H), 6.24 (1H, s, dihydrothiazole-H), 6.90–6.93 (2H, d, *J* = 8.64 Hz, Ar–H), 7.25–7.34 (4H, q, *J* = 10.44 Hz, Ar–H), 7.65–7.69 (2H, d, *J* = 8.55 Hz, Ar–H). ^13^C-NMR (75 MHz, DMSO-*d*_6_): *δ* = 14.42, 21.30, 24.42, 25.50, 47.99, 49.31, 99.69, 115.07, 116.96, 127.26, 128.42, 128.60, 128.99, 129.68, 133.26, 139.13, 140.60, 152.11, 154.95, 167.15. HRMS (*m*/*z*): [M + H]^+^ calcd for C_26_H_30_N_4_ S [M + H]^+^: 431.2264; found: 431.224.

##### 4.1.5.11 3-Allyl-4-(4-fluorophenyl)-2-((1-(4-(piperidin-1yl)phenyl)ethylidene)hydrazineylidene)-2,3-dihydrothiazole (D2b)

Yield: 65%, m.p.: 116 °C, IR (cm^−1^): 3076 (C=C–H stretching band), 2931 (C–H stretching band), 1591 (C=N stretching band), 1535 (C=C band), 1220 (C–N stretching band), 817 (4-substituted benzene out-of-plane deformation band).

^1^H-NMR (300 MHz, DMSO-*d*_6_): 1.60 (6H, s, piperidine-H), 2.30 (3H, s, methyl-H), 3.22 (4H, br.s, piperidine-H), 4.38–4.39 (2H, s, allyl-H), 4.91–4.96 (1H, d, *J* = 17.20 Hz, allyl-H), 5.07–5.11 (1H, d, *J* = 10.29 Hz, allyl-H), 5.78–5.86 (1H, m, allyl-H), 6.32 (1H, s, dihydrothiazole-H), 6.92–6.94 (2H, d, *J* = 8.71 Hz, Ar–H), 7.30–7.34 (2H, t, *J* = 8.70 Hz, Ar–H), 7.49–7.53 (2H, m, Ar–H), 7.67–7.69 (2H, d, *J* = 8.65 Hz, Ar–H). ^13^C-NMR (75 MHz, DMSO-*d*_6_): *δ* = 14.46, 24.43, 25.50, 47.97, 49.30, 100.49, 115.06, 116.02, 116.23, 117.06, 127.28, 128.55, 131.44, 131.53, 133.18, 139.45, 152.14, 155.13, 167.01. HRMS (*m*/*z*): [M + H]^+^ calcd for C_25_H_27_N_4_ FS [M + H]^+^: 435.2013; found: 435.2009.

##### 4.1.5.12 3-Allyl-4-(4-nitrophenyl)-2-((1-(4-(piperidin-1-yl)phenyl)ethylidene)hydrazineylidene)-2,3-dihydrothiazole (D2c)

Yield: 85%, m.p.: 155.6 °C, IR (cm^−1^): 3080 (C=C–H stretching band), 2933 (C–H stretching band), 1587 (C=N stretching band), 1552 (C=C band), 1236 (C–N stretching band), 819 (4-substituted benzene out-of-plane deformation band). ^1^H-NMR (300 MHz, DMSO-*d*_6_): 1.58 (6H, s, piperidine-H), 2.30 (3H, s, methyl-H), 3.21 (4H, br.s, piperidine-H), 4.47 (2H, s, allyl-H), 4.92–4.98 (1H, d, = 17.23 Hz, allyl-H), 5.09–5.13 (1H, d, *J* = 10.41 Hz, allyl-H), 5.79–5.90 (1H, m, allyl-H), 6.60–6.61 (1H, br. s, dihydrothiazole-H), 6.91–6.94 (2H, d, *J* = 7.89 Hz, Ar–H), 7.66–7.76 (4H, dd, *J*_1_ & *J*_2_ = 5.43, 7.23 Hz, Ar–H), 8.29–8.32 (2H, d, *J* = 7.20 Hz, Ar–H). ^13^C-NMR (75 MHz, DMSO-*d*_6_): *δ* = 14.53, 24.42, 25.48, 48.33, 49.25, 103.67, 115.03, 117.32, 124.35, 127.36, 128.32, 130.00, 133.10, 137.55, 138.74, 147.88, 152.20, 155.73, 167.07. HRMS (*m*/*z*): [M + H]^+^ calcd for C_25_H_27_N_5_ O_2_S [M + H]^+^: 462.1958; found: 462.1947.

##### 4.1.5.13 3-Allyl-4-(4-chlorophenyl)-2-((1-(4-(piperidin-1 yl)phenyl)ethylidene)hydrazineylidene)-2,3-dihydrothiazole (D2d)

Yield: 75%, m.p.: 145.2 °C, IR (cm^−1^): 3080 (C=C–H stretching band), 2931 (C–H stretching band), 1591 (C=N stretching band), 1556 (C=C band), 1232 (C–N stretching band), 817 (4-substituted benzene out-of-plane deformation band). ^1^H-NMR (300 MHz, DMSO-*d*_6_): 1.60 (6H, s, piperidine-H), 2.29 (3H, s, methyl-H), 3.22 (4H, br.s, piperidine-H), 3.80 (3H, s, methyl-H), 4.38 (2H, s, allyl-H), 4.93–4.97 (1H, d, = 17.20 Hz, allyl-H), 5.10–5.13 (1H, d, *J* = 10.26 Hz, allyl-H), 5.80–5.88 (1H, m, allyl-H), 6.22 (1H, s, dihydrothiazole-H), 6.92–6.94 (2H, d, *J* = 8.41 Hz, Ar–H), 7.02–7.04 (2H, d, *J* = 8.10 Hz, Ar–H), 7.37–7.39 (2H, d, *J* = 8.15 Hz, Ar–H), 7.67–7.69 (2H, d, *J* = 8.30 Hz, Ar–H). ^13^C-NMR (75 MHz, DMSO-*d*_6_): *δ* = 14.45, 24.43, 25.50, 47.96, 49.32, 55.72, 99.30, 114.54, 115.08, 116.95, 123.48, 127.26, 130.59, 133.30, 140.39, 152.11, 154.89, 160.27, 167.10. HRMS (*m*/*z*): [M + H]^+^ calcd for C_25_H_27_N_4_ S Cl [M + H]^+^: 451.1718; found: 451.1716.

##### 4.1.5.14 3-Allyl-4-(4-methoxyphenyl)-2-((1-(4-(piperidin-1-yl)phenyl)ethylidene)hydrazineylidene)-2,3-dihydrothiazole (D2e)

Yield: 65%, m.p.: 145.2 °C, IR (cm^−1^): 3080 (C=C–H stretching band), 2918 (C–H stretching band), 1587 (C=N stretching band), 1504 (C=C band), 1232 (C–N stretching band), 821 (4-substituted benzene out-of-plane deformation band). ^1^H-NMR (300 MHz, DMSO-*d*_6_): 1.60 (6H, s, piperidine-H), 2.29 (3H, s, methyl-H), 3.22 (4H, br.s, piperidine-H), 3.80 (3H, s, methyl-H), 4.38 (2H, s, allyl-H), 4.93–4.97 (1H, d, = 17.20 Hz, allyl-H), 5.10–5.13 (1H, d, *J* = 10.26 Hz, allyl-H), 5.80–5.88 (1H, m, allyl-H), 6.22 (1H, s, dihydrothiazole-H), 6.92–6.94 (2H, d, *J* = 8.41 Hz, Ar–H), 7.02–7.04 (2H, d, *J* = 8.10 Hz, Ar–H), 7.37–7.39 (2H, d, *J* = 8.15 Hz, Ar–H), 7.67–7.69 (2H, d, *J* = 8.30 Hz, Ar–H). ^13^C-NMR (75 MHz, DMSO-*d*_6_): *δ* = 14.45, 24.43, 25.50, 47.96, 49.32, 55.72, 99.30, 114.54, 115.08, 116.95, 123.48, 127.26, 130.59, 133.30, 140.39, 152.11, 154.89, 160.27, 167.10. HRMS (*m*/*z*): [M + H]^+^ calcd for C_26_H_30_N_4_O S [M + H]^+^: 447.2213; found: 447.2213.

##### 4.1.5.15 4-(3-Allyl-2-((1-(4-(piperidin-1-yl)phenyl)ethylidene)hydrazineylidene)-2,3-dihydrothiazol-4-yl)benzonitrile (D2f)

Yield: 90%, m.p.: 195.1 °C, IR (cm^−1^): 3379 (C=C–H tension band), 2931 (C–H stretching band), 2243 (nitirile-CN stretching band), 1595 (C=N stretching band), 1571 (C=C band), 1236 (C–N stretching band), 817 (4-substituted benzene out-of-plane deformation band). ^1^H-NMR (300 MHz, DMSO-*d*_6_): 1.58–1.59 (6H, br.s, piperidine-H), 2.29 (3H, s, methyl-H), 3.20–3.22 (4H, br.s, piperidine-H), 3.64–3.76 (2H, m, allyl-H), 4.96–5.01 (2H, m, allyl-H), 5.75–5.85 (1H, m, allyl-H), 6.91–6.93 (2H, d, *J* = 8.59 Hz, Ar–H), 7.18 (1H, s, dihydrothiazole-H), 7.67–7.69 (2H, d, *J* = 6.33 Hz, Ar–H), 7.72–7.75 (2H, d, *J* = 6.03 Hz, Ar–H), 7.87–7.89 (2H, d, *J* = 8.07 Hz, Ar–H). ^13^C-NMR (75 MHz, DMSO-*d*_6_): *δ* = 14.53, 24.42, 25.51, 41.98, 47.35, 49.24, 94.34, 111.56, 114.97, 116.88, 119.07, 127.40, 127.92, 128.38, 134.71, 148.20, 152.28, 156.87, 165.37. HRMS (*m*/*z*): [M + H]^+^ calcd for C_26_H_27_N_5_ S [M + H]^+^: 442.2060; found: 442.2051.

##### 4.1.5.16 3-Allyl-4-(2,4-dichlorophenyl)-2-((1-(4-(piperidin-1-yl)phenyl)ethylidene)hydrazineylidene)-2,3-dihydrothiazole (D2g)

Yield: 86%, m.p.: 115.5 °C, IR (cm^−1^): 3078 (C=C–H stretching band), 2935 (C–H stretching band), 1589 (C=N stretching band), 1573 (C=C band), 1236 (C–N stretching band), 823 (2,4-disubstituted benzene out-of-plane deformation band). ^1^H-NMR (300 MHz, DMSO-*d*_6_): 1.57(6H, s, piperidine-H), 2.28 (3H, s, methyl-H), 3.20–3.24(4H, br.s, piperidine-H), 3.67–3.71 (2H, br.s, allyl-H), 4.78–4.89 (2H, m, allyl-H), 5.71–5.82 (1H, m, allyl-H), 6.90–6.93 (2H, d, *J* = 8.8 Hz, Ar–H), 7.32 (1H, s, dihydrothiazole-H), 7.51–7.55 (1H, d, *J* = 8.5 Hz, Ar–H), 7.64–7.68 (3H, m, Ar–H), 7.91–7.94 (1H, d, *J* = 8.6 Hz, Ar–H). ^13^C-NMR (75 MHz, DMSO-*d*_6_): *δ* = 14.46, 24.42, 25.52, 31.15, 47.03, 49.28, 92.92, 115.00, 116.78, 127.31, 127.61, 128.52, 130.95, 131.49, 133.48, 134.50, 134.59, 138.13, 152.20, 156.14, 165.60.

##### 4.1.5.17 3-Allyl-4-(3,4-dichlorophenyl)-2-(((*E*)-1-(4-(piperidin-1-yl)phenyl)ethylidene)hydrazineylidene)-2,3-dihydrothiazole (D2h)

Yield: 65%, m.p.: 115.5 °C, IR (cm^−1^): 3082 (C=C–H stretching band), 2924 (C–H stretching band), 1593 (C=N stretching band), 1504 (C=C band), 1232 (C–N stretching band), 821 (3,4-disubstituted phenyl out-of-plane deformation band). ^1^H-NMR (300 MHz, DMSO-*d*_6_): 1.60 (6H, s, piperidine-H), 2.30 (3H, s, methyl-H), 3.22 (4H, br.s, piperidine-H), 4.42–4.43 (2H, br.s, allyl-H), 4.94–4.99 (1H, d, *J* = 17.20 Hz, allyl-H), 5.12–5.15 (1H, d, *J* = 10.34 Hz, allyl-H), 5.81–5.90 (1H, m, allyl-H), 6.48 (1H, s, dihydrothiazole-H), 6.91–6.94 (2H, d, *J* = 8.32 Hz, Ar–H), 7.45–7.48 (1H, d, *J* = 8.30 Hz, Ar–H), 7.67–7.69 (2H, d, *J* = 8.25 Hz, Ar–H), 7.73–7.76 (2H, d, *J* = 7.50 Hz, Ar–H). ^13^C-NMR (75 MHz, DMSO-*d*_6_): *δ* = 14.50, 24.43, 25.49, 48.20, 49.28, 102.18, 115.04, 117.13, 127.32, 128.43, 129.20, 130.85, 131.13, 131.76, 131.90, 132.33, 138.10, 152.18, 155.47, 166.94. HRMS (*m*/*z*): [M + H]^+^ calcd for C_25_H_26_N_4_ S Cl_2_ [M + H]^+^: 485.1328; found: 485.1310.

##### 4.1.5.18 3-Allyl-4-(2,4-dimethylphenyl)-2-(((*E*)-1-(4-(piperidin-1-yl)phenyl)ethylidene)hydrazineylidene)-2,3-dihydrothiazole (D2i)

Yield: 70%, m.p.: 176.6 °C, IR (cm^−1^): 3100 (C=C–H stretching band), 2935 (C–H stretching band), 1591 (C=N stretching band), 1552 (C=C band), 1236 (C–N stretching band), 823 (2,4-disubstituted phenyl out-of-plane deformation band). ^1^H-NMR (300 MHz, DMSO-*d*_6_): 1.57 (6H, s, piperidine-H), 2.15 (3H, s, methyl-H), 2.29 (3H, s, methyl-H), 2.32 (3H, s, methyl-H), 3.20 (4H, br.s, piperidine-H), 4.13 (2H, s, allyl-H), 4.80–4.86 (1H, d, *J* = 17.2 Hz, allyl-H), 5.02–5.05 (1H, d, *J* = 10.2 Hz, allyl-H), 5.65–5.78 (1H, m, allyl-H), 6.14 (1H, s, dihydrothiazole-H), 6.90–6.93 (2H, d, *J* = 8.52 Hz, Ar–H), 7.06–7.15 (3H, m, Ar–H), 7.66–7.69 (2H, d, *J* = 8.47 Hz, Ar–H). ^13^C-NMR (75 MHz, DMSO-*d*_6_): *δ* = 14.42, 19.72, 21.28, 24.43, 25.50, 47.32, 49.31, 99.55, 115.09, 117.23, 126.88, 127.22, 127.82, 128.66, 130.89, 131.28, 132.73, 137.69, 138.80, 139.49, 152.08, 154.74, 166.57. HRMS (*m*/*z*): [M + H]^+^ calcd for C_27_H_32_N_4_ S [M + H]^+^: 445.2420, found: 445.2427.

##### 4.1.5.19 3-Ethyl-2-((1-(4-(piperidin-1-yl)phenyl)ethylidene)hydrazineylidene)-4-(*p*-tolyl)-2,3-dihydrothiazole (D3a)

Yield: 75%, m.p.: 164.2 °C, IR (cm^−1^): 3100 (C=C–H stretching band), 2924 (C–H stretching band), 1593 (C=N stretching band), 1585 (C=C band), 1234 (C–N stretching band), 827 (4-substituted phenyl out-of-plane deformation band). ^1^H-NMR (300 MHz, DMSO-*d*_6_): *δ* = 1.10–1.14 (3H, t, *J* = 6.88 Hz, methyl-H), 1.56 (6H, s, piperidine-H), 2.31 (3H, s, methyl-H), 2.35 (3H, s, methyl-H), 3.20 (4H, m, piperidine-H), 3.73–3.80 (2H, q, *J* = 6.6 Hz, methylene-H), 6.19 (1H, s, dihydrothiazole-H), 6.90–6.93 (2H, d, *J* = 8.78 Hz, Ar–H), 7.27–7.35 (4H, q, *J* = 6.96 Hz, Ar–H), 7.66–7.69 (2H, d, *J* = 8.70 Hz, Ar–H). ^13^C-NMR (75 MHz, DMSO-*d*_6_): *δ* = 13.47, 14.42, 21.32, 24.42, 25.50, 41.00, 49.32, 99.68, 100.96, 115.10, 126.13, 126.22, 127.21, 128.63, 128.70, 129.07, 139.13, 140.48, 152.07, 154.53, 167.21. HRMS (*m*/*z*): [M + H]^+^ calcd for C_25_H_30_N_4_ S [M + H]^+^: 419.2264; found: 419.2251.

##### 4.1.5.20 3-Ethyl-4-(4-fluorophenyl)-2-((1-(4-(piperidin-1-yl)phenyl)ethylidene)hydrazineylidene)-2,3-dihydrothiazole (D3b)

Yield: 70%, m.p.: 150 °C, IR (cm^−1^): 3109 (C=C–H stretching band), 2937 (C–H stretching band), 1583 (C=N stretching band), 1548 (C=C band), 1238 (C–N stretching band), 815 (4-substituted phenyl out-of-plane deformation band). ^1^H-NMR (300 MHz, DMSO-*d*_6_): *δ* = 1.12–1.15 (3H, t, *J* = 6.95 Hz, methyl-H), 1.57–1.61 (6H, br.s, piperidine-H), 2.33 (3H, s, methyl-H), 3.20–3.22 (4H, q, *J* = 6.87 Hz, piperidine-H), 3.75–3.79 (2H, q, *J* = 7.19 Hz, methylene-H), 6.27 (1H, s, dihydrothiazole-H), 6.91–6.94 (2H, d, *J* = 8.79 Hz, Ar–H), 7.32–7.36 (2H, t, *J* = 8.74 Hz, Ar–H), 7.52–7.55 (2H, m, Ar–H), 7.67–7.70 (2H, d, *J* = 8.69 Hz, Ar–H). ^13^C-NMR (75 MHz, DMSO-*d*_6_): *δ* = 13.58, 14.44, 24.43, 25.50, 49.33, 56.49, 100.41, 115.09, 116.15, 116.37, 127.25, 127.96, 128.66, 131.52, 131.60, 139.35, 152.11, 154.74, 161.32, 164.12, 167.04. HRMS (*m*/*z*): [M + H]^+^ calcd for C_24_H_27_N_4_ FS [M + H]^+^: 423.2013; found: 423.1996.

##### 4.1.5.21 3-Ethyl-4-(4-nitrophenyl)-2-((1-(4-(piperidin-1 yl)phenyl)ethylidene)hydrazineylidene)-2,3-dihydrothiazole (D3c)

Yield: 89%, m.p.: 166.5 °C, IR (cm^−1^): 3124 (C=C–H stretching band), 2943 (C–H stretching band), 1589 (C=N stretching band), 1504 (C=C band), 1234 (C–N stretching band), 823 (4-substituted phenyl out-of-plane deformation band). ^1^H-NMR(300 MHz, DMSO-*d*_6_): 1.16–1.19 (3H, t, *J* = 6.93 Hz, methyl-H), 1.59–1.60 (6H, s, piperidine-H), 2.09 (3H, s, methyl-H), 3.22–3.23 (4H, s, piperidine-H), 3.82–3.87 (2H, q, *J* = 6.93 Hz, methylene-H), 6.55 (1H, s, dihydrothiazole-H), 6.93–6.95 (2H, d, *J* = 8.72 Hz, Ar–H), 7.68–7.70 (2H, d, *J* = 8.62 Hz, Ar–H), 7.78–7.80 (2H, d, *J* = 8.52 Hz, Ar–H), 8.33–8.35 (2H, d, *J* = 8.65 Hz, Ar–H). ^13^C-NMR (75 MHz, DMSO-*d*_6_): *δ* = 13.51, 14.53, 24.43, 25.49, 49.27, 103.52, 115.06, 124.47, 127.33, 128.42, 130.11, 137.73, 138.63, 152.18, 155.37, 167.12. HRMS (*m*/*z*): [M + H]^+^ calcd for C_24_H_27_N_5_ O_2_S [M + H]^+^: 450.1958; found: 450.1949.

##### 4.1.5.22 4-(4-Chlorophenyl)-3-ethyl-2-((1-(4-(piperidin-1-yl)phenyl)ethylidene)hydrazineylidene)-2,3-dihydrothiazole (D3d)

Yield: 74%, m.p.: 156.5 °C, IR (cm^−1^): 3080 (C=C–H stretching band), 2931 (C–H stretching band), 1587 (C=N stretching band), 1562 (C=C band), 1236 (C–N stretching band), 812 (4-substituted phenyl out-of-plane deformation band). ^1^H-NMR (300 MHz, DMSO-*d*_6_): 1.13–1.16 (3H, t, *J* = 6.82 Hz, methyl-H), 1.60 (6H, s, piperidine-H), 2.33 (3H, s, methyl-H), 3.22 (4H, br.s, piperidine-H), 3.77–3.79 (2H, q, *J* = 6.92 Hz, methylene-H), 6.32 (1H, s, dihydrothiazole-H), 6.92–6.94 (2H, d, *J* = 8.65 Hz, Ar–H), 7.50–7.58 (4H, m, Ar–H), 7.67–7.69 (2H, d, *J* = 8.54 Hz, Ar–H). ^13^C-NMR (75 MHz, DMSO-*d*_6_): *δ* = 13.47, 14.44, 24.42, 25.49, 41.12, 49.01, 49.30, 100.95, 113.91, 115.08, 127.26, 128.59, 129.34, 130.32, 130.98, 134.34, 139.21, 152.11, 154.85, 167.10. HRMS (*m*/*z*): [M + H]^+^ calcd for C_24_ H_27_ N_4_ S Cl [M + H]^+^: 439.1718; found: 439.1713.

##### 4.1.5.23 3-Ethyl-4-(4-methoxyphenyl)-2-((1-(4-(piperidin-1-yl)phenyl)ethylidene)hydrazineylidene)-2,3-dihydrothiazole (D3e)

Yield: 90%, m.p.: 148.1 °C, IR (cm^−1^): 3101 (C=C–H stretching band), 2929 (C–H stretching band), 1587 (C=N stretching band), 1556 (C=C band), 1238 (C–N stretching band), 812 (4-substituted phenyl out-of-plane deformation band).

^1^H-NMR(300 MHz, DMSO-*d*_6_): 1.12–1.16 (3H, t, *J* = 6.86 Hz, methyl-H), 1.60 (6H, s, piperidine-H), 2.33 (3H, s, methyl-H), 3.22 (4H, br.s, piperidine-H), 3.77 (2H, q, *J* = 6.98 Hz, methylene-H), 3.78–3.8116 (3H, s, methyl-H), 6.16 (1H, s, dihydrothiazole-H), 6.92–6.94 (2H, d, *J* = 8.47 Hz, Ar–H), 7.04–7.06 (2H, d, *J* = 8.08 Hz, Ar–H), 7.39–7.41 (2H, d, *J* = 8.03 Hz, Ar–H), 7.67–7.69 (2H, d, *J* = 8.36 Hz, Ar–H). ^13^C-NMR (75 MHz, DMSO-*d*_6_): *δ* = 13.46, 14.41, 24.43, 25.51, 40.95, 49.35, 55.72, 99.29, 114.66, 115.10, 123.70, 127.22, 128.76, 130.65, 140.29, 152.08, 154.49, 160.24, 167.15. HRMS (*m*/*z*): [M + H]^+^ calcd for C_25_H_30_N_4_O S [M + H]^+^: 435.2213; found: 435.2193.

##### 4.1.5.24 4-(-3-Ethyl-2-((-1-(4-(piperidin-1-yl)phenyl)ethylidene)hydrazineylidene)-2,3-dihydrothiazol-4-yl)benzonitrile (D3f)

Yield: 75%, m.p.: 166.3 °C, IR (cm^−1^): 3074 (C=C–H stretching band), 2929 (C–H stretching band), 2225 (nitrile-CN stretching), 1585 (C=N stretching band), 1556 (C=C band), 1238 (C–N stretching band), 819 (4-substituted phenyl out-of-plane deformation band), ^1^H-NMR(300 MHz, DMSO-*d*_6_): 1.15 (3H, br.s, methyl-H), 1.60 (6H, s, piperidine-H), 2.34 (3H, s, methyl-H), 3.22 (4H, br.s, piperidine-H), 3.80–3.82 (2H, q, *J* = 5.61 Hz, methylene-H), 6.48 (1H, s, dihydrothiazole-H), 6.93–6.95 (2H, d, *J* = 7.79 Hz, Ar–H), 7.69 (4H, br.s, Ar–H), 7.97–7.99 (2H, d, *J* = 6.95 Hz, Ar–H), ^13^C-NMR (75 MHz, DMSO-*d*_6_): *δ* = 12.64, 13.66, 14.33, 15.36, 23.79, 25.48, 41.40, 49.26, 51.10,101.54, 112.03, 113.98, 116.14, 118.94, 119.00, 126.21, 128.39, 128.68, 130.86, 132.17, 134.42, 136.04, 138.96, 152.15, 155.21, 167.15, HRMS (*m*/*z*): [M + H]^+^ calcd for C_25_H_27_N_5_ S [M + H]^+^: 430.2060; found: 430.2040.

##### 4.1.5.25 4-(2,4-Dichlorophenyl)-3-ethyl-2-((1-(4-(piperidin-1 yl)phenyl)ethylidene)hydrazineylidene)-2,3-dihydrothiazole (D3g)

Yield: 80%, m.p.: 163.7 °C, IR (cm^−1^): 3100 (C=C–H stretching band), 2922 (C–H stretching band), 1589 (C=N stretching band), 1573 (C=C band), 1234 (C–N stretching band), 812 (2,4-disubstituted phenyl out-of-plane deformation band), ^1^H-NMR(300 MHz, DMSO-*d*_6_): 1.02–1.06 (3H, t, *J* = 6.90 Hz, methyl-H), 1.60 (6H, br.s, piperidine-H), 2.30 (3H, s, methyl-H), 3.21 (4H, s, piperidine-H), 3.17–3.20 (2H, m, methylene-H), 6.91–6.93 (2H, d, *J* = 8.67 Hz, Ar–H), 7.24 (1H, s, dihydrothiazole-H), 7.54–7.56 (1H, m, Ar–H), 7.66–7.69 (3H, m, Ar–H), 7.93–7.96 (2H, d, *J* = 8.58 Hz, Ar–H), ^13^C-NMR (75 MHz, DMSO-*d*_6_): *δ* = 13.46, 14.41, 24.43, 25.53, 49.29, 93.35, 115.02, 127.26, 127.75, 128.63, 128.84, 131.03, 131.55, 133.26, 134.50, 138.37, 152.16, 155.62, 165.54, HRMS (*m*/*z*): [M + H]^+^ calcd for C_24_H_26_N_4_ S Cl_2_ [M + H]^+^: 473.1328; found: 473.1319.

##### 4.1.5.26 4-(3,4-Dichlorophenyl)-3-ethyl-2-((1-(4-(piperidin-1 yl)phenyl)ethylidene)hydrazineylidene)-2,3-dihydrothiazole (D3h)

Yield: 73%, m.p.: 138.36 °C, IR (cm^−1^): 3099 (C=C–H stretching band), 2935 (C–H stretching band), 1591 (C=N stretching band), 1541 (C=C band), 1236 (C–N stretching band), 819 (3,4-disubstituted phenyl out-of-plane deformation band), ^1^H-NMR(300 MHz, DMSO-*d*_6_): 1.11–1.16 (3H, t, *J* = 6.99 Hz, methyl-H), 1.57 (6H, br.s, piperidine-H), 2.32 (3H, s, methyl-H), 3.21 (4H, br.s, piperidine-H), 3.76–3.82 (2H, q, *J* = 6.83 Hz, methylene-H), 6.41 (1H, s, dihydrothiazole-H), 6.90–6.93 (2H, d, *J* = 7.49 Hz, Ar–H), 7.46–7.49 (1H, d, *J* = 8.28 Hz, Ar–H), 7.66–7.69 (2H, d, *J* = 8.63 Hz, Ar–H), 7.74–7.78 (2H, d, *J* = 13.4 Hz, Ar–H), ^13^C-NMR (75 MHz, DMSO-*d*_6_): *δ* = 13.48, 14.46, 24.42, 25.50, 41.99, 49.30, 94.27, 102.03, 114.80, 115.06, 127.28, 128.07, 128.53, 129.31, 131.03, 131.40, 131.97, 132.38, 137.94, 144.34, 152.13, 152.23, 155.03, 156.30, 165.36, 166.97, HRMS (*m*/*z*): [M + H]^+^ calcd for C_24_H_26_N_4_ S Cl_2_ [M + H]^+^: 473.1328; found: 473.1319.

##### 4.1.5.27 4-(2,4-Dimethylphenyl)-3-ethyl-2-((1-(4-(piperidin-1-yl)phenyl)ethylidene)hydrazineylidene)-2,3-dihydrothiazole (D3i)

Yield: 68%, m.p.: 158.9 °C, IR (cm^−1^): 3115 (C=C–H stretching band), 2935 (C–H stretching band), 1595 (C=N stretching band), 1573 (C=C stretching band), 1236 (C–N stretching band), 819 (2,4-disubstituted phenyl out-of-plane deformation band), ^1^H-NMR (300 MHz, DMSO-*d*_6_): 0.98–1.02 (3H, t, *J* = 6.83 Hz, methyl-H), 1.55–1.57 (6H, d, piperidine-H), 2.16 (3H, s, methyl-H), 2.26–2.31 (6H, s, methyl-H), 3.20 (4H, d, piperidine-H), 3.35 (2H, s, methylene-H), 6.11 (1H, s, dihydrothiazole-H), 6.84–6.93 (2H, m, Ar–H), 7.11–7.18 (3H, m, Ar–H), 7.66–7.69 (1H, d, *J* = 8.95, Ar–H), 7.79–7.82 (1H, d, *J* = 8.96, Ar–H), ^13^C-NMR (75 MHz, DMSO-*d*_6_): *δ* = 13.56, 14.46, 24.42, 25.50, 48.27, 49.02, 49.33, 99.38, 113.91, 114.48, 115.11, 126.56, 127.06, 127.18, 127.61, 128.10, 128.78, 130.76, 131.03, 131.30, 132.57, 133.36, 135.80, 137.67, 138.61, 152.37, 154.35, 166.60, HRMS (*m*/*z*): [M + H]^+^ calcd for C_26_H_32_N_4_ S [M + H]^+^: 433.2423; found: 433.2420.

### 4.2. Determination of biological activity

The potential inhibitory effects of the obtained molecules on the AChE and BChE enzymes was examined using a variation of the Ellman method.^31,33,41–43^ Distilled water from the Millipore/Milli-Q purification apparatus was utilized at every stage of the biological activity investigations during the evaluation process. In less than a week, all the solutions required for the biological investigation were meticulously prepared from scratch. A “BioTek-Precision Power (United States)” robotic pipetting system was utilized to distribute the prepared solutions and apply the test compounds and enzyme–substrate solutions to 96-well plates. The “BioTek-Synergy H1 Microplate Reader (United States)” was employed to establish and follow the enzyme protocol for spectrophotometric measurements. Solutions of the compounds were prepared at two different concentrations, 10^−3^ M and 10^−4^ M, using dimethylsulfoxide (DMSO) as 2% solution, and these were used in biological activity studies. Activity values were determined, ranging from 0% to 100% for each compound.

### 4.3. Cytotoxicity effect studies

For cytotoxicity tests, the NIH/3T3 mouse embryonic fibroblast cell line (ATCC® CRL-1658TM, London, UK) was employed. The supplier's recommendation served as the basis for the NIH/3T3 cells' incubation time. In each well of the 96-well plates, 1104 NIH/3T3 cells were planted. The MTT assay was performed in compliance with the previously mentioned standards.^29,35^

### 4.4. Computational docking analysis

A method based on *in silico* molecular docking was employed to identify the potential interaction and binding sites of the D1f compound, which emerged as the most effective inhibitor against AChE and BChE, enzymes among synthesized derivatives in this thesis. Protein–ligand interaction analysis for the AChE enzyme was conducted using its crystal structure (PDB: 4EY7) as a ref. 36. First, the “Protein Preparation Wizard” protocol in Schrödinger Suite 2015 (ref. 44) was utilized to prepare the crystal structure for docking studies. The potential charges of the atoms on the ionizable residues were then automatically ascertained under the given environmental conditions, and bond lengths were adjusted using the OPLS 2005 force field. Compounds for molecular docking studies were prepared using the LigPrep 3.8 module (L. Schrödinger, LigPrep, Version 3.8, Schrödinger, LLC, New York, NY, USA, (2016)).^45^ The grid generation and docking studies were performed using Glide 7.1 (L. Schrödinger, Glide, Version 7.1, Schrödinger, LLC: New York, NY, USA, (2016)) with single precision (SP).^46^

### 4.5. Molecular dynamics studies

Molecular dynamics (MD) simulations were used to evaluate the time-dependent stability of compound D1f within the drug-receptor complex over 100 ns. In the dynamic study, simulations were performed using Desmond (Schrödinger Suite 2020-3) with the TIP3P water model and OPLS3e force field (Impact S. LLC, New York, NY, 2016; Prime, Schrödinger, LLC, New York, NY, 2020. Google Scholar; Schrödinger Release 2020-3, Maestro, Schrödinger, LLC, New York, NY, USA, 2020). In order to neutralize the system and represent the physiological concentration of monovalent ions, the simulations incorporated a final salt concentration of 0.15 M with Na^+^ and Cl^−^ ions.^47^ The simulations were conducted at a constant temperature of 310.55 K and a pressure of 1.01325 bar using the NPT ensemble class. The equations of motion were integrated using the RESPA integrator with NH thermostats maintaining constant temperature and the MTK method controlling pressure. The PME approach was utilized to compute long-range electrostatic interactions, while 9.0 Å was established as the threshold for van der Waals and short-range electrostatic interactions. System equilibration was achieved using Desmond's default protocol, involving a series of constrained minimization and MD simulations to gradually relax the system. After setting up the system, MD simulations were conducted with the specified parameters. The radius of gyration (*R*_g_), and root mean square deviation (RMSD) values were calculated using the Desmond application.

### 4.6. Prediction of physicochemical and pharmacokinetics

The determination of physicochemical and pharmacokinetic properties of the synthesized compounds was performed using computational methods. pkCSM, a web-based application from Cambridge (available at http://biosig.unimelb.edu.au/pkcsm/prediction),^48^ was used to predict pharmacokinetic parameters and the Drug Metabolism and Excretion (DMET) profile (“pkCSM). The smiles of the compounds were obtained from ChemDraw 17.0 software.

The pkCSM online tool (available at https://biosig.lab.uq.edu.au/pkcsm/prediction) offers insights into various pharmacokinetic parameters. It also evaluates toxicity risks such as organ toxicity. Furthermore, the pharmacological characteristics and drug-likeness of the compounds were assessed using the SwissADME online tool (available at http://www.swissadme.ch/).^49^

## Author contributions

Sazan Haji Ali: writing – original draft, methodology, investigation, formal analysis, conceptualization, Derya Osmaniye: software, methodology, formal analysis, conceptualization and Zafer Asim Kaplancikli: review & editing, conceptualization.

## Conflicts of interest

The authors declare no conflicts of interest.

## Supplementary Material

RA-015-D5RA05755H-s001

## Data Availability

All data supporting this study are provided in the supplementary information (SI). Supplementary information is available. See DOI: https://doi.org/10.1039/d5ra05755h.
