# Supplementary material for: Synthesis and biological evaluation of novel hydrazone derivatives for the treatment of Alzheimer's disease
Source: RSC Adv. 2025 Nov 21;15(53):45729–43. doi: 10.1039/d5ra05755h (PMC12637020; doi:10.1039/d5ra05755h)
Supplement: RA-015-D5RA05755H-s001 [file RA-015-D5RA05755H-s001.pdf]

Supplementary Information (SI) for RSC Advances.
This journal is © The Royal Society of Chemistry 2025

Synthesis and Biological Evaluation of Novel Hydrazone Derivatives for The Treatment of Alzheimer's Disease

Sazan Haji Ali [1], Derya Osmaniye[2,3] Zafer Asım Kaplancıklı [2]

[1]Department of Pharmaceutical Chemistry, College of Pharmacy, Hawler Medical University, Erbil 44000, Iraq

[2]Department of Pharmaceutical Chemistry, Faculty of Pharmacy, Anadolu University, Eski¸sehir 26470, Turkey

[3]Central Research Laboratory, Faculty of Pharmacy, Anadolu University, Eskişehir 26470, Turkey

Item	Value
Acquired Date&Time	2.05.2024 13:59:59
Acquired by	System Administrator
Filename	C:\Users\dopnalab\Desktop\MASAÜSTÜ\sazan\hic\hic-1a1.ispd
Spectrum name	hic-1a1
Sample name	hic-1a
Sample ID	
Option	
Comment	
No. of Scans	30
Resolution	4 [cm-1]
Apodization	Happ-Genzel

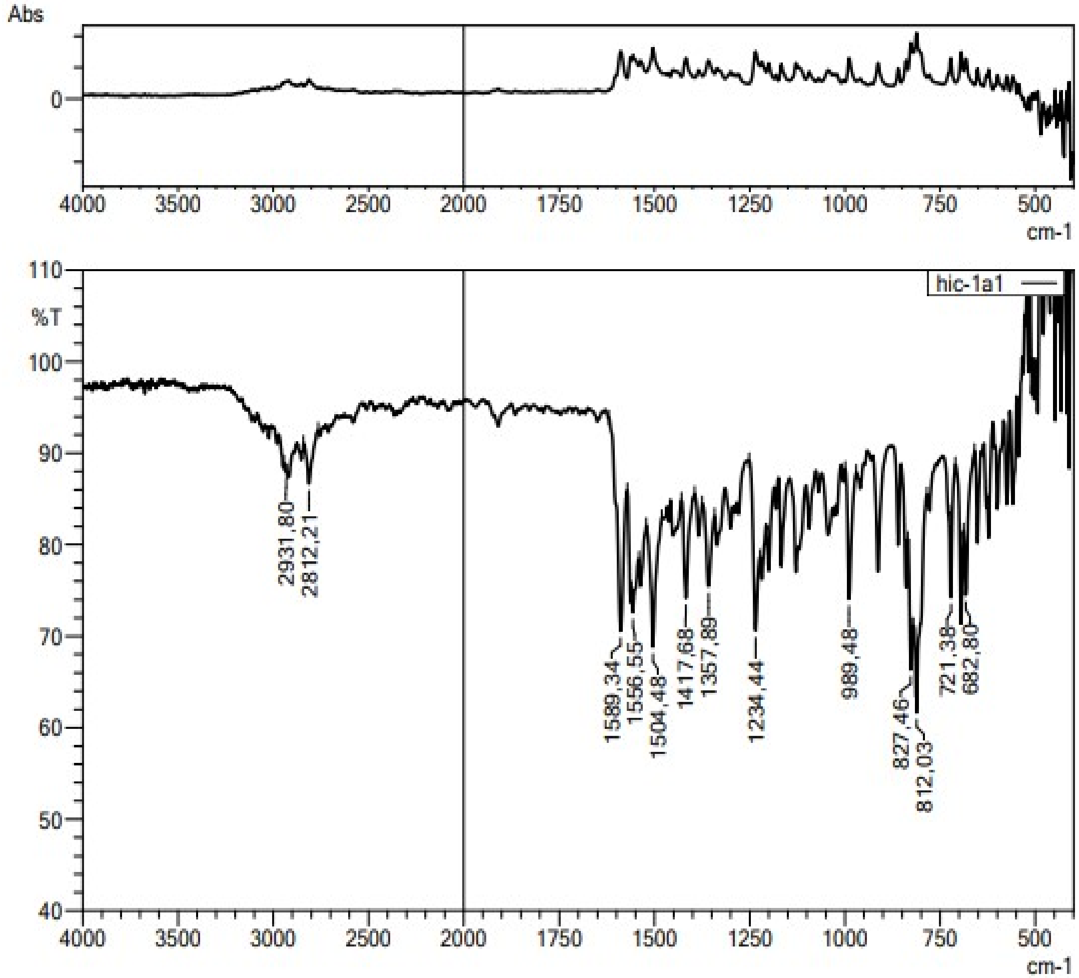

Figure 1S. IR spectrum of **D1a**

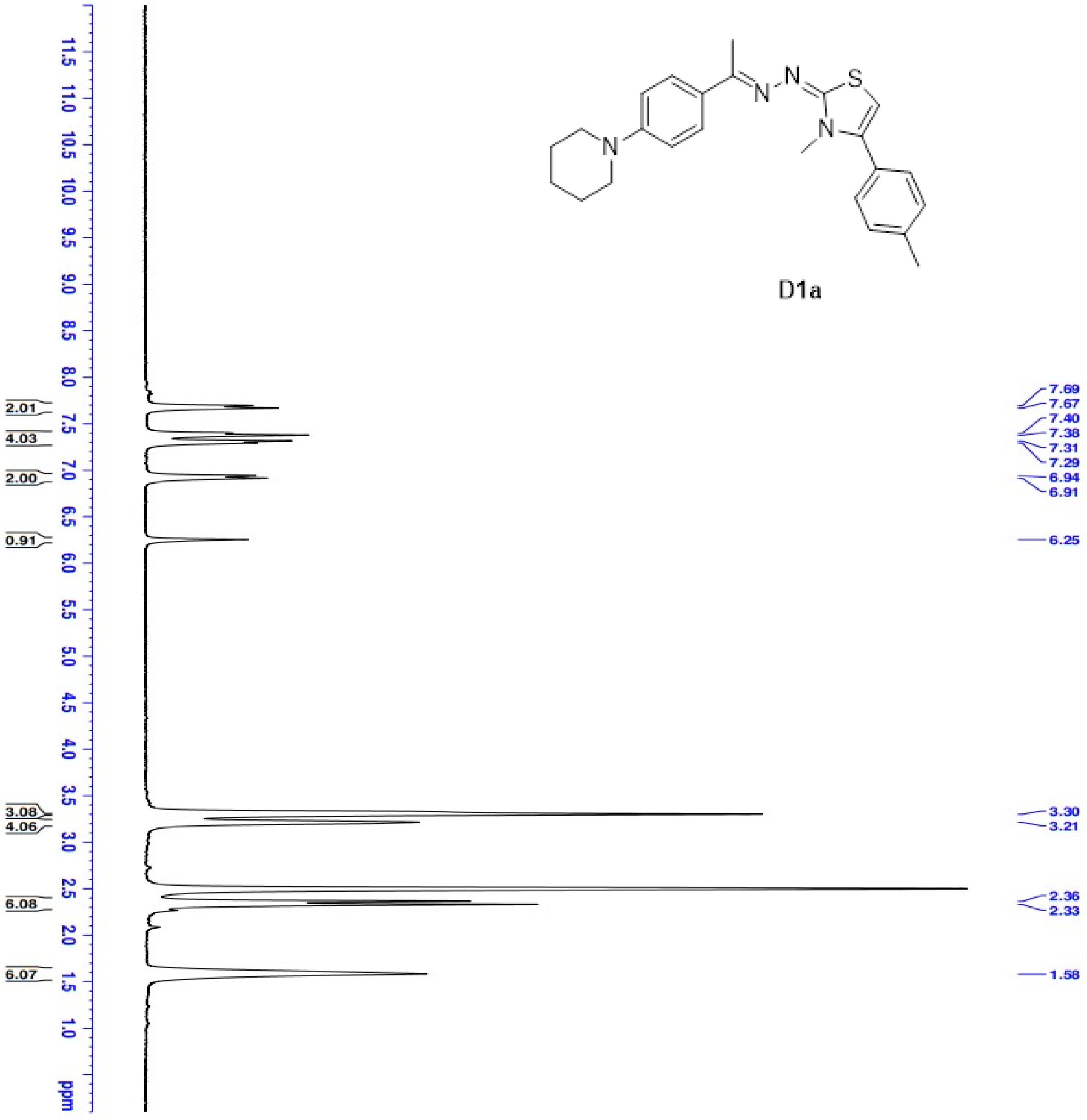
11.5 11.0 10.5 10.0 9.5 9.0 8.5 8.0 7.5 7.0 6.5 6.0 5.5 5.0 4.5 4.0 3.5 3.0 2.5 2.0 1.5 1.0 ppm
2.01
4.03
2.00
0.91
3.08
4.06
6.08
6.07
7.69
7.67
7.40
7.38
7.31
7.29
6.94
6.91
6.25
3.30
3.21
2.36
2.33
1.58

D1a

Current Data Parameters
NAME HIC-1As
EXPNO 5
PROCNO 1
F2 - Acquisition Parameters
Date_ 20240509
Time 15.38
INSTRUM FOURIER300
PROBHD 5 mm DUL 13C-1
PULPROG zg
TD 16384
SOLVENT DMSO
NS 16
DS 0
SWH 6103.516 Hz
FIDRES 0.372529 Hz
AQ 1.3421173 sec
RG 3.981
DW 81.920 usec
DE 6.50 usec
TE 296.1 K
D1 3.00000000 sec
TD0 1
======== CHANNEL f1 ========
SFO1 300.1818537 MHz
NUC1 1H
P1 13.00 usec
PLW1 10.00000000 W
F2 - Processing parameters
SI 65536
SF 300.1800000 MHz
WDW EM
SSB 0
LB 0.30 Hz
GB 0
PC 1.00

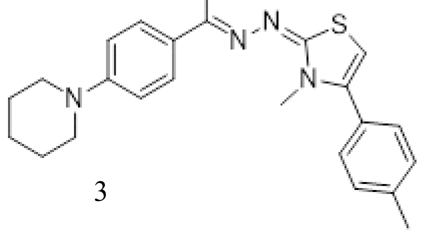

D1a

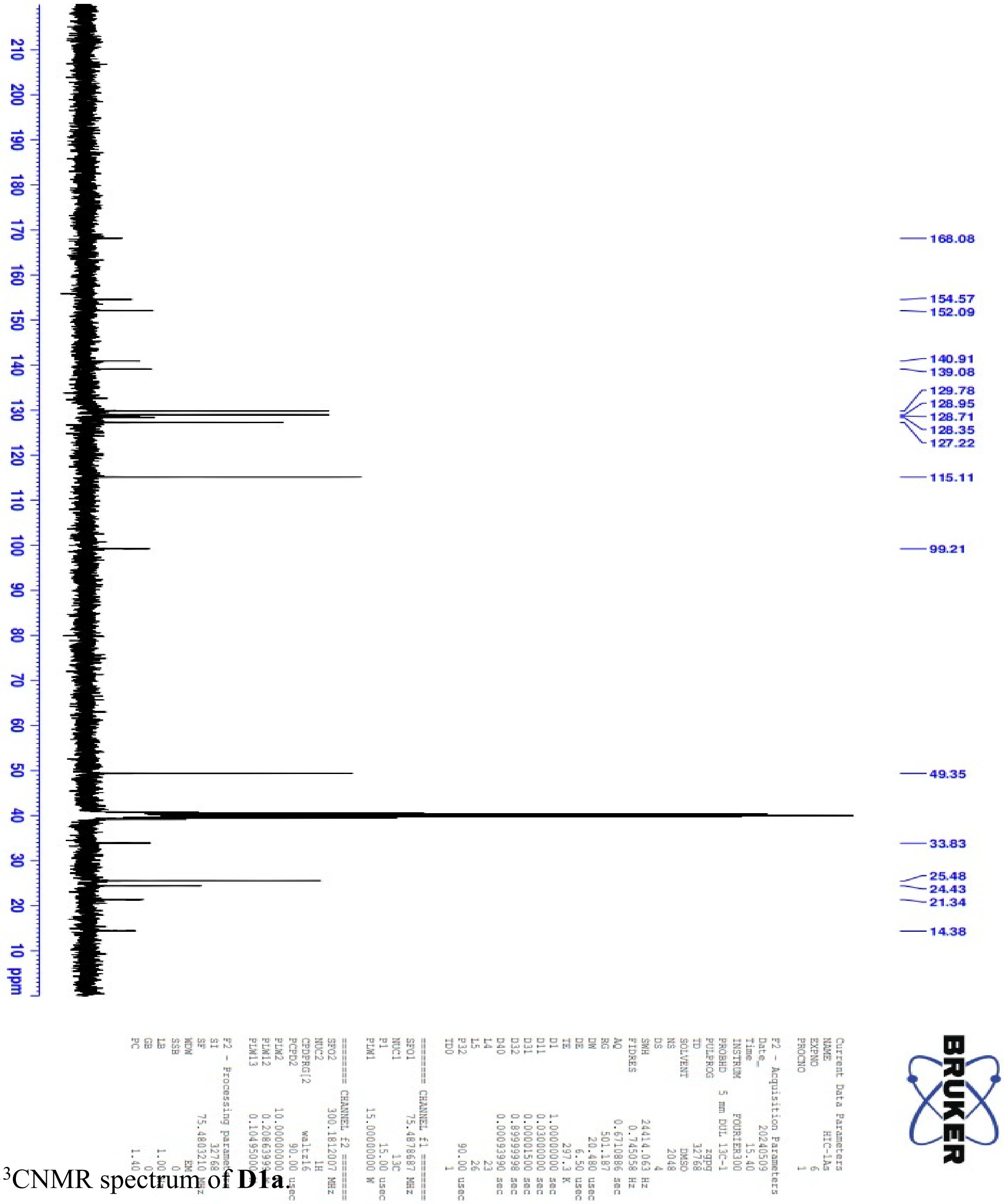

Figure 3S. ^{13}CNMR spectrum of **D1a**.

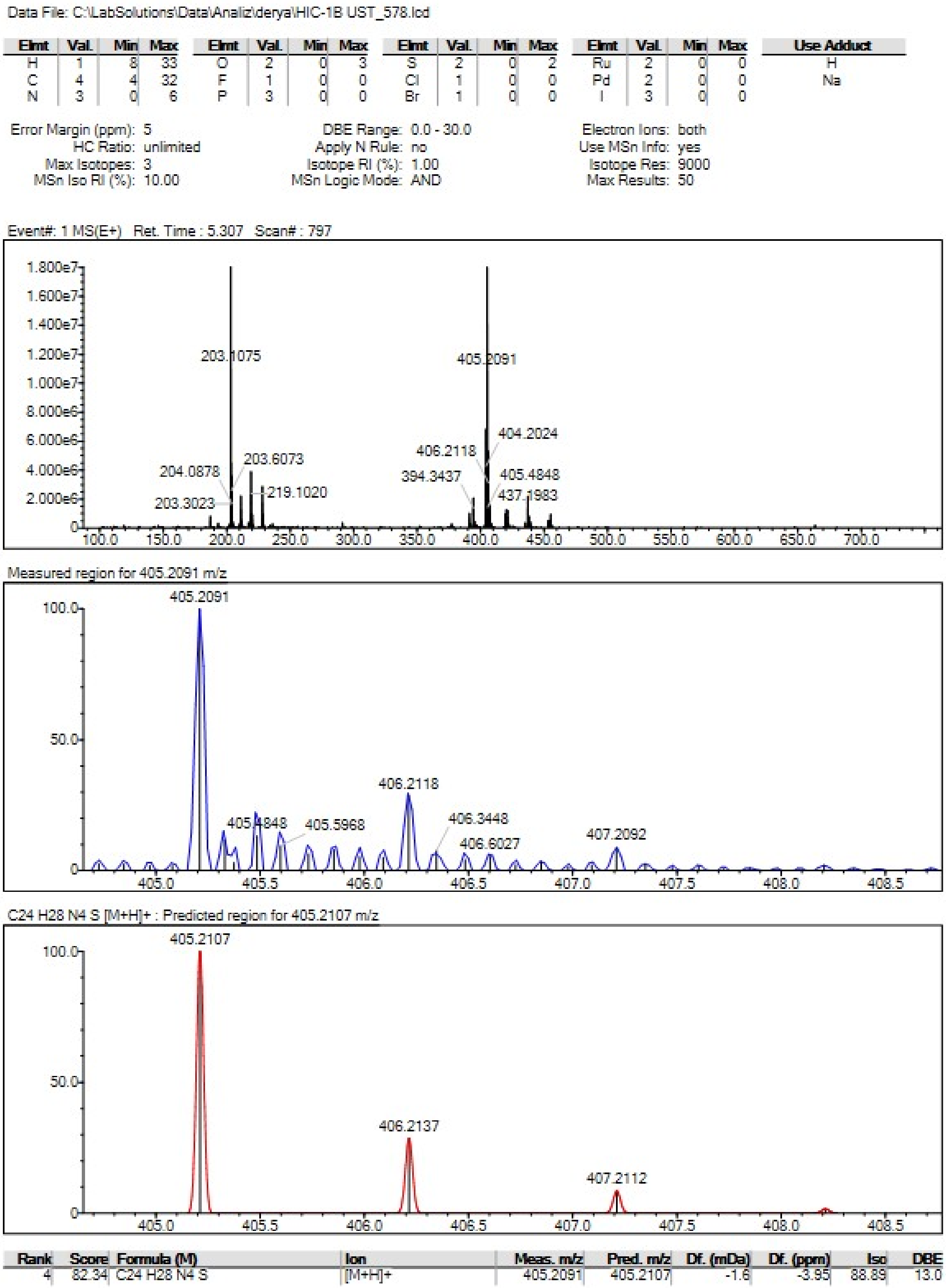

Figure 4S. HRMS spectrogram of **D1a**.

	Item	Value
	Acquired Date&Time	2.05.2024 14:07:25
	Acquired by	System Administrator
	Filename	C:\Users\dopnalab\Desktop\MASAÜSTÜ\sazan\hic\hic-1b1.ispd
	Spectrum name	hic-1b1
	Sample name	hic-1b
	Sample ID	
	Option	
	Comment	
	No. of Scans	30
	Resolution	4 [cm-1]
	Apodization	Happ-Genzel

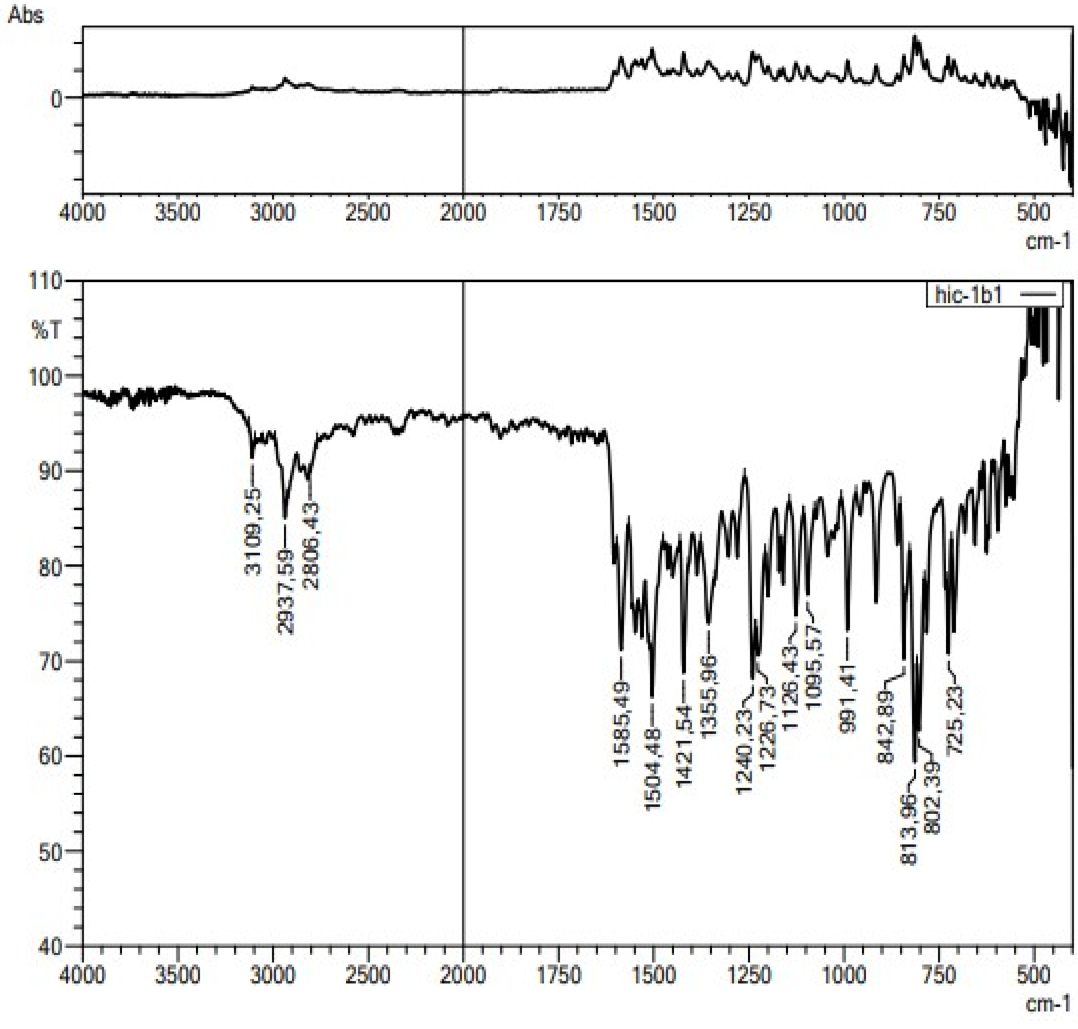

Figure 5S. IR fingerprint of **D1b.**F. 50

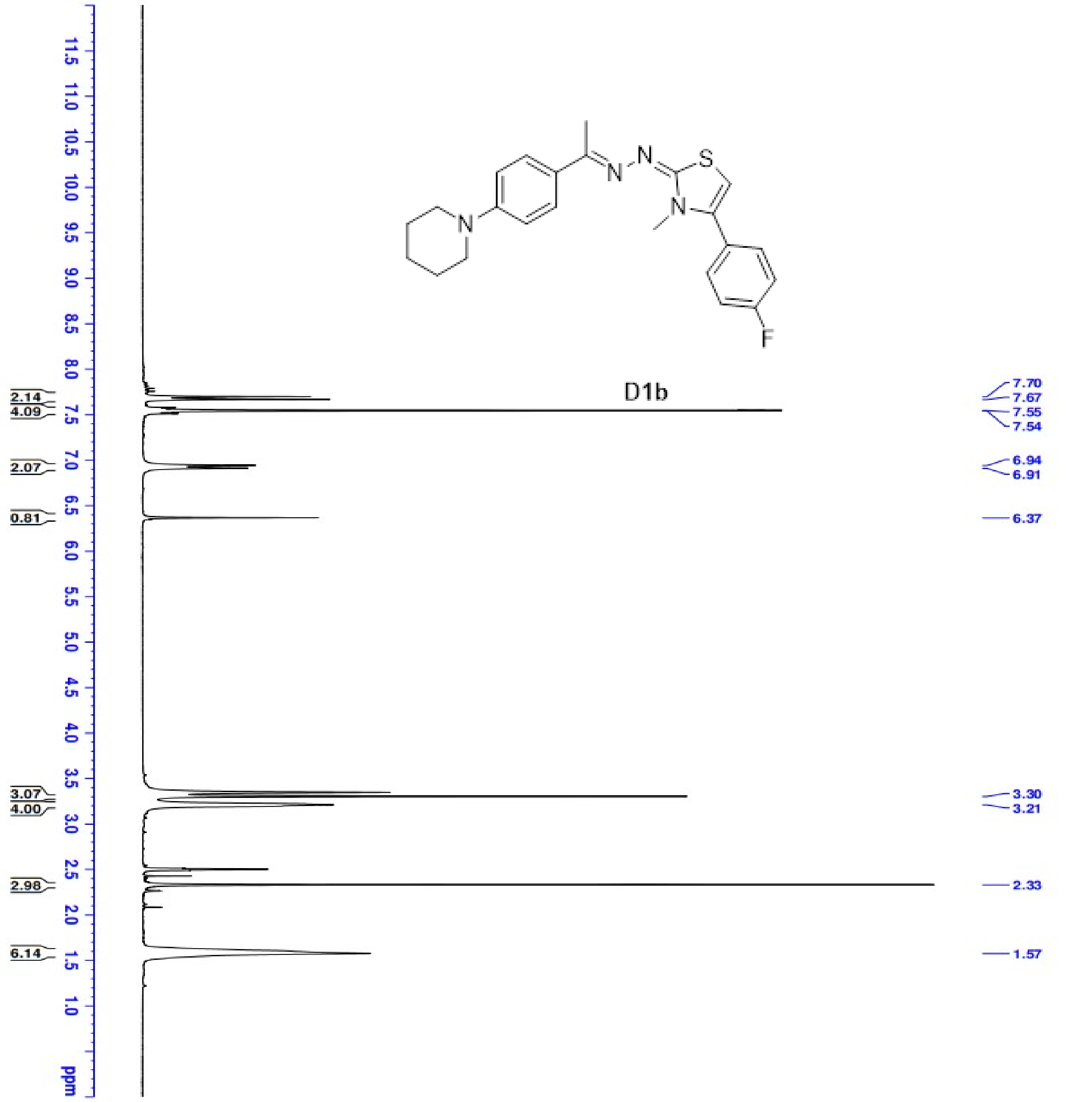

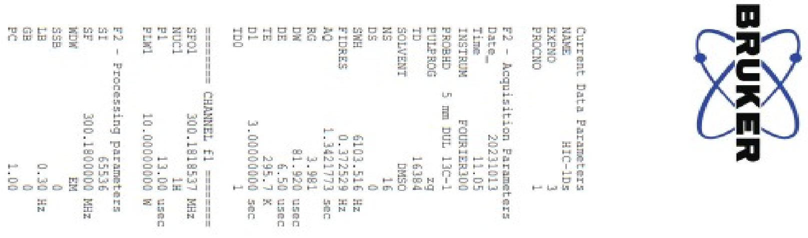

Figure 6S. ^{1}HNMR spectrum of **D1b**.

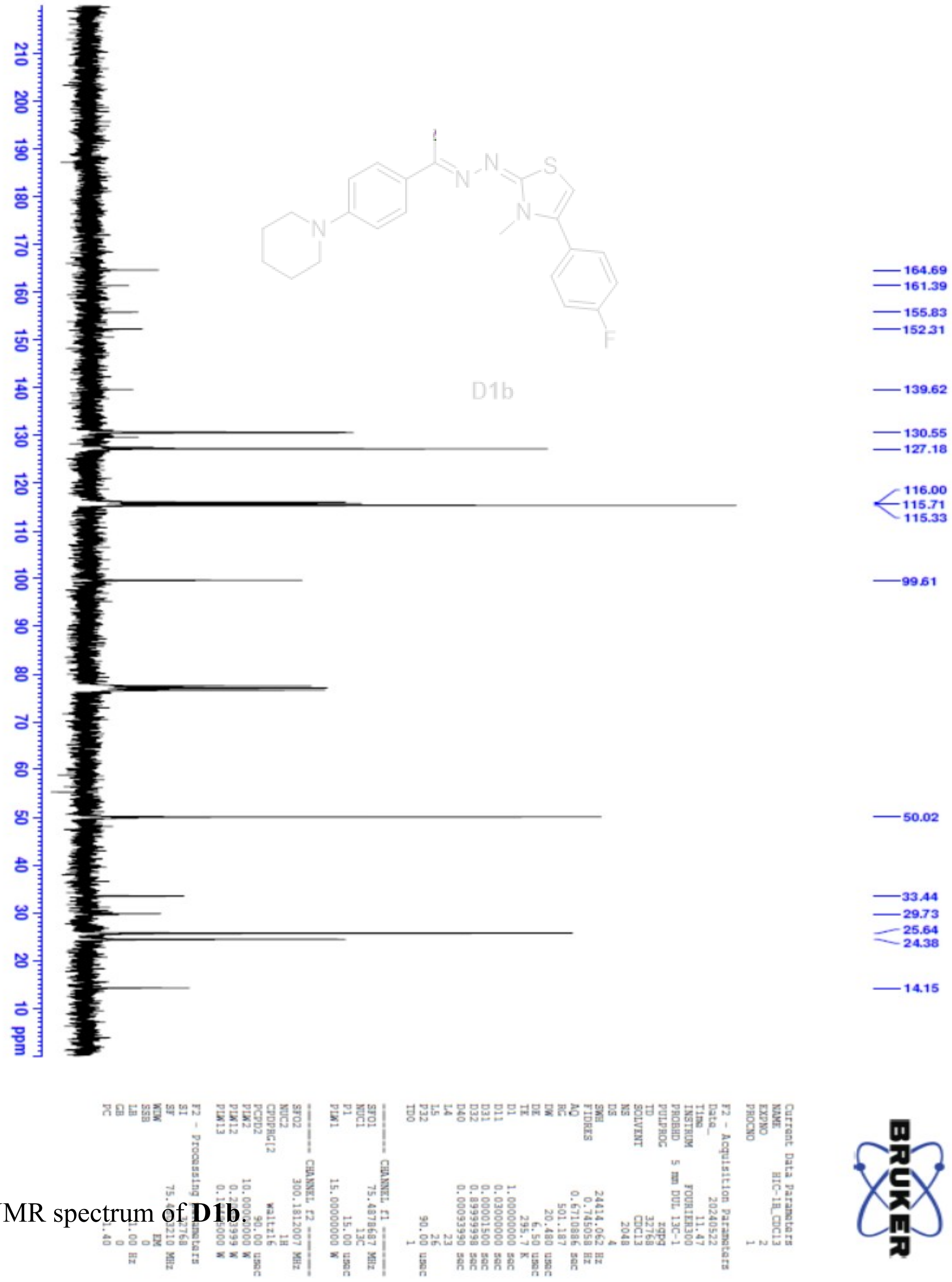

Figure 7S. 13CNMR spectrum of **D1b**.

Data File: C:\LabSolutions\Data\Analiz\derya\HIC-1B UST_578.lcd

Elmt	Val.	Min	Max	Elmt	Val.	Min	Max	Elmt	Val.	Min	Max	Elmt	Val.	Min	Max	Use Adduct
H	1	8	33	O	2	0	3	S	2	0	2	Ru	2	0	0	H
C	4	4	32	F	1	1	1	Cl	1	0	0	Pd	2	0	0	Na
N	3	0	6	P	3	0	0	Br	1	0	0	I	3	0	0	

Error Margin (ppm): 5
HC Ratio: unlimited
Max Isotopes: 3
MSn Iso RI (%): 10.00

DBE Range: 0.0 - 30.0
Apply N Rule: no
Isotope RI (%): 1.00
MSn Logic Mode: AND

Electron Ions: both
Use MSn Info: yes
Isotope Res: 9000
Max Results: 50

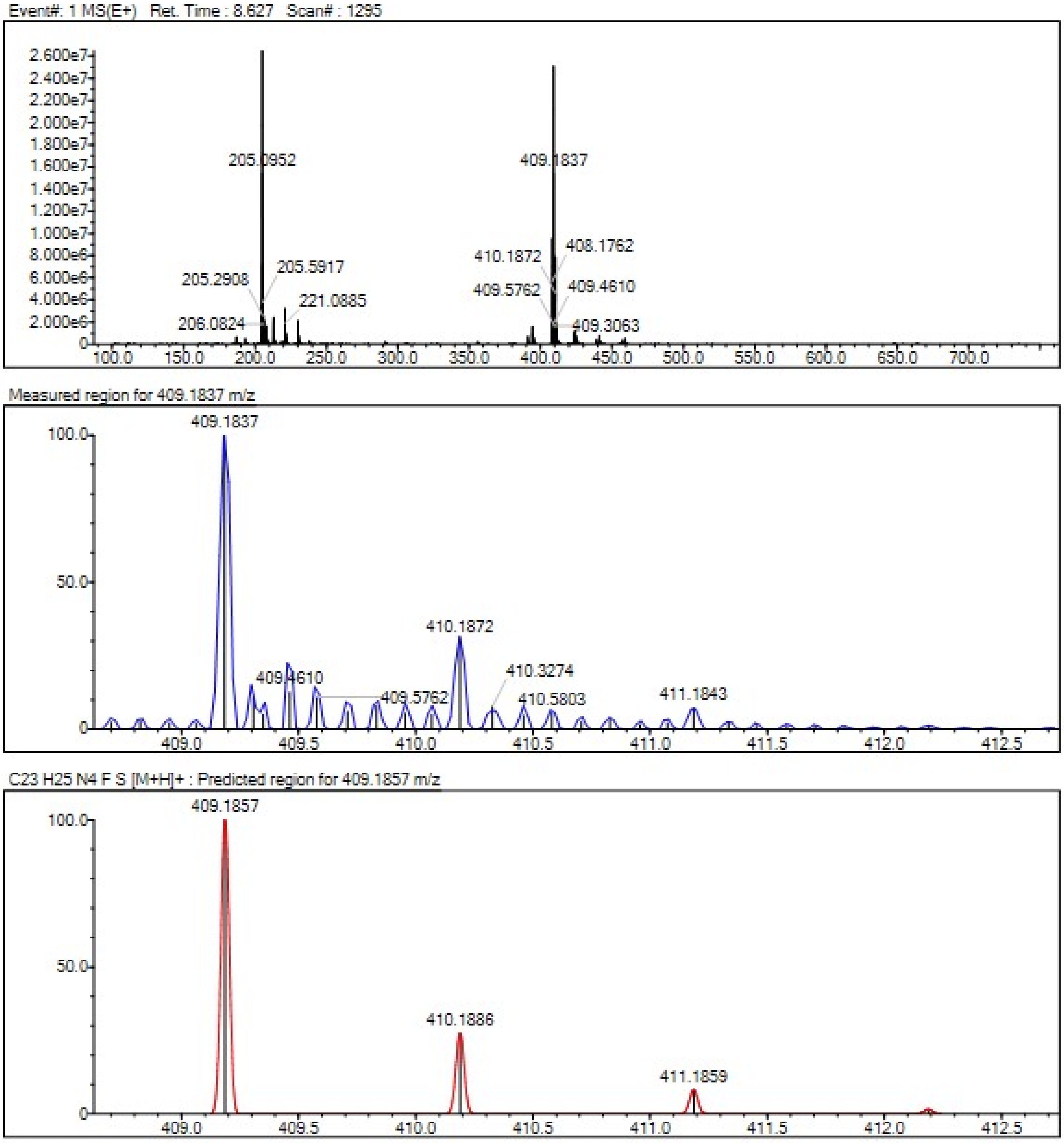

Rank	Score	Formula (M)	Ion	Meas. m/z	Pred. m/z	Df. (mDa)	Df. (ppm)	Iso	DBE
6	85.88	C23 H25 N4 F S	[M+H]+	409.1837	409.1857	-2.0	-4.89	95.13	13.0

Figure 8S. HRMS spectrogram of **D1b**.

Item	Value
Acquired Date&Time	2.05.2024 14:11:58
Acquired by	System Administrator
Filename	C:\Users\dopnalab\Desktop\MASAÜSTÜ\sazan\hic\hic-1c1.ispd
Spectrum name	hic-1c1
Sample name	hic-1c
Sample ID	
Option	
Comment	
No. of Scans	30
Resolution	4 [cm-1]
Apodization	Happ-Genzel

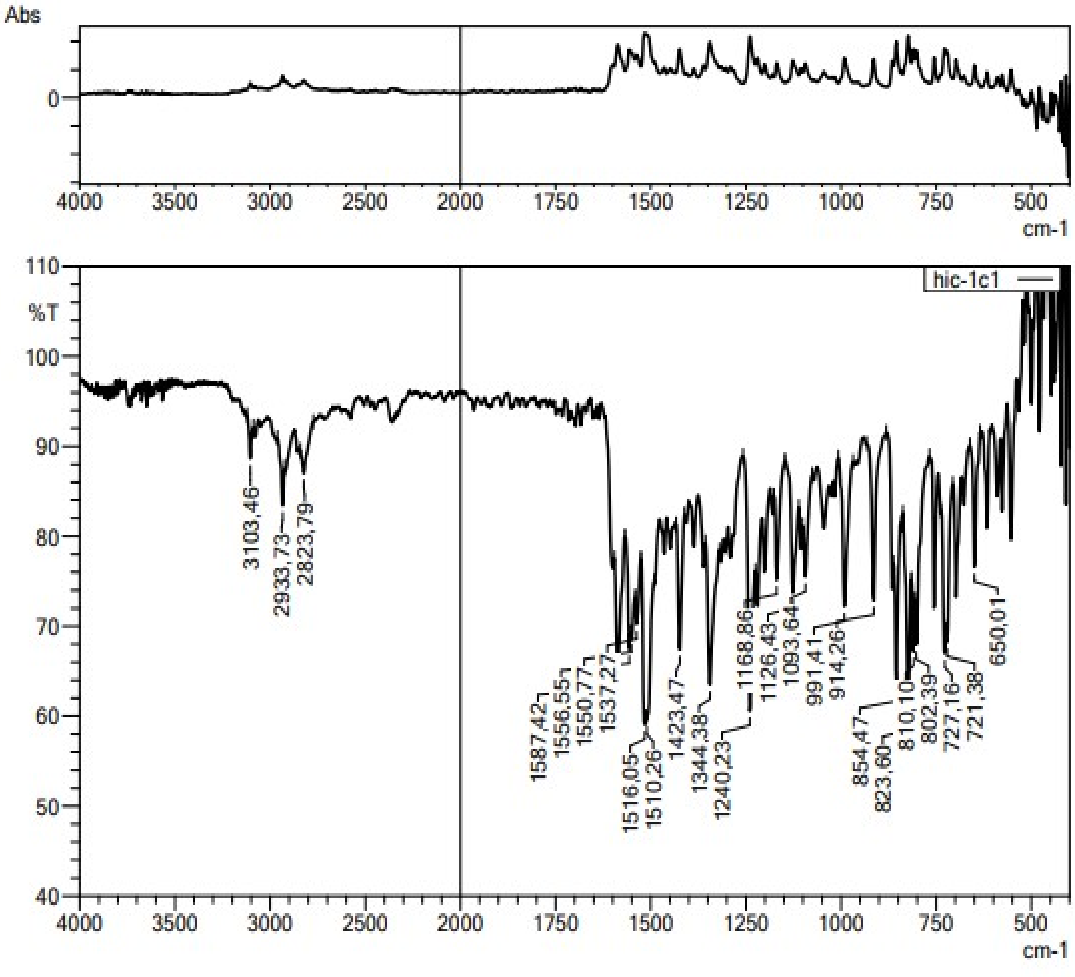

Figure 9S. IR fingerprint of **D1c**.

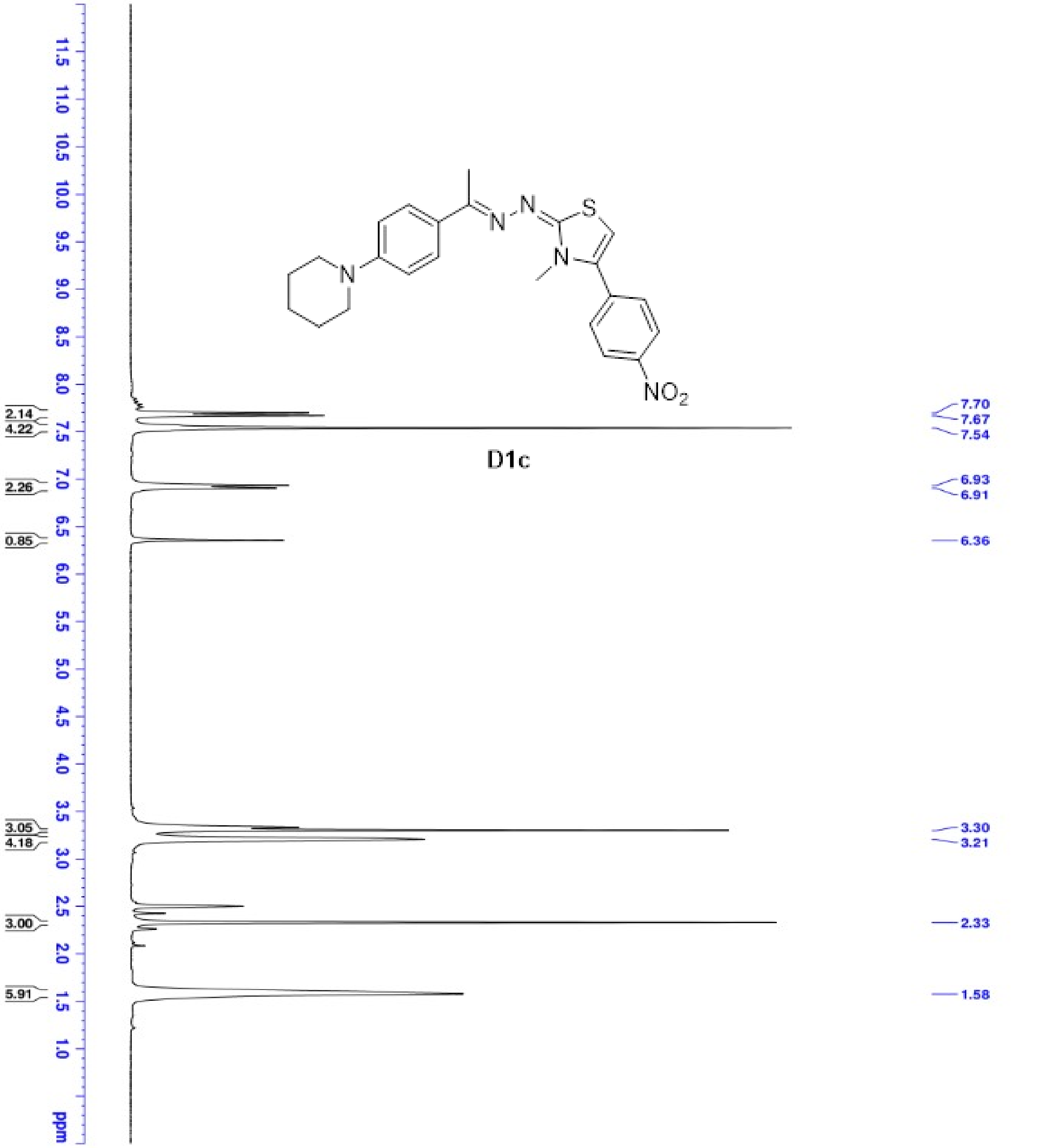

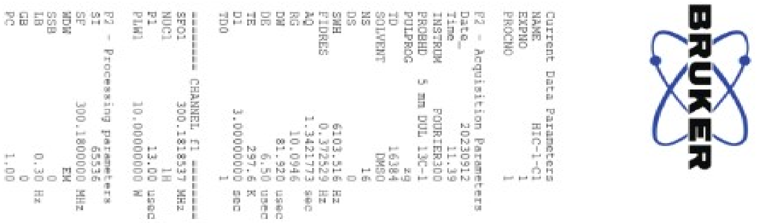

Figure 10S. ^{1}HNMR spectrum of **D1c**..

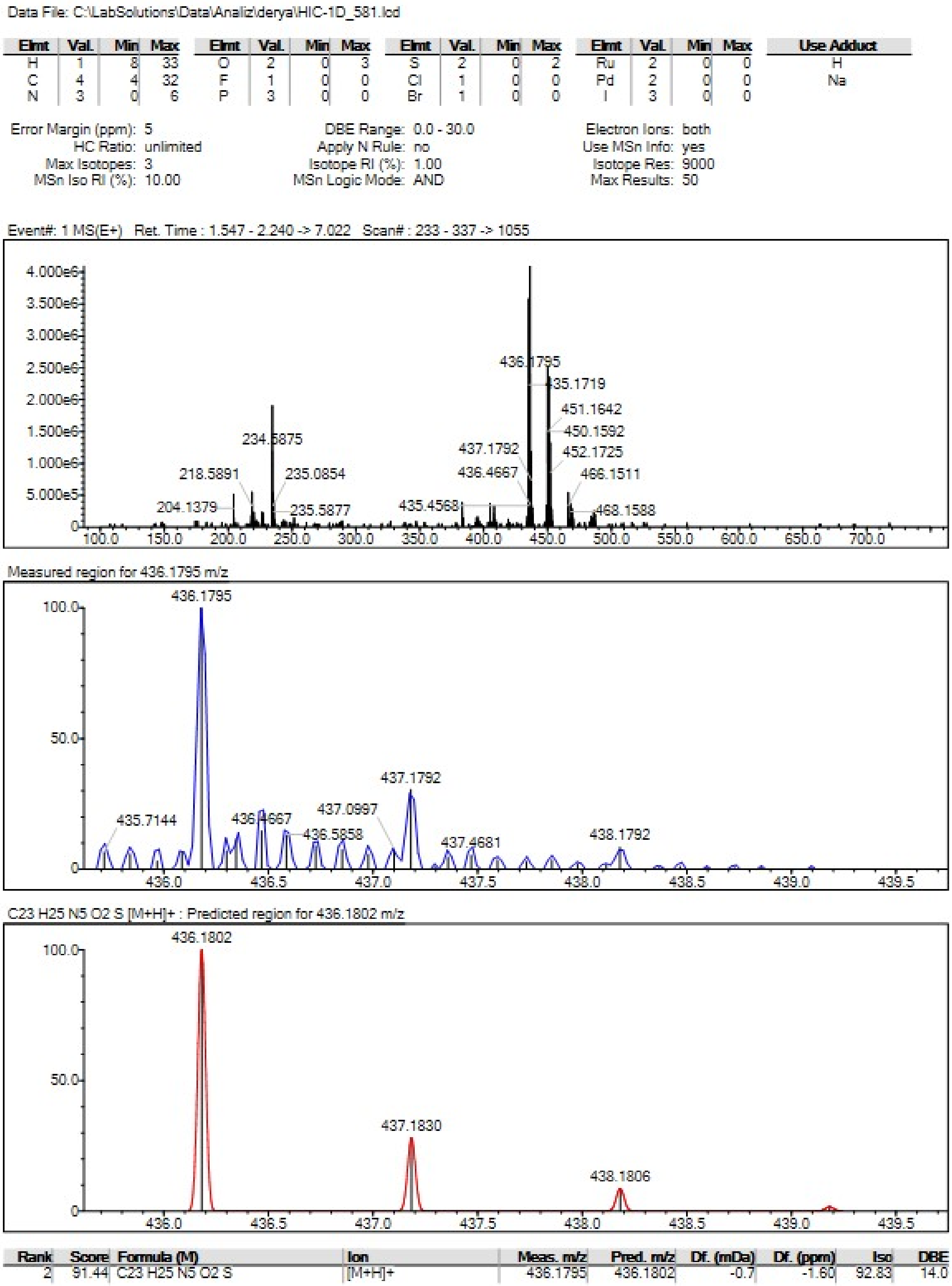

Figure 11S. HRMS spectrogram of **D1c**.

	Item	Value
	Acquired Date&Time	2.05.2024 14:17:43
	Acquired by	System Administrator
	Filename	C:\Users\dopnalab\Desktop\MASAÜSTÜ\sazan\hic\hic-1d1.ispd
	Spectrum name	hic-1d1
	Sample name	hic-1d
	Sample ID	
	Option	
	Comment	
	No. of Scans	30
	Resolution	4 [cm-1]
	Apodization	Happ-Genzel

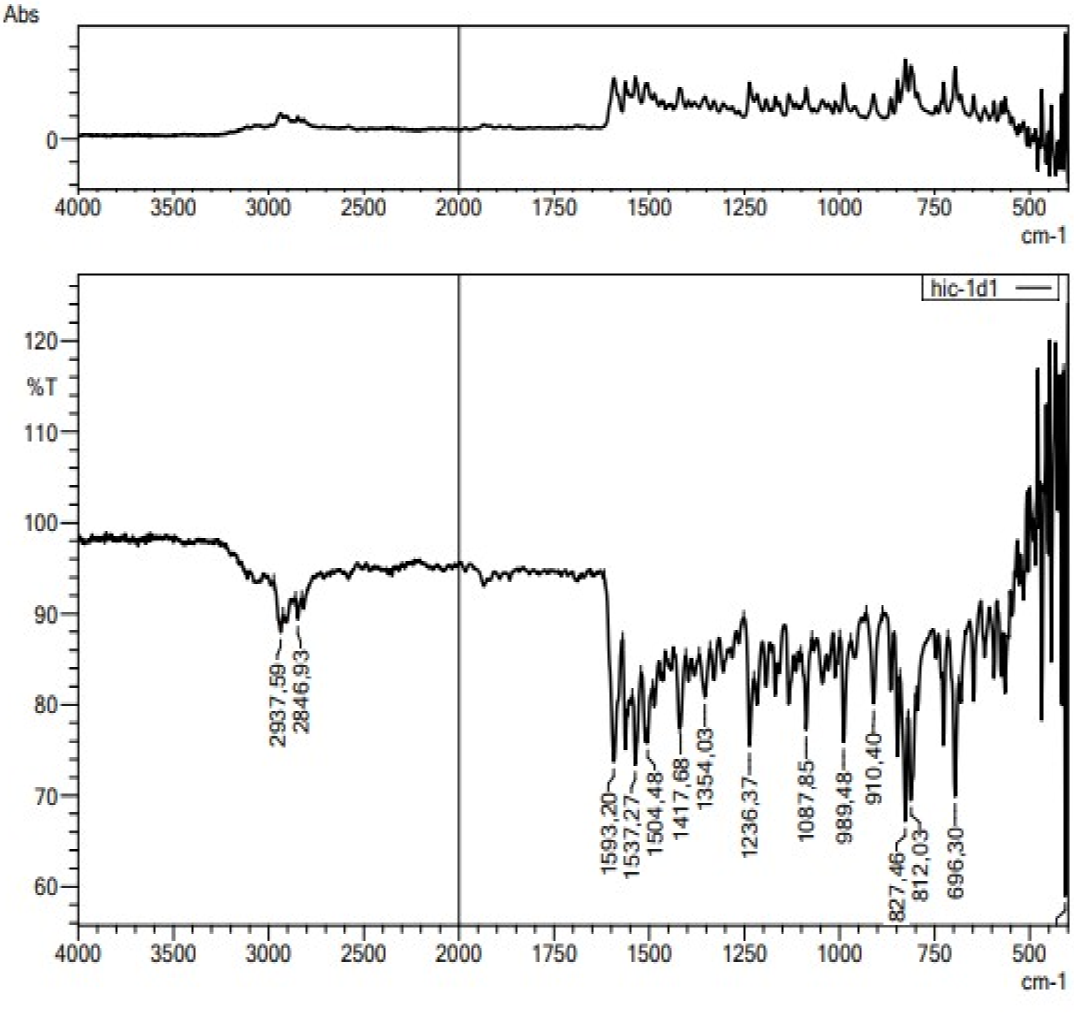

Figure 12S. IR fingerprint of **D1d.**F

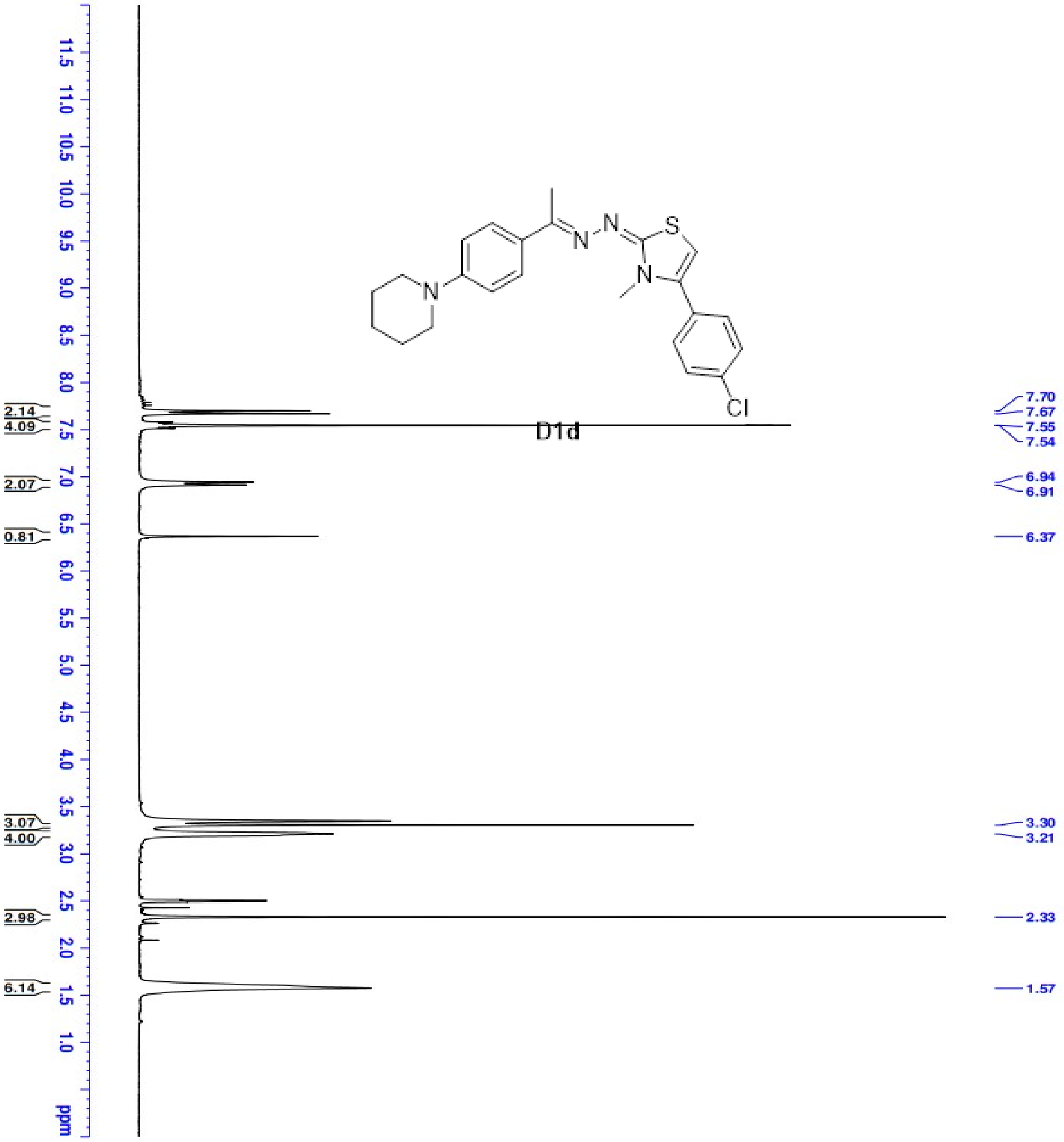

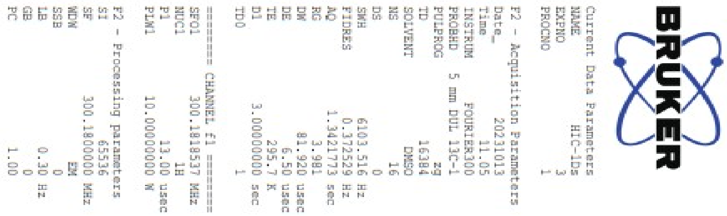

Figure 13S. ¹HNMR spectrum of **D1d**.F

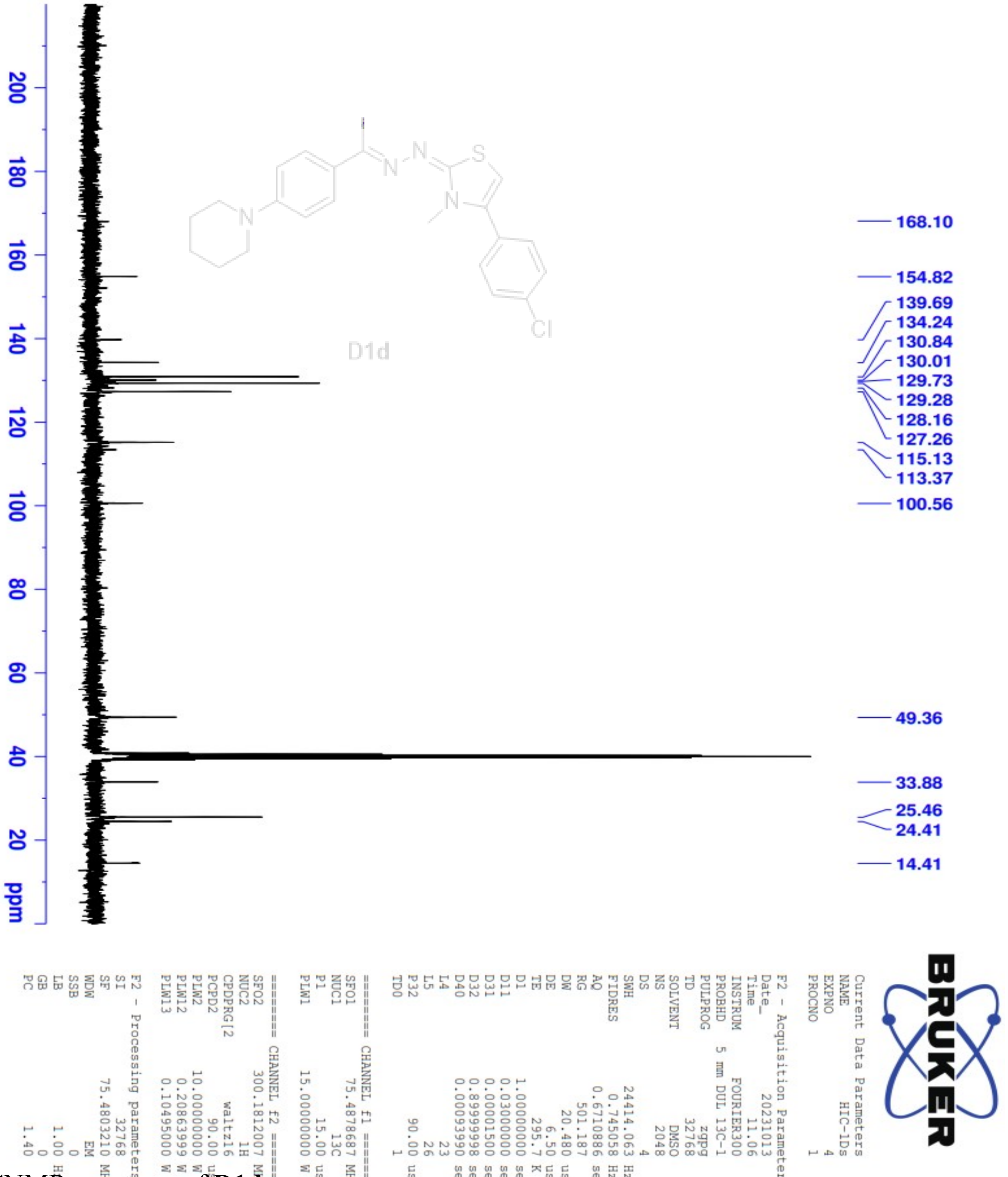

Figure 14S.13CNMR spectrum of **D1d.**

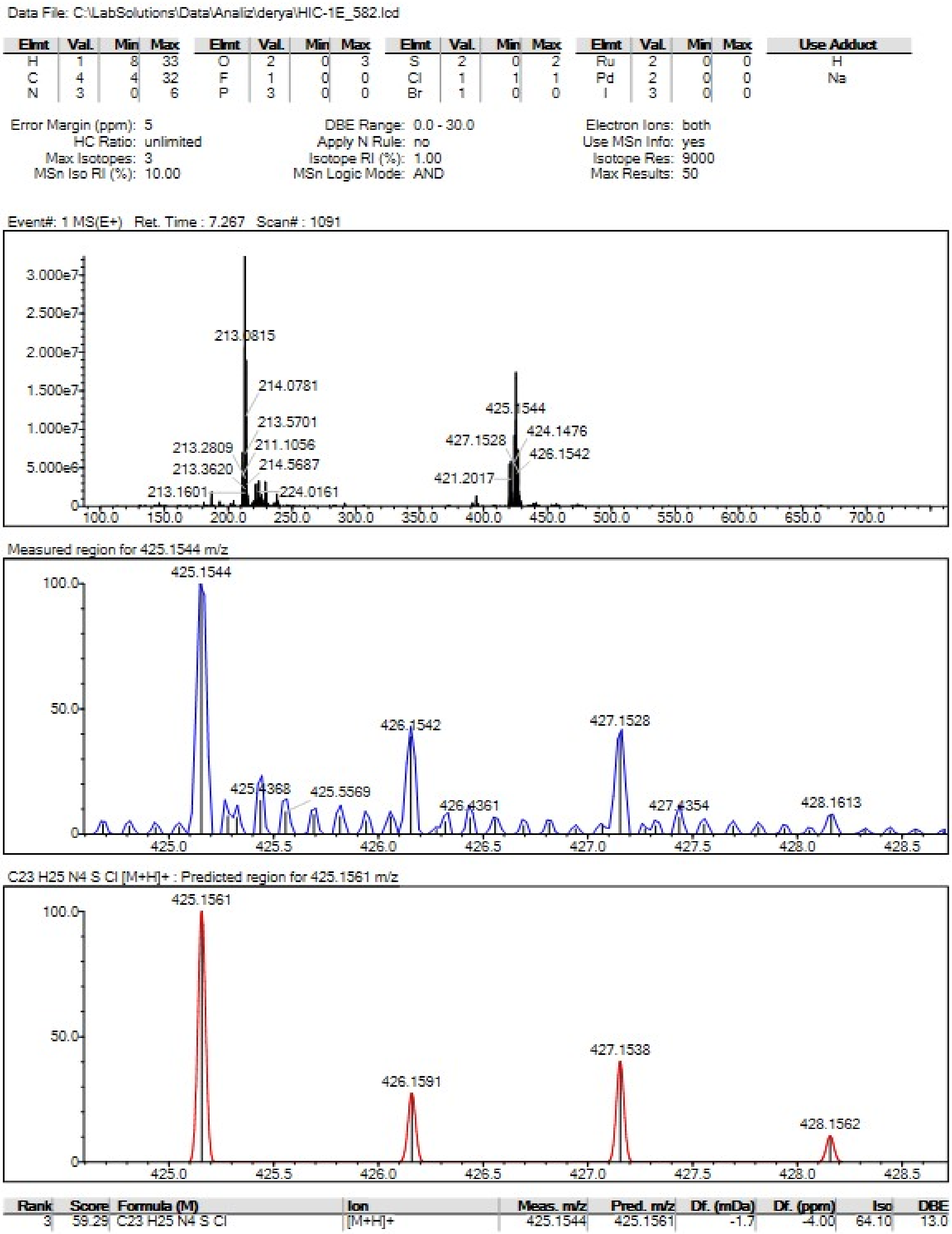

Figure 15S. HRMS spectrogram of **D1d**.

	Item	Value
	Acquired Date&Time	2.05.2024 14:21:51
	Acquired by	System Administrator
	Filename	C:\Users\dopnalab\Desktop\MASAÜSTÜ\sazan\hic\hic-1e1.ispd
	Spectrum name	hic-1e1
	Sample name	hic-1e
	Sample ID	
	Option	
	Comment	
	No. of Scans	30
	Resolution	4 [cm-1]
	Apodization	Happ-Genzel

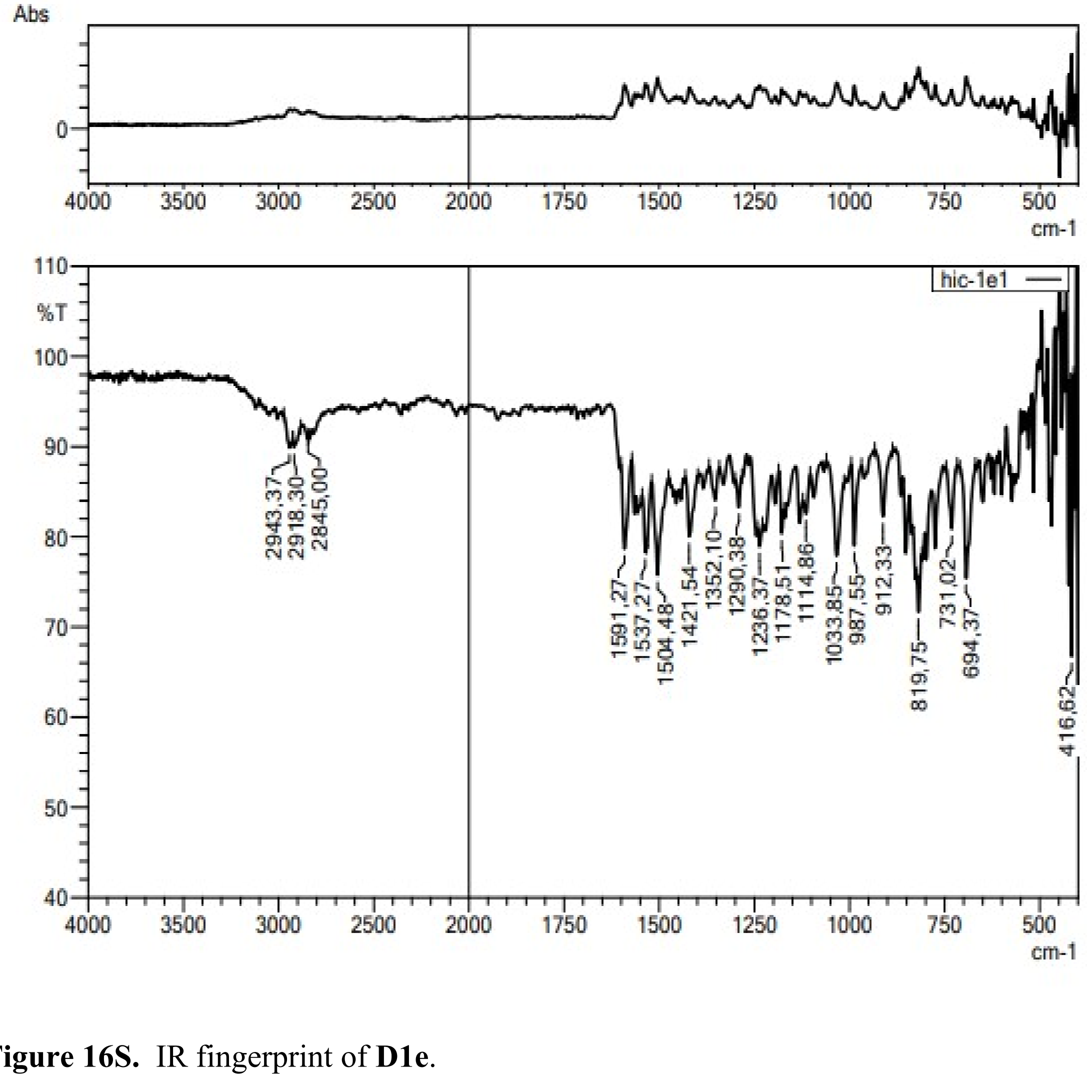

Figure 16S. IR fingerprint of **D1e**.

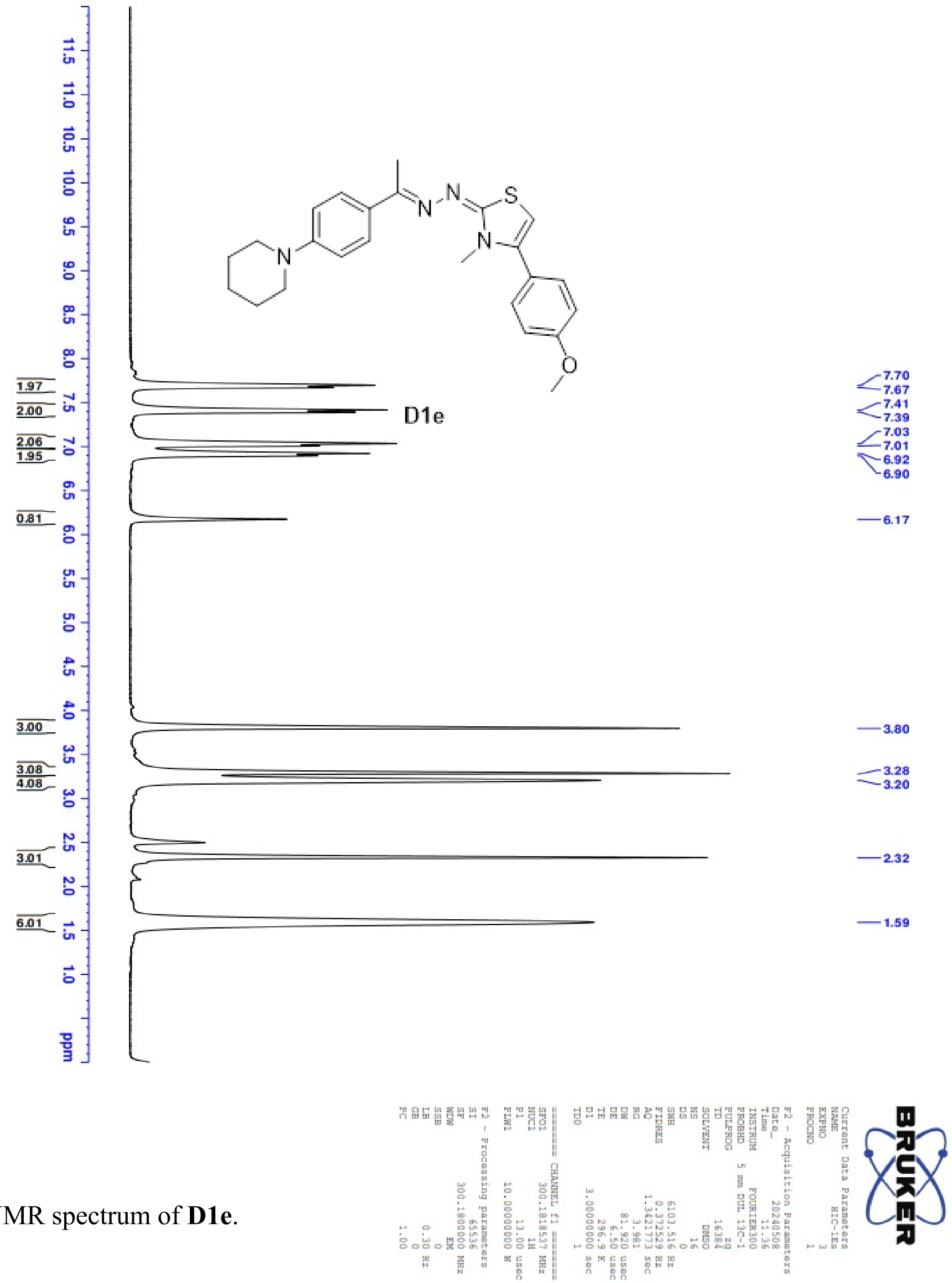

Figure 17S.^{1}HNMR spectrum of **D1e**.

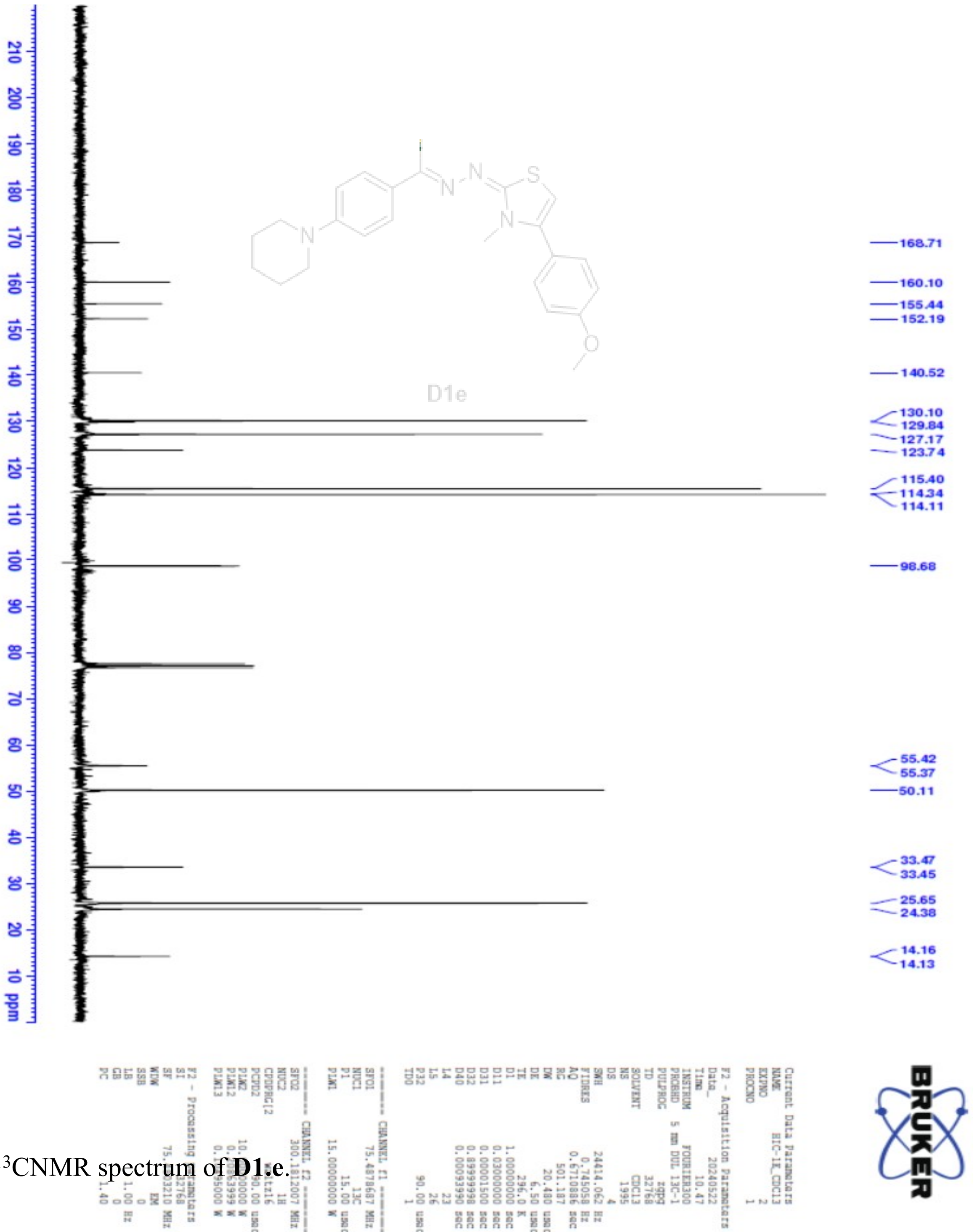

Figure 18S. ^{13}CNMR spectrum of **D1.e**.

Data File: C:\LabSolutions\Data\Analiz\derya\HIC-1F UST_583.lcd

Elmt	Val.	Min	Max	Elmt	Val.	Min	Max	Elmt	Val.	Min	Max	Elmt	Val.	Min	Max	Use Adduct
H	1	8	33	O	2	0	3	S	2	0	2	Ru	2	0	0	H
C	4	4	32	F	1	0	0	Cl	1	0	0	Pd	2	0	0	Na
N	3	0	6	P	3	0	0	Br	1	0	0	I	3	0	0	

Error Margin (ppm): 5
HC Ratio: unlimited
Max Isotopes: 3
MSn Iso RI (%): 10.00

DBE Range: 0.0 - 30.0
Apply N Rule: no
Isotope RI (%): 1.00
MSn Logic Mode: AND

Electron Ions: both
Use MSn Info: yes
Isotope Res: 9000
Max Results: 50

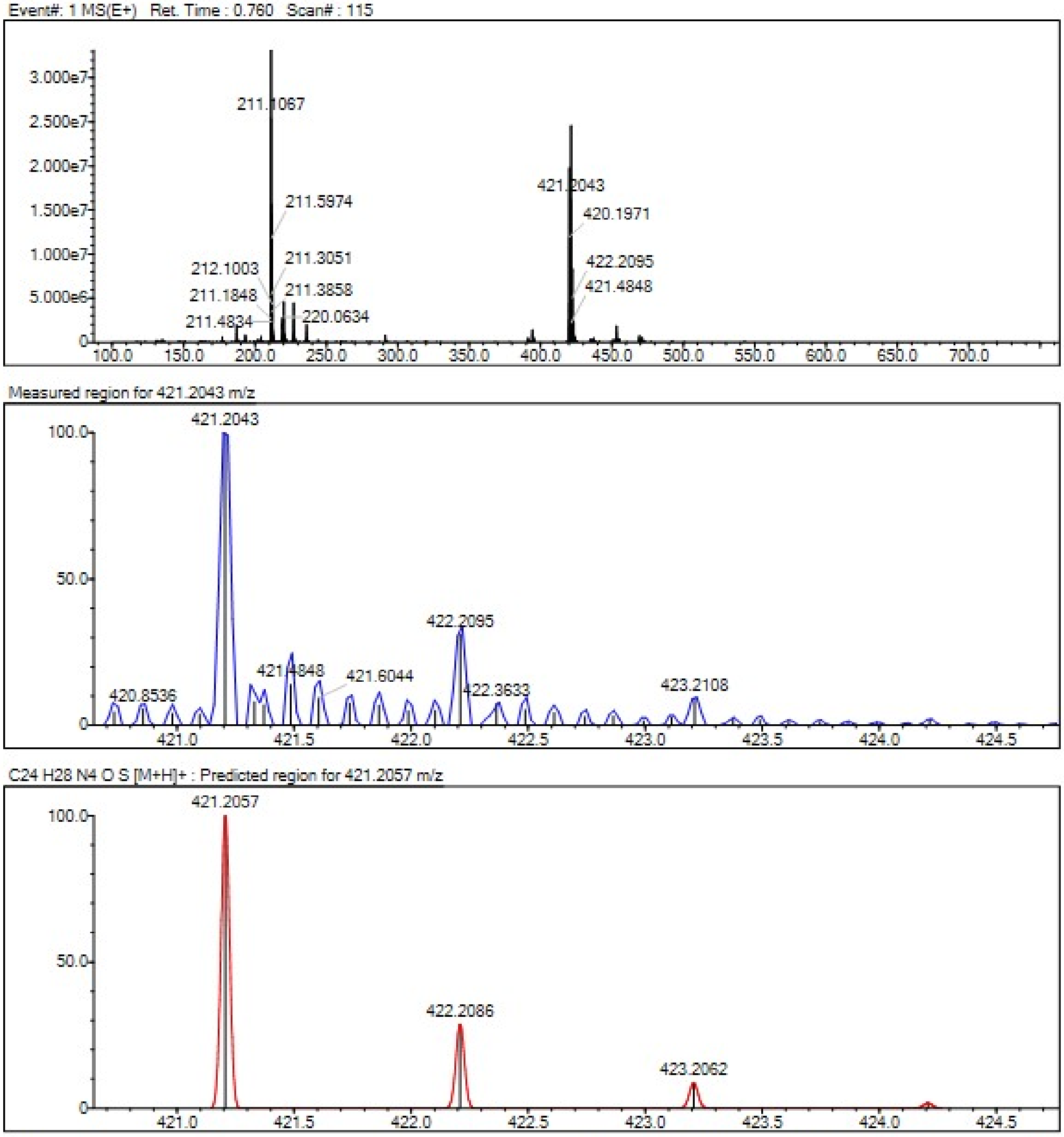

Rank	Score	Formula (M)	Ion	Meas. m/z	Pred. m/z	Df. (mDa)	Df. (ppm)	Iso	DBE
2	82.91	C24 H28 N4 O S	[M+H]+	421.2043	421.2057	-1.4	-3.32	88.02	13.0

Figure 19S. HRMS spectrogram of **D1e**.

	Item	Value
	Acquired Date&Time	2.05.2024 14:26:20
	Acquired by	System Administrator
	Filename	C:\Users\dopnalab\Desktop\MASAÜSTÜ\sazan\hic\hic-1f1.ispd
	Spectrum name	hic-1f1
	Sample name	hic-1f
	Sample ID	
	Option	
	Comment	
	No. of Scans	30
	Resolution	4 [cm-1]
	Apodization	Happ-Genzel

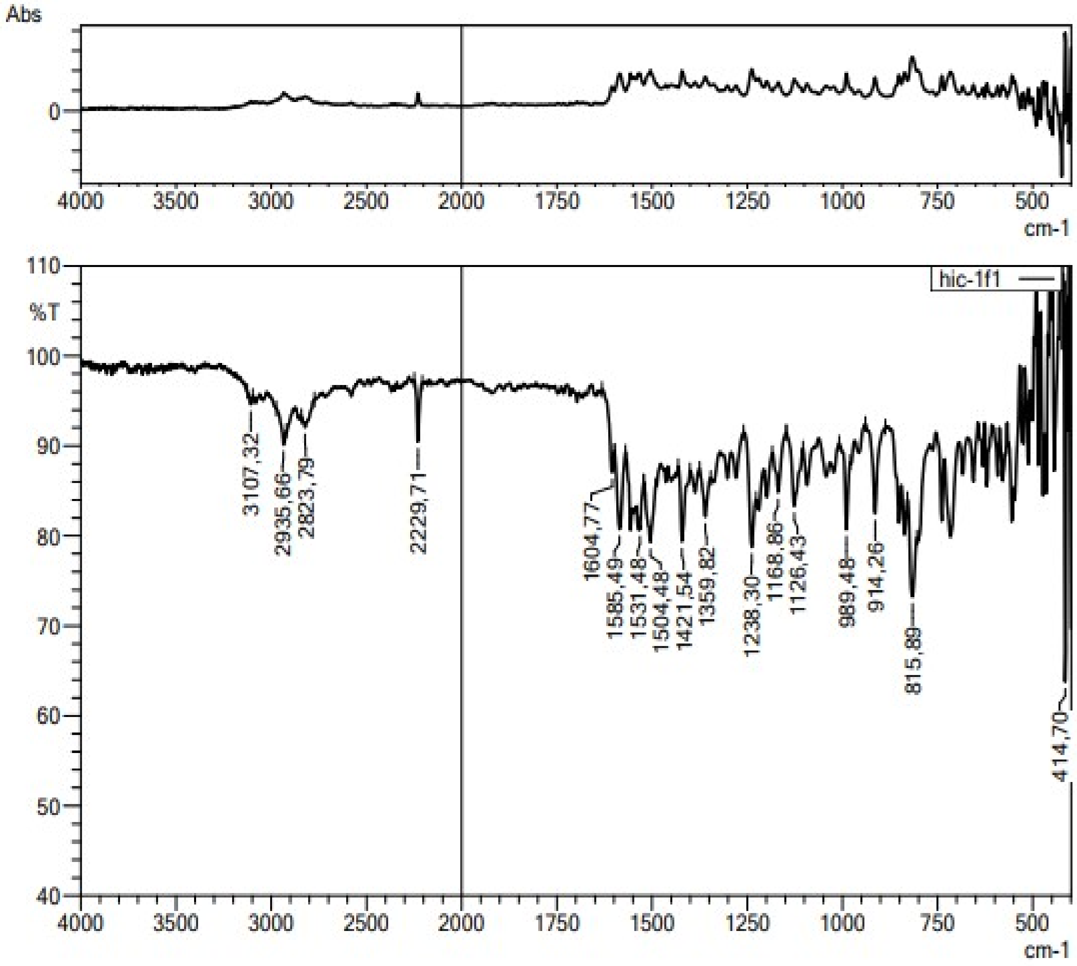

Figure 20S. IR fingerprint of **D1f**.F

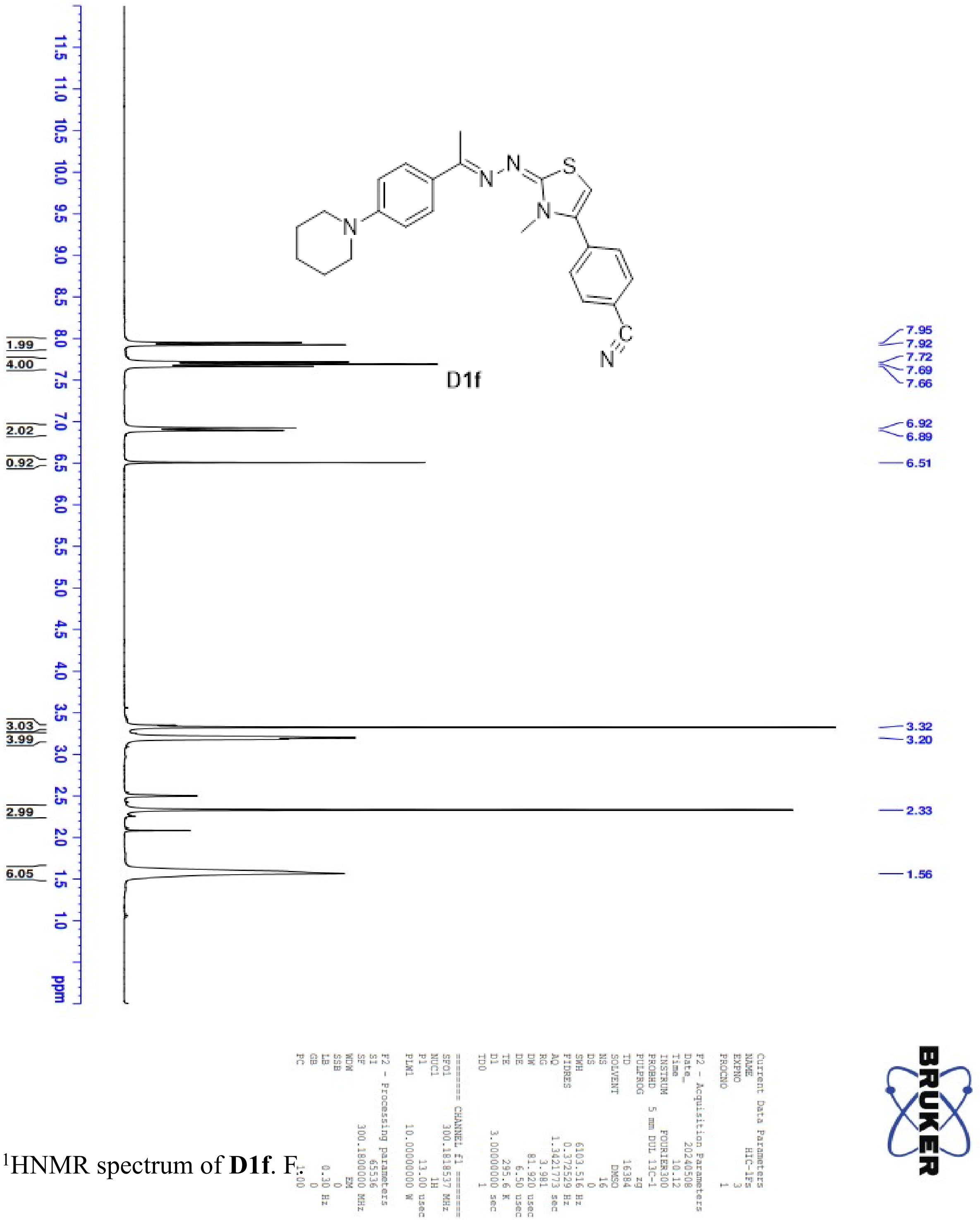

Figure 21S. ^{1}HNMR spectrum of **D1f**. F.

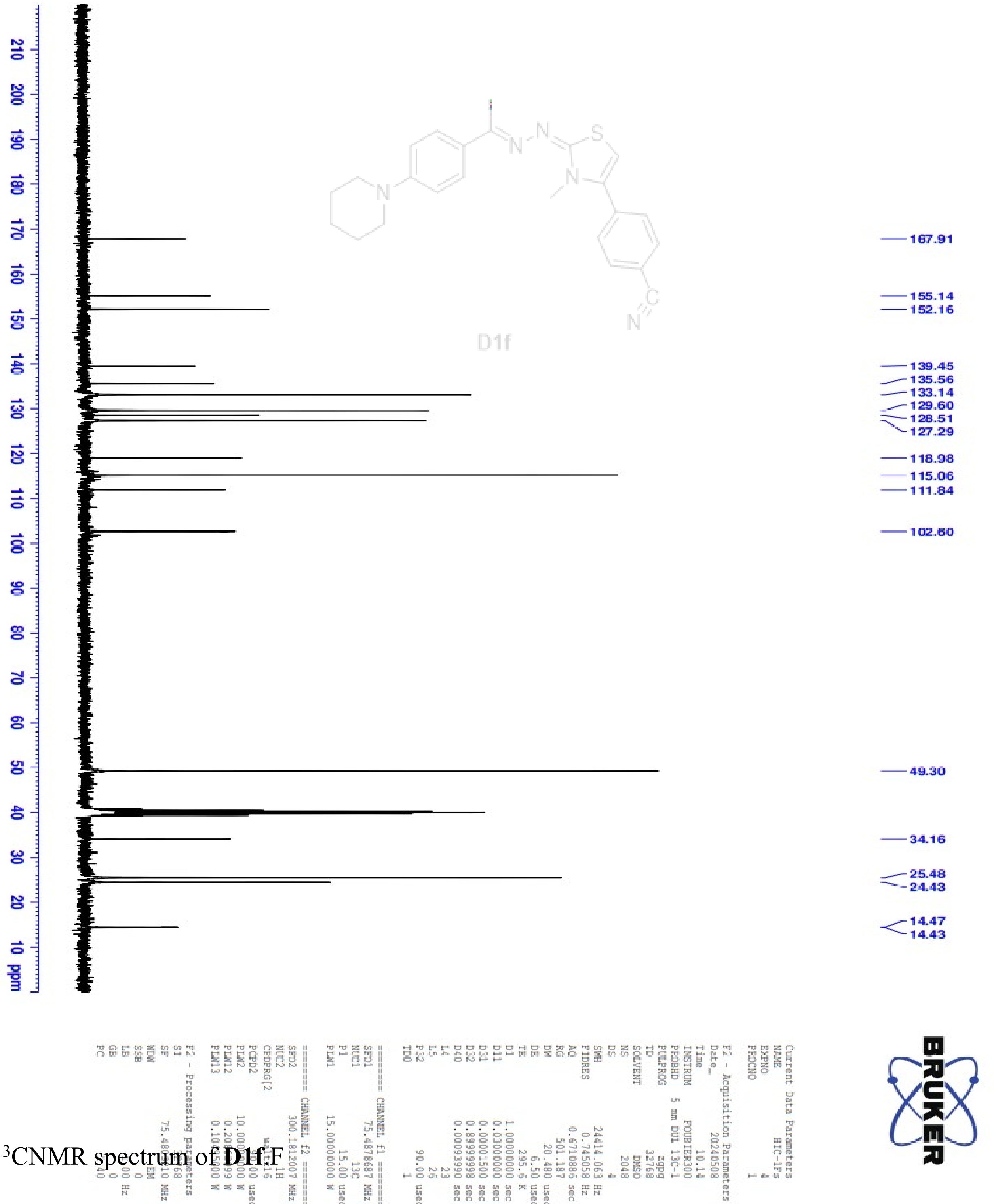

Figure 22S. ^{13}CNMR spectrum of **D1f**.F

Data File: C:\LabSolutions\Data\Analiz\derya\HIC-1F ALT_584.lcd

Elmt	Val.	Min	Max	Elmt	Val.	Min	Max	Elmt	Val.	Min	Max	Elmt	Val.	Min	Max	Use Adduct
H	1	8	33	O	2	0	3	S	2	0	2	Ru	2	0	0	H
C	4	4	32	F	1	0	0	Cl	1	0	0	Pd	2	0	0	Na
N	3	0	6	P	3	0	0	Br	1	0	0	I	3	0	0	

Error Margin (ppm): 5
HC Ratio: unlimited
Max Isotopes: 3
MSn Iso RI (%): 10.00

DBE Range: 0.0 - 30.0
Apply N Rule: no
Isotope RI (%): 1.00
MSn Logic Mode: AND

Electron Ions: both
Use MSn Info: yes
Isotope Res: 9000
Max Results: 50

Event#: 1 MS(E+) Ret. Time : 7.960 Scan# : 1195

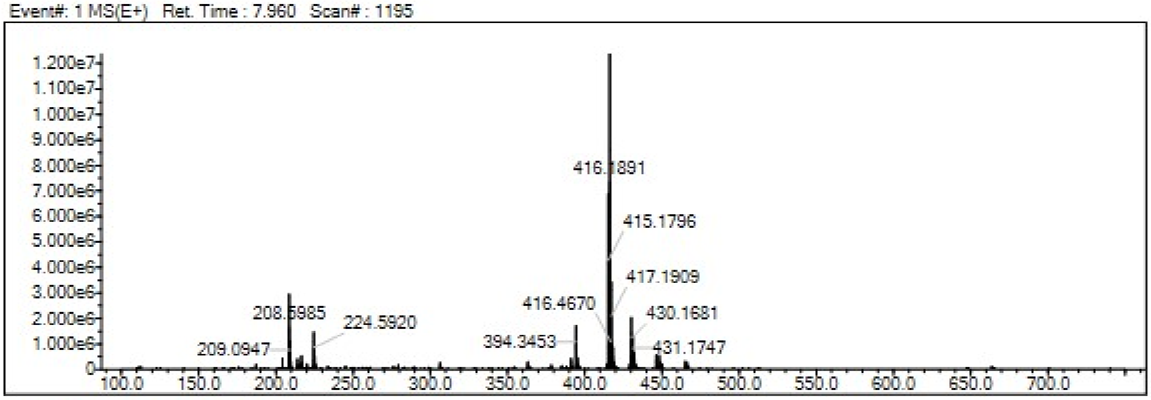

Measured region for 416.1891 m/z

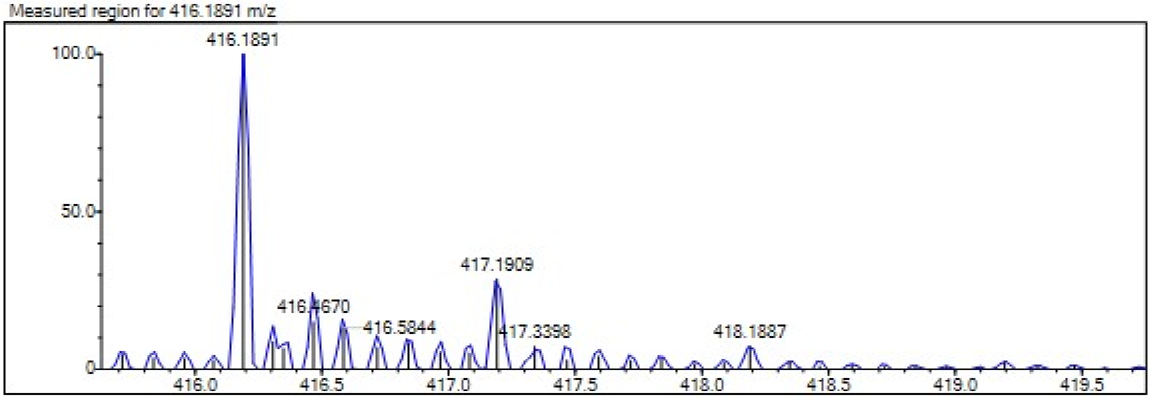

C24 H25 N5 S [M+H]+ : Predicted region for 416.1903 m/z

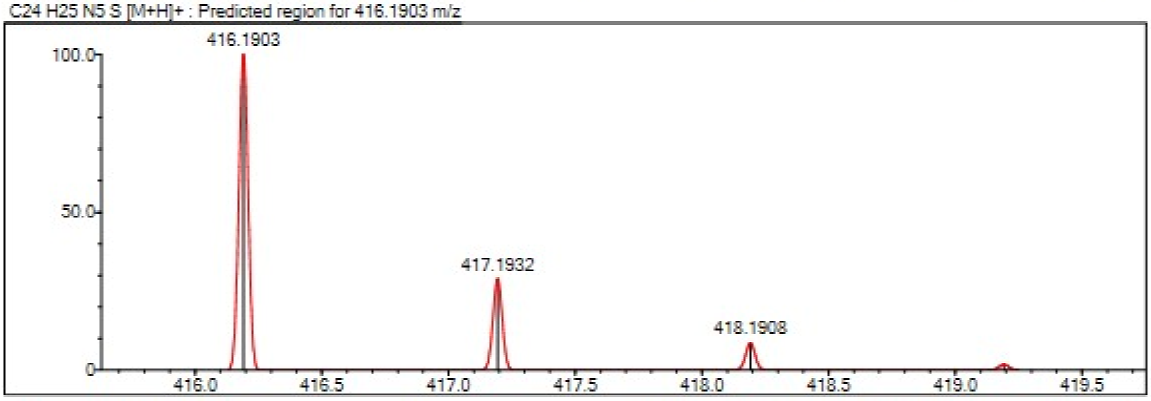

Rank	Score	Formula (M)	Ion	Meas. m/z	Pred. m/z	Df. (mDa)	Df. (ppm)	Iso	DBE
2	94.20	C24 H25 N5 S	[M+H]+	416.1891	416.1903	-1.2	-2.88	98.84	15.0

Figure 23S. HRMS spectrogram of **D1f**.

Item	Value
Acquired Date&Time	2.05.2024 14:32:01
Acquired by	System Administrator
Filename	C:\Users\dopnalab\Desktop\MASAÜSTÜ\sazan\hic\hic-1h1.ispd
Spectrum name	hic-1h1
Sample name	hic-1h
Sample ID	
Option	
Comment	
No. of Scans	30
Resolution	4 [cm-1]
Apodization	Happ-Genzel

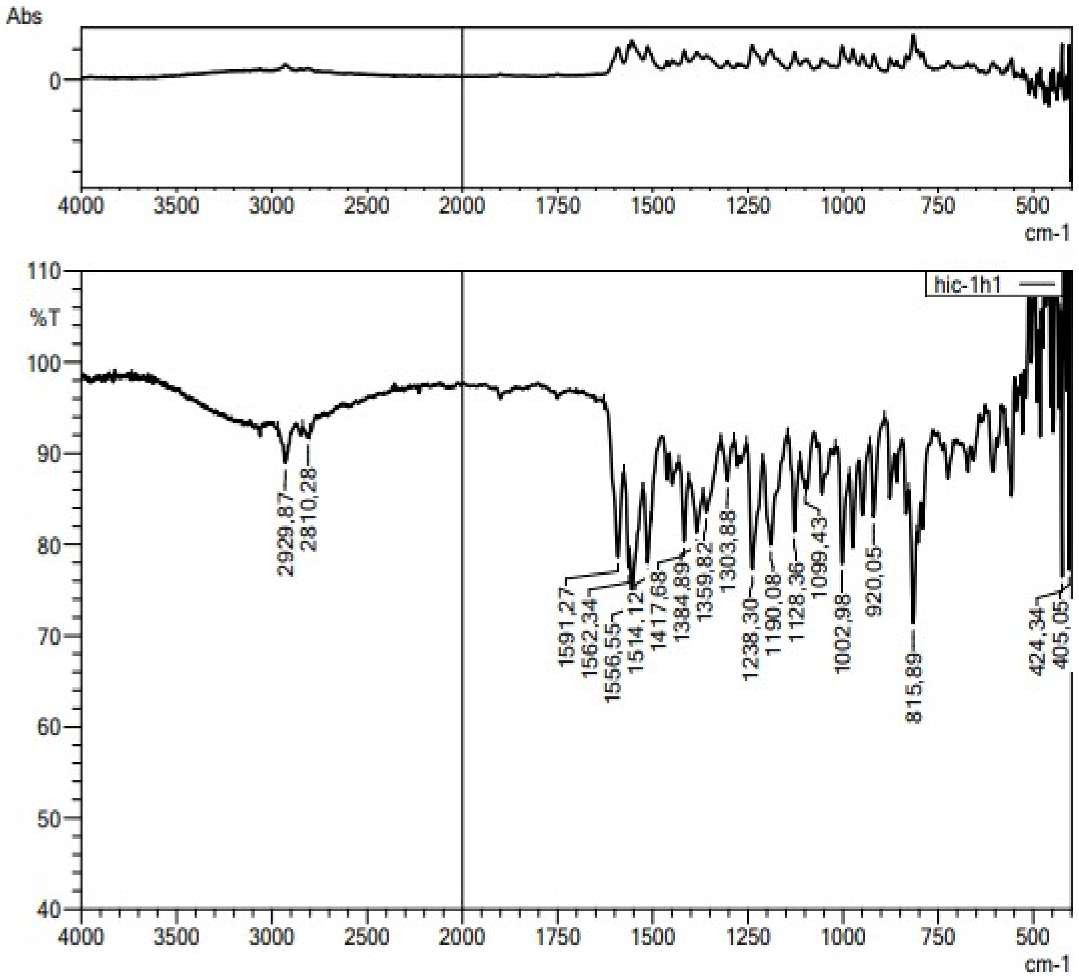

Figure 24S. IR fingerprint of **D1.g**.F

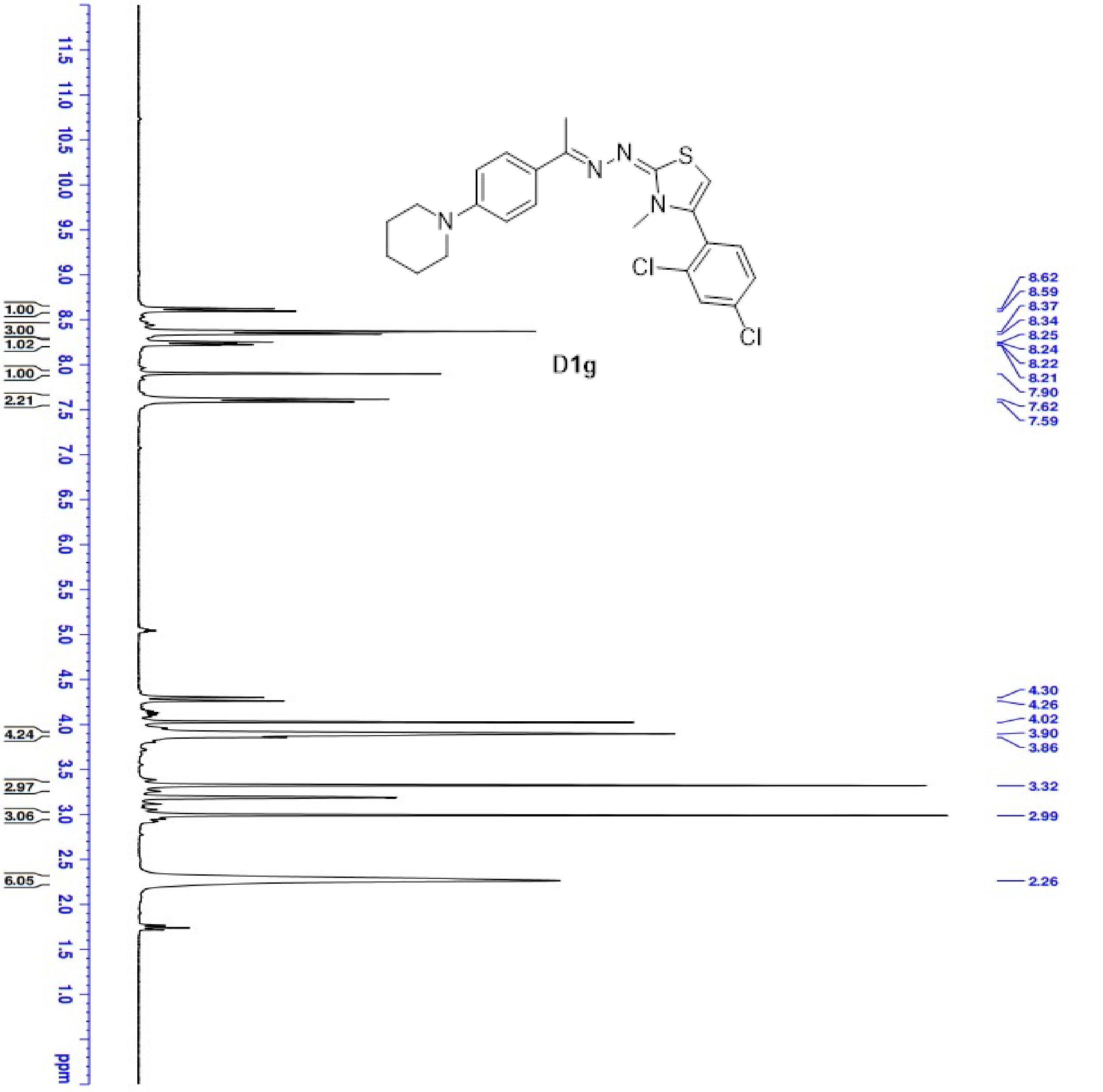

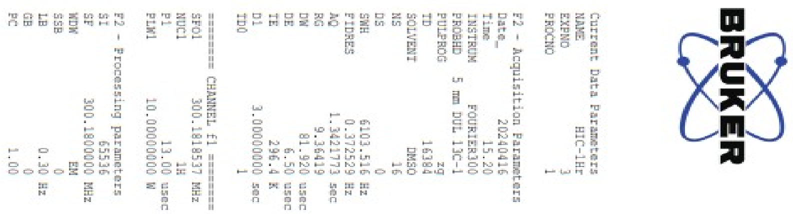

Figure 25S.^{1}HNMR spectrum of **D1g**.F

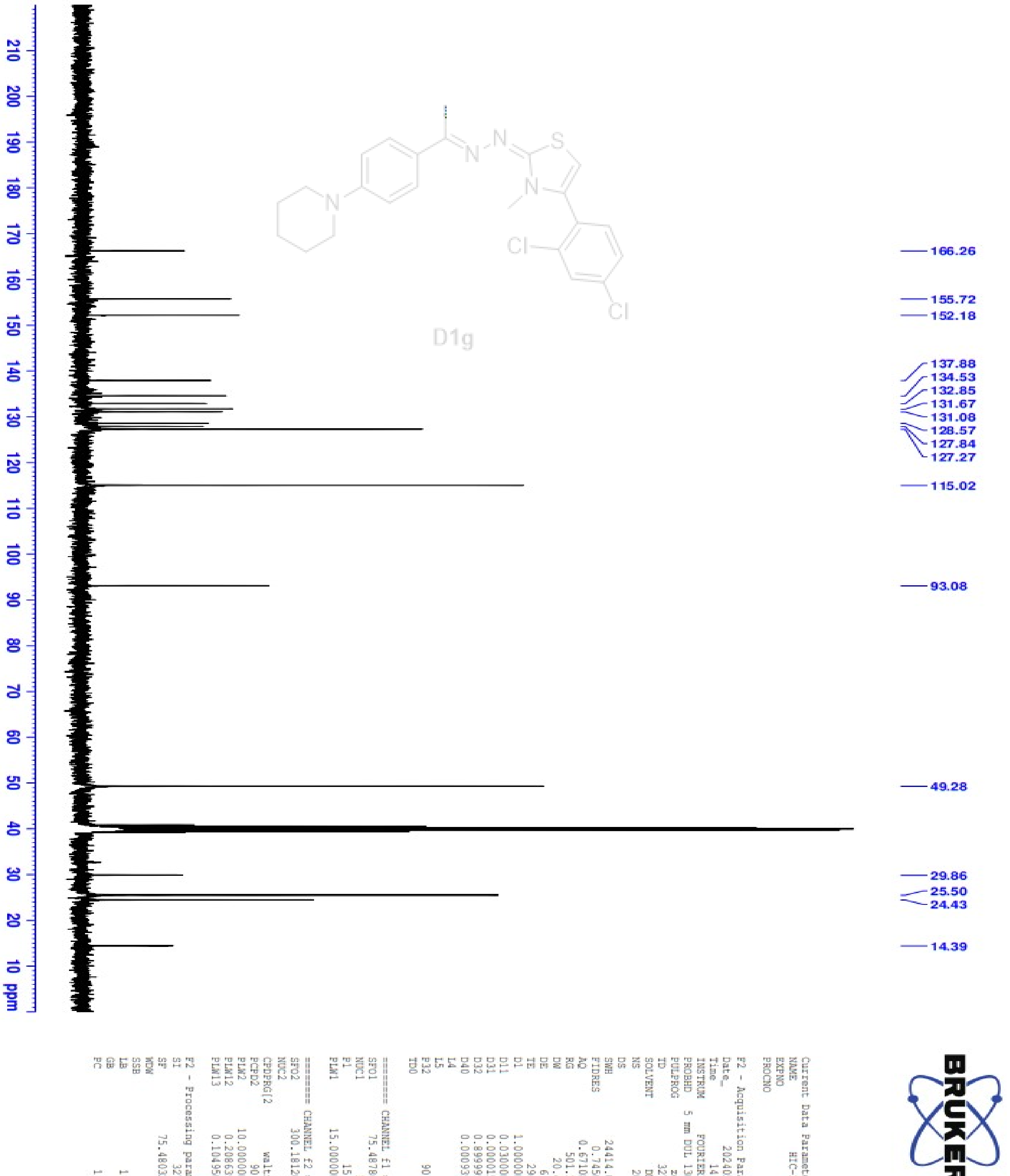

Figure 26S. ^{13}CNMR spectrum of **1Dg**. F

Data File: C:\LabSolutions\Data\Analiz\derya\HIC-1I ALT_587.lcd

Elmt	Val.	Min	Max	Elmt	Val.	Min	Max	Elmt	Val.	Min	Max	Elmt	Val.	Min	Max	Use Adduct
H	1	8	33	O	2	0	3	S	2	0	2	Ru	2	0	0	H
C	4	4	32	F	1	0	0	Cl	1	2	2	Pd	2	0	0	Na
N	3	0	6	P	3	0	0	Br	1	0	0	I	3	0	0	

Error Margin (ppm):	5	DBE Range:	0.0 - 30.0	Electron Ions:	both
HC Ratio:	unlimited	Apply N Rule:	no	Use MSn Info:	yes
Max Isotopes:	3	Isotope RI (%):	1.00	Isotope Res:	9000
MSn Iso RI (%):	10.00	MSn Logic Mode:	AND	Max Results:	50

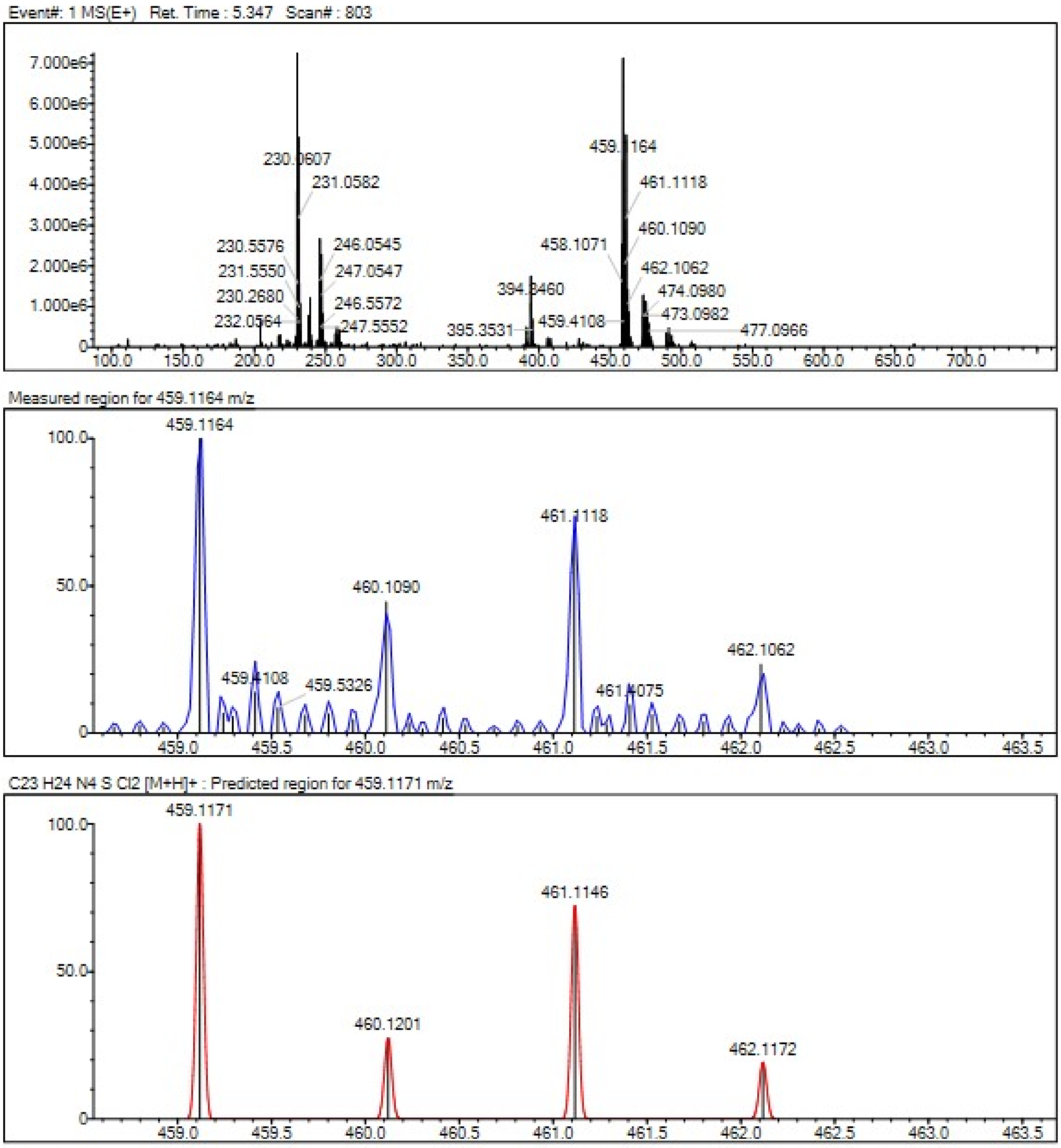

Rank	Score	Formula (M)	Ion	Meas. m/z	Pred. m/z	Df. (mDa)	Df. (ppm)	Iso	DBE
2	54.47	C23 H24 N4 S Cl2	[M+H]+	459.1164	459.1171	-0.7	-1.52	55.19	13.0

Figure 27S. HRMS spectrogram of **D1g**.

Item	Value
Acquired Date&Time	2.05.2024 14:43:58
Acquired by	System Administrator
Filename	C:\Users\dopnalab\Desktop\MASAÜSTÜ\sazan\hic\hic-1i2.ispd
Spectrum name	hic-1i2
Sample name	hic-1i
Sample ID	
Option	
Comment	
No. of Scans	30
Resolution	4 [cm-1]
Apodization	Happ-Genzel

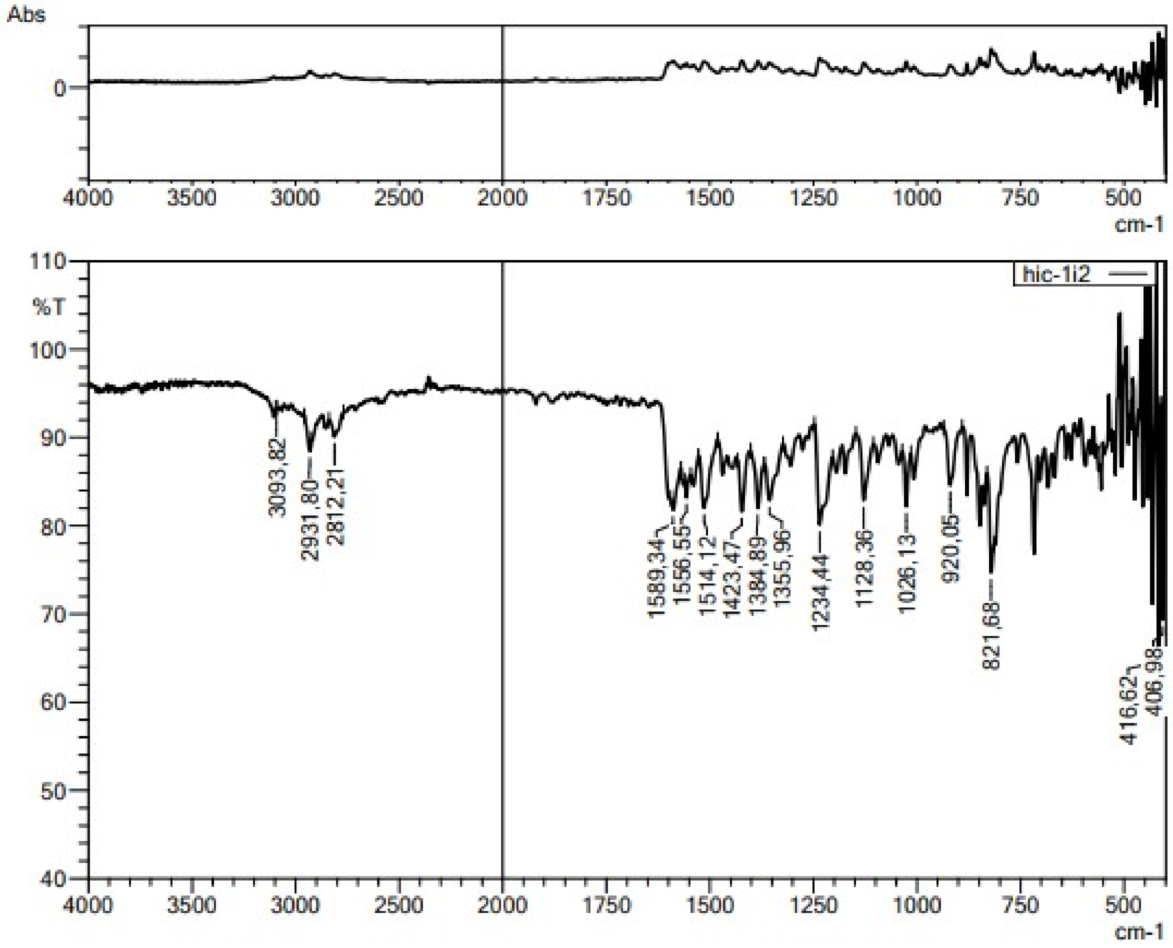

Figure 28S. IR fingerprint of **D1h**.

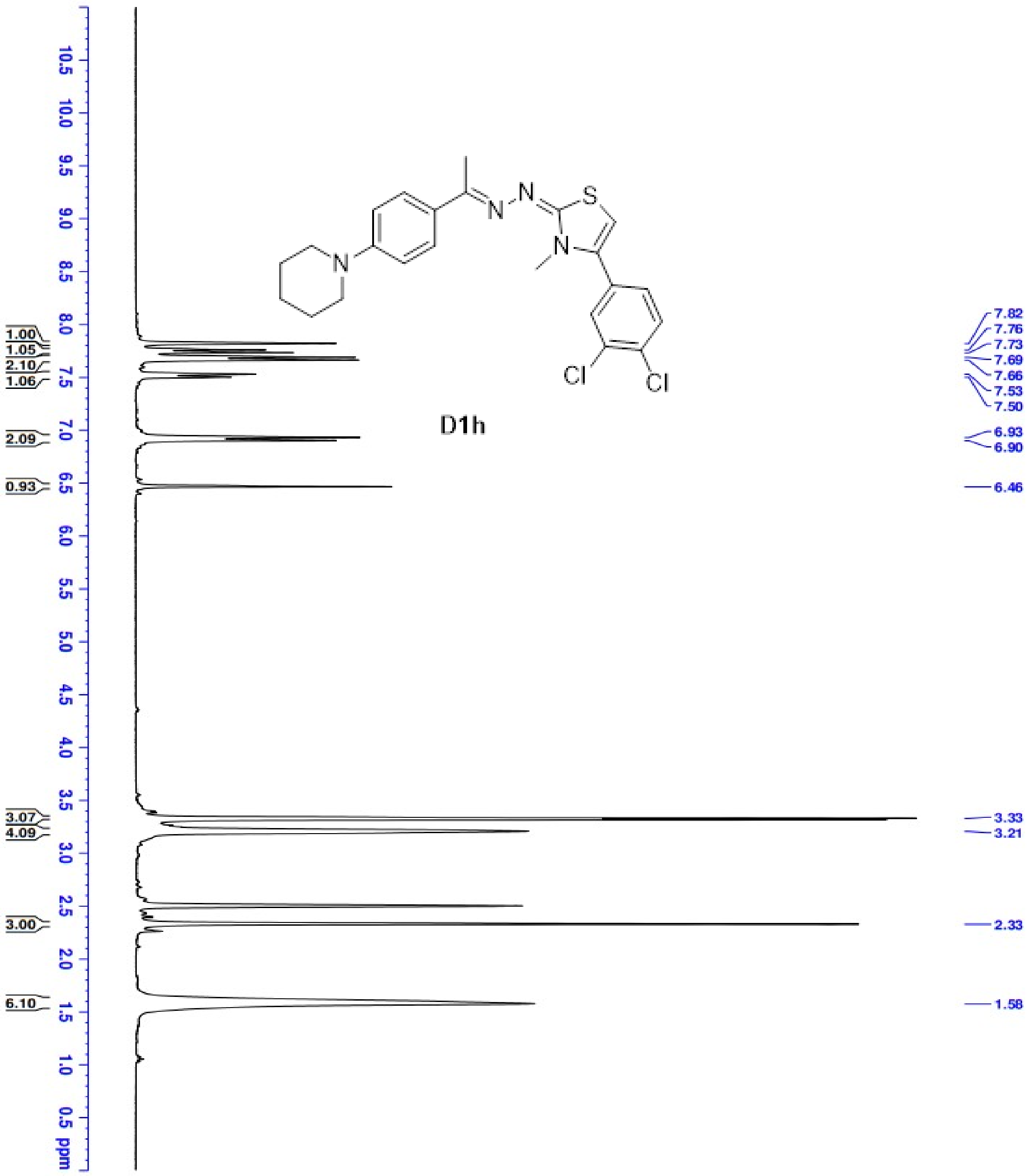

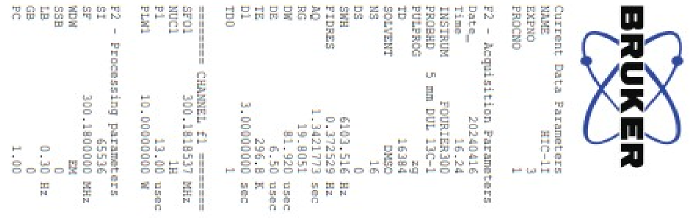

Figure 29S.^{1}HNMR spectrum of **D1h**. F.

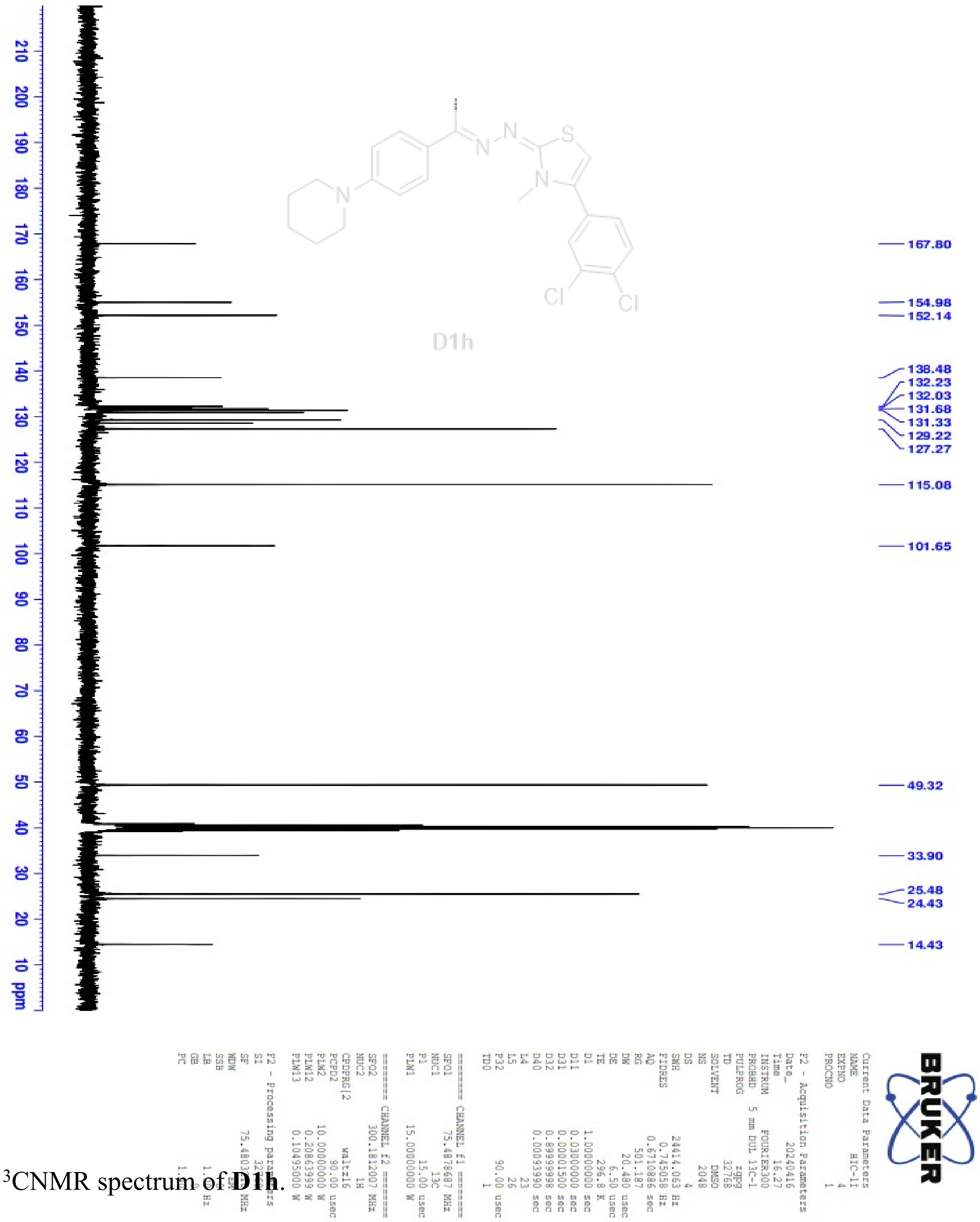

Figure 30S.^{13}CNMR spectrum of **D1h**.

Data File: C:\LabSolutions\Data\Analiz\derya\HIC-1I UST_588.lcd

Elmt	Val.	Min	Max	Elmt	Val.	Min	Max	Elmt	Val.	Min	Max	Elmt	Val.	Min	Max	Use Adduct
H	1	8	33	O	2	0	3	S	2	0	2	Ru	2	0	0	H
C	4	4	32	F	1	0	0	Cl	1	2	2	Pd	2	0	0	Na
N	3	0	6	P	3	0	0	Br	1	0	0	I	3	0	0	

Error Margin (ppm): 5
HC Ratio: unlimited
Max Isotopes: 3
MSn Iso RI (%): 10.00

DBE Range: 0.0 - 30.0
Apply N Rule: no
Isotope RI (%): 1.00
MSn Logic Mode: AND

Electron Ions: both
Use MSn Info: yes
Isotope Res: 9000
Max Results: 50

Event#: 1 MS(E+) Ret. Time : 1.787 Scan# : 269

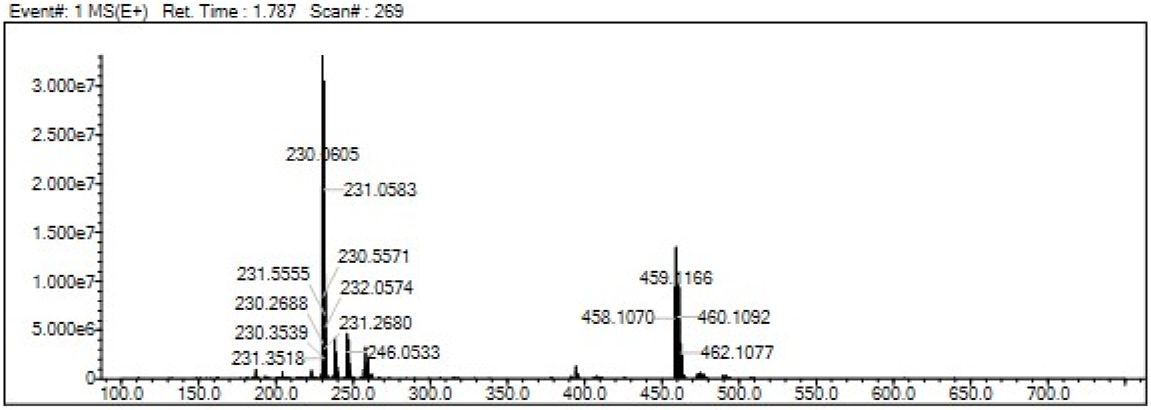

Measured region for 459.1166 m/z

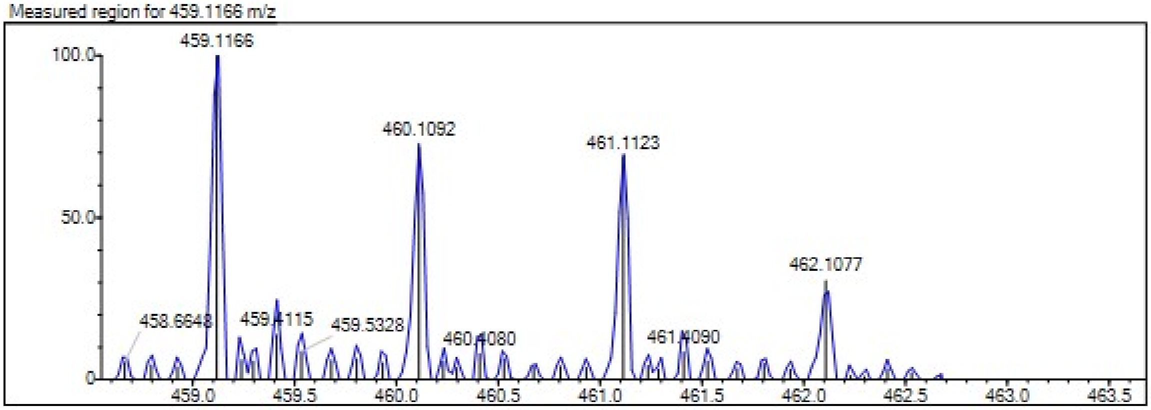

C23 H24 N4 S Cl2 [M+H]+ : Predicted region for 459.1171 m/z

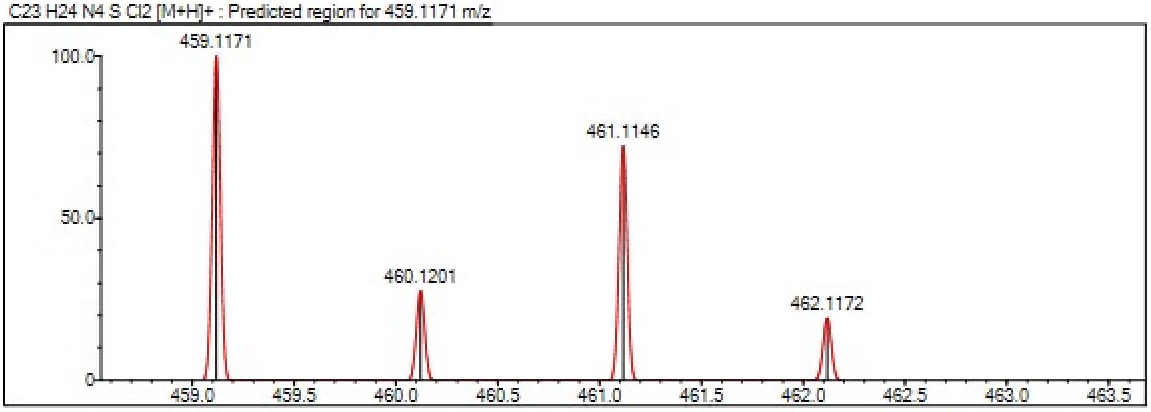

Rank	Score	Formula (M)	Ion	Meas. m/z	Pred. m/z	Df. (mDa)	Df. (ppm)	Iso	DBE
8	26.59	C23 H24 N4 S Cl2	[M+H]+	459.1166	459.1171	-0.5	-1.09	26.65	13.0

Figure 31S. HRMS spectrogram of **D1h**.

	Item	Value
	Acquired Date&Time	2.05.2024 14:48:21
	Acquired by	System Administrator
	Filename	C:\Users\dopnalab\Desktop\MASAÜSTÜ\sazan\hic\hic-1j1.ispd
	Spectrum name	hic-1j1
	Sample name	hic-1j
	Sample ID	
	Option	
	Comment	
	No. of Scans	30
	Resolution	4 [cm-1]
	Apodization	Happ-Genzel

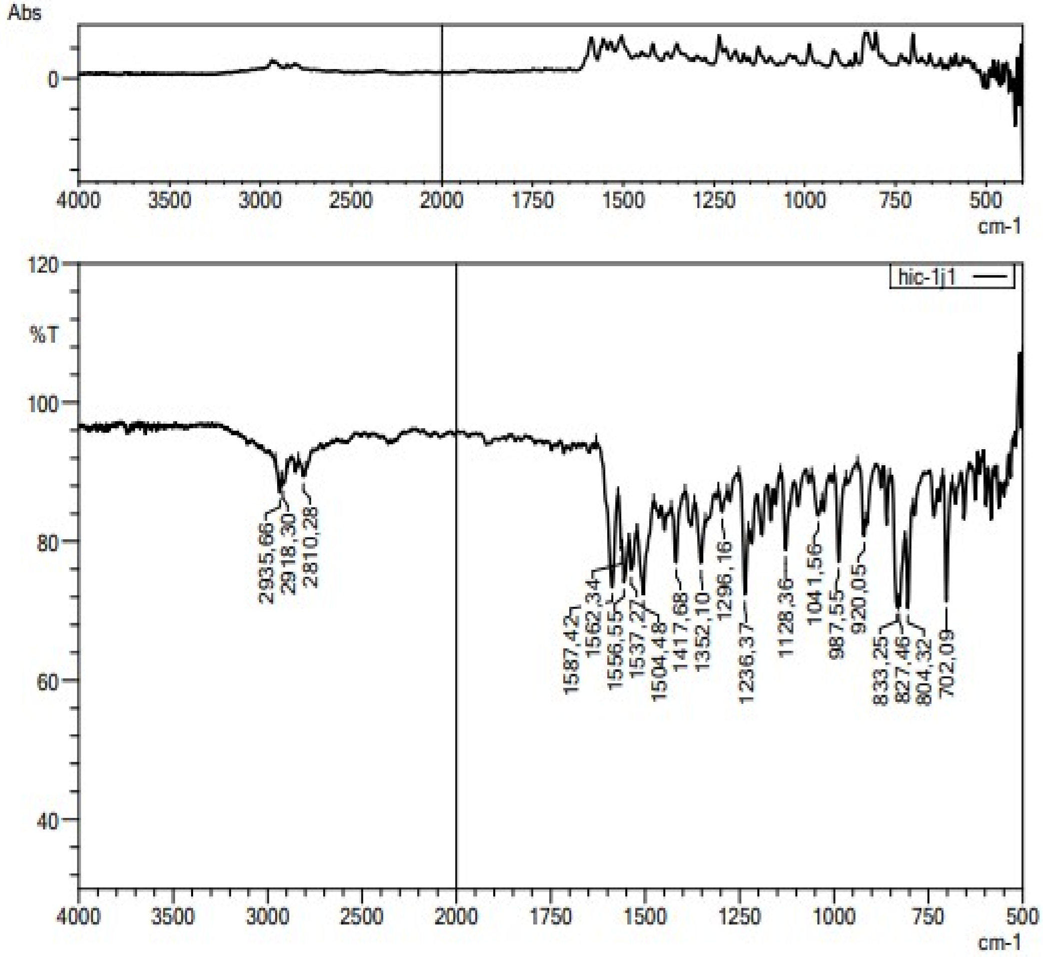

Figure 32S. IR fingerprint of **D1i**.

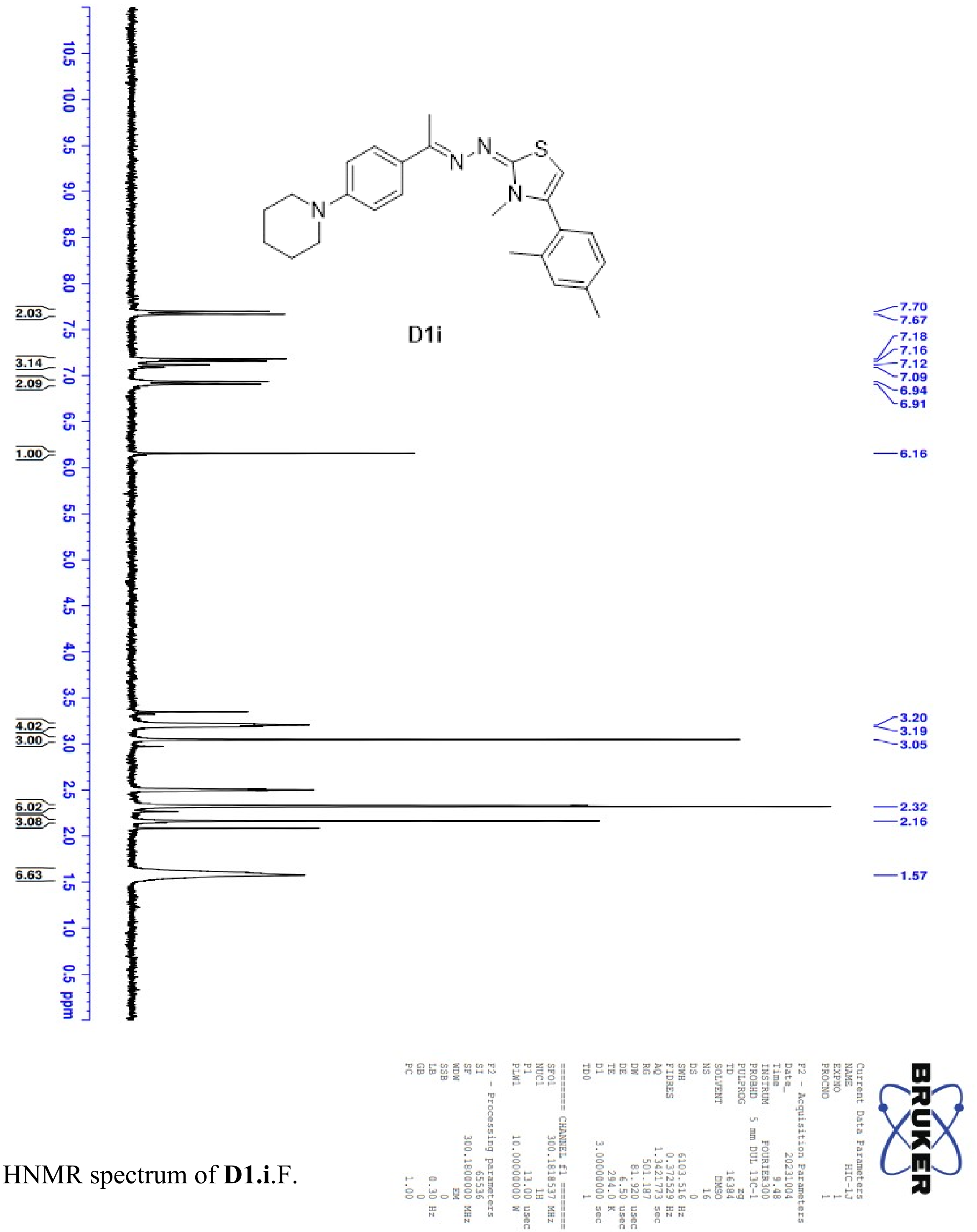

Figure 33S. ^{1}HNMR spectrum of **D1.i**.F.

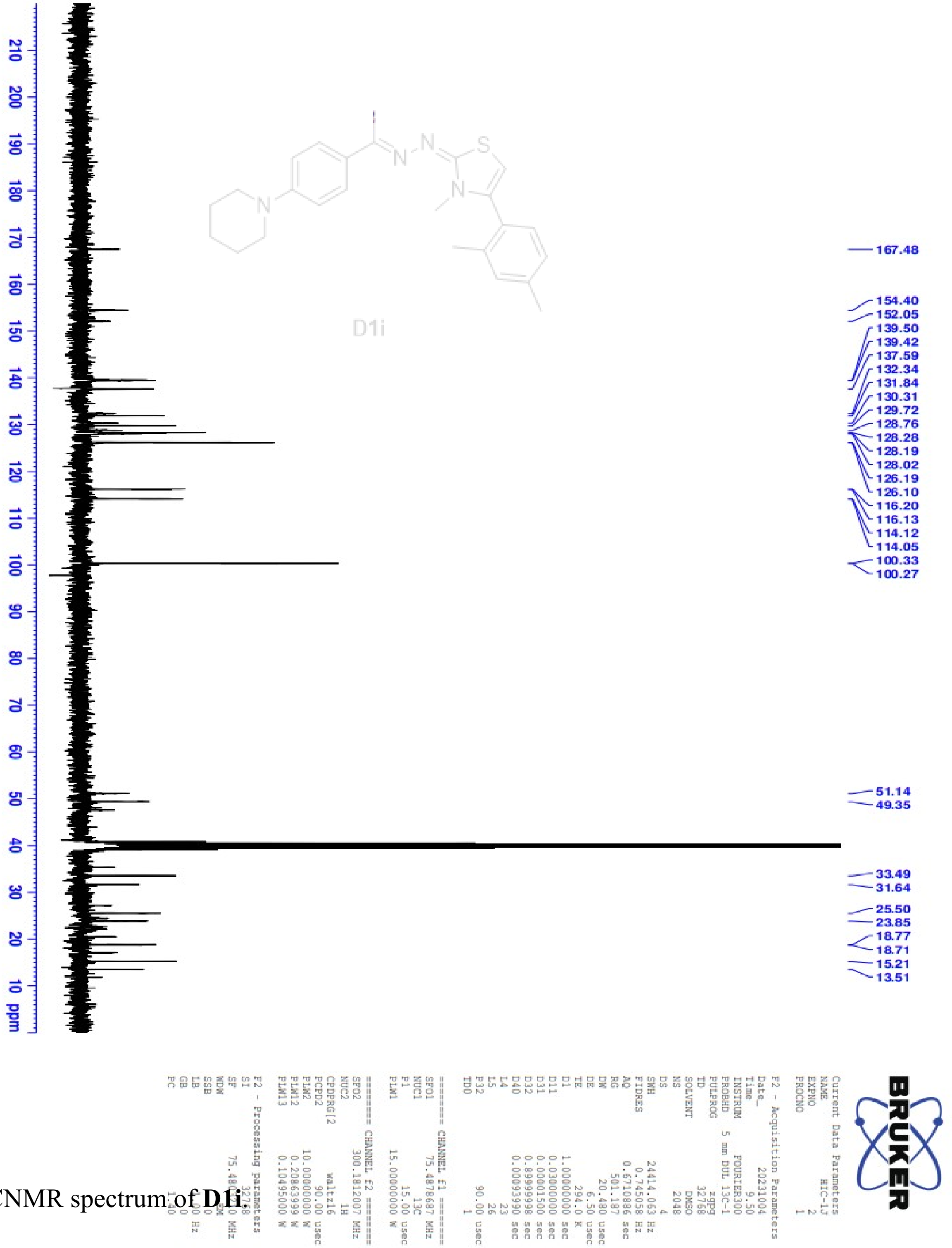

Figure 34S. ^{13}CNMR spectrum of **D1i**.

Data File: C:\LabSolutions\Data\Analiz\derya\HIC-1J ALT_591.lcd

Elmt	Val.	Min	Max	Elmt	Val.	Min	Max	Elmt	Val.	Min	Max	Elmt	Val.	Min	Max	Use Adduct
H	1	8	33	O	2	0	3	S	2	0	2	Ru	2	0	0	H
C	4	4	32	F	1	0	0	Cl	1	0	0	Pd	2	0	0	Na
N	3	0	6	P	3	0	0	Br	1	0	0	I	3	0	0	

Error Margin (ppm): 5
HC Ratio: unlimited
Max Isotopes: 3
MSn Iso RI (%): 10.00

DBE Range: 0.0 - 30.0
Apply N Rule: no
Isotope RI (%): 1.00
MSn Logic Mode: AND

Electron Ions: both
Use MSn Info: yes
Isotope Res: 9000
Max Results: 50

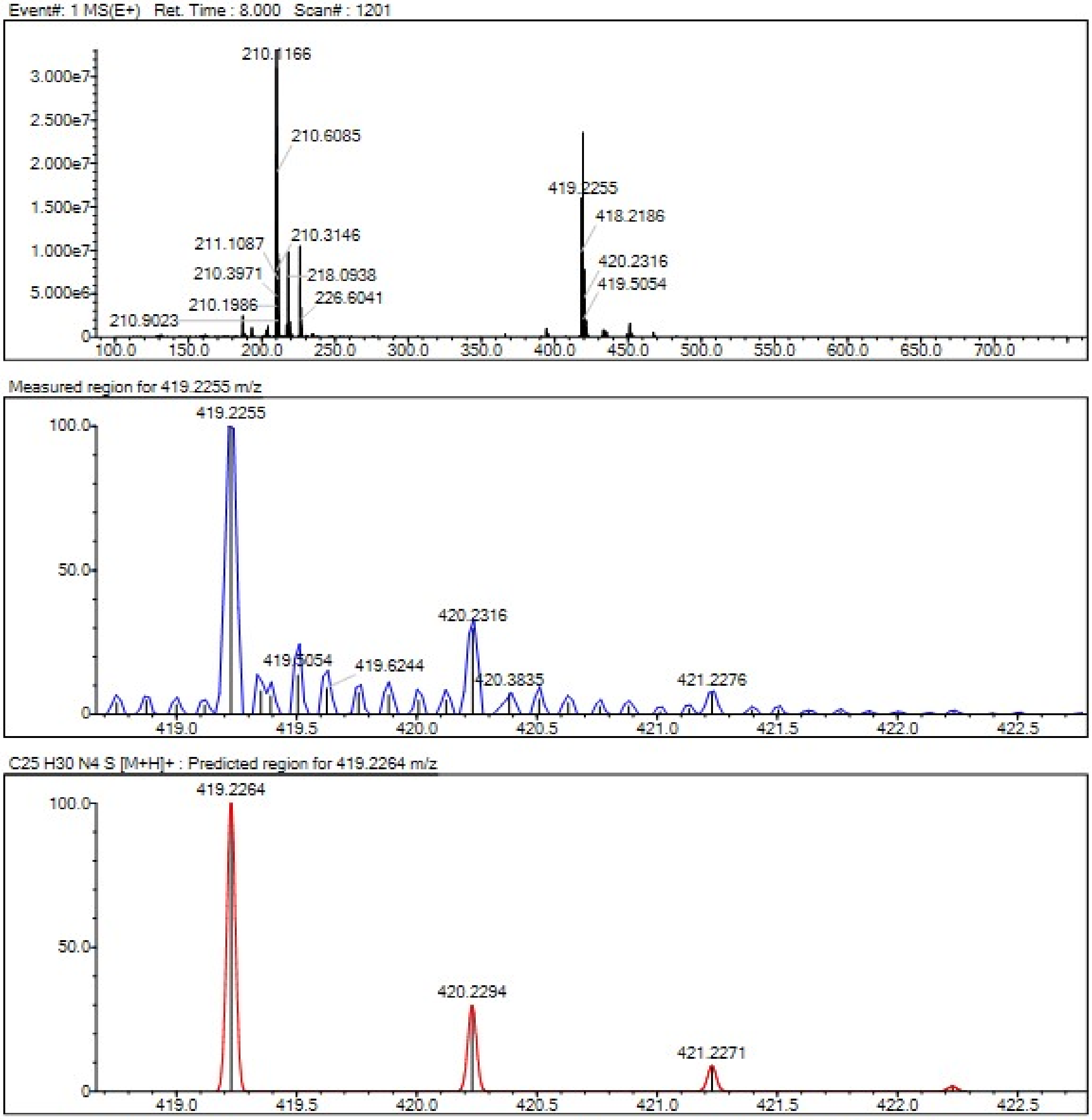

Rank	Score	Formula (M)	Ion	Meas. m/z	Pred. m/z	Df. (mDa)	Df. (ppm)	Iso	DBE
1	85.65	C25 H30 N4 S	[M+H]+	419.2255	419.2264	-0.9	-2.15	88.19	13.0

Figure 35S. HRMS spectrogram of **D1i**.F

Item	Value
Acquired Date&Time	3.05.2024 10:32:16
Acquired by	System Administrator
Filename	C:\Users\dopnalab\Desktop\MASAÜSTÜ\sazan\hic\hic-2a1.ispd
Spectrum name	hic-2a1
Sample name	hic-2a
Sample ID	
Option	
Comment	
No. of Scans	30
Resolution	4 [cm-1]
Apodization	Happ-Genzel

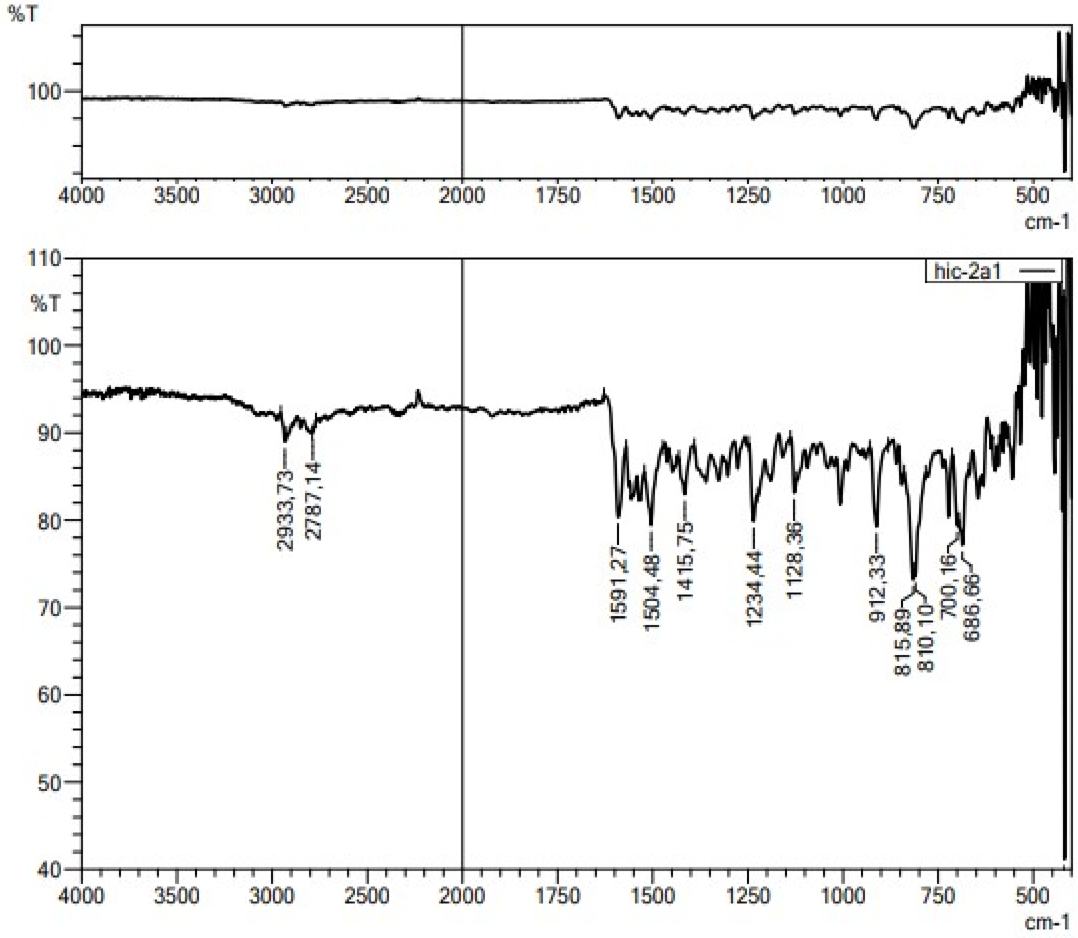

Figure 36S. IR fingerprint of **D2a**.*F*

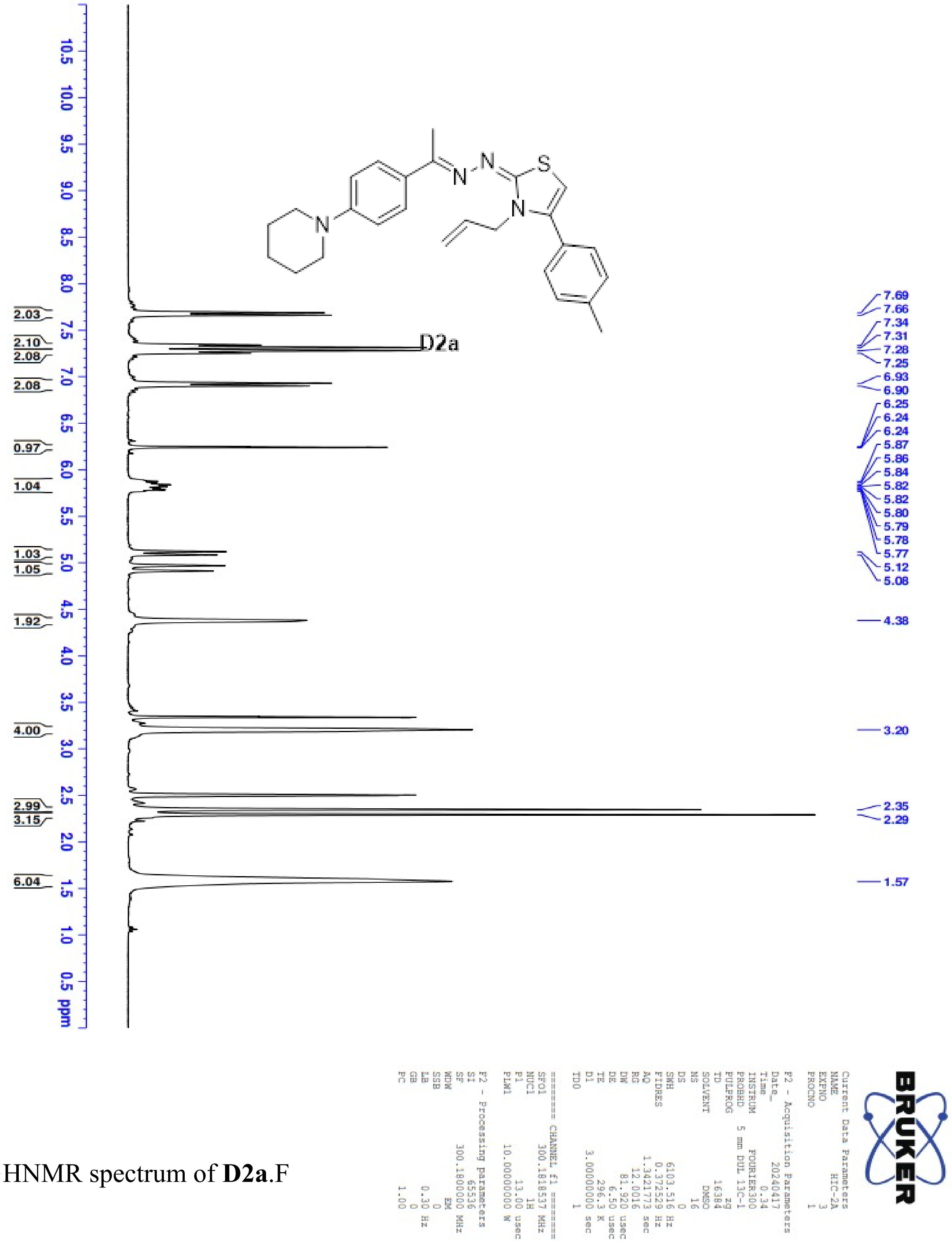

Figure 37S. ^{1}HNMR spectrum of **D2a**.F

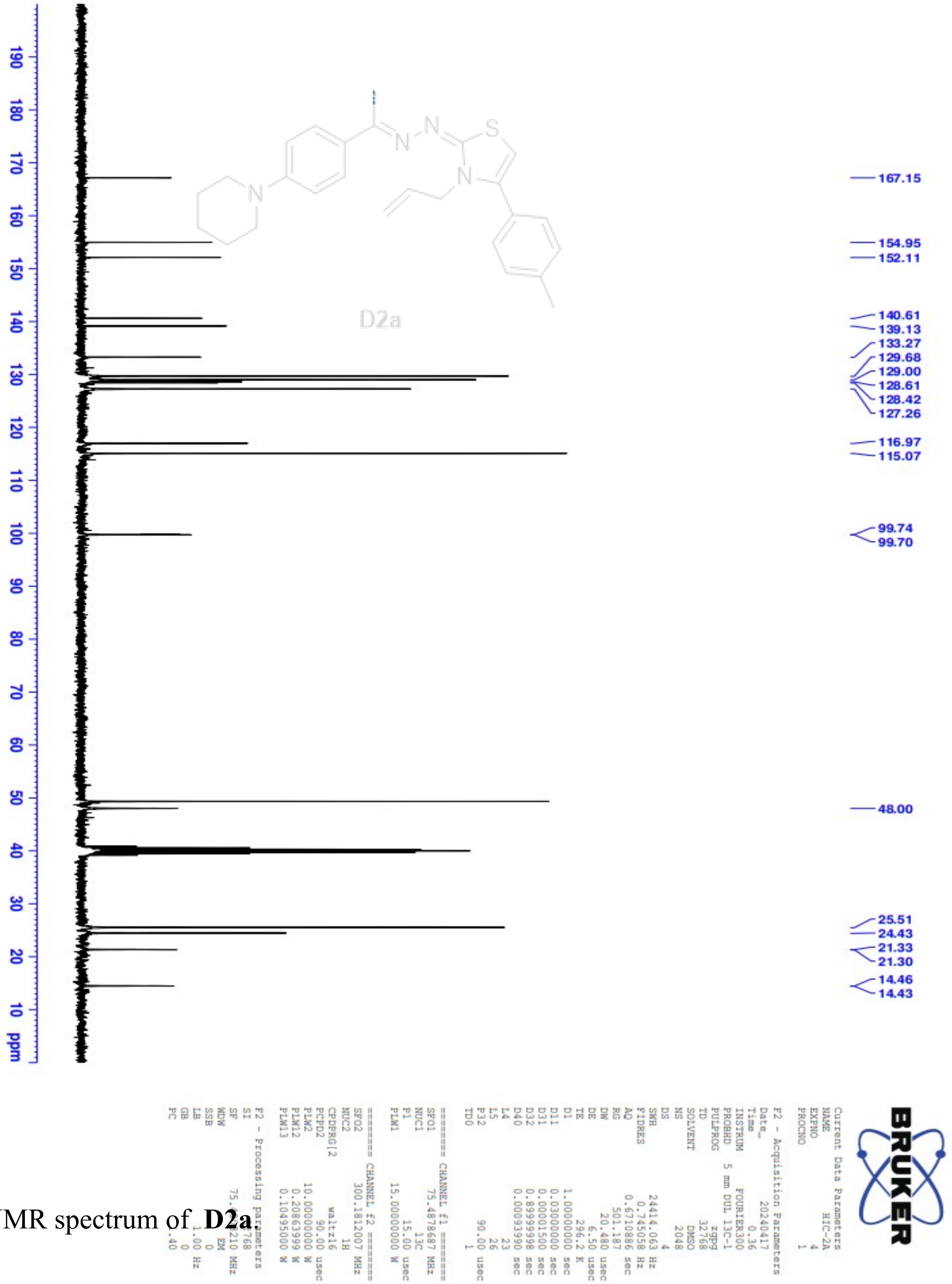

Figure 38S. ^{13}CNMR spectrum of **D2a.**

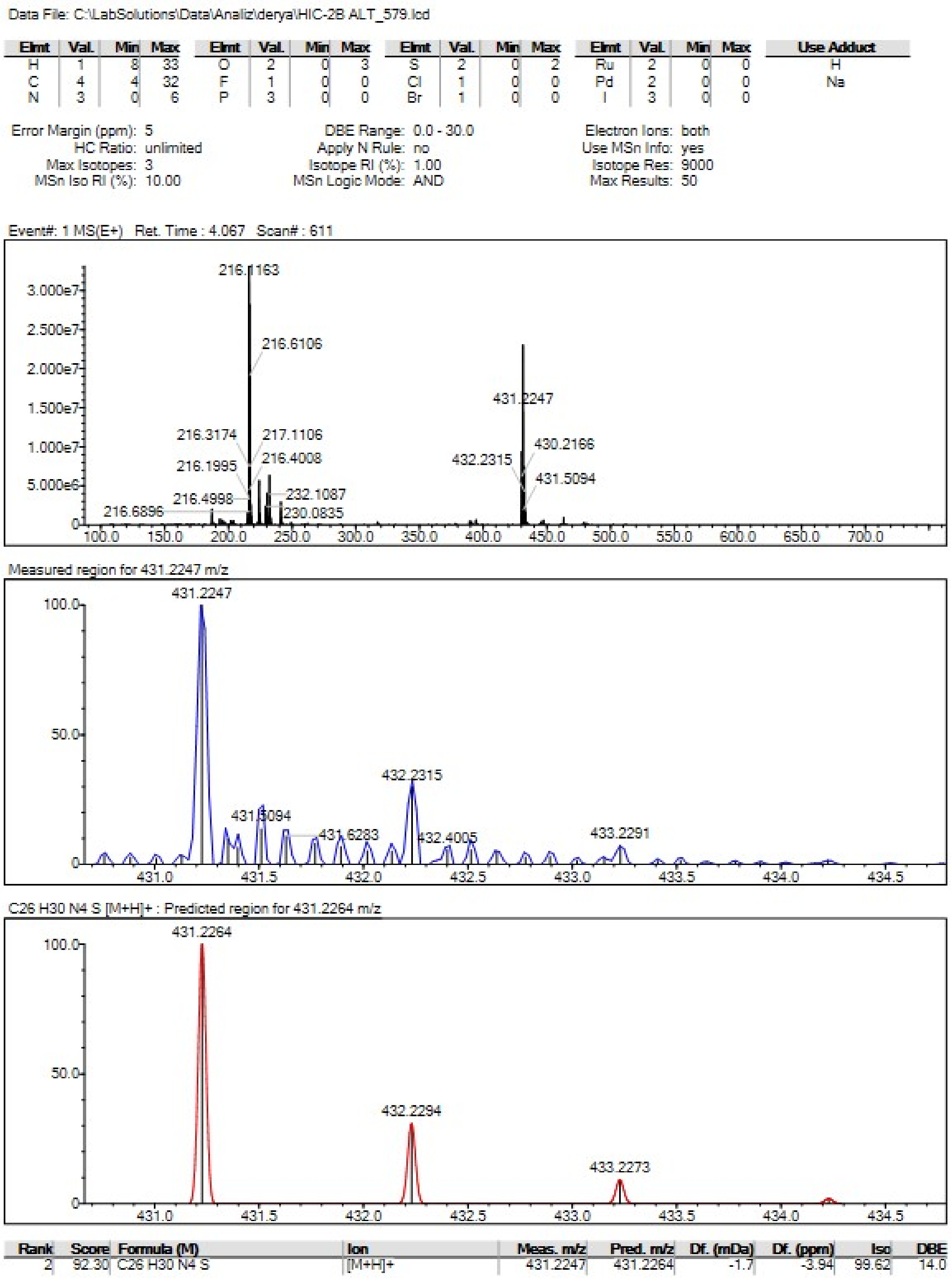

Figure 39S. HRMS spectrogram of **D2a**.

Item	Value
Acquired Date&Time	3.05.2024 10:37:27
Acquired by	System Administrator
Filename	C:\Users\dopnalab\Desktop\MASAÜSTÜ\sazan\hic\hic-2b1.ispd
Spectrum name	hic-2b1
Sample name	hic-2b
Sample ID	
Option	
Comment	
No. of Scans	30
Resolution	4 [cm-1]
Apodization	Happ-Genzel

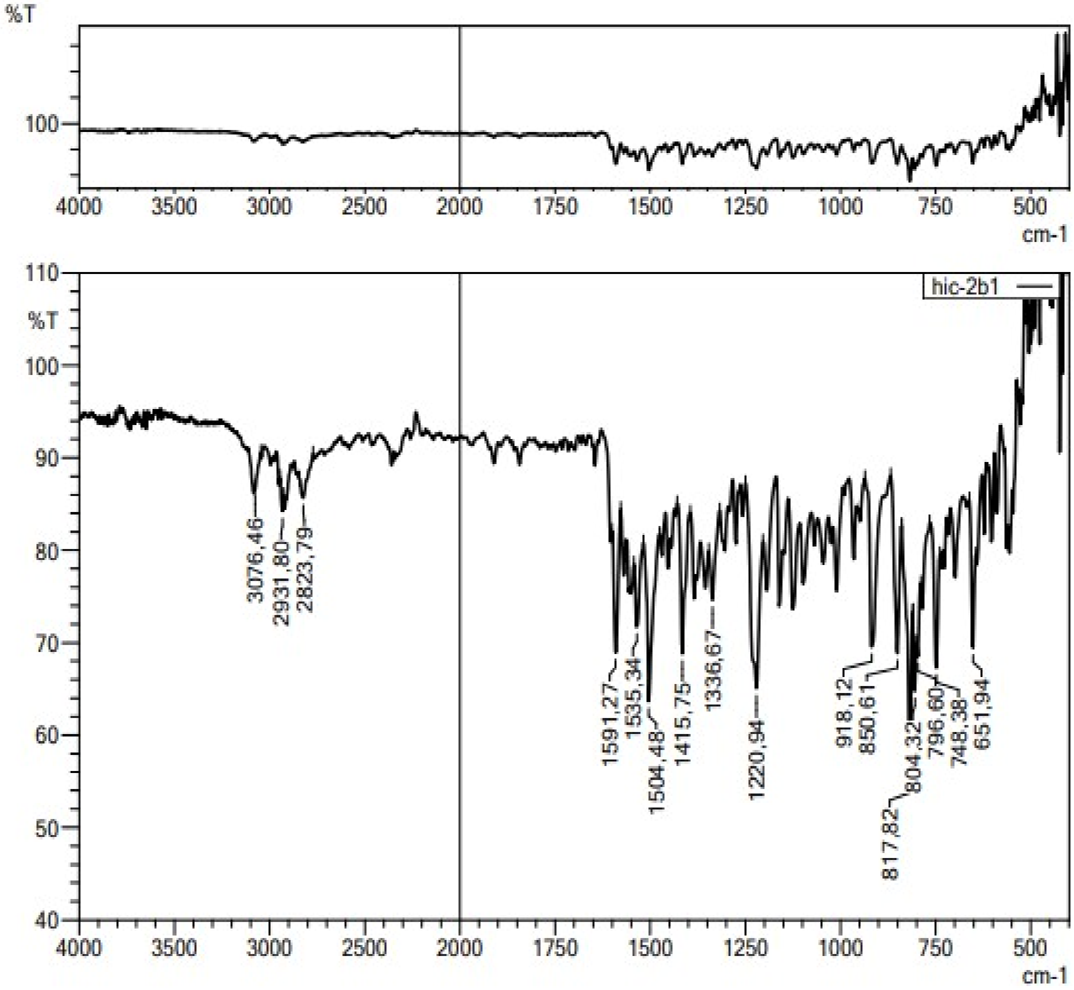

Figure 40S. IR fingerprint of **D2b**.F

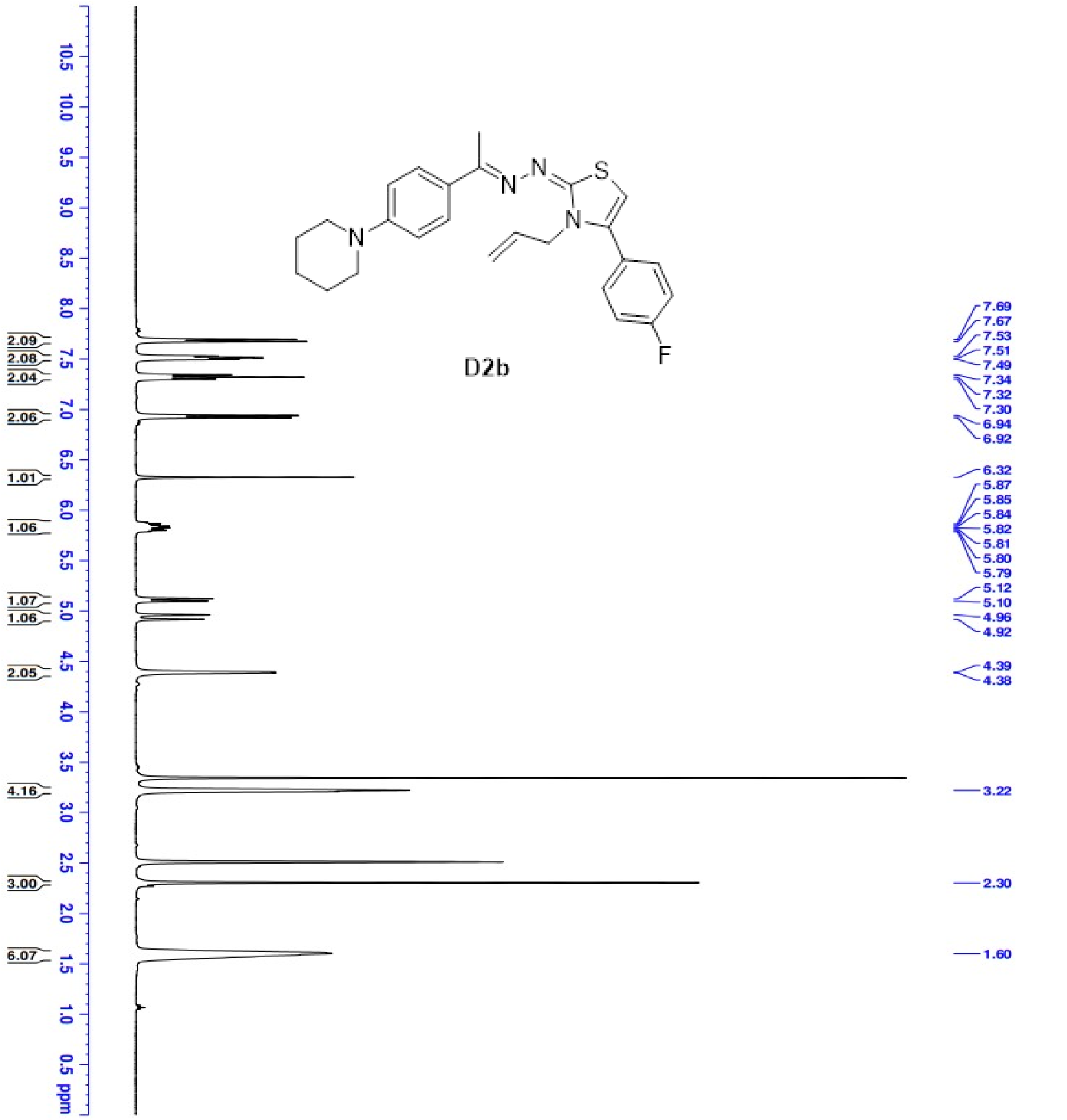

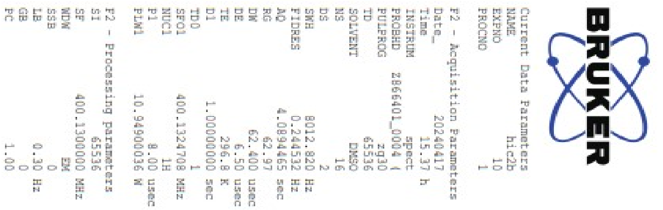

Figure 41S. ^{1}HNMR spectrum of **D2b**.F

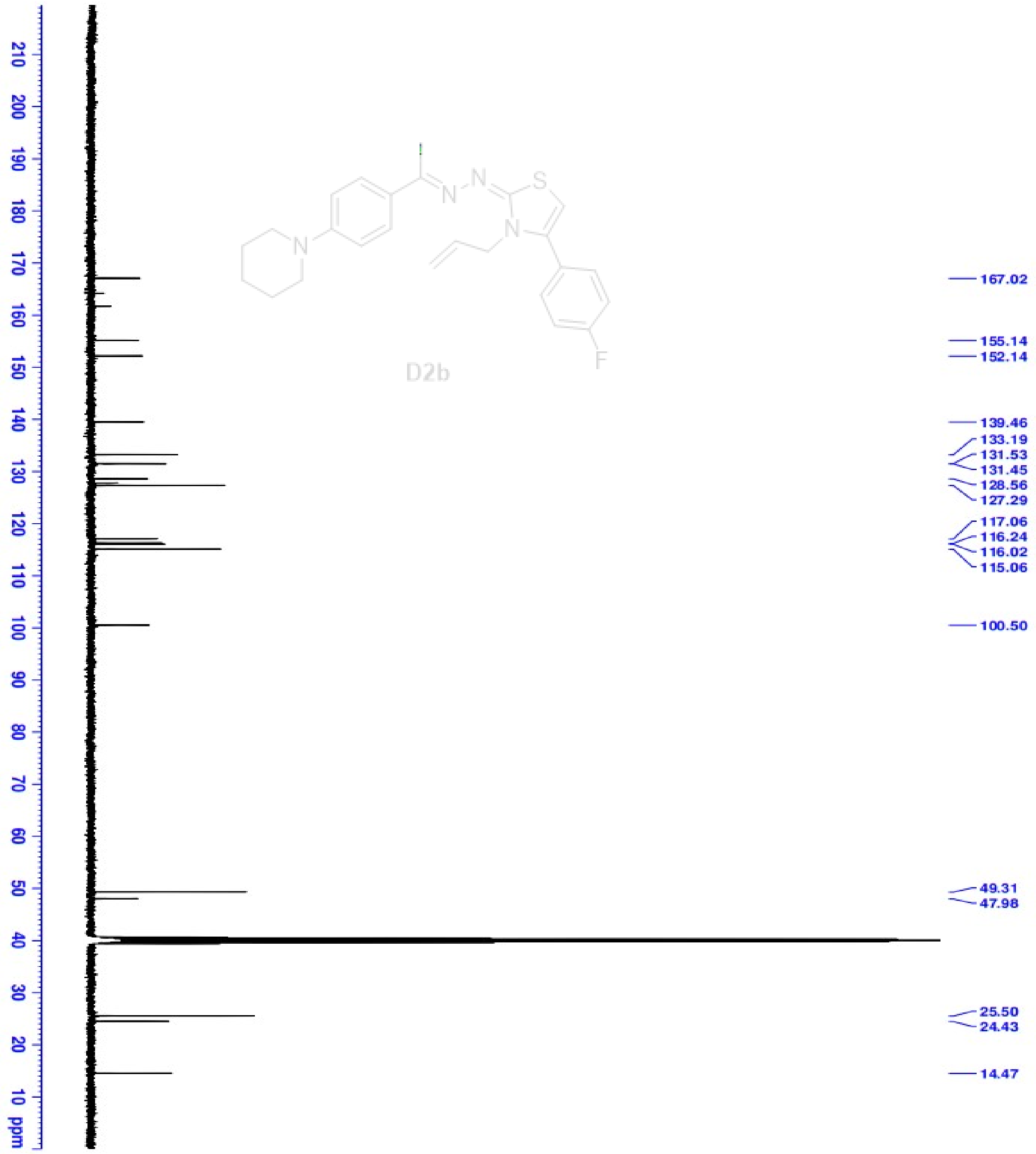

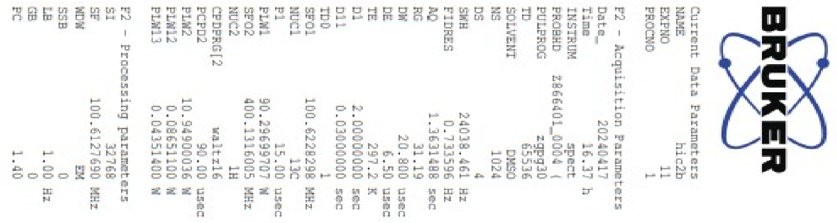

Figure 42S. ^{13}CNMR spectrum of **D2b**.

Data File: C:\LabSolutions\Data\Analiz\derya\HIC-2C UST_580.lcd

Elmt	Val.	Min	Max	Elmt	Val.	Min	Max	Elmt	Val.	Min	Max	Elmt	Val.	Min	Max	Use Adduct
H	1	8	33	O	2	0	3	S	2	0	2	Ru	2	0	0	H
C	4	4	32	F	1	1	1	Cl	1	0	0	Pd	2	0	0	Na
N	3	0	6	P	3	0	0	Br	1	0	0	I	3	0	0	

Error Margin (ppm): 5
HC Ratio: unlimited
Max Isotopes: 3
MSn Iso RI (%): 10.00

DBE Range: 0.0 - 30.0
Apply N Rule: no
Isotope RI (%): 1.00
MSn Logic Mode: AND

Electron Ions: both
Use MSn Info: yes
Isotope Res: 9000
Max Results: 50

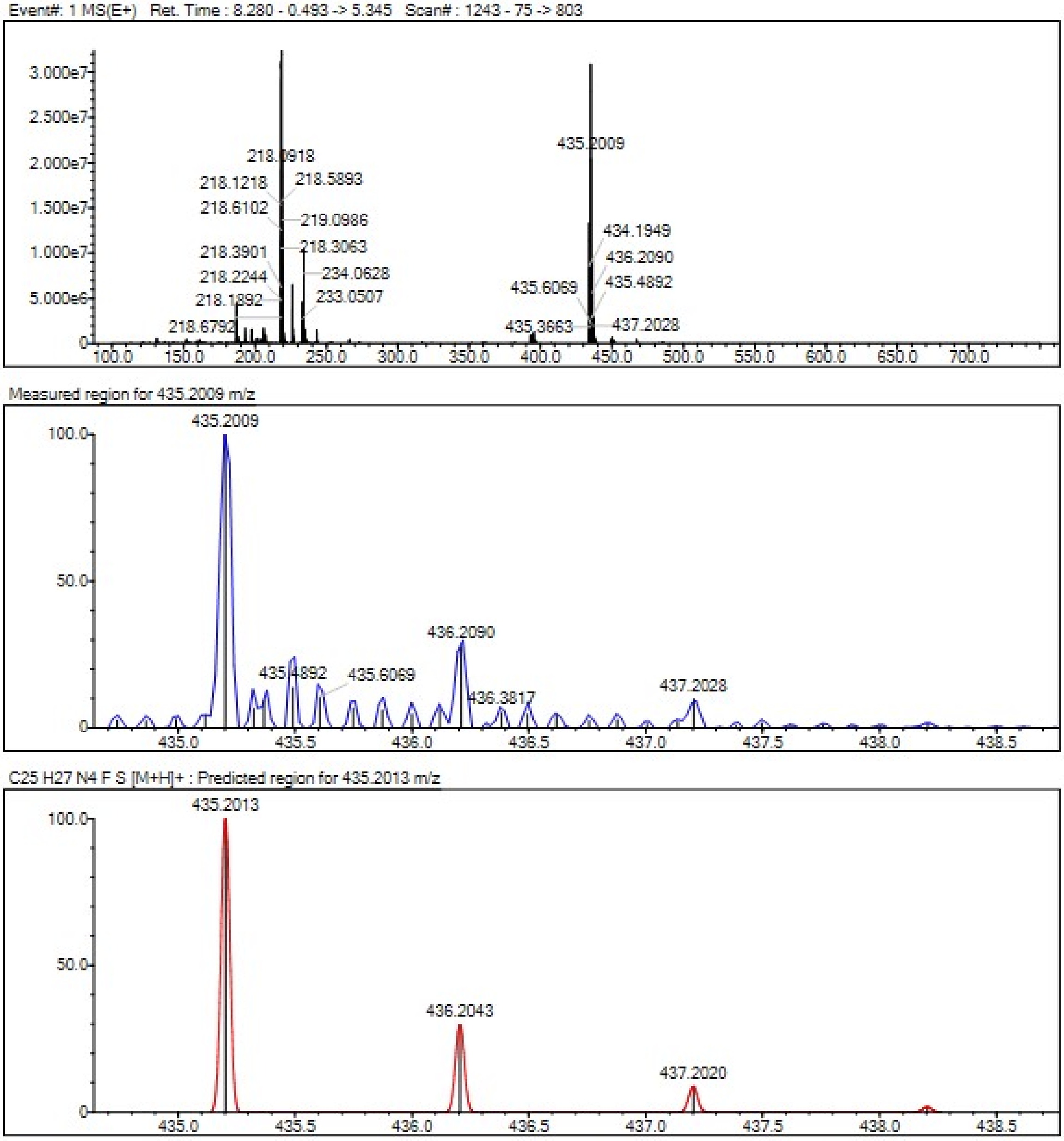

Rank	Score	Formula (M)	Ion	Meas. m/z	Pred. m/z	Df. (mDa)	Df. (ppm)	Iso	DBE
2	90.52	C25 H27 N4 F S	[M+H]+	435.2009	435.2013	-0.4	-0.92	90.52	14.0

Figure 43S. HRMS spectrogram of **D2b**.F

Item	Value
Acquired Date&Time	3.05.2024 10:42:36
Acquired by	System Administrator
Filename	C:\Users\dopnalab\Desktop\MASAÜSTÜ\sazan\hic\hic-2c1.ispd
Spectrum name	hic-2c1
Sample name	hic-2c
Sample ID	
Option	
Comment	
No. of Scans	30
Resolution	4 [cm-1]
Apodization	Happ-Genzel

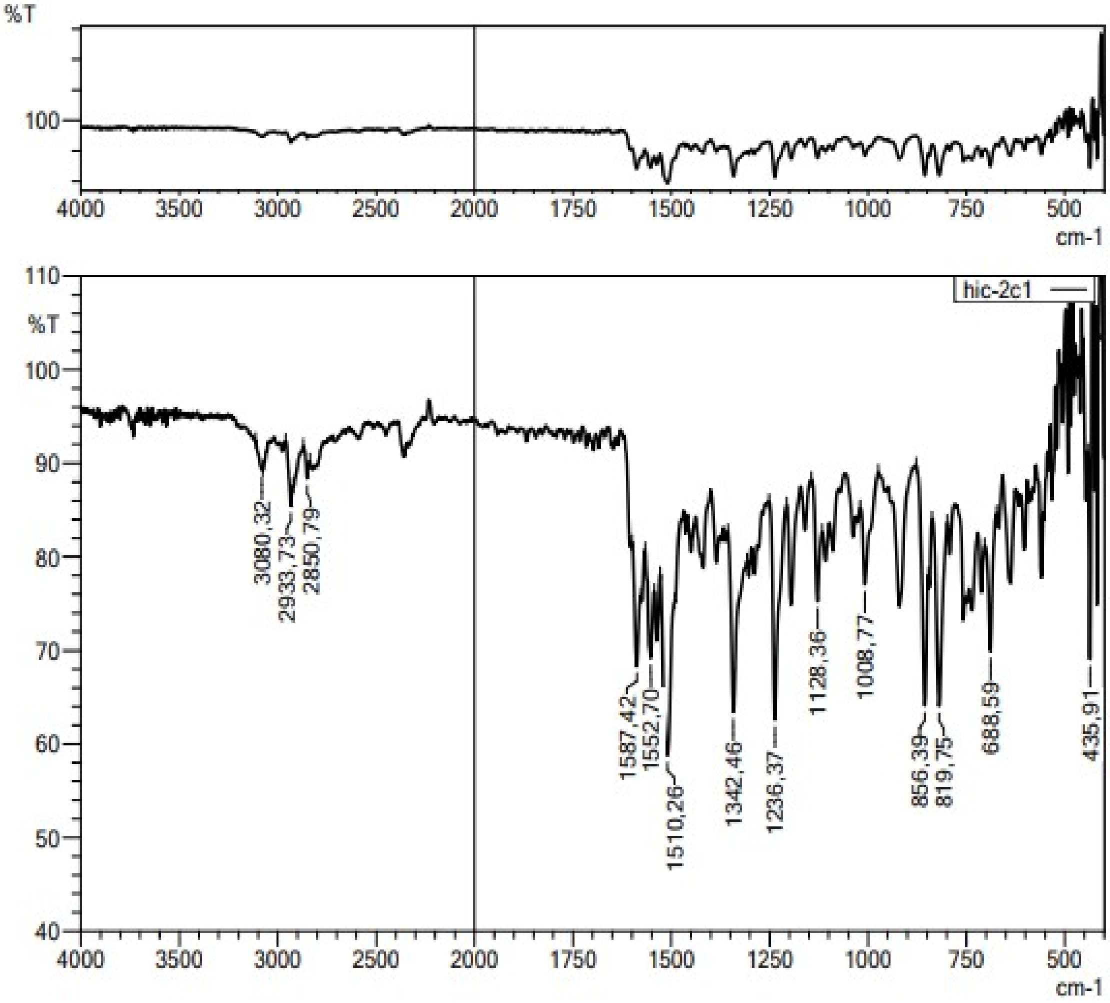

Figure 44S. IR fingerprint of **D2c**.F

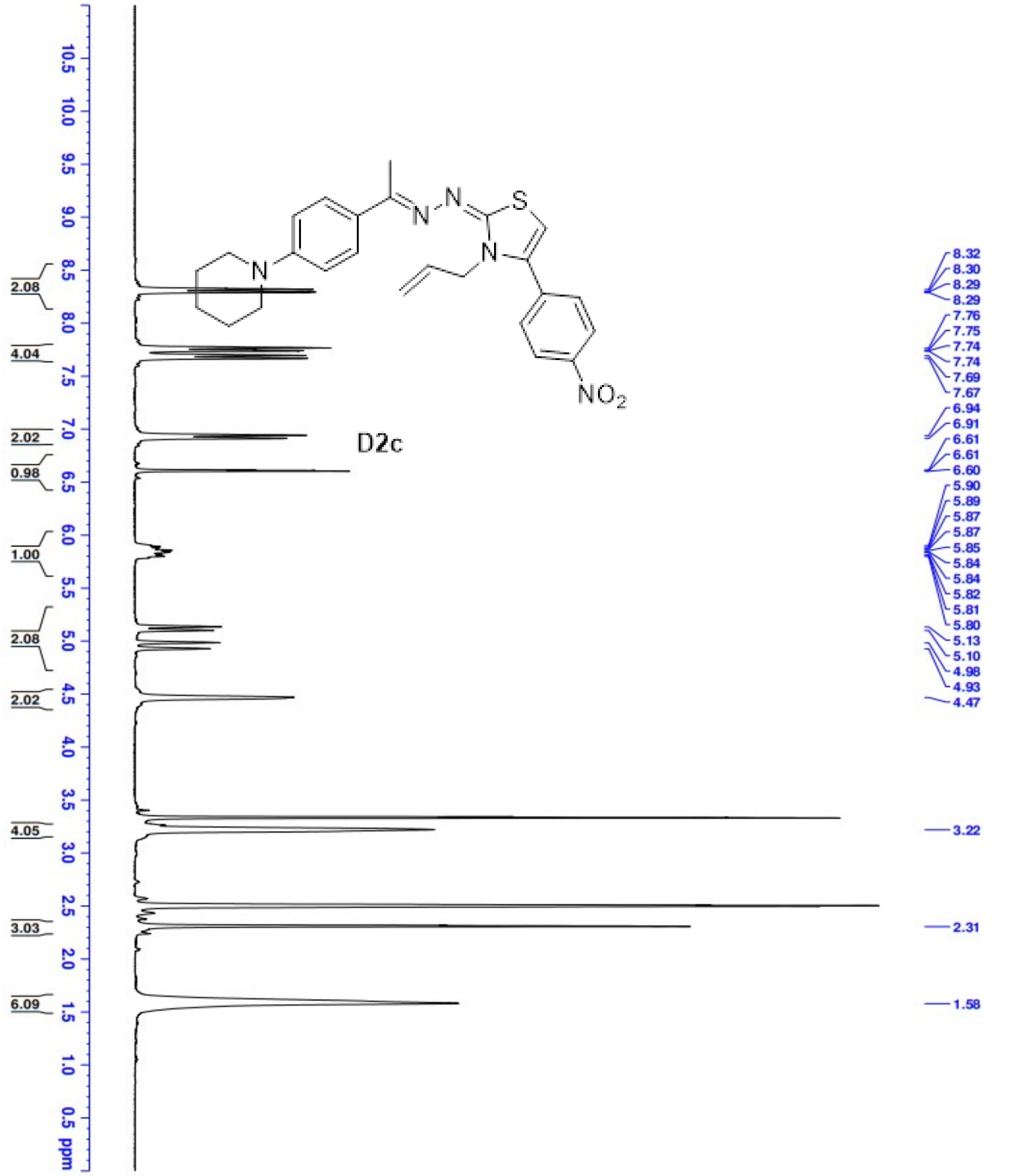

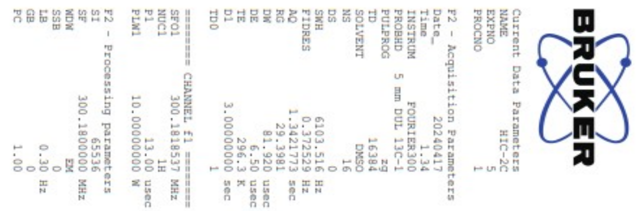

Figure 45S. ^{1}HNMR spectrum of **D2c**.F

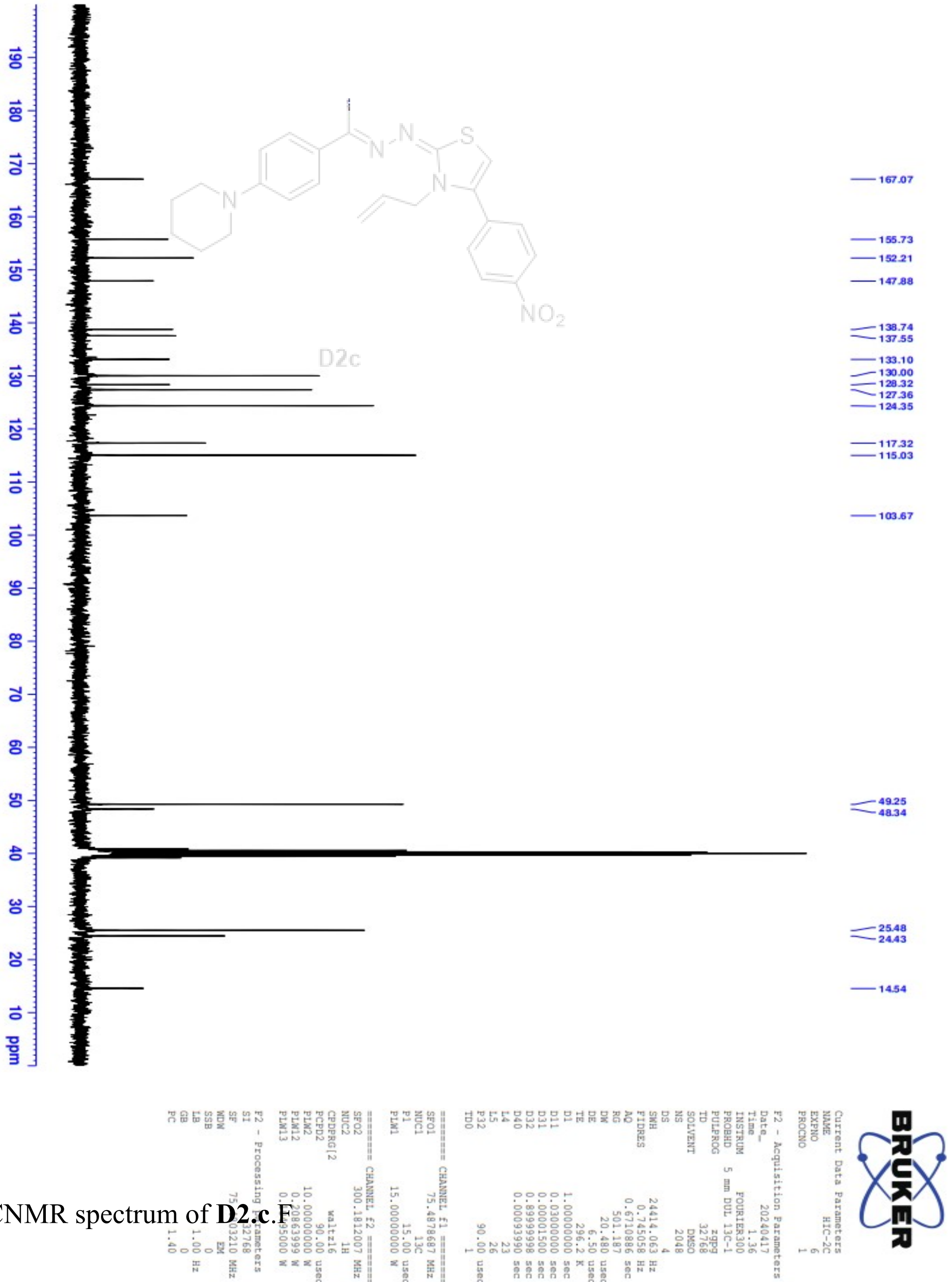

Figure 46S. ^{13}CNMR spectrum of **D2.c**.F

Data File: C:\LabSolutions\Data\Analiz\derya\HIC-2C ALT_581.lcd

Elmt	Val.	Min	Max	Elmt	Val.	Min	Max	Elmt	Val.	Min	Max	Elmt	Val.	Min	Max	Use Adduct
H	1	8	33	O	2	0	3	S	2	0	2	Ru	2	0	0	H
C	4	4	32	F	1	0	0	Cl	1	0	0	Pd	2	0	0	Na
N	3	0	6	P	3	0	0	Br	1	0	0	I	3	0	0	

Error Margin (ppm): 5
HC Ratio: unlimited
Max Isotopes: 3
MSn Iso RI (%): 10.00

DBE Range: 0.0 - 30.0
Apply N Rule: no
Isotope RI (%): 1.00
MSn Logic Mode: AND

Electron Ions: both
Use MSn Info: yes
Isotope Res: 9000
Max Results: 50

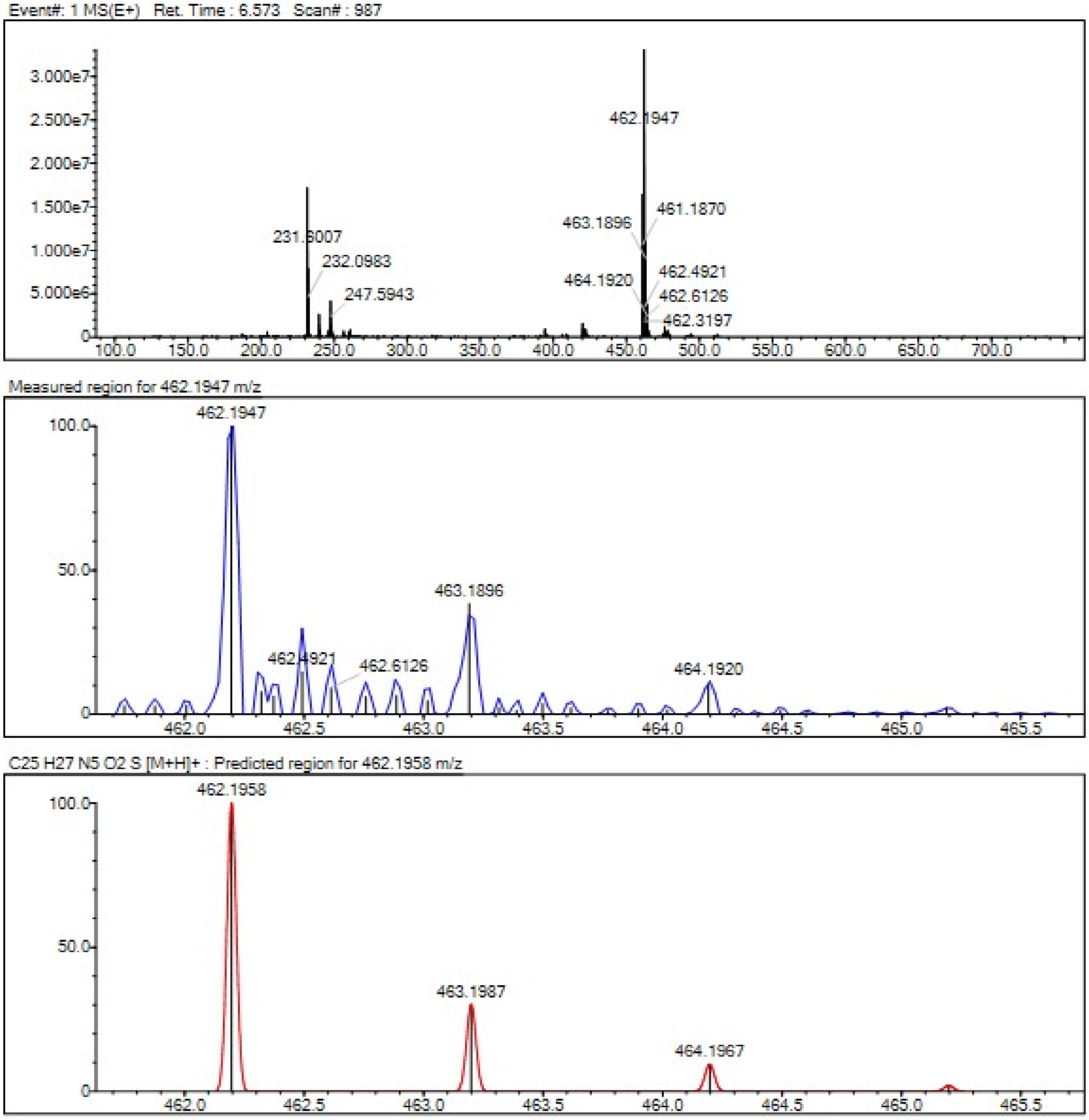

Rank	Score	Formula (M)	Ion	Meas. m/z	Pred. m/z	Df. (mDa)	Df. (ppm)	Iso	DBE
1	73.79	C25 H27 N5 O2 S	[M+H]+	462.1947	462.1958	-1.1	-2.38	76.42	15.0

Figure 47S. HRMS spectrogram of **D2c**.F

	Item	Value
	Acquired Date&Time	3.05.2024 10:47:11
	Acquired by	System Administrator
	Filename	C:\Users\dopnalab\Desktop\MASAÜSTÜ\sazan\hic\hic-2d1.ispd
	Spectrum name	hic-2d1
	Sample name	hic-2d
	Sample ID	
	Option	
	Comment	
	No. of Scans	30
	Resolution	4 [cm-1]
	Apodization	Happ-Genzel

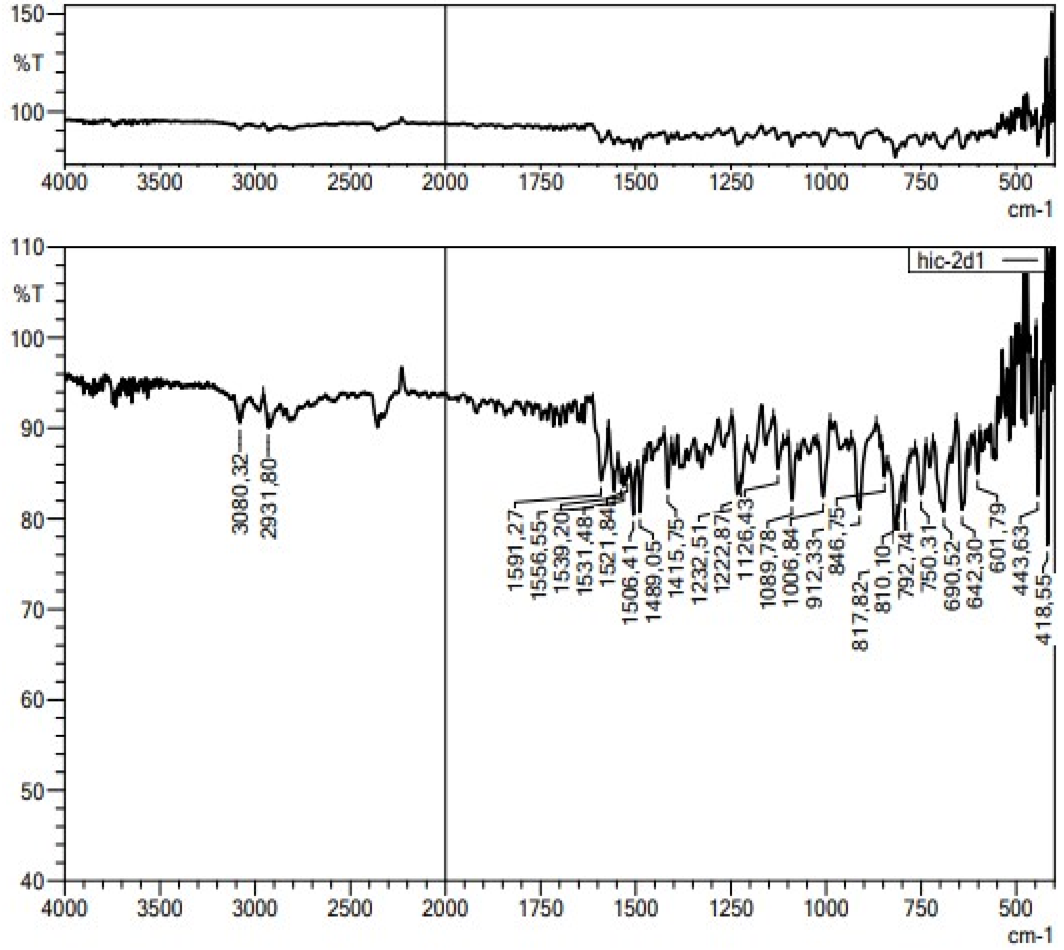

Figure 48S. IR fingerprint of **D2d.**F

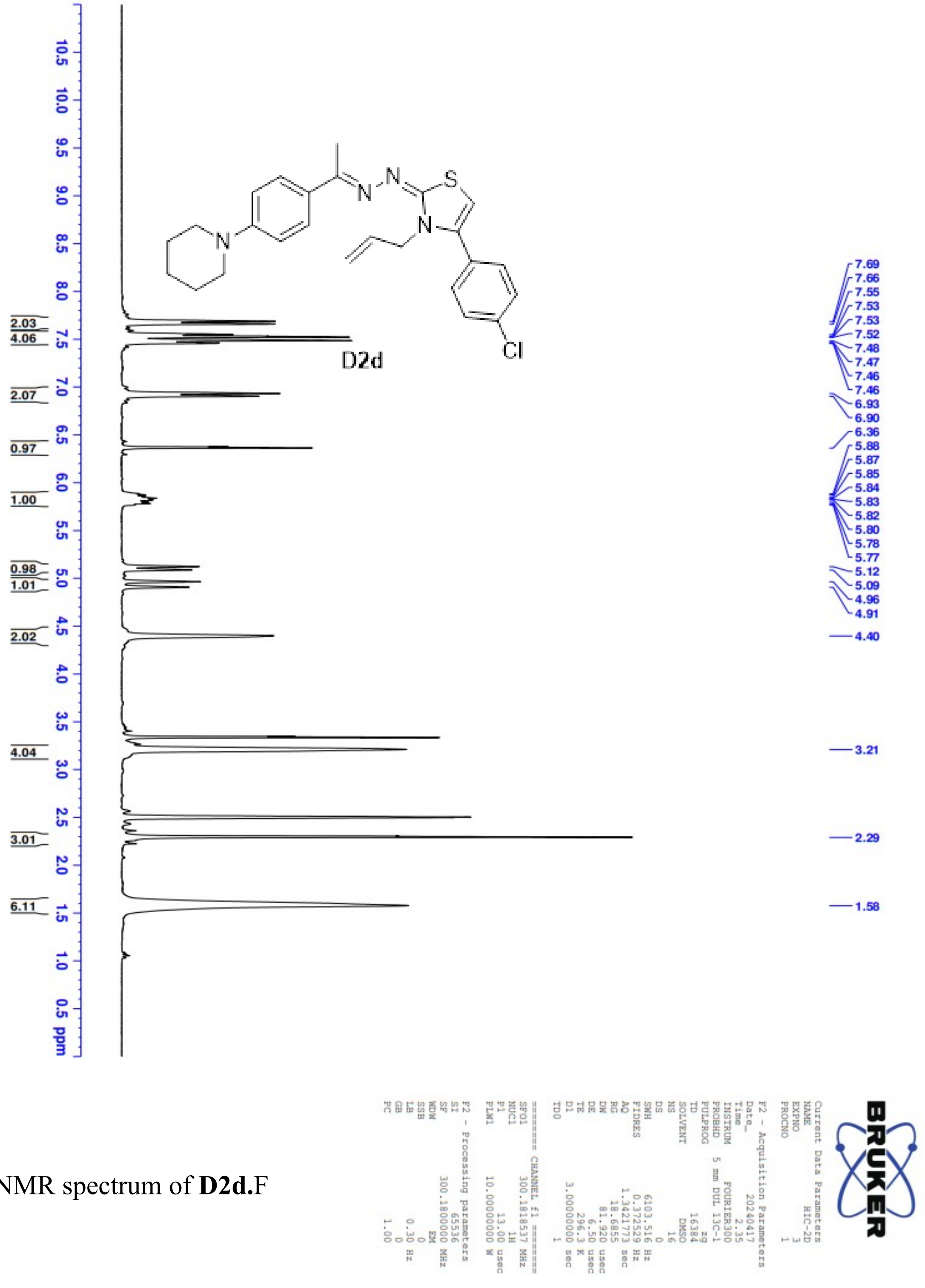

Figure 49S. :^{1}HNMR spectrum of **D2d.**F

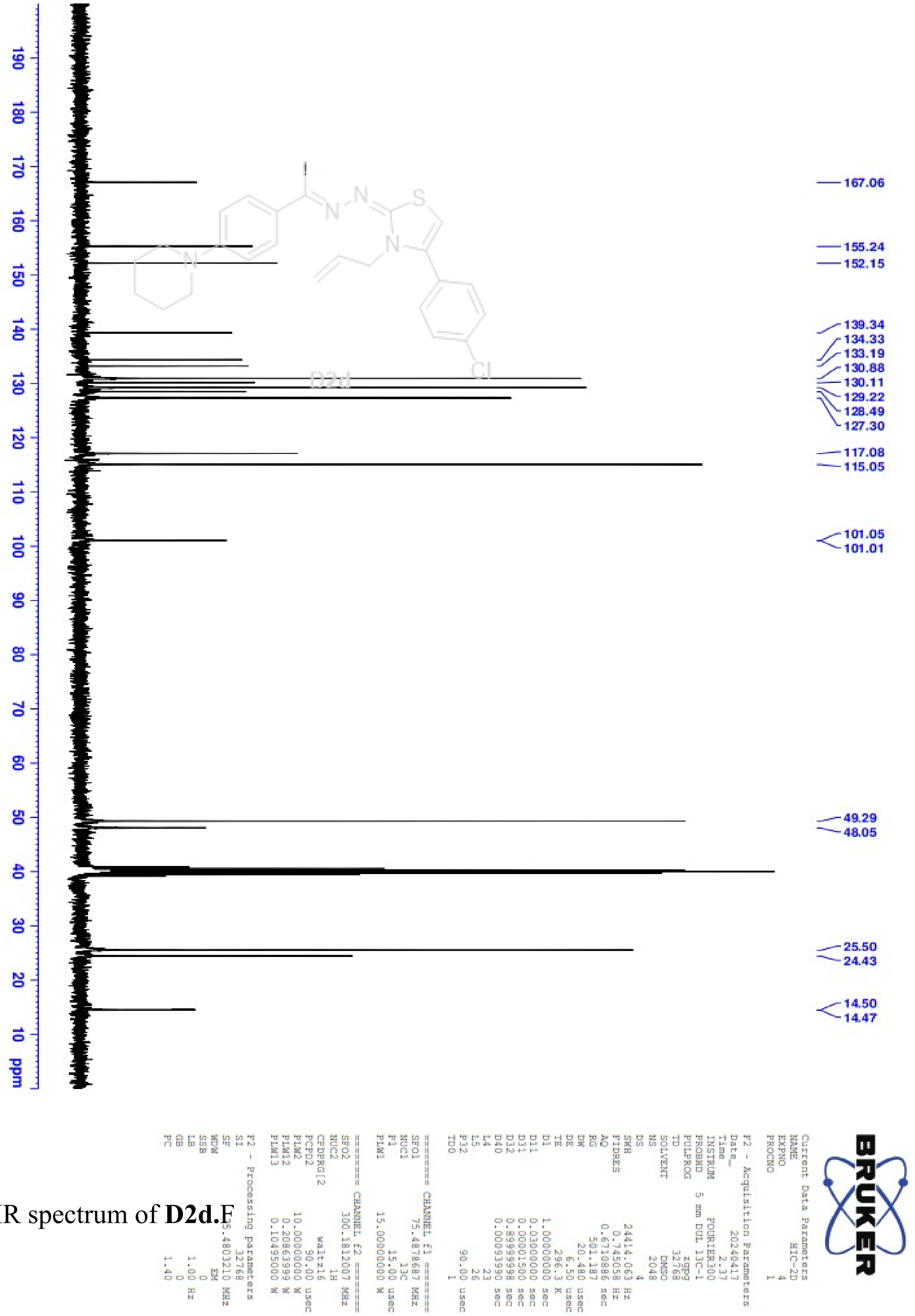

Figure 50S. ^{13}CNMR spectrum of **D2d.**F

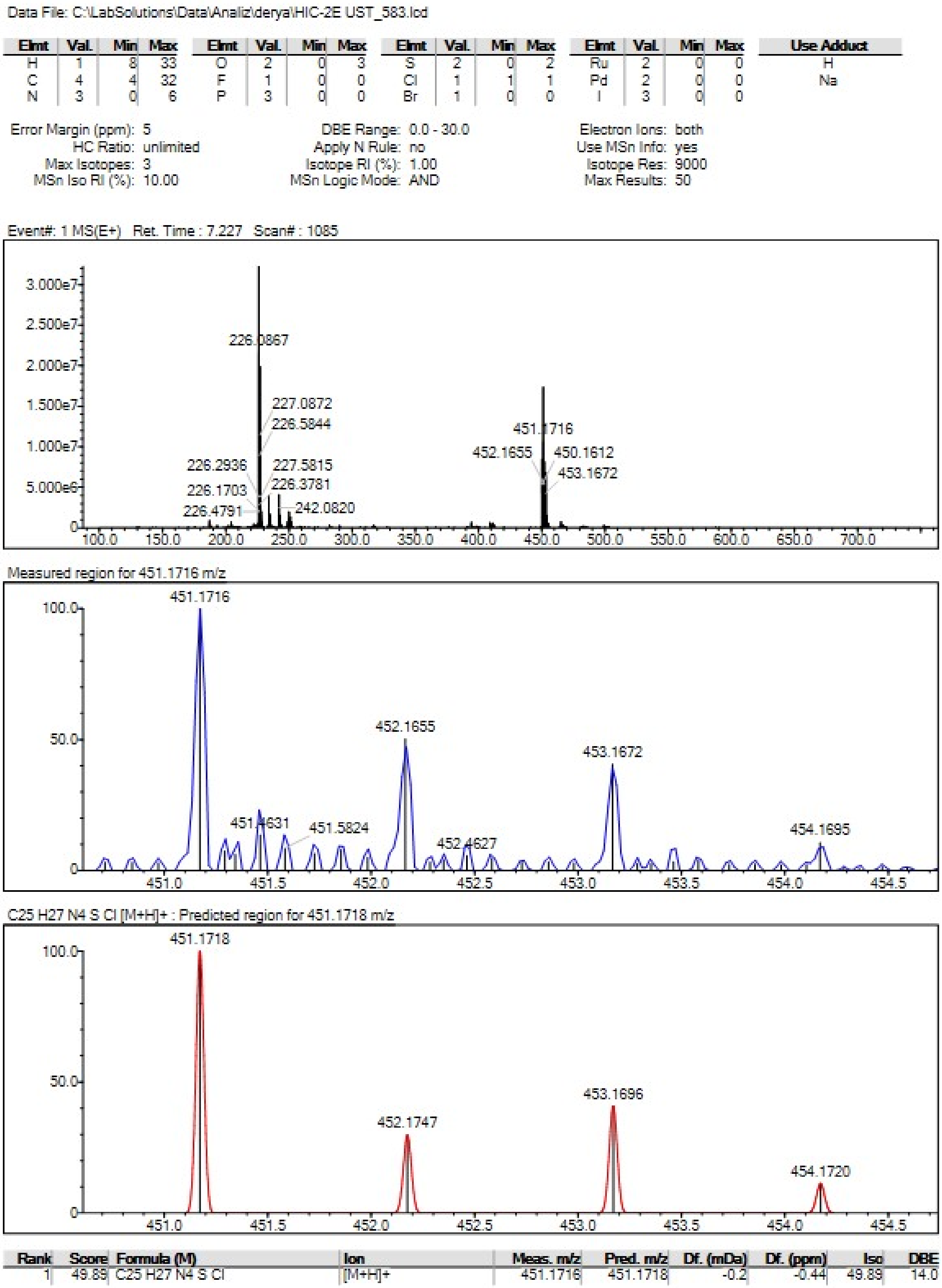

Figure 51S. HRMS spectrogram of **D2d**.F

	Item	Value
	Acquired Date&Time	3.05.2024 10:53:49
	Acquired by	System Administrator
	Filename	C:\Users\dopnalab\Desktop\MASAÜSTÜ\sazan\hic\hic-2e1.ispd
	Spectrum name	hic-2e1
	Sample name	hic-2e
	Sample ID	
	Option	
	Comment	
	No. of Scans	30
	Resolution	4 [cm-1]
	Apodization	Happ-Genzel

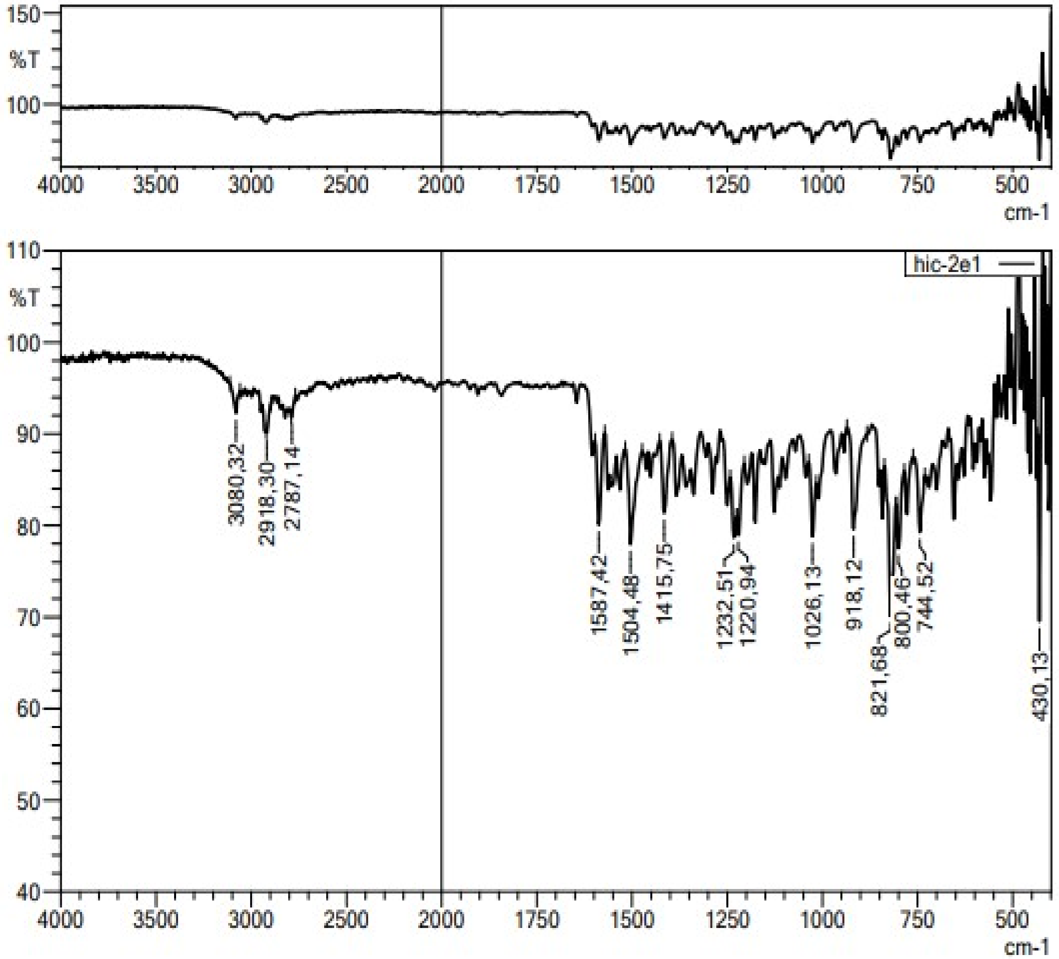

Figure 52S. IR fingerprint of **D2e**.

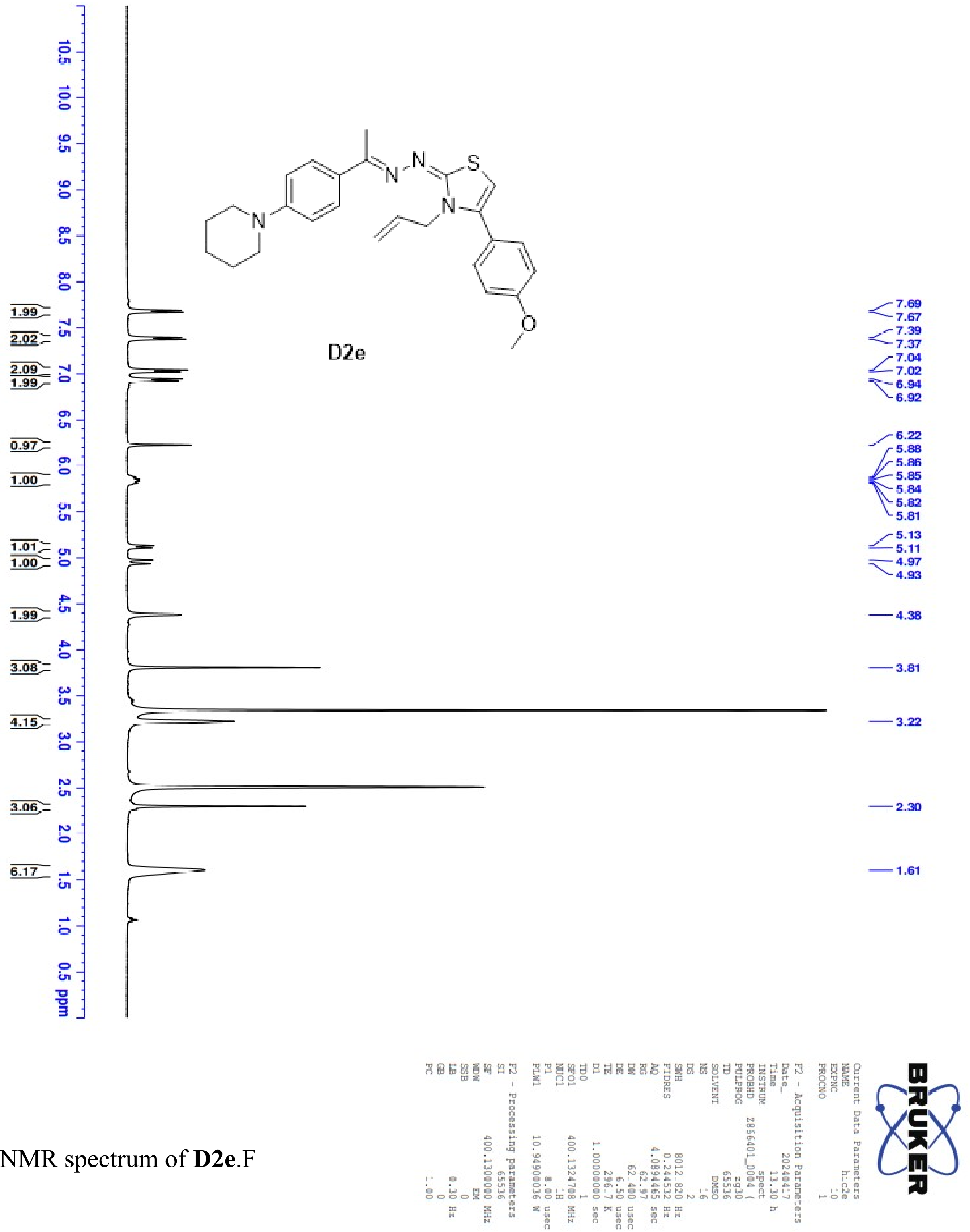

Figure 53S.^{1}HNMR spectrum of **D2e**.F

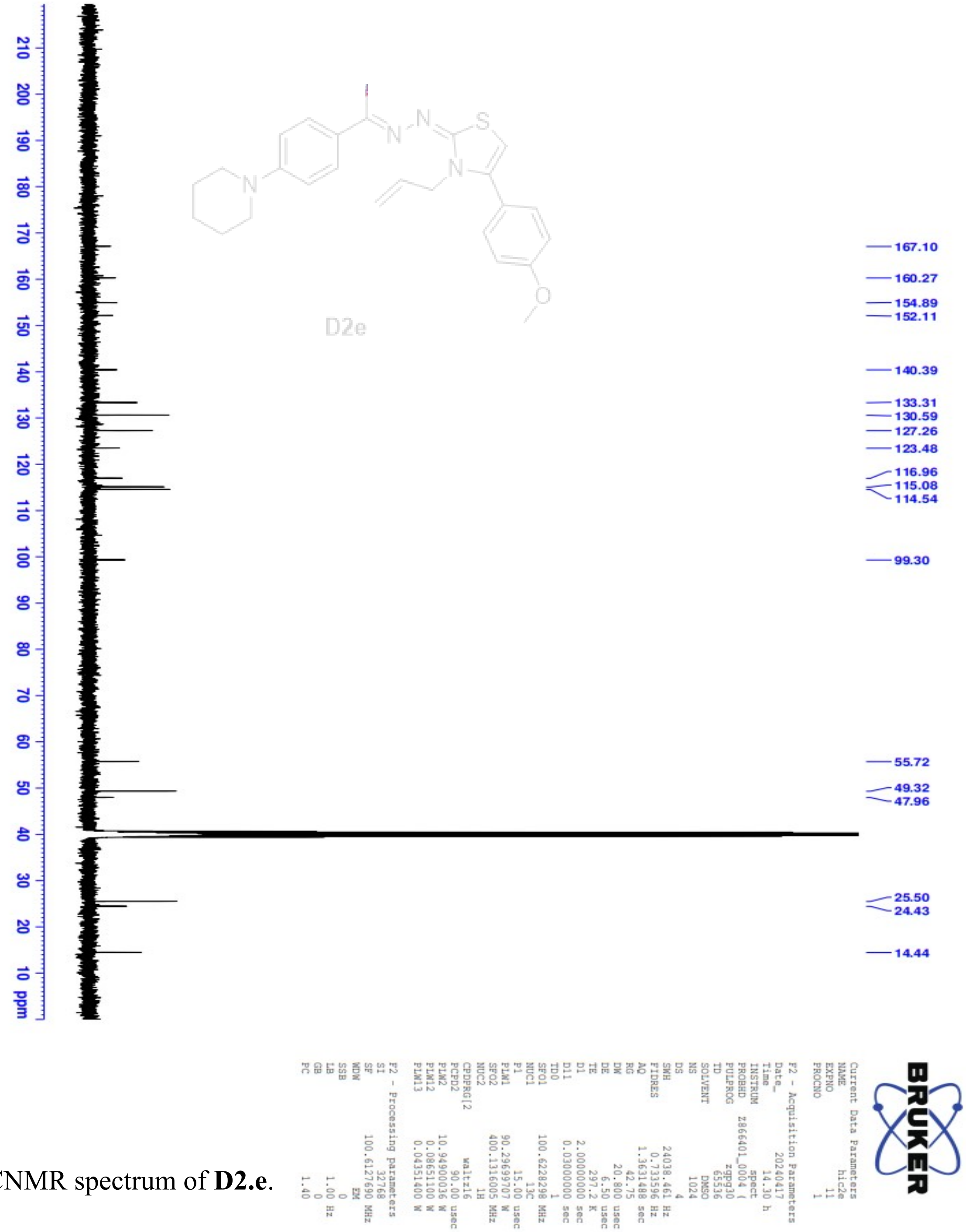

Figure 54S.^{13}CNMR spectrum of **D2.e**.

Data File: C:\LabSolutions\Data\Analiz\derya\HIC-2F UST_585.lcd

Elmt	Val.	Min	Max	Elmt	Val.	Min	Max	Elmt	Val.	Min	Max	Elmt	Val.	Min	Max	Use Adduct
H	1	8	33	O	2	0	3	S	2	0	2	Ru	2	0	0	H
C	4	4	32	F	1	0	0	Cl	1	0	0	Pd	2	0	0	Na
N	3	0	6	P	3	0	0	Br	1	0	0	I	3	0	0	

Error Margin (ppm): 5
HC Ratio: unlimited
Max Isotopes: 3
MSn Iso RI (%): 10.00

DBE Range: 0.0 - 30.0
Apply N Rule: no
Isotope RI (%): 1.00
MSn Logic Mode: AND

Electron Ions: both
Use MSn Info: yes
Isotope Res: 9000
Max Results: 50

Event#: 1 MS(E+) Ret. Time : 6.720 - 0.387 -> 4.039 Scan# : 1009 - 59 -> 607

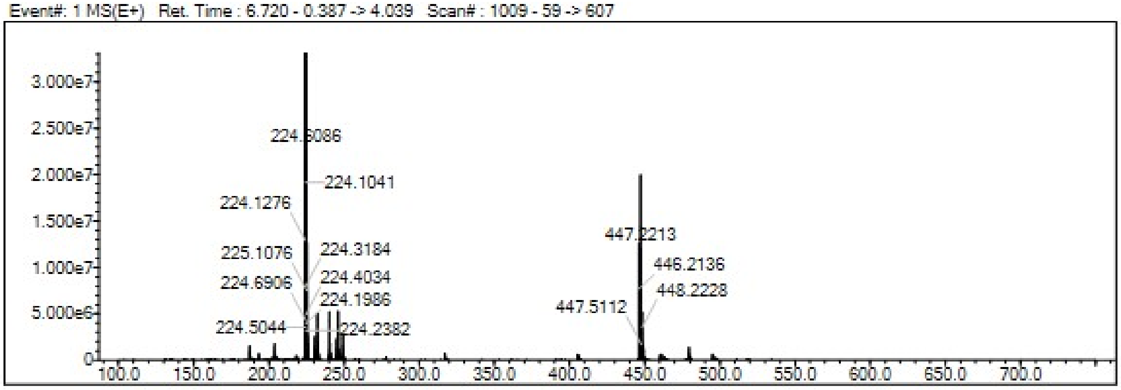

Measured region for 447.2213 m/z

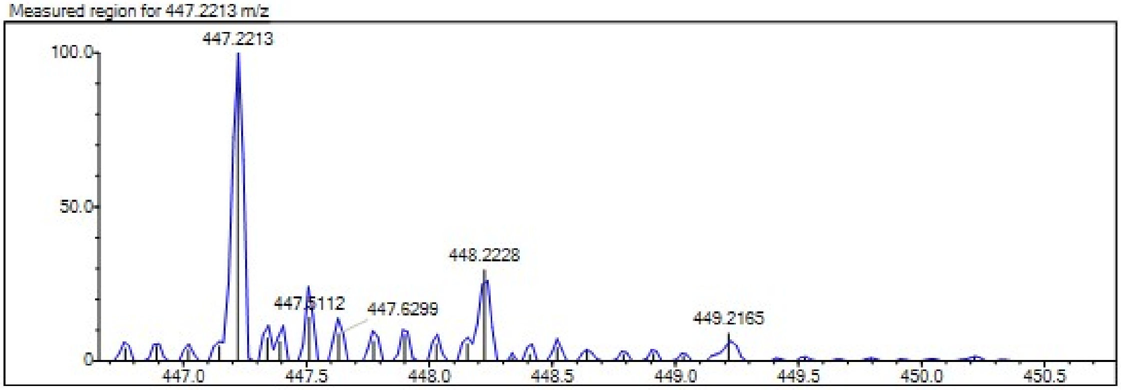

C26 H30 N4 O S [M+H]+ : Predicted region for 447.2213 m/z

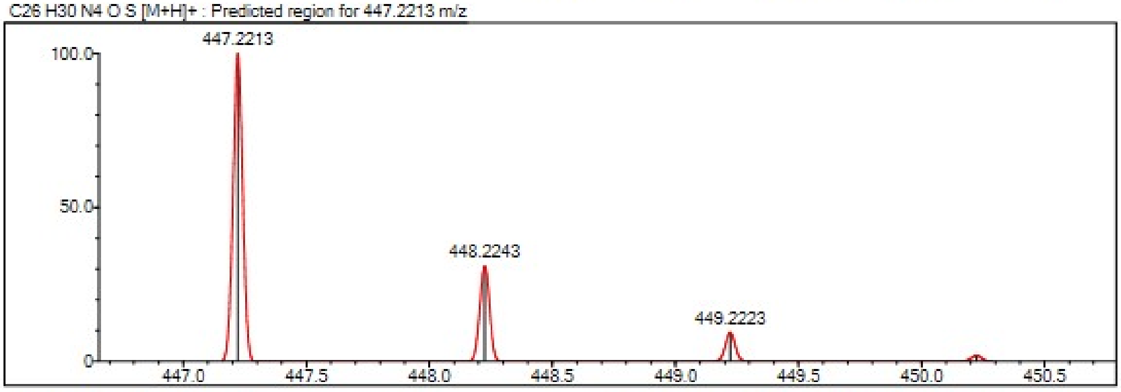

Rank	Score	Formula (M)	Ion	Meas. m/z	Pred. m/z	Df. (mDa)	Df. (ppm)	Iso	DBE
1	67.97	C26 H30 N4 O S	[M+H]+	447.2213	447.2213	-0.0	0.00	67.97	14.0

Figure 55S. HRMS spectrogram of **D2e**.

Item	Value
Acquired Date&Time	3.05.2024 10:57:59
Acquired by	System Administrator
Filename	C:\Users\dopnalab\Desktop\MASAÜSTÜ\sazan\hic\hic-2f1.ispd
Spectrum name	hic-2f1
Sample name	hic-2f
Sample ID	
Option	
Comment	
No. of Scans	30
Resolution	4 [cm-1]
Apodization	Happ-Genzel

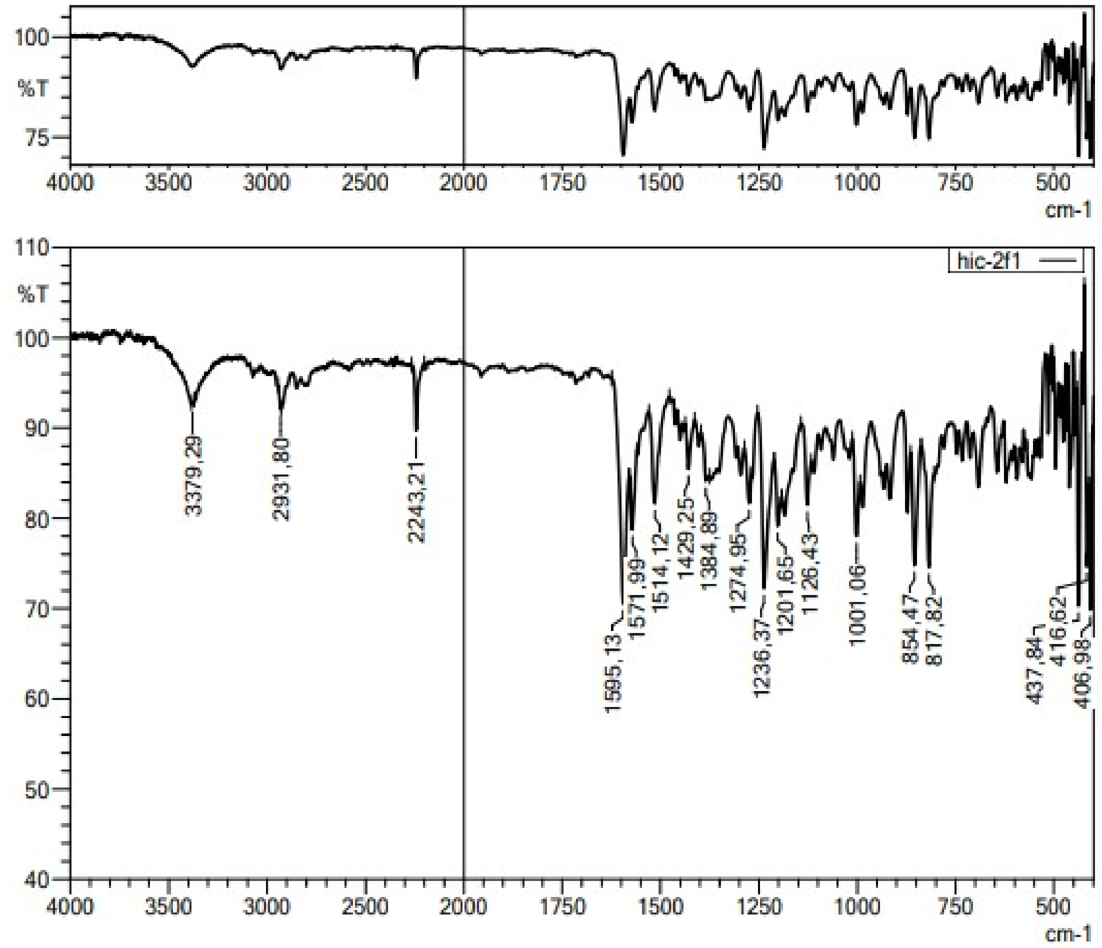

Figure 56S. IR fingerprint of **D2f**.

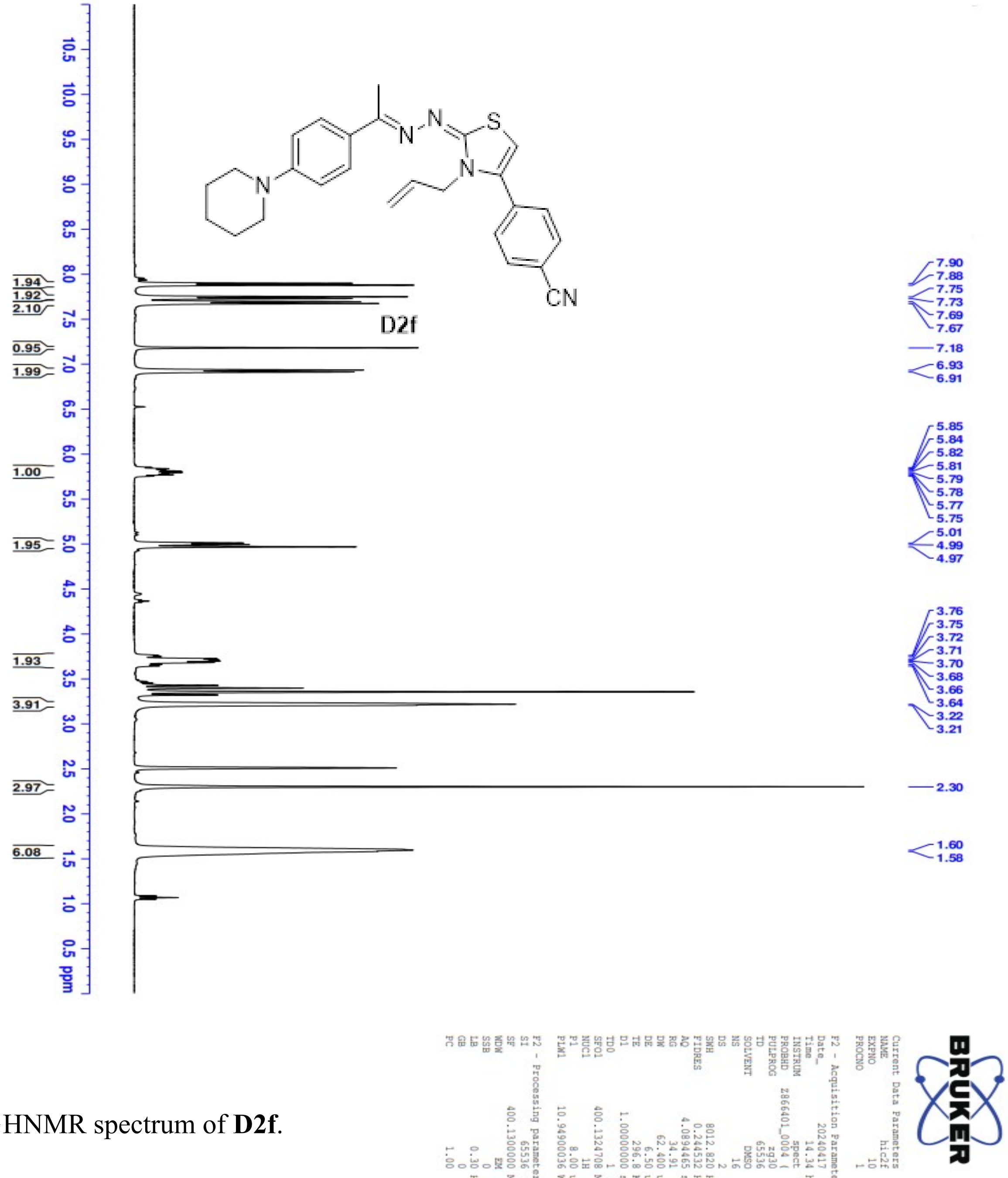

Figure 57S. ¹HNMR spectrum of **D2f**.

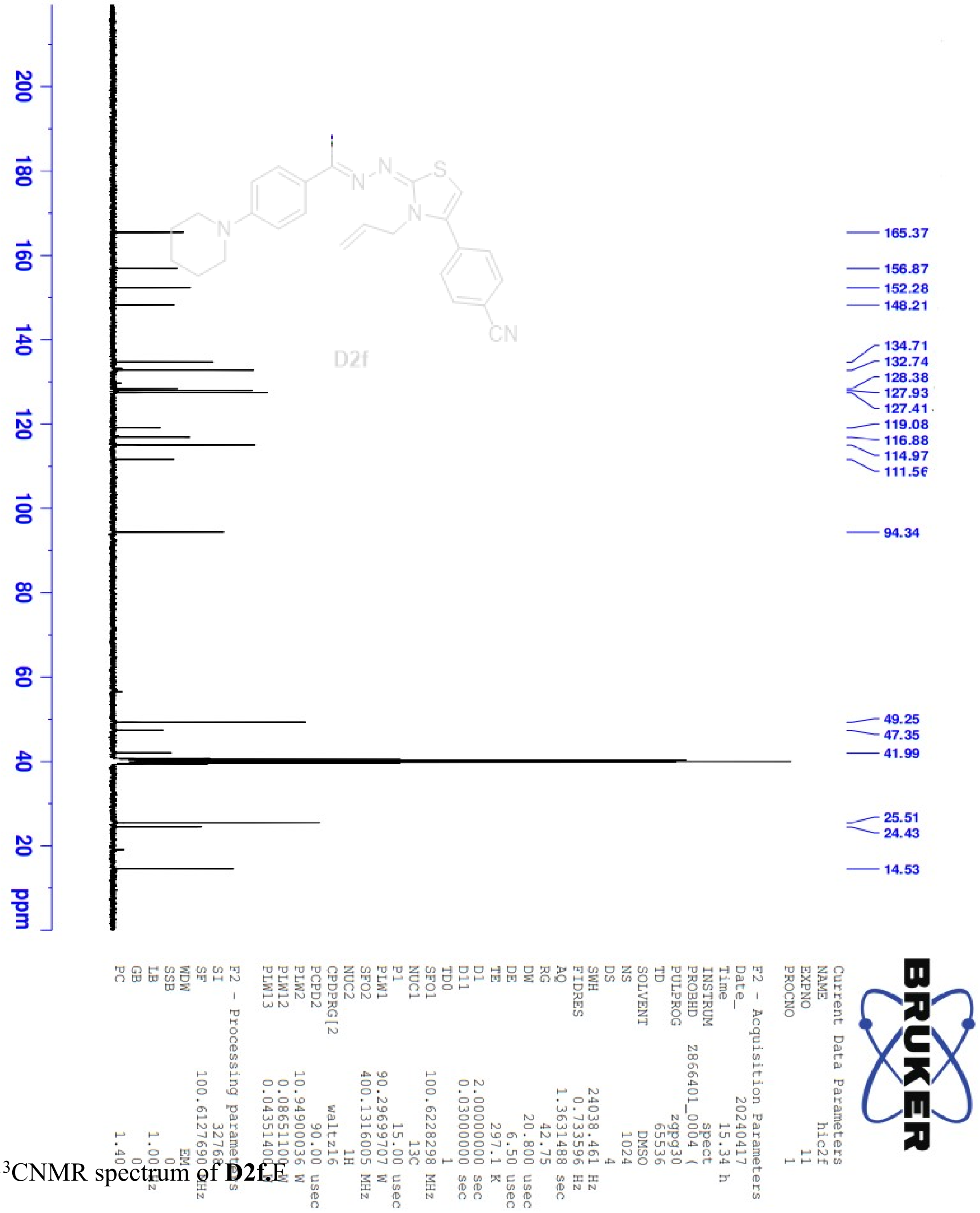

Figure 58S. ^{13}CNMR spectrum of **D2f**.F

Data File: C:\LabSolutions\Data\Analiz\derya\HIC-2I ALT_587.lcd

Elmt	Val.	Min	Max	Elmt	Val.	Min	Max	Elmt	Val.	Min	Max	Elmt	Val.	Min	Max	Use Adduct
H	1	8	33	O	2	0	3	S	2	0	2	Ru	2	0	0	H
C	4	4	32	F	1	0	0	Cl	1	0	0	Pd	2	0	0	Na
N	3	0	6	P	3	0	0	Br	1	0	0	I	3	0	0	

Error Margin (ppm): 5
HC Ratio: unlimited
Max Isotopes: 3
MSn Iso RI (%): 10.00

DBE Range: 0.0 - 30.0
Apply N Rule: no
Isotope RI (%): 1.00
MSn Logic Mode: AND

Electron Ions: both
Use MSn Info: yes
Isotope Res: 9000
Max Results: 50

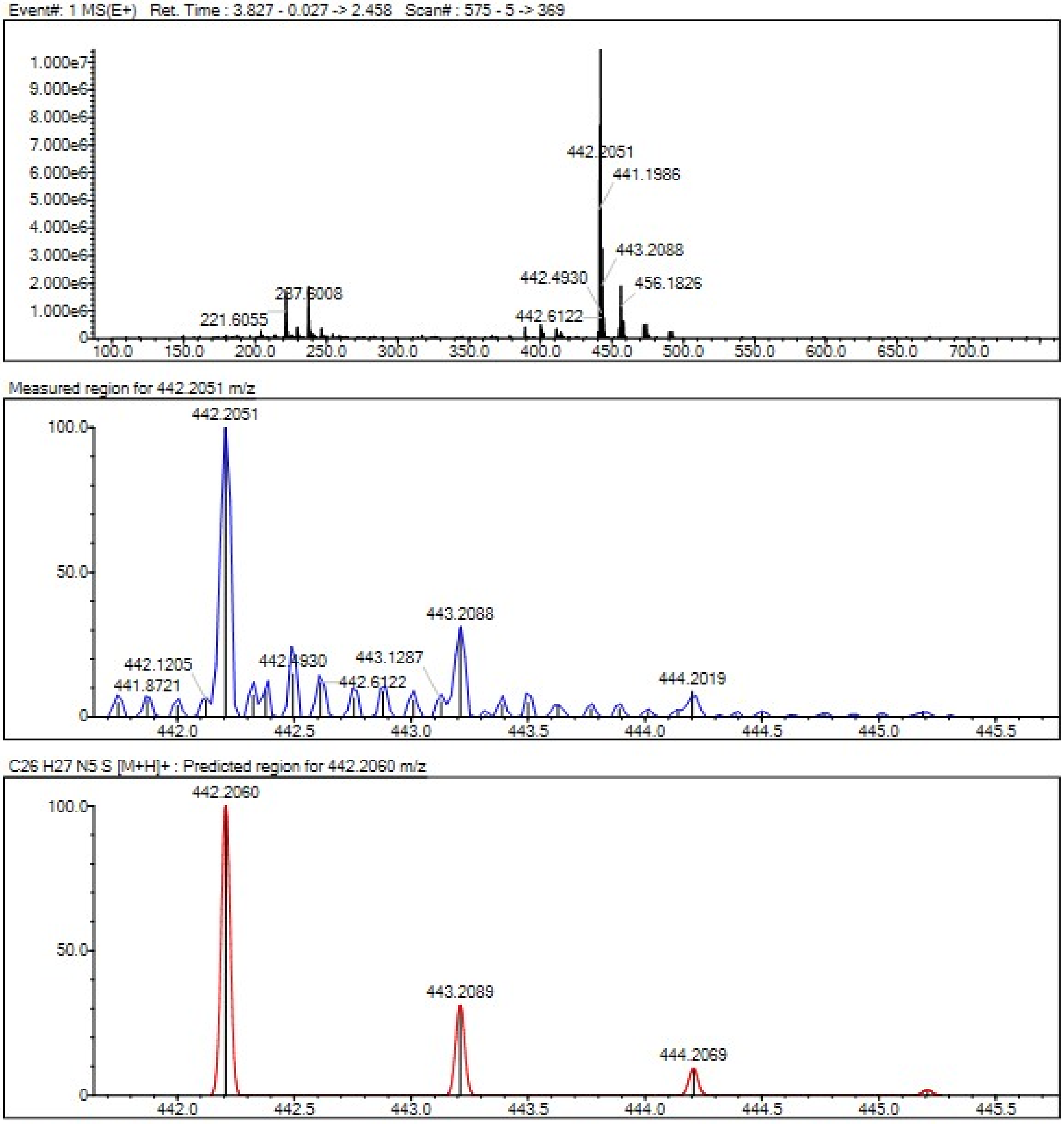

Rank	Score	Formula (M)	Ion	Meas. m/z	Pred. m/z	Df. (mDa)	Df. (ppm)	Iso	DBE
3	73.73	C26 H27 N5 S	[M+H]+	442.2051	442.2060	-0.9	-2.04	75.70	16.0

Figure 59S. HRMS spectrogram of **D2f.**F

Item	Value
Acquired Date&Time	3.05.2024 11:05:55
Acquired by	System Administrator
Filename	C:\Users\dopnalab\Desktop\MASAÜSTÜ\sazan\hic\hic-2h1.ispd
Spectrum name	hic-2h1
Sample name	hic-2h
Sample ID	
Option	
Comment	
No. of Scans	30
Resolution	4 [cm-1]
Apodization	Happ-Genzel

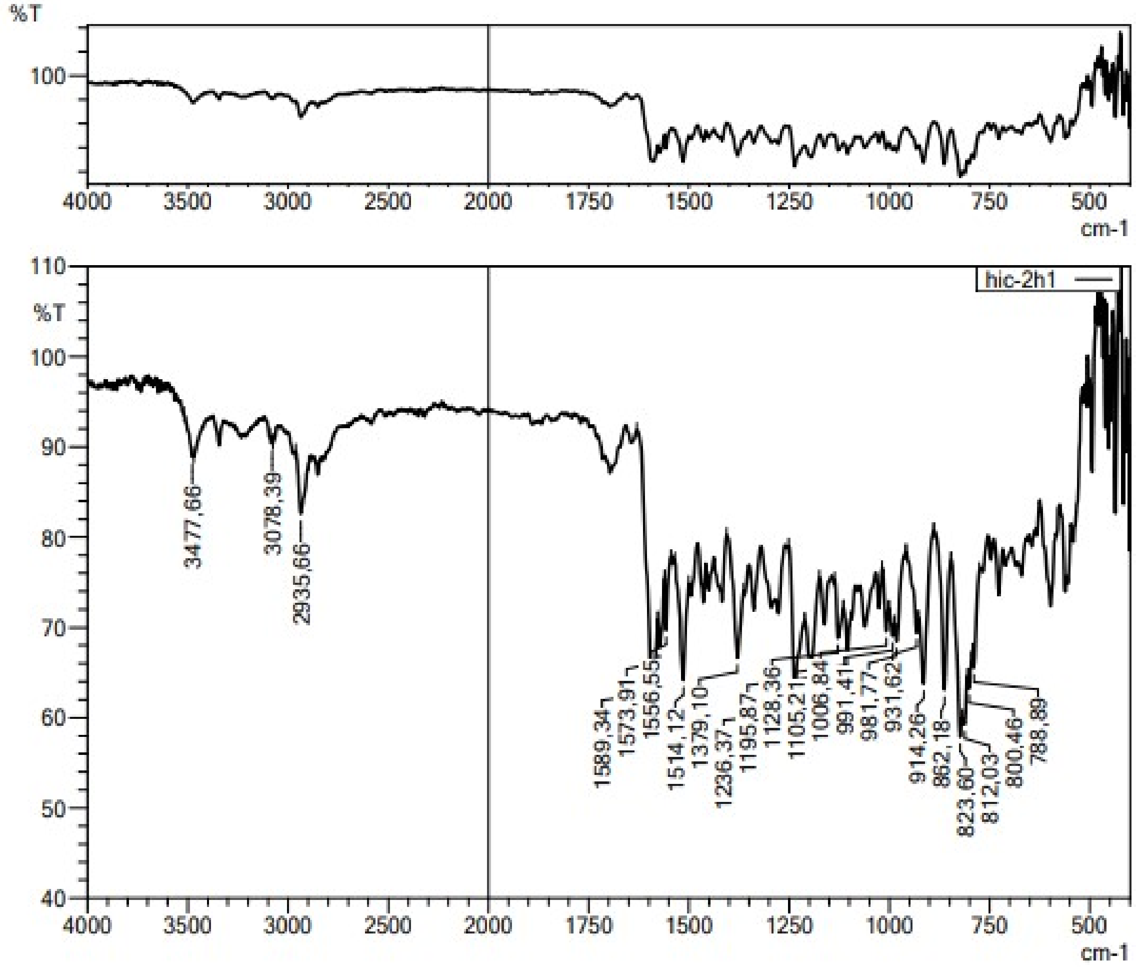

Figure 60S. IR fingerprint of **D2g**.F

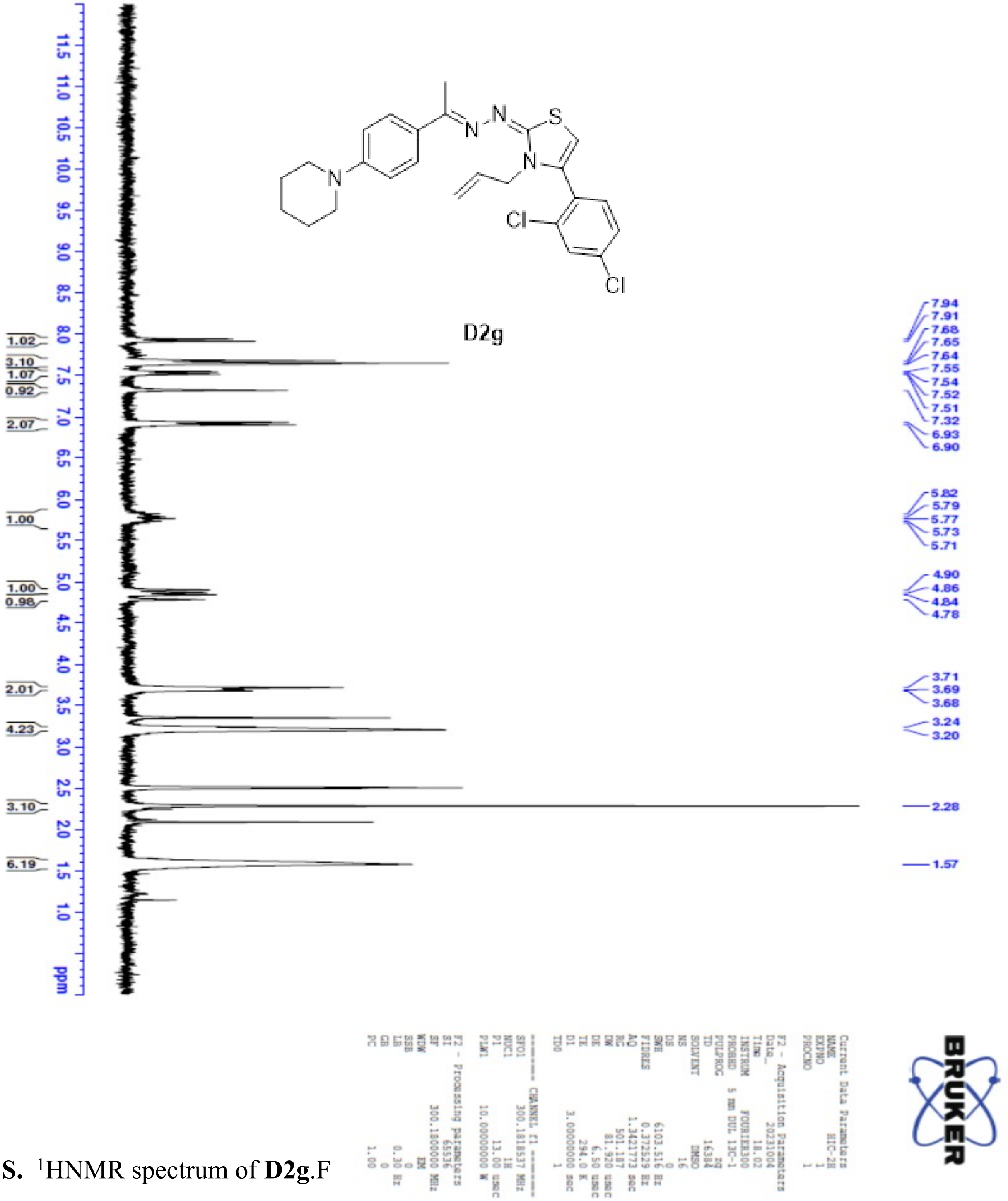

Figure 61S. ^{1}HNMR spectrum of **D2g**.F

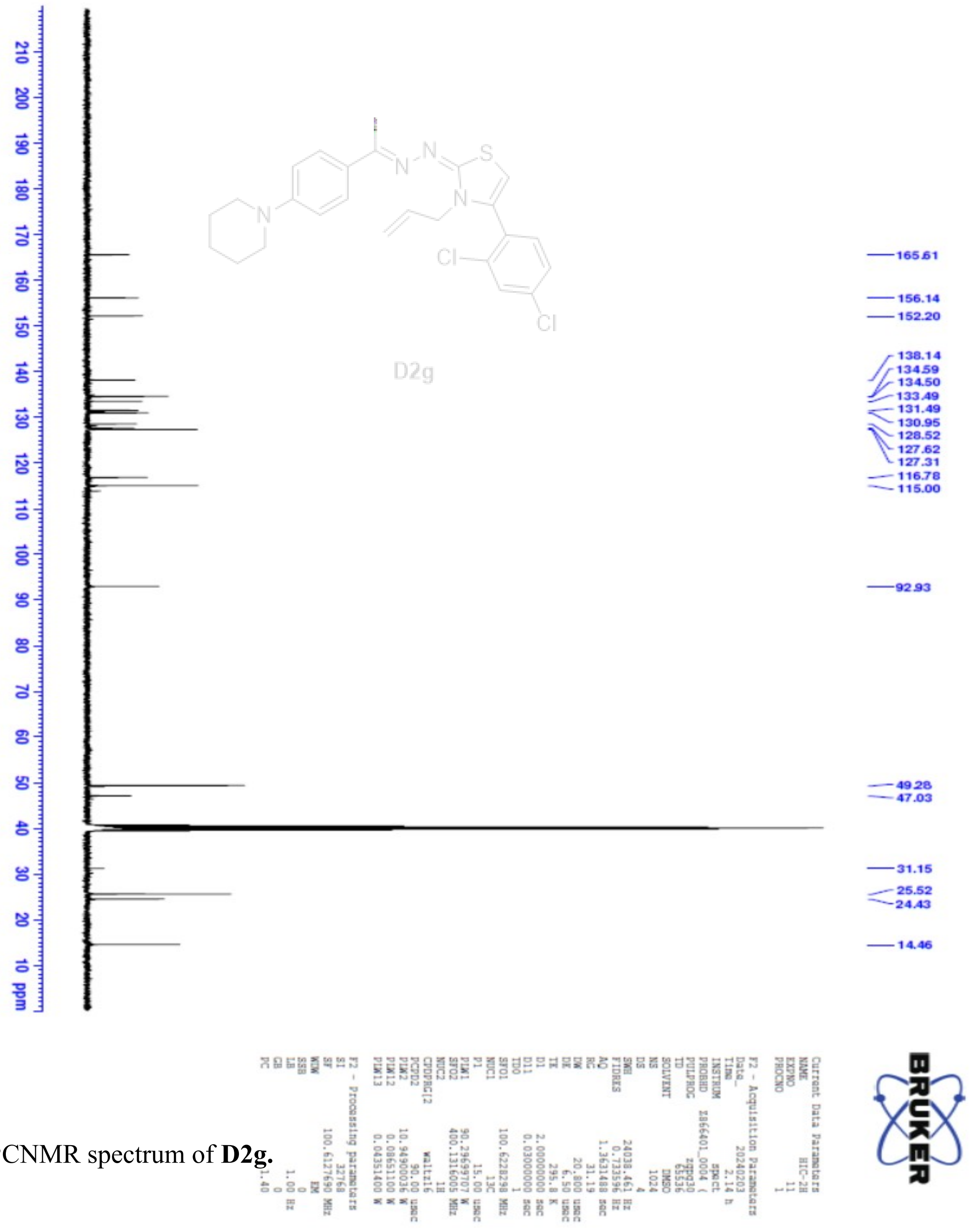

Figure 62S. ^{13}CNMR spectrum of **D2g.**

Item	Value
Acquired Date&Time	3.05.2024 11:10:46
Acquired by	System Administrator
Filename	C:\Users\dopnalab\Desktop\MASAÜSTÜ\sazan\hic\hic-2i1.ispd
Spectrum name	hic-2i1
Sample name	hic-2i
Sample ID	
Option	
Comment	
No. of Scans	30
Resolution	4 [cm-1]
Apodization	Happ-Genzel

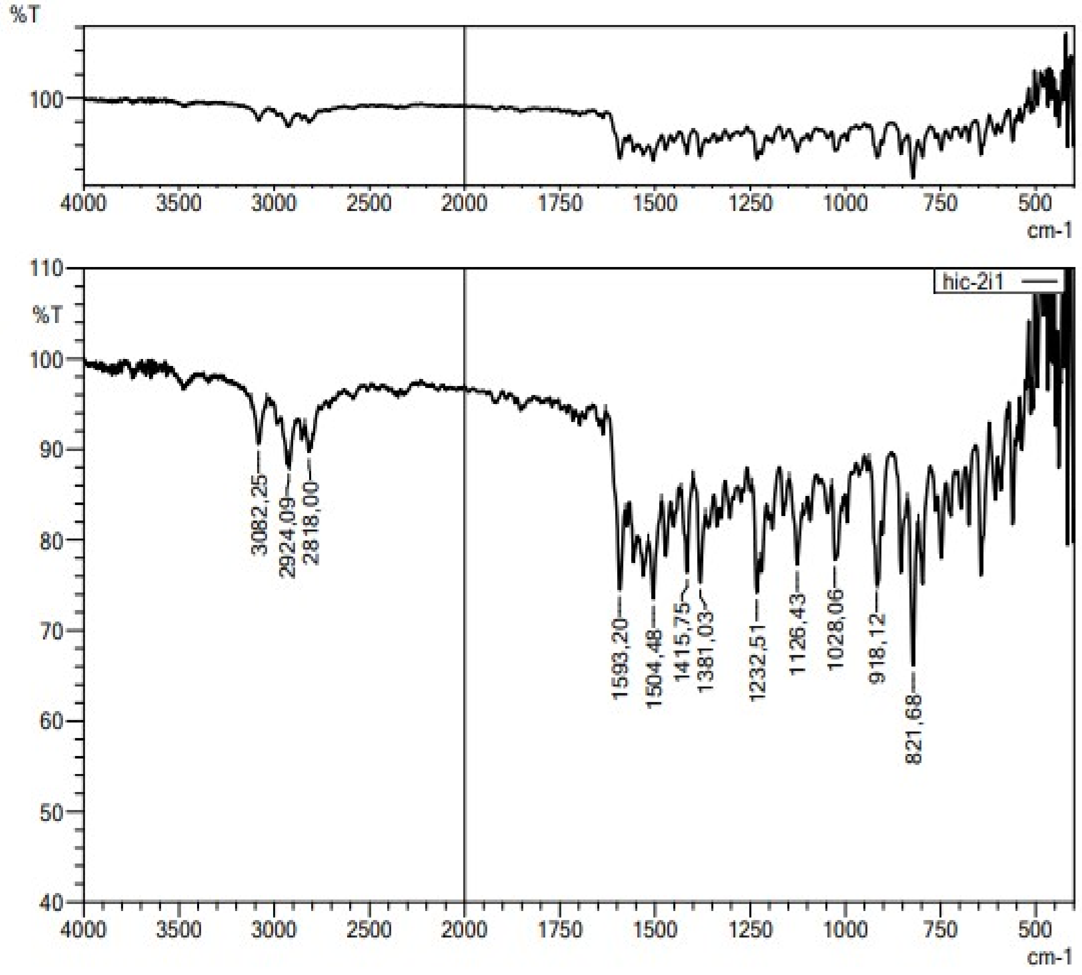

Figure 63S. IR fingerprint of **D2h**.F

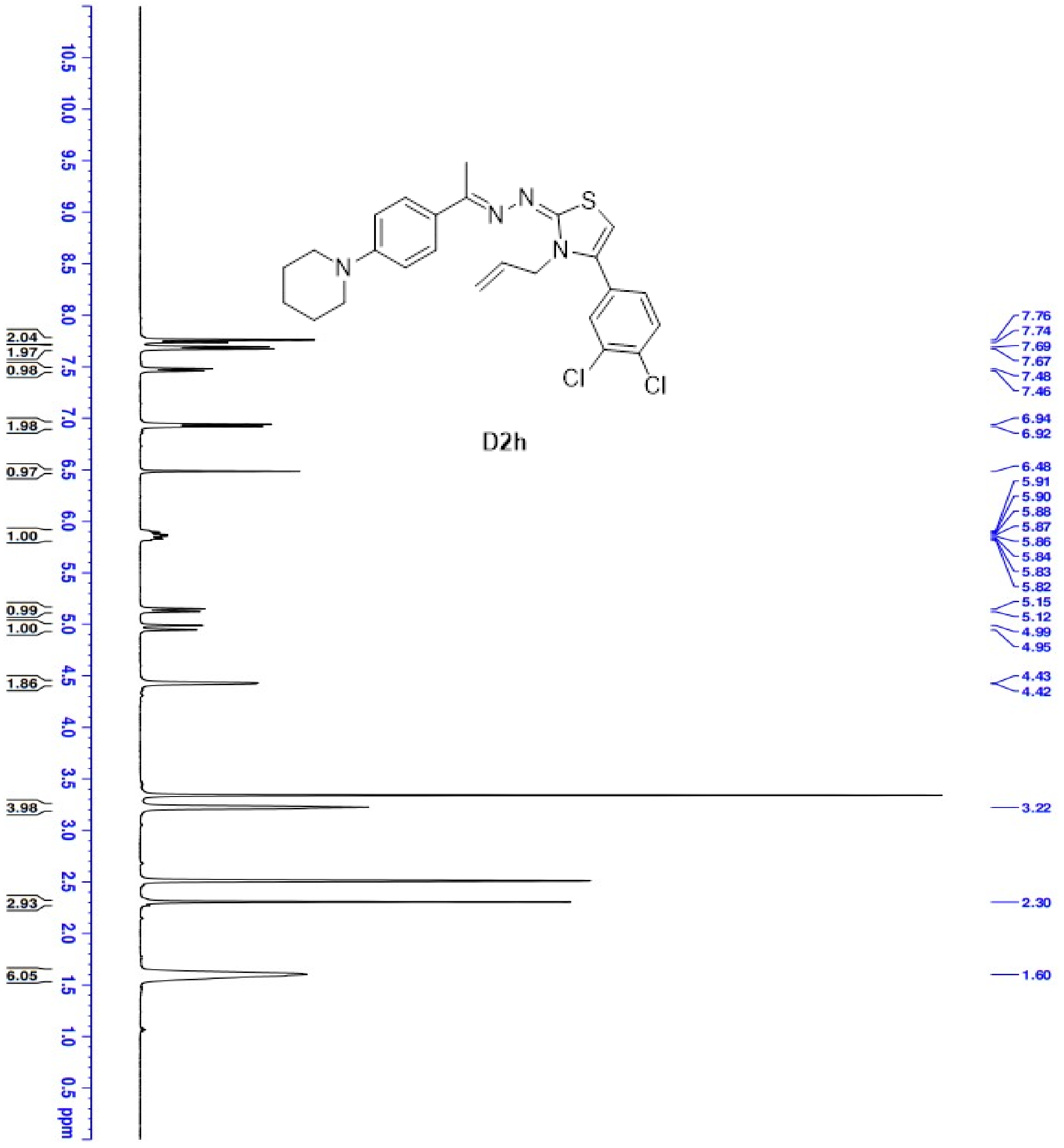

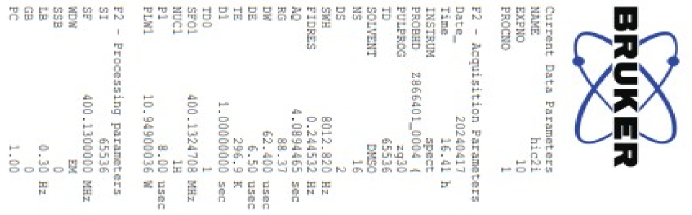

Figure 64S. ^{1}HNMR spectrum of **D2h**. F

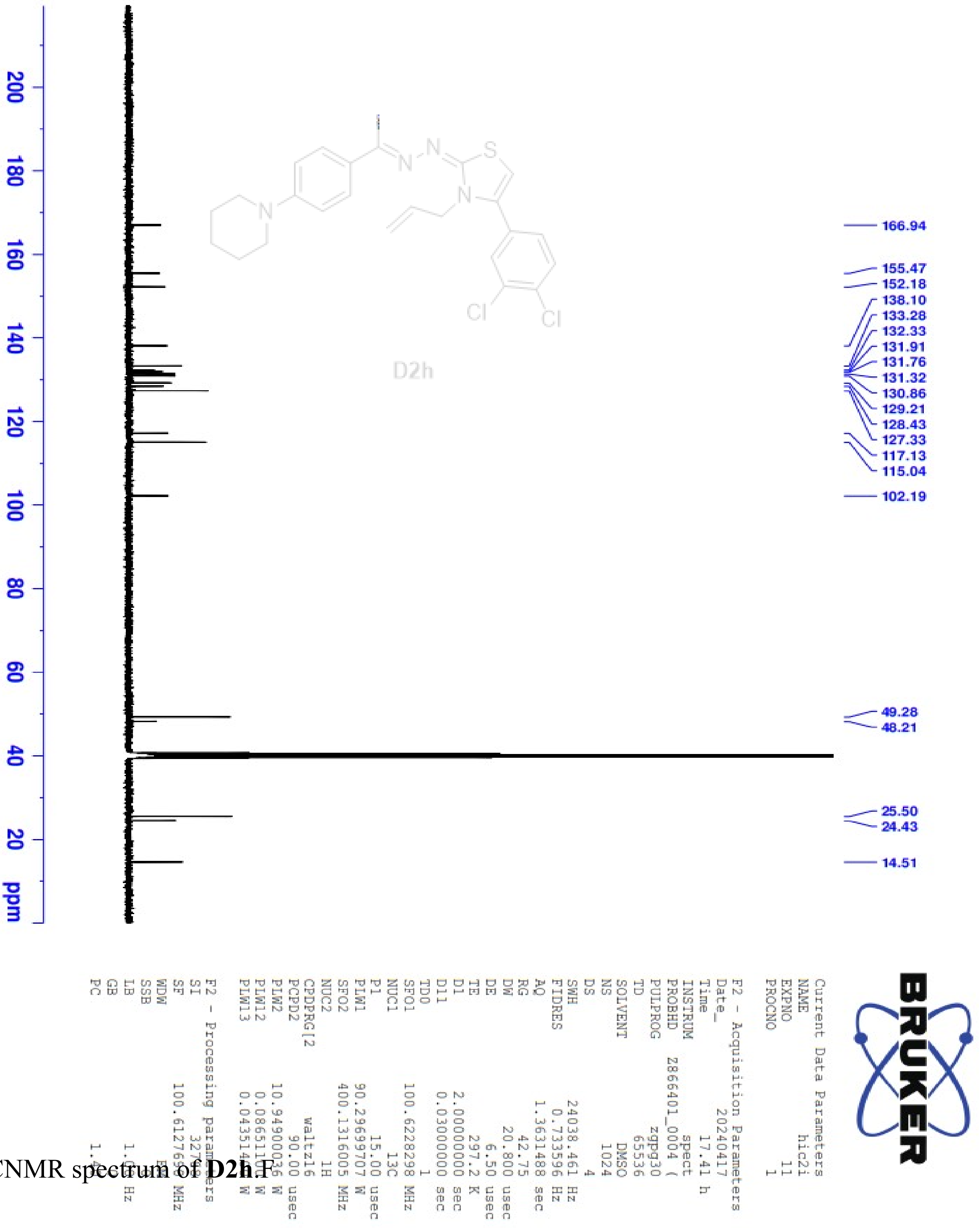

Figure 65S. ^{13}CNMR spectrum of **D2h**.F

Data File: C:\LabSolutions\Data\Analiz\derya\MEOH_589.lcd

Elmt	Val.	Min	Max	Elmt	Val.	Min	Max	Elmt	Val.	Min	Max	Elmt	Val.	Min	Max	Use Adduct
H	1	8	33	O	2	0	3	S	2	1	1	Ru	2	0	0	H
C	4	4	32	F	1	0	0	Cl	1	2	2	Pd	2	0	0	Na
N	3	0	4	P	3	0	0	Br	1	0	0	I	3	0	0	

Error Margin (ppm): 5
HC Ratio: unlimited
Max Isotopes: 3
MSn Iso RI (%): 10.00

DBE Range: 0.0 - 30.0
Apply N Rule: no
Isotope RI (%): 1.00
MSn Logic Mode: AND

Electron Ions: both
Use MSn Info: yes
Isotope Res: 9000
Max Results: 50

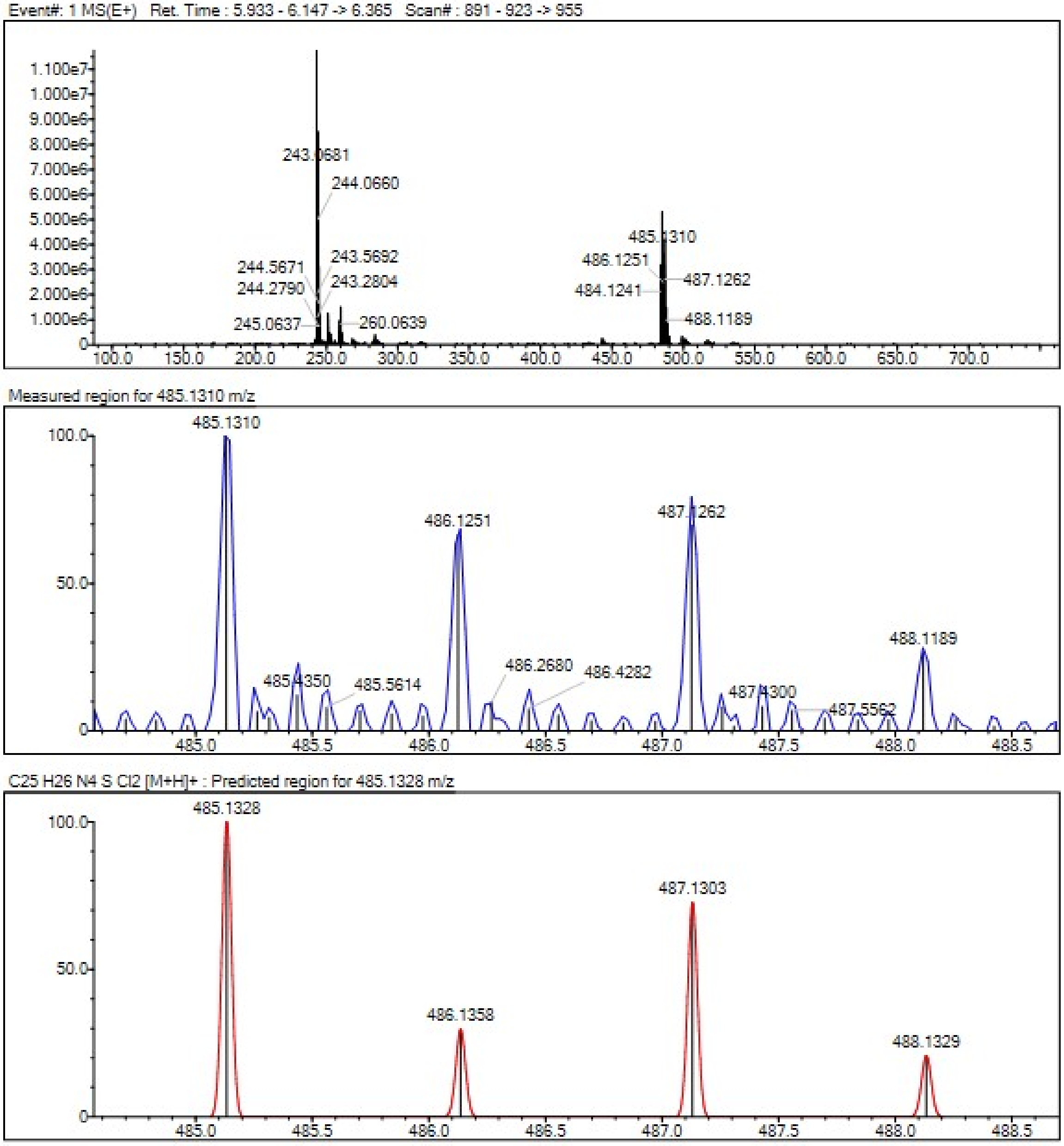

Rank	Score	Formula (M)	Ion	Meas. m/z	Pred. m/z	Df. (mDa)	Df. (ppm)	Iso	DBE
4	25.69	C25 H26 N4 S Cl2	[M+H]+	485.1310	485.1328	-1.8	-3.71	27.56	14.0

Figure 66S. HRMS spectrogram of **D2h**.F

	Item	Value
	Acquired Date&Time	3.05.2024 11:15:49
	Acquired by	System Administrator
	Filename	C:\Users\dopnalab\Desktop\MASAÜSTÜ\sazan\hic\hic-2j1.ispd
	Spectrum name	hic-2j1
	Sample name	hic-2j
	Sample ID	
	Option	
	Comment	
	No. of Scans	30
	Resolution	4 [cm-1]
	Apodization	Happ-Genzel

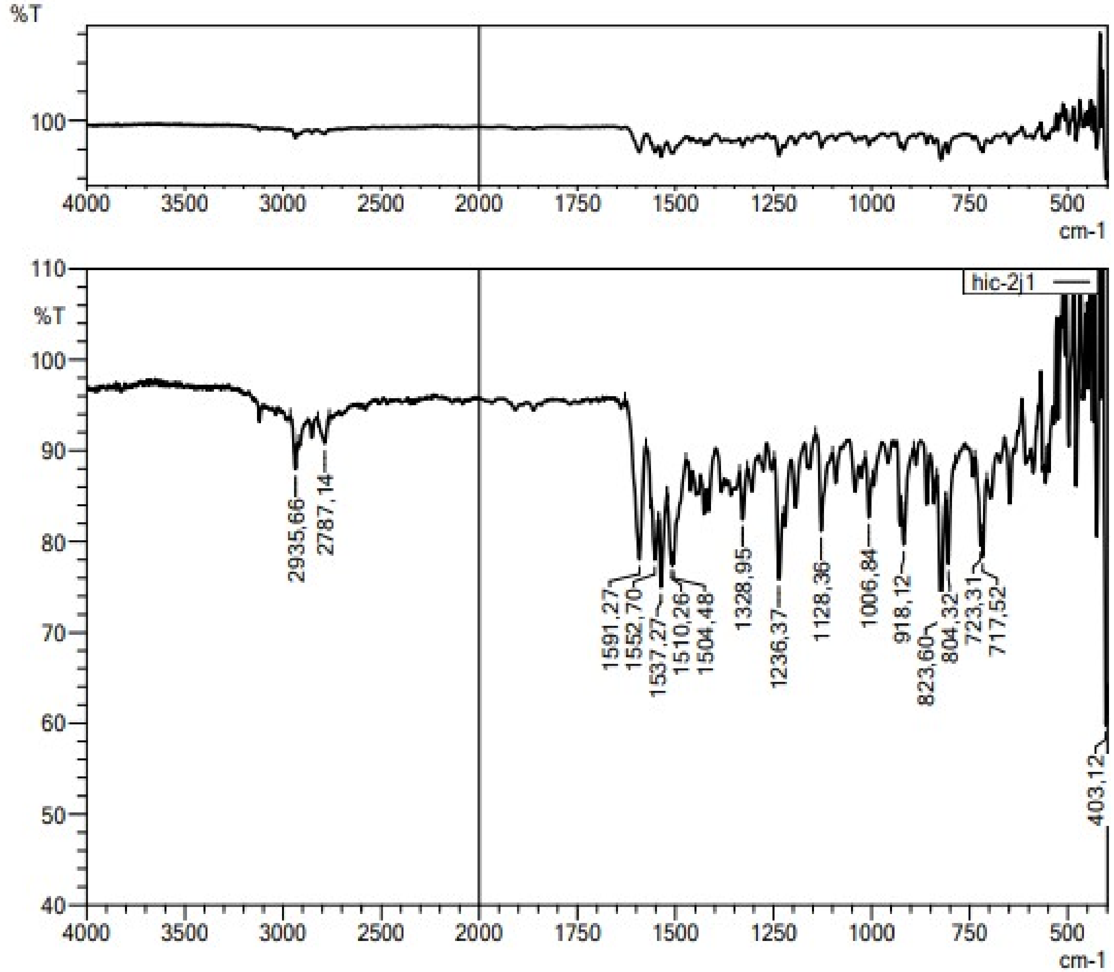

Figure 67S. IR fingerprint of **D2i**.F

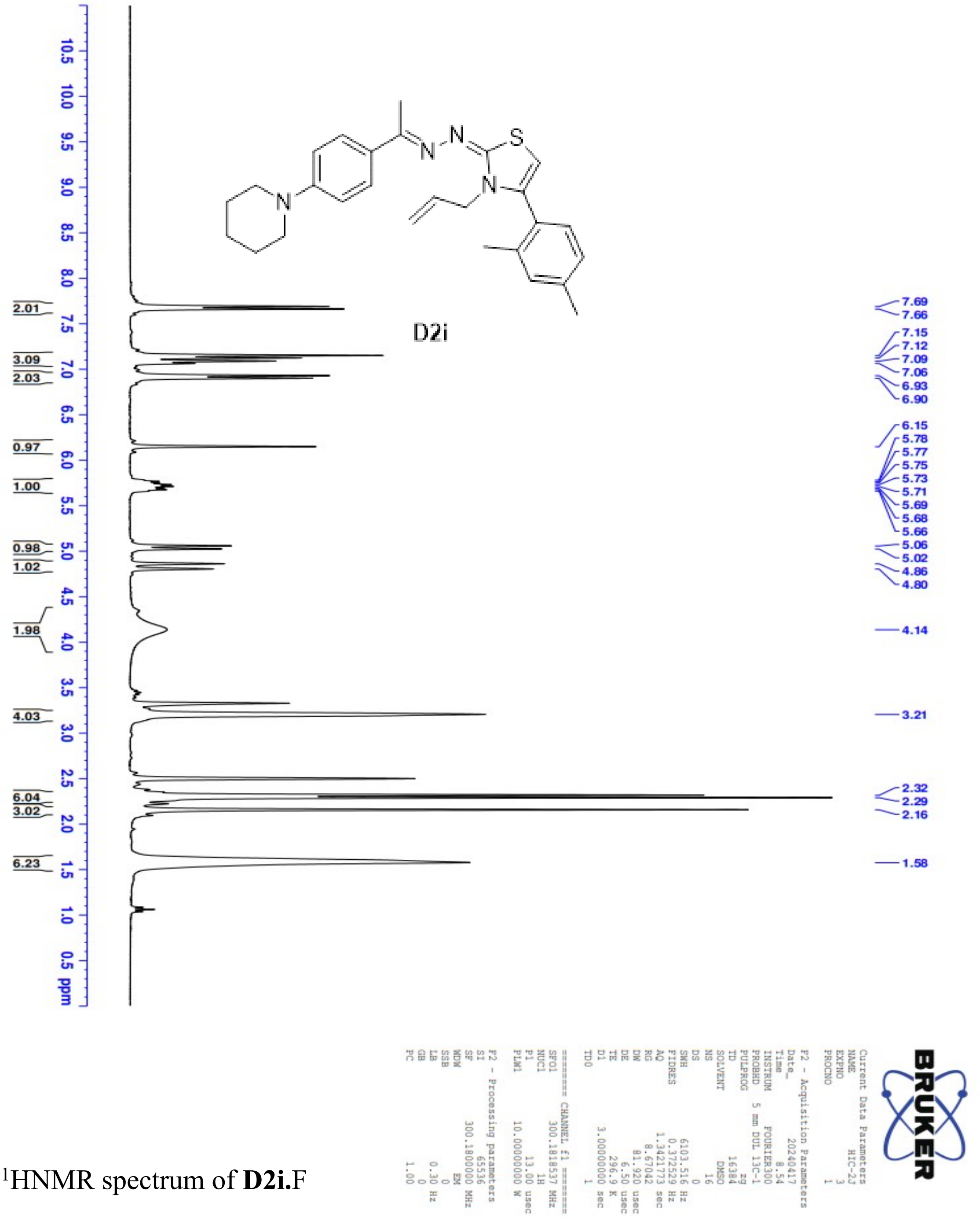

Figure 68S. ^{1}HNMR spectrum of **D2i.**F

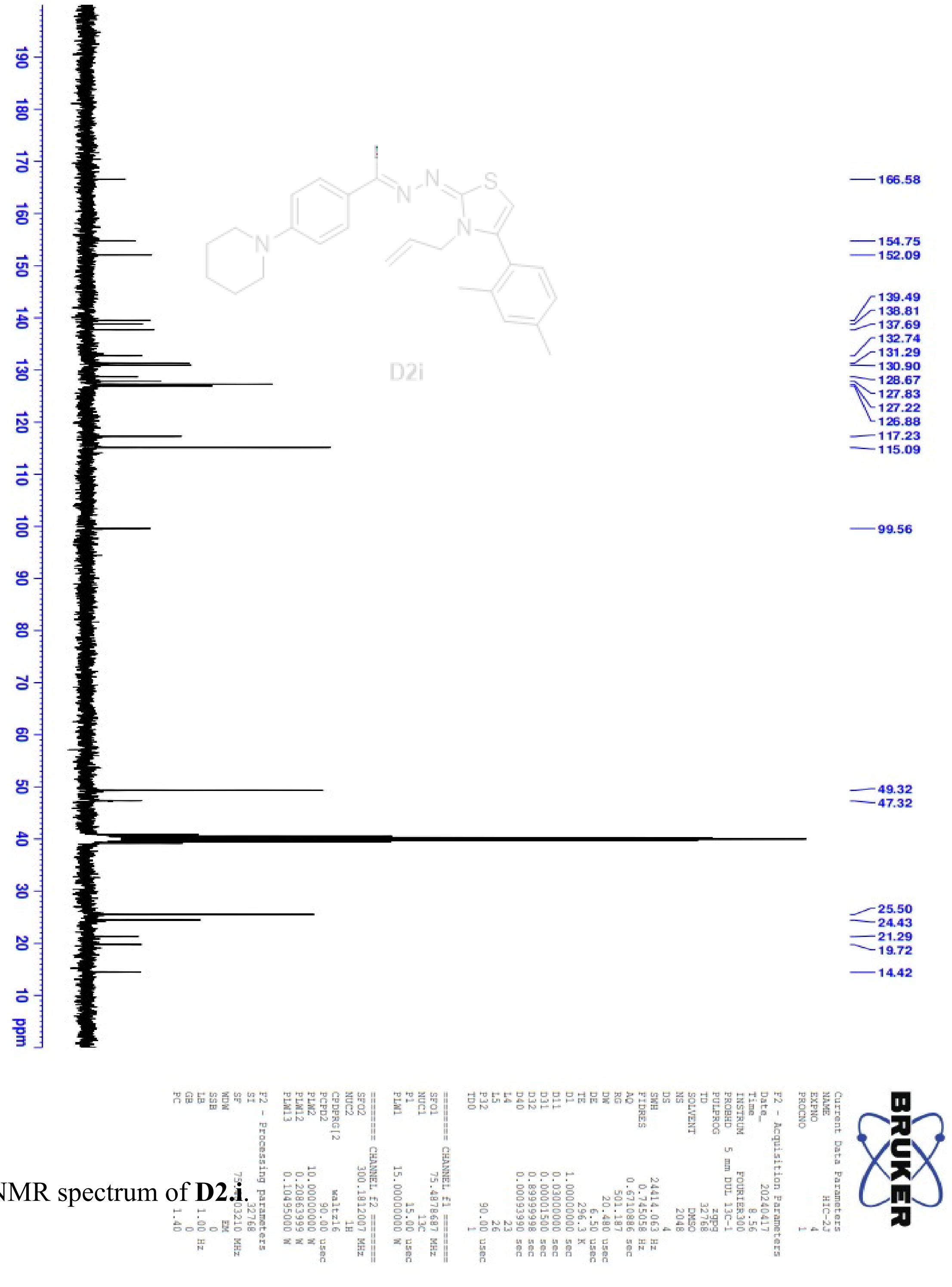

Figure 69S. ^{13}CNMR spectrum of **D2.i**.

Data File: C:\LabSolutions\Data\Analiz\derya\MEOH_589.lcd

Elmt	Val.	Min	Max	Elmt	Val.	Min	Max	Elmt	Val.	Min	Max	Elmt	Val.	Min	Max	Use Adduct
H	1	8	33	O	2	0	3	S	2	0	2	Ru	2	0	0	H
C	4	4	32	F	1	0	0	Cl	1	0	0	Pd	2	0	0	Na
N	3	0	6	P	3	0	0	Br	1	0	0	I	3	0	0	

Error Margin (ppm):	5	DBE Range:	0.0 - 30.0	Electron Ions:	both
HC Ratio:	unlimited	Apply N Rule:	no	Use MSn Info:	yes
Max Isotopes:	3	Isotope RI (%):	1.00	Isotope Res:	9000
MSn Iso RI (%):	10.00	MSn Logic Mode:	AND	Max Results:	50

Event#: 1 MS(E+) Ret. Time : 6.240 Scan# : 937

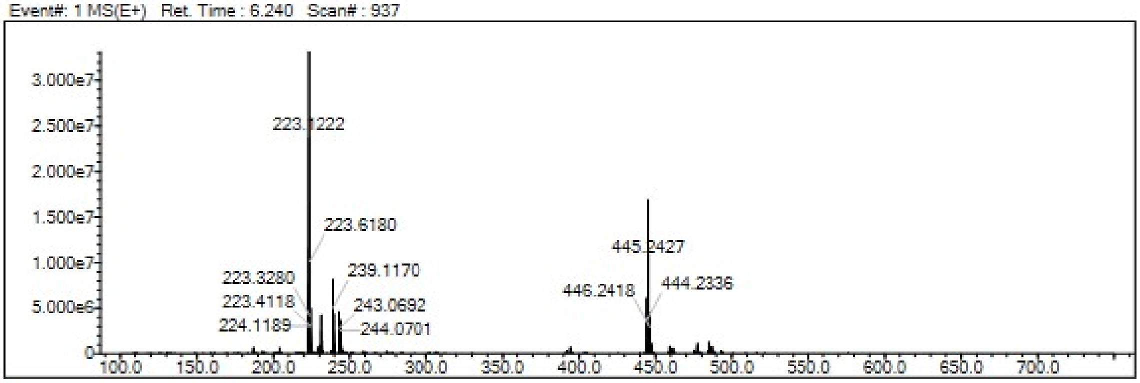

Measured region for 445.2427 m/z

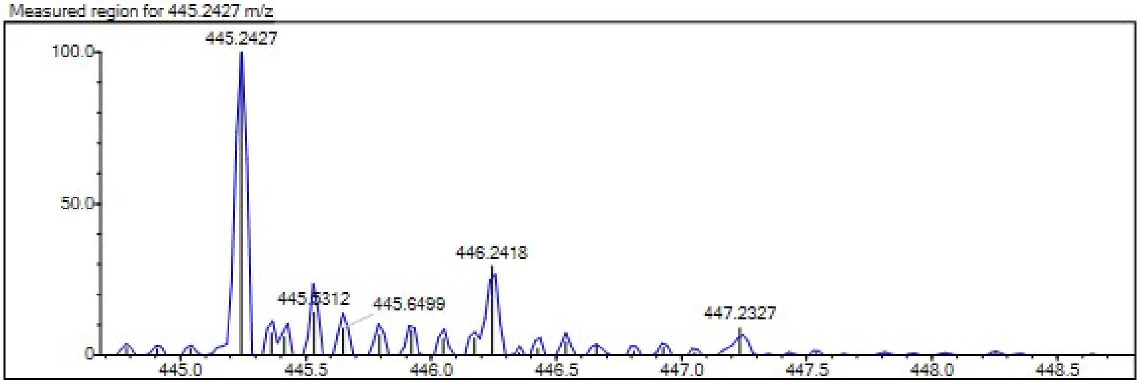

C27 H32 N4 S [M+H]+ : Predicted region for 445.2420 m/z

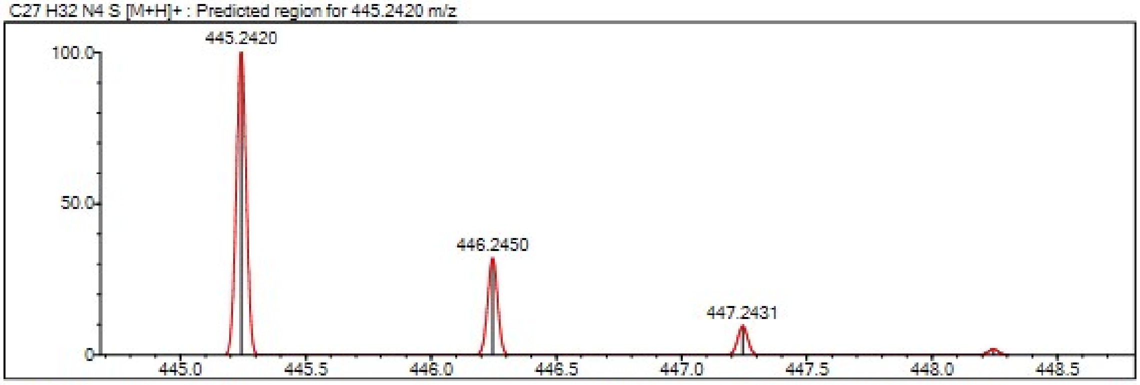

Rank	Score	Formula (M)	Ion	Meas. m/z	Pred. m/z	Df. (mDa)	Df. (ppm)	Iso	DBE
2	61.39	C27 H32 N4 S	[M+H]+	445.2427	445.2420	0.7	1.57	62.28	14.0

Figure 70S. HRMS spectrogram of **D2i**.

	Item	Value
	Acquired Date&Time	3.05.2024 11:19:45
	Acquired by	System Administrator
	Filename	C:\Users\dopnalab\Desktop\MASAÜSTÜ\sazan\hic\hic-3a1.ispd
	Spectrum name	hic-3a1
	Sample name	hic-3a
	Sample ID	
	Option	
	Comment	
	No. of Scans	30
	Resolution	4 [cm-1]
	Apodization	Happ-Genzel

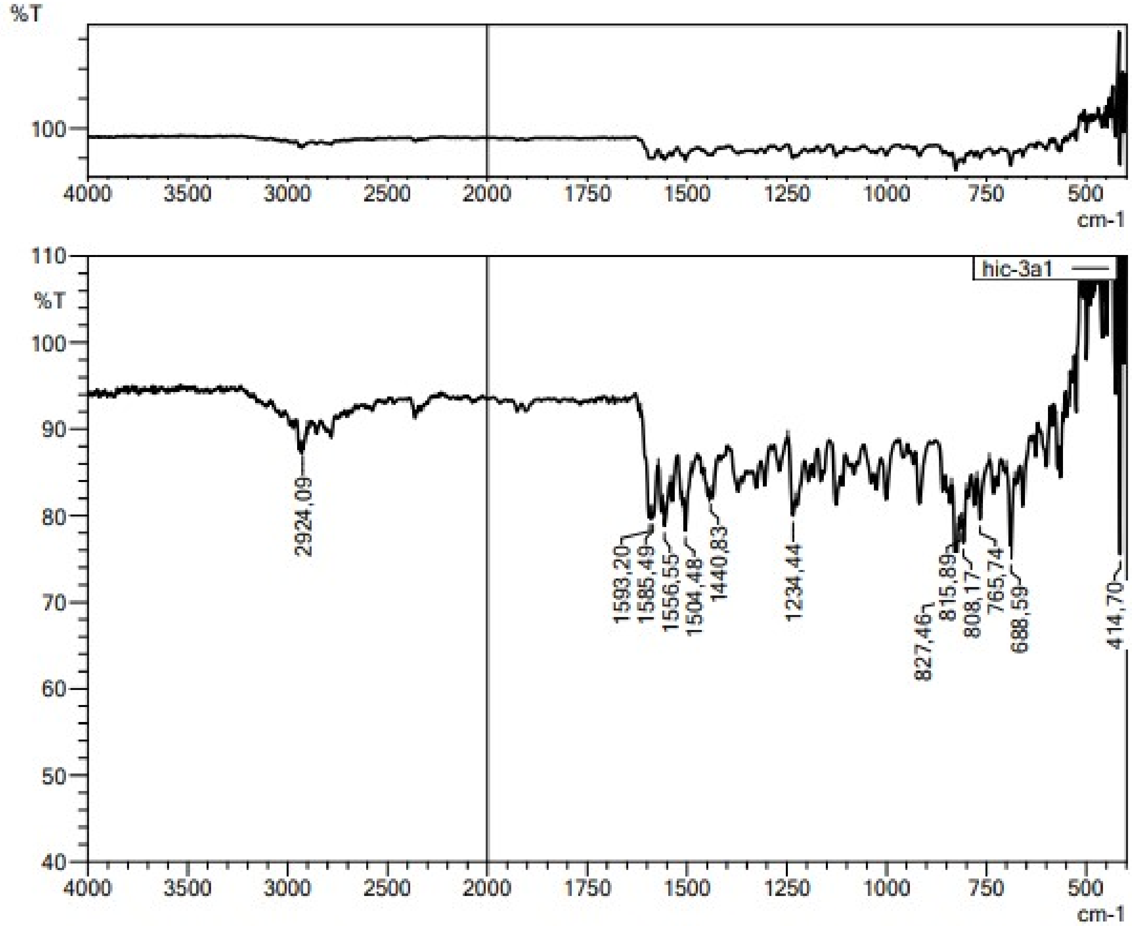

Figure 71S. IR fingerprint of **D3a**.F

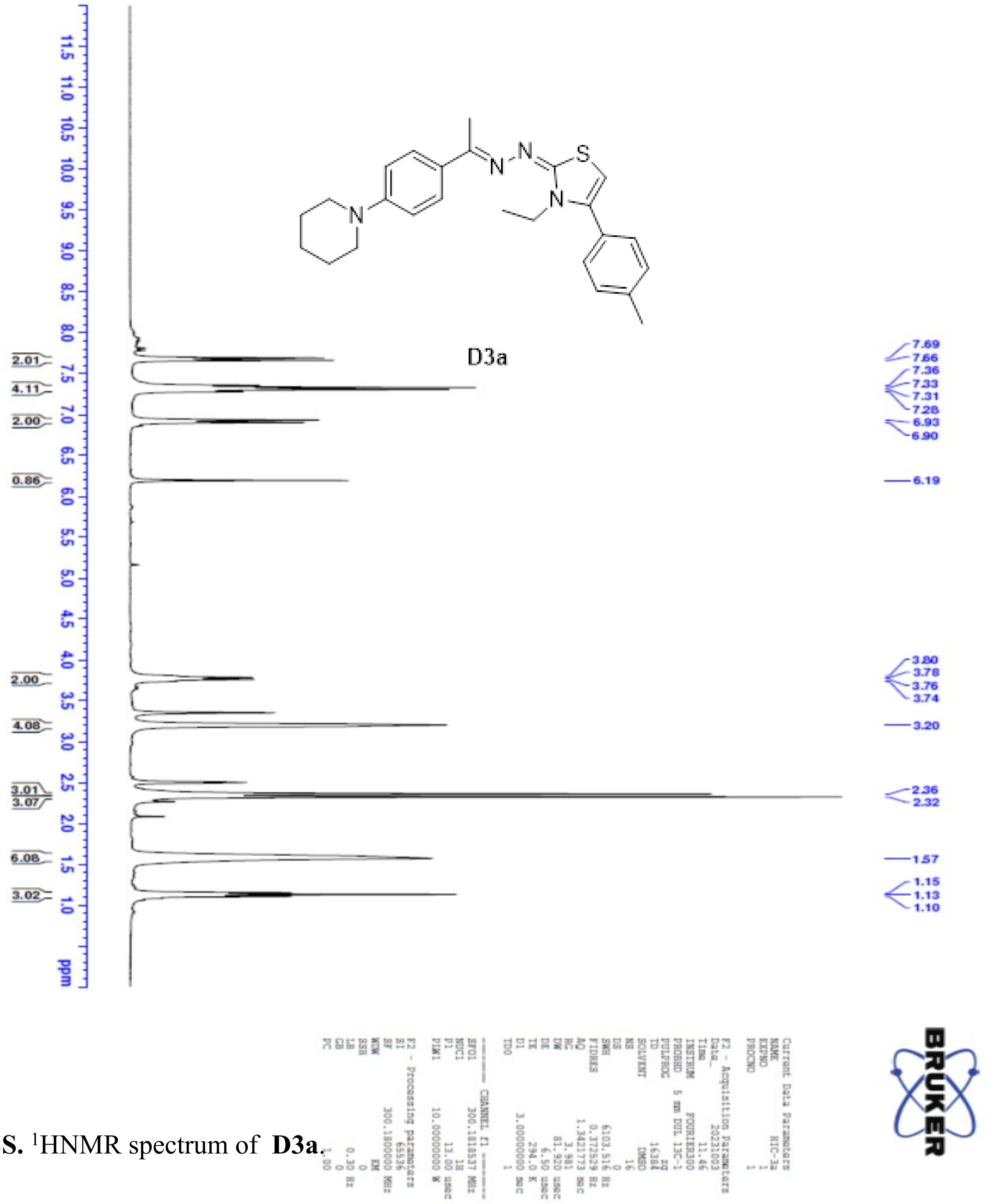

Figure 72S. ^{1}HNMR spectrum of **D3a**.

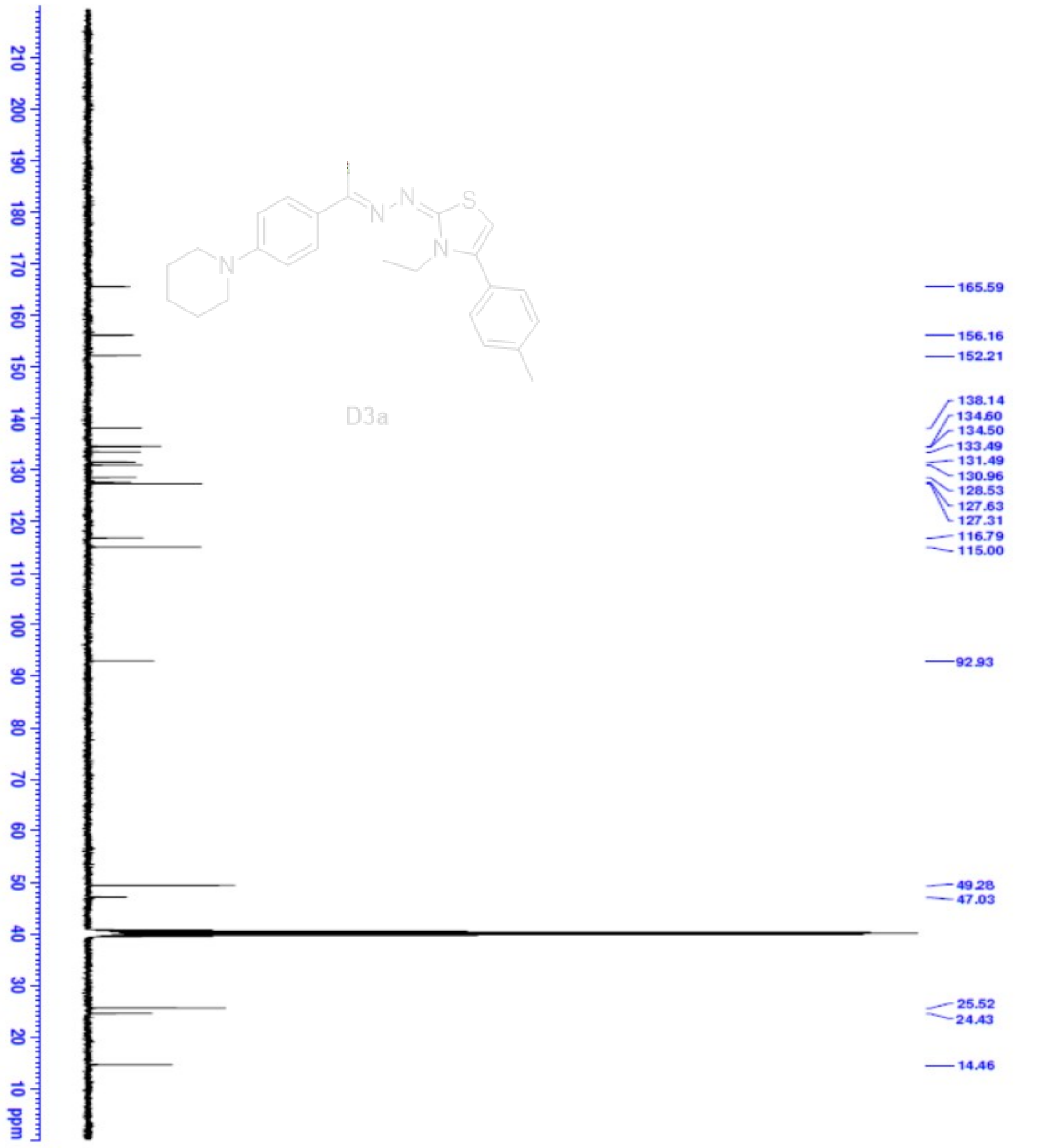

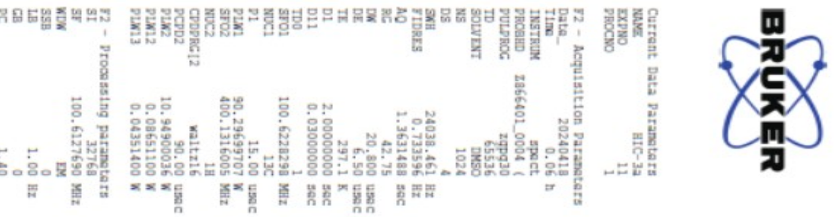

Figure 73S. ^{13}CNMR spectrum of **D3a**.F

Data File: C:\LabSolutions\Data\Analiz\derya\HIC-3A X_582.lcd

Elmt	Val.	Min	Max	Elmt	Val.	Min	Max	Elmt	Val.	Min	Max	Elmt	Val.	Min	Max	Use Adduct
H	1	8	33	O	2	0	3	S	2	1	1	Ru	2	0	0	H
C	4	4	32	F	1	0	0	Cl	1	0	0	Pd	2	0	0	Na
N	3	0	4	P	3	0	0	Br	1	0	0	I	3	0	0	

Error Margin (ppm):	5	DBE Range:	0.0 - 30.0	Electron Ions:	both
HC Ratio:	unlimited	Apply N Rule:	no	Use MSn Info:	yes
Max Isotopes:	3	Isotope RI (%):	1.00	Isotope Res:	9000
MSn Iso RI (%):	10.00	MSn Logic Mode:	AND	Max Results:	50

Event#: 1 MS(E+) Ret. Time : 2.960 Scan# : 445

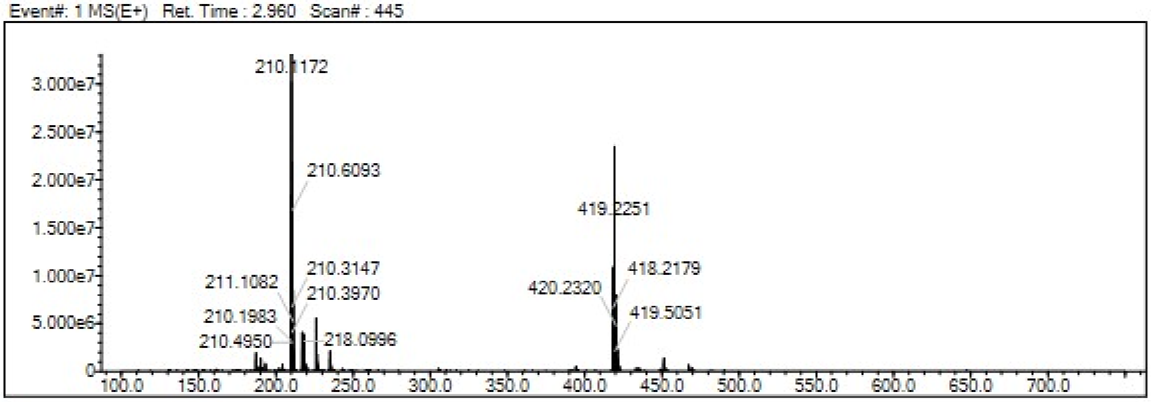

Measured region for 419.2251 m/z

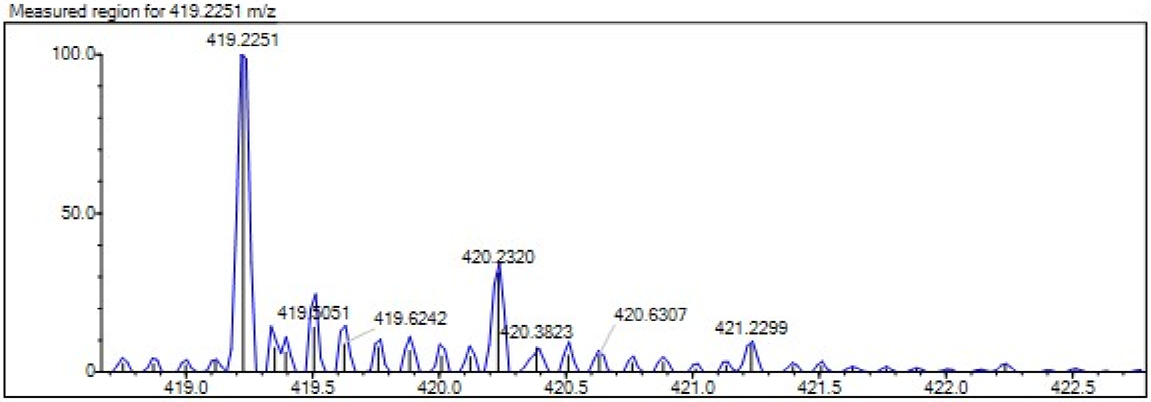

C25 H30 N4 S [M+H]+ : Predicted region for 419.2264 m/z

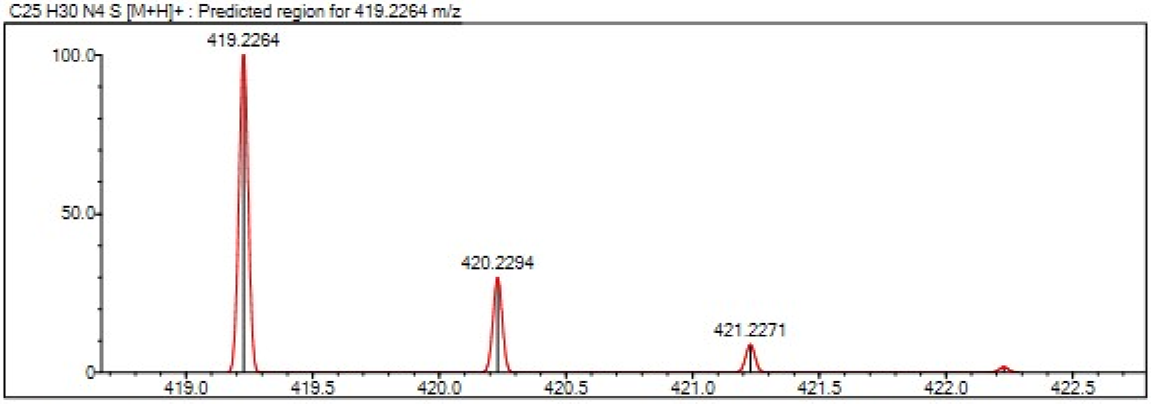

Rank	Score	Formula (M)	Ion	Meas. m/z	Pred. m/z	Df. (mDa)	Df. (ppm)	Iso	DBE
1	90.34	C25 H30 N4 S	[M+H]+	419.2251	419.2264	-1.3	-3.10	95.34	13.0

Figure 74S. HRMS spectrogram of **D3a.**F

Item	Value
Acquired Date&Time	3.05.2024 11:23:45
Acquired by	System Administrator
Filename	C:\Users\dopnalab\Desktop\MASAÜSTÜ\sazan\hic\hic-3b1.ispd
Spectrum name	hic-3b1
Sample name	hic-3b
Sample ID	
Option	
Comment	
No. of Scans	30
Resolution	4 [cm-1]
Apodization	Happ-Genzel

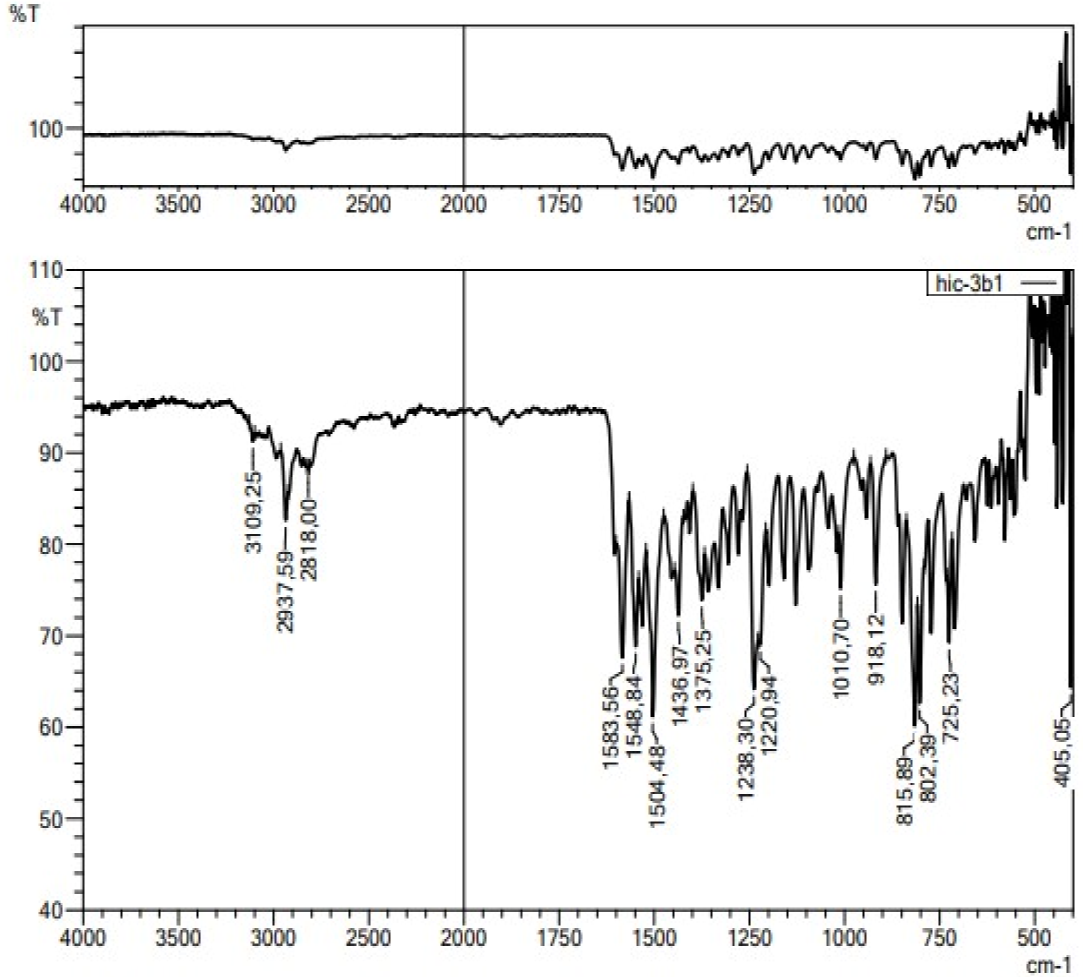

Figure 75S. IR fingerprint of **D3b**.

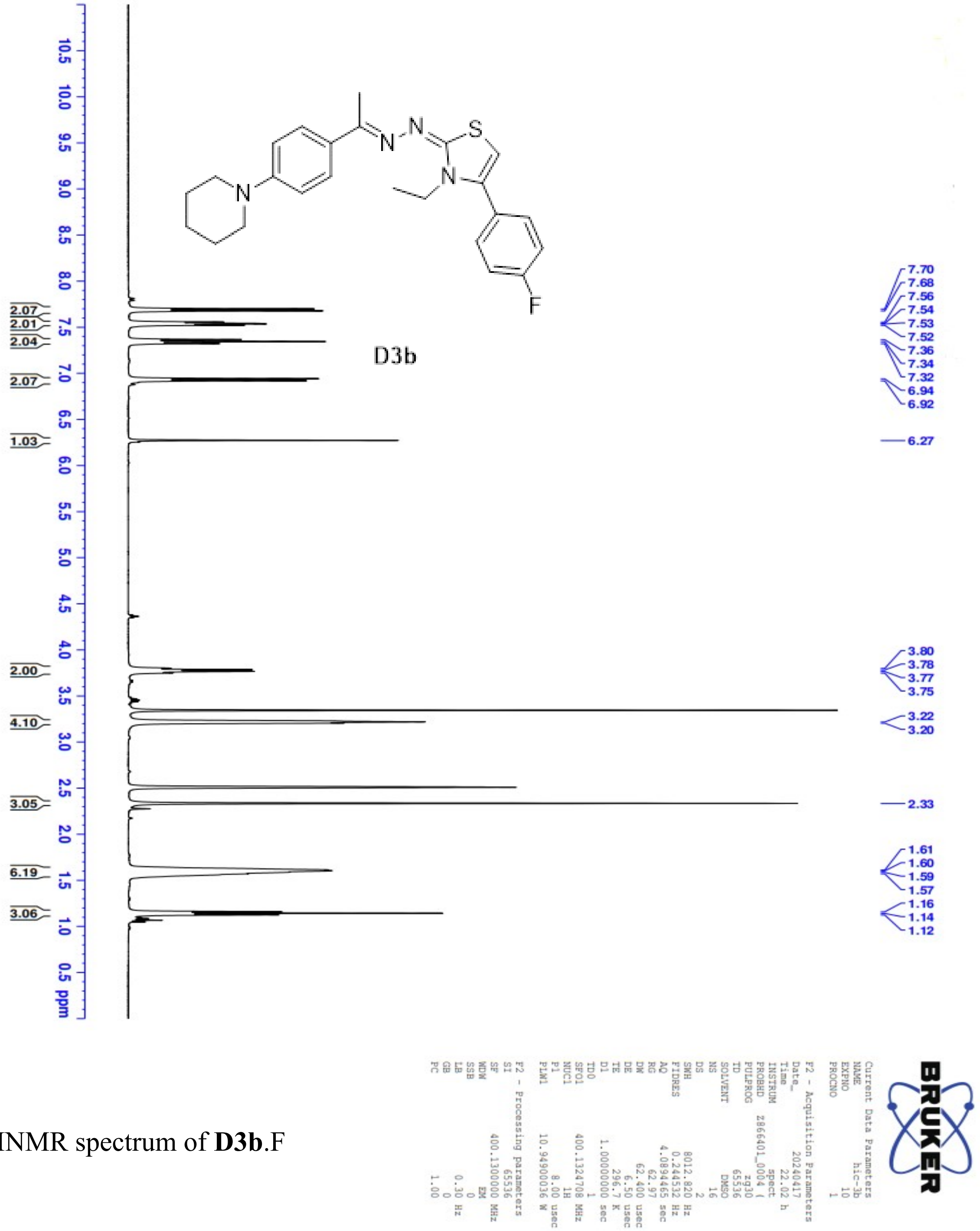

Figure 76S. ^{1}HNMR spectrum of **D3b**.F

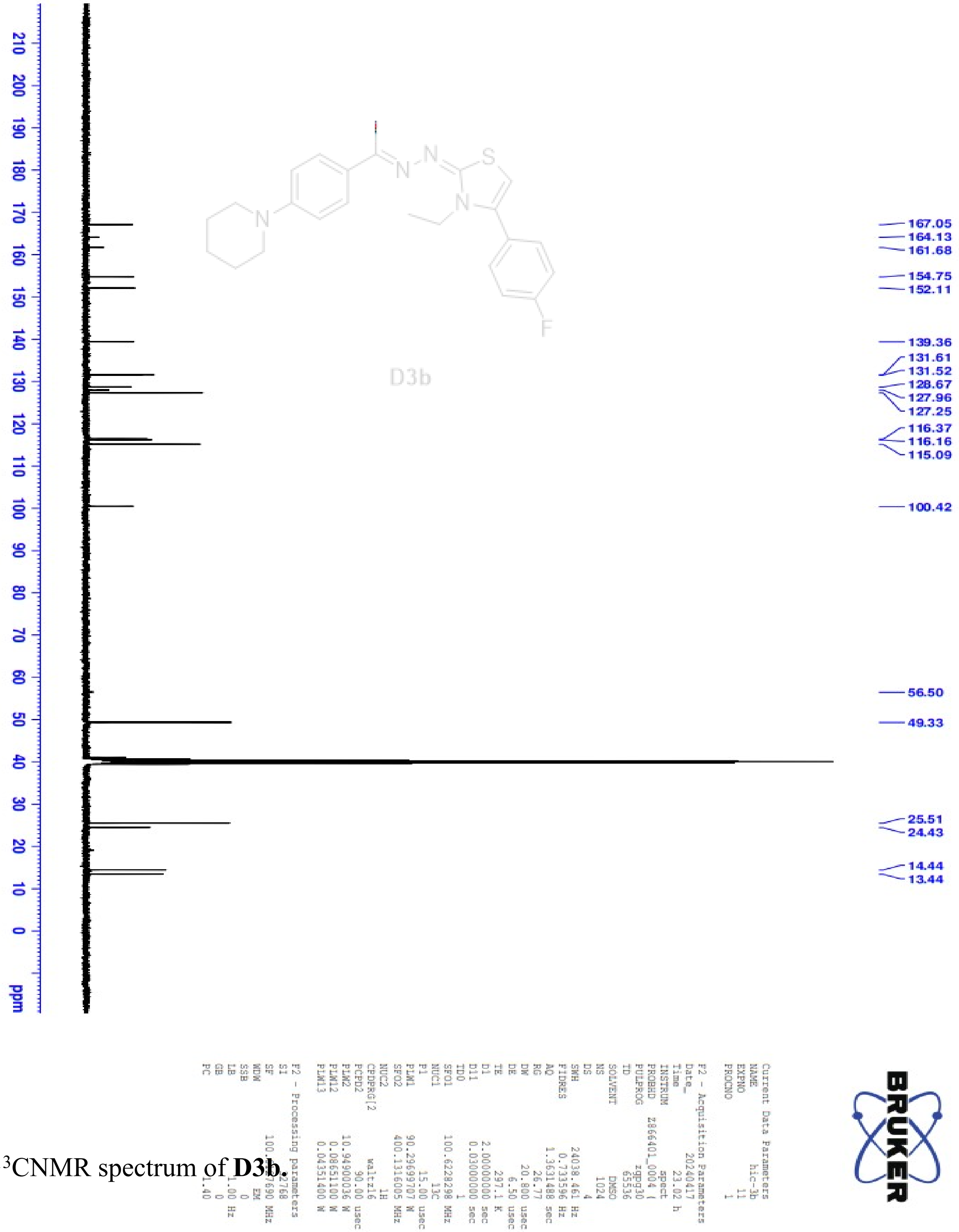

Figure 77S. ^{13}CNMR spectrum of **D3b.**

Data File: C:\LabSolutions\Data\Analiz\derya\HIC-3B_1.lcd

Elmt	Val.	Min	Max	Elmt	Val.	Min	Max	Elmt	Val.	Min	Max	Elmt	Val.	Min	Max	Use Adduct
H	1	8	33	O	2	0	3	S	2	1	1	Ru	2	0	0	H
C	4	4	32	F	1	1	1	Cl	1	0	0	Pd	2	0	0	Na
N	3	0	4	P	3	0	0	Br	1	0	0	I	3	0	0	

Error Margin (ppm):	5	DBE Range:	0.0 - 30.0	Electron Ions:	both
HC Ratio:	unlimited	Apply N Rule:	no	Use MSn Info:	yes
Max Isotopes:	3	Isotope RI (%):	1.00	Isotope Res:	9000
MSn Iso RI (%):	10.00	MSn Logic Mode:	AND	Max Results:	50

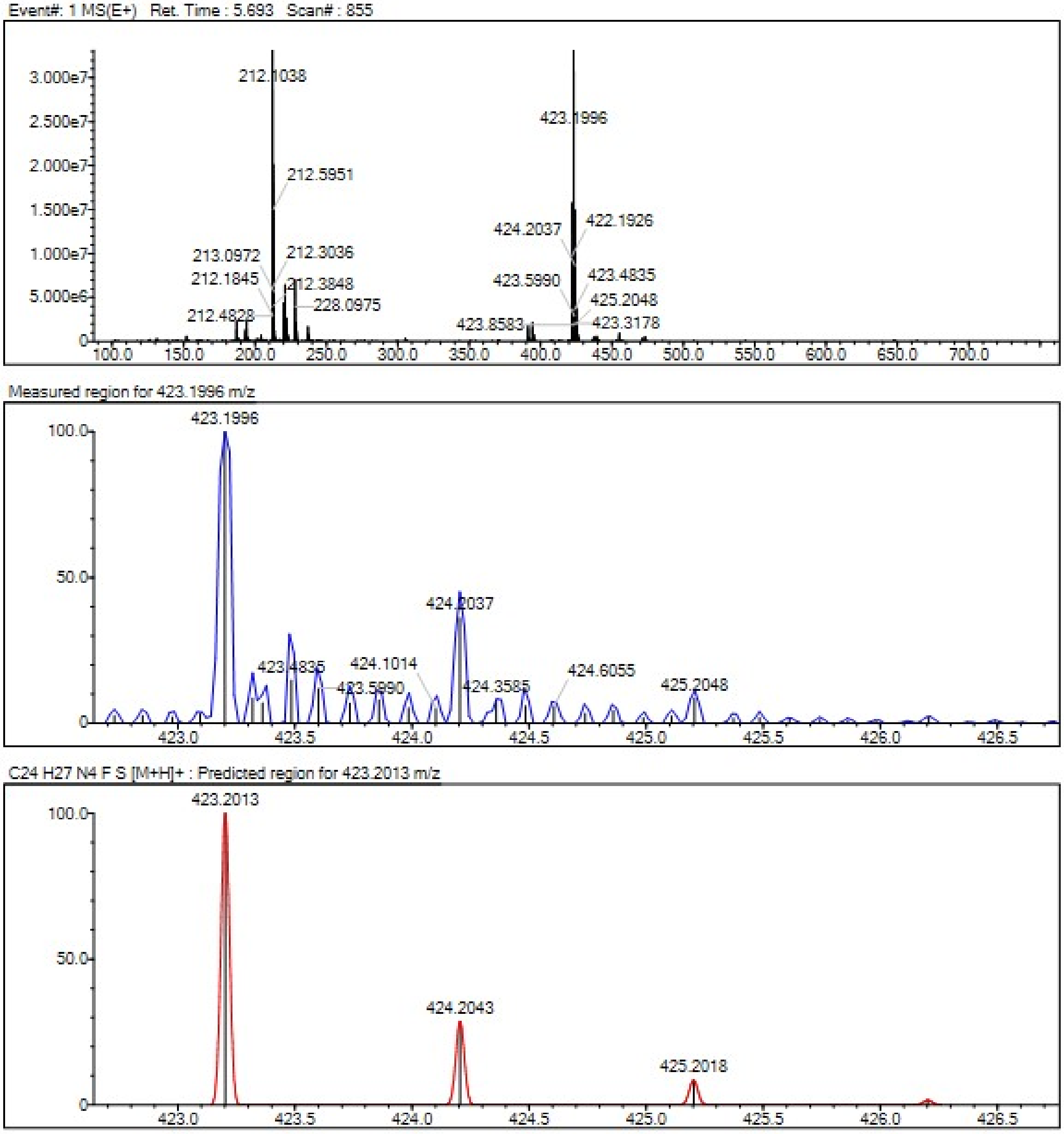

Rank	Score	Formula (M)	Ion	Meas. m/z	Pred. m/z	Df. (mDa)	Df. (ppm)	Iso	DBE
2	61.09	C24 H27 N4 F S	[M+H]+	423.1996	423.2013	-1.7	-4.02	66.08	13.0

Figure 78S. HRMS spectrogram of **D3b.**

	Item	Value
	Acquired Date&Time	3.05.2024 11:28:37
	Acquired by	System Administrator
	Filename	C:\Users\dopnalab\Desktop\MASAÜSTÜ\sazan\hic\hic-3c1.ispd
	Spectrum name	hic-3c1
	Sample name	hic-3c
	Sample ID	
	Option	
	Comment	
	No. of Scans	30
	Resolution	4 [cm-1]
	Apodization	Happ-Genzel

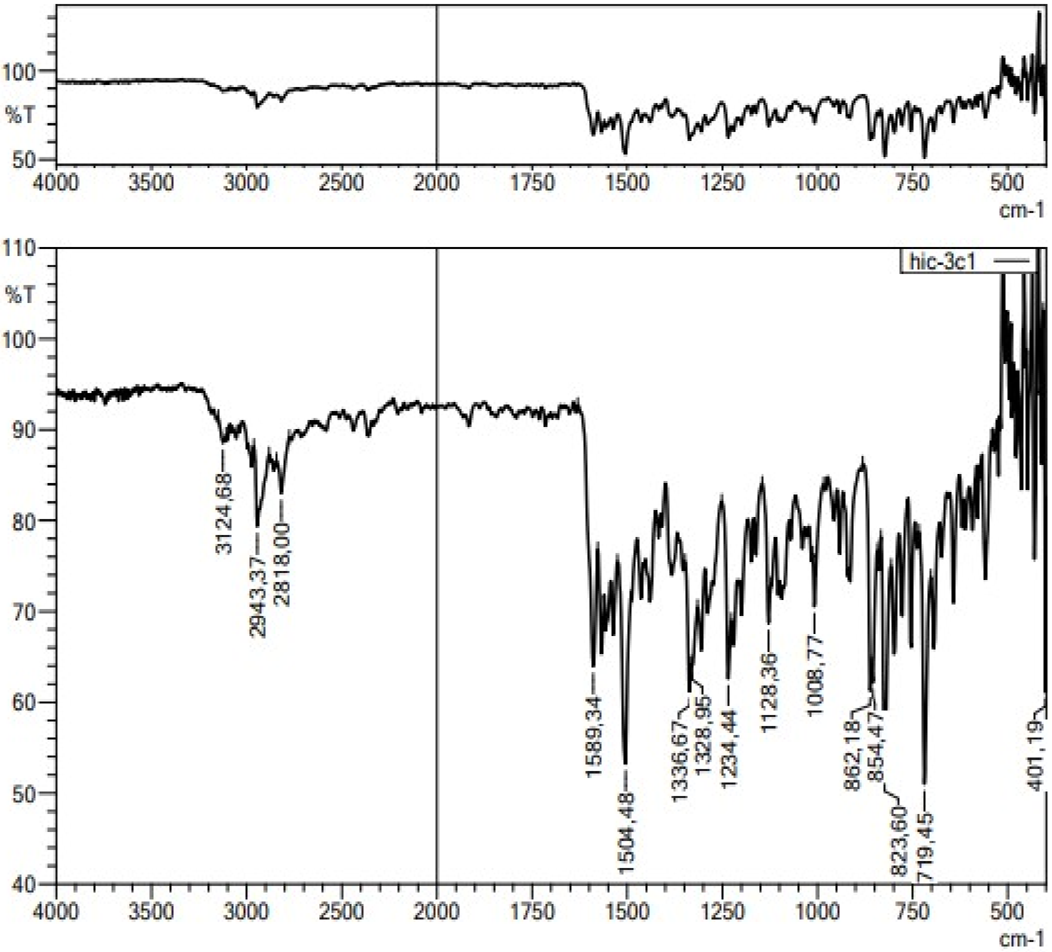

Figure 79S. IR fingerprint of **D3c**.

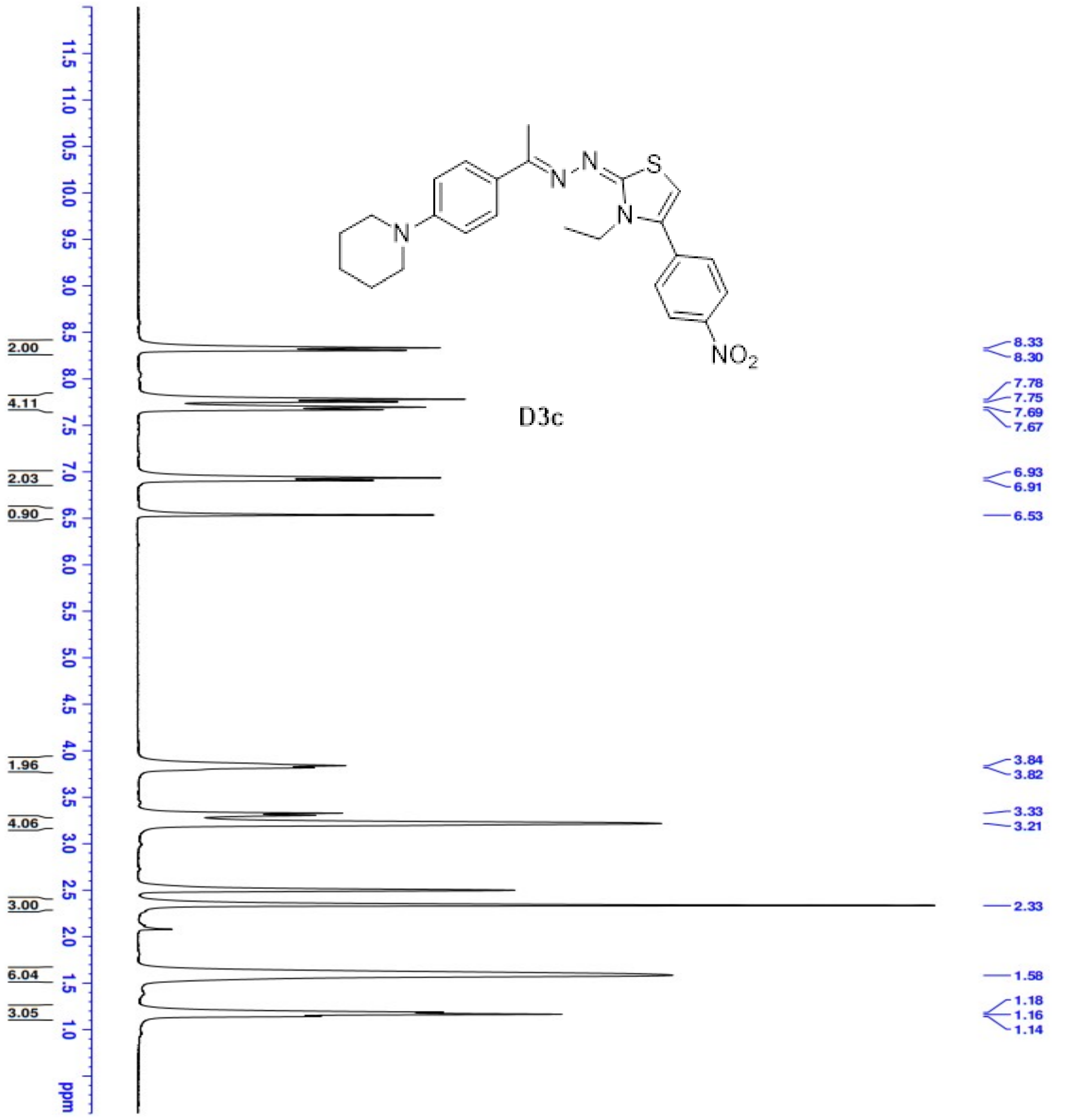

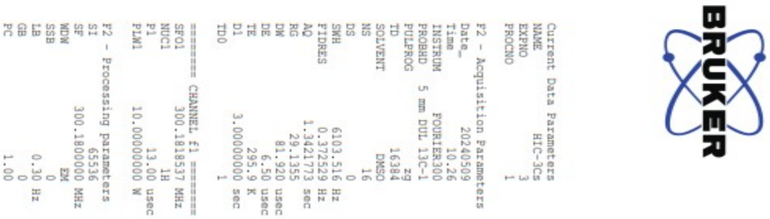

Figure 80S. ^{1}HNMR spectrum of **D3c**.

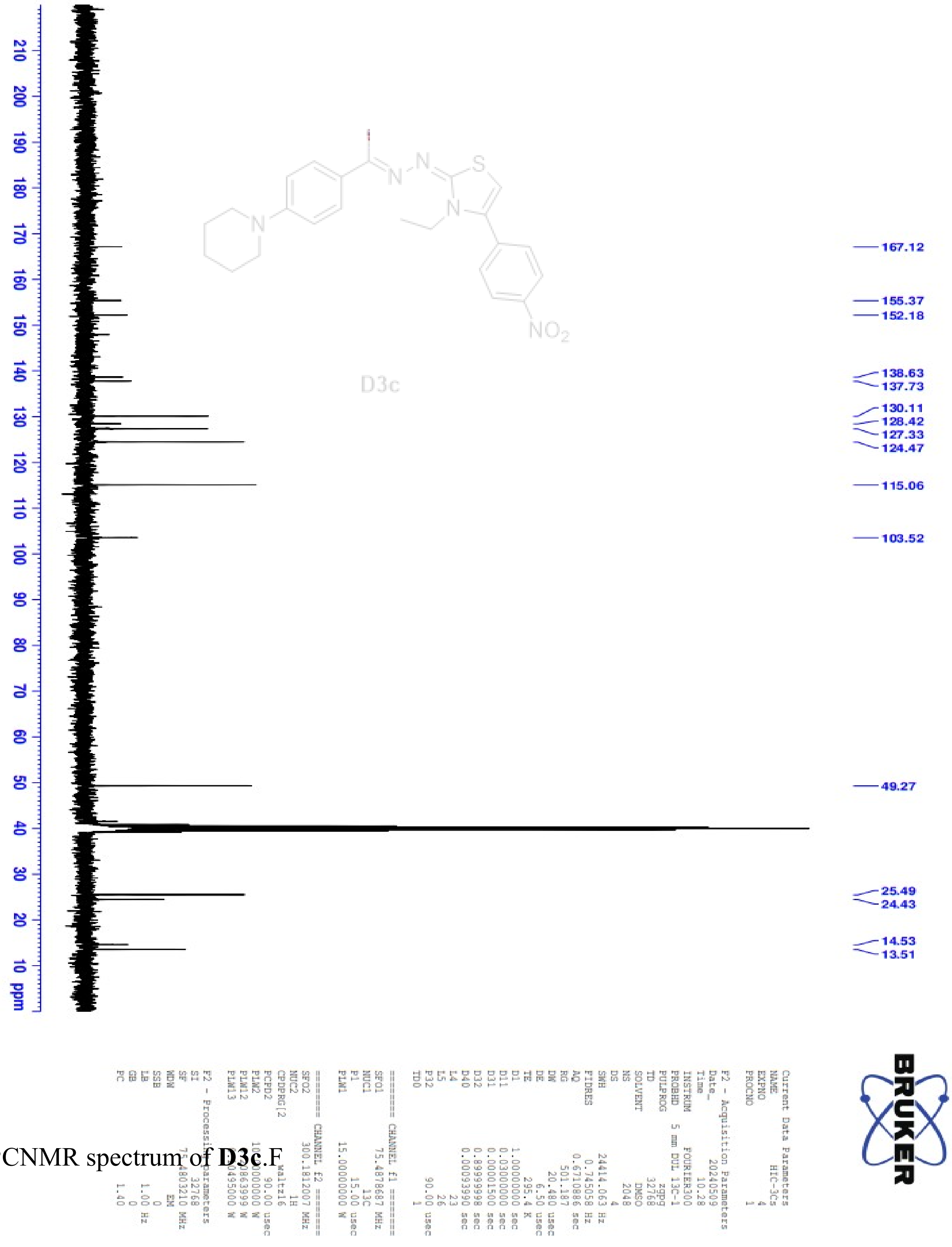

Figure 81S. ^{13}CNMR spectrum of **D3c**.F

Data File: C:\LabSolutions\Data\Analiz\derya\HIC-3D ALT_3.lcd

Elmt	Val.	Min	Max	Elmt	Val.	Min	Max	Elmt	Val.	Min	Max	Elmt	Val.	Min	Max	Use Adduct
H	1	8	33	O	2	0	3	S	2	1	1	Ru	2	0	0	H
C	4	4	32	F	1	0	0	Cl	1	0	0	Pd	2	0	0	Na
N	3	0	5	P	3	0	0	Br	1	0	0	I	3	0	0	

Error Margin (ppm): 5
HC Ratio: unlimited
Max Isotopes: 3
MSn Iso RI (%): 10.00

DBE Range: 0.0 - 30.0
Apply N Rule: no
Isotope RI (%): 1.00
MSn Logic Mode: AND

Electron Ions: both
Use MSn Info: yes
Isotope Res: 9000
Max Results: 50

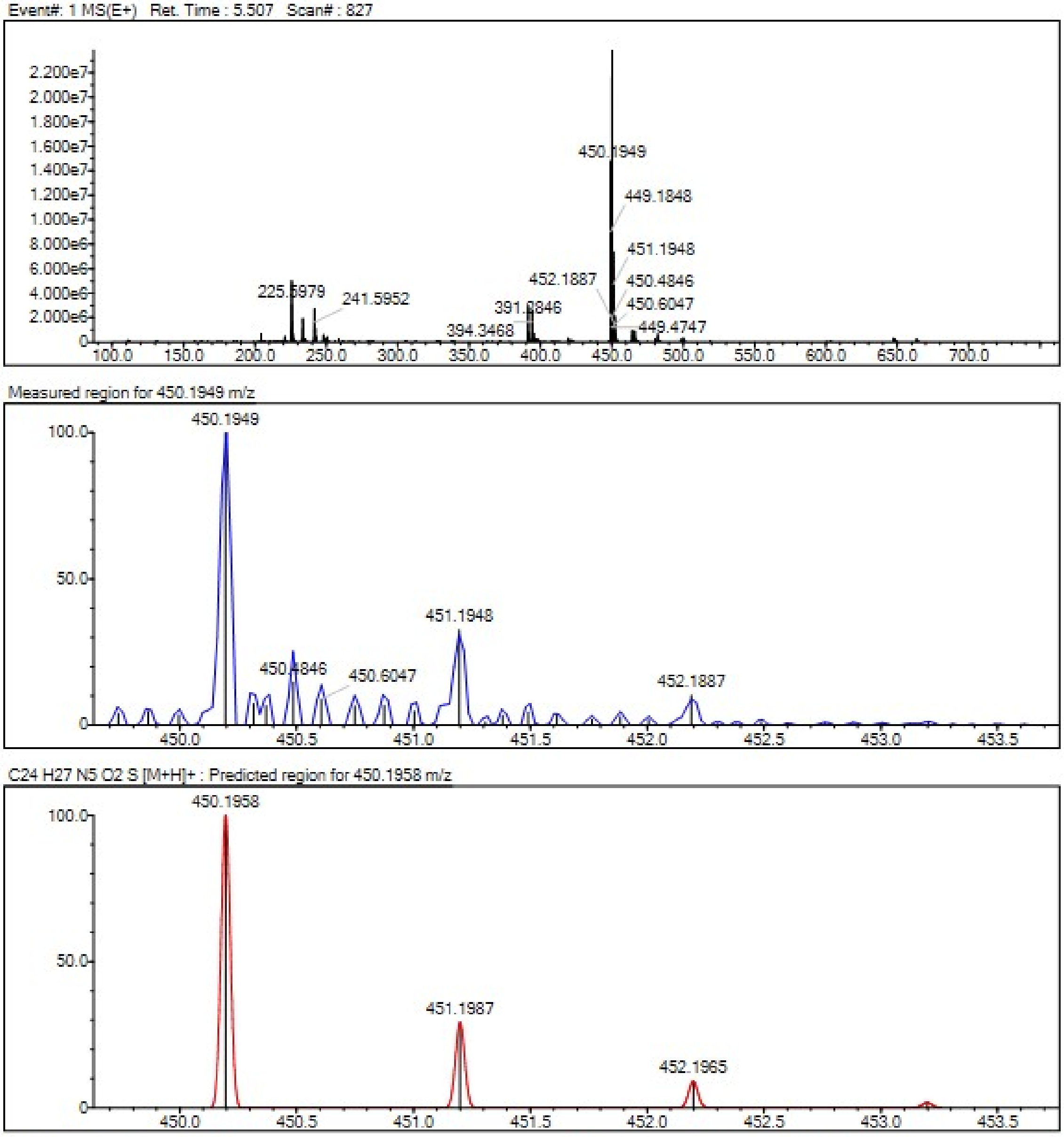

Rank	Score	Formula (M)	Ion	Meas. m/z	Pred. m/z	Df. (mDa)	Df. (ppm)	Iso	DBE
1	73.22	C24 H27 N5 O2 S	[M+H]+	450.1949	450.1958	-0.9	-2.00	75.10	14.0

Figure 82S. HRMS spectrogram of **D3c**.F

Item	Value
Acquired Date&Time	3.05.2024 11:34:38
Acquired by	System Administrator
Filename	C:\Users\dopnalab\Desktop\MASAÜSTÜ\sazan\hic\hic-3d1.ispd
Spectrum name	hic-3d1
Sample name	hic-3d
Sample ID	
Option	
Comment	
No. of Scans	30
Resolution	4 [cm-1]
Apodization	Happ-Genzel

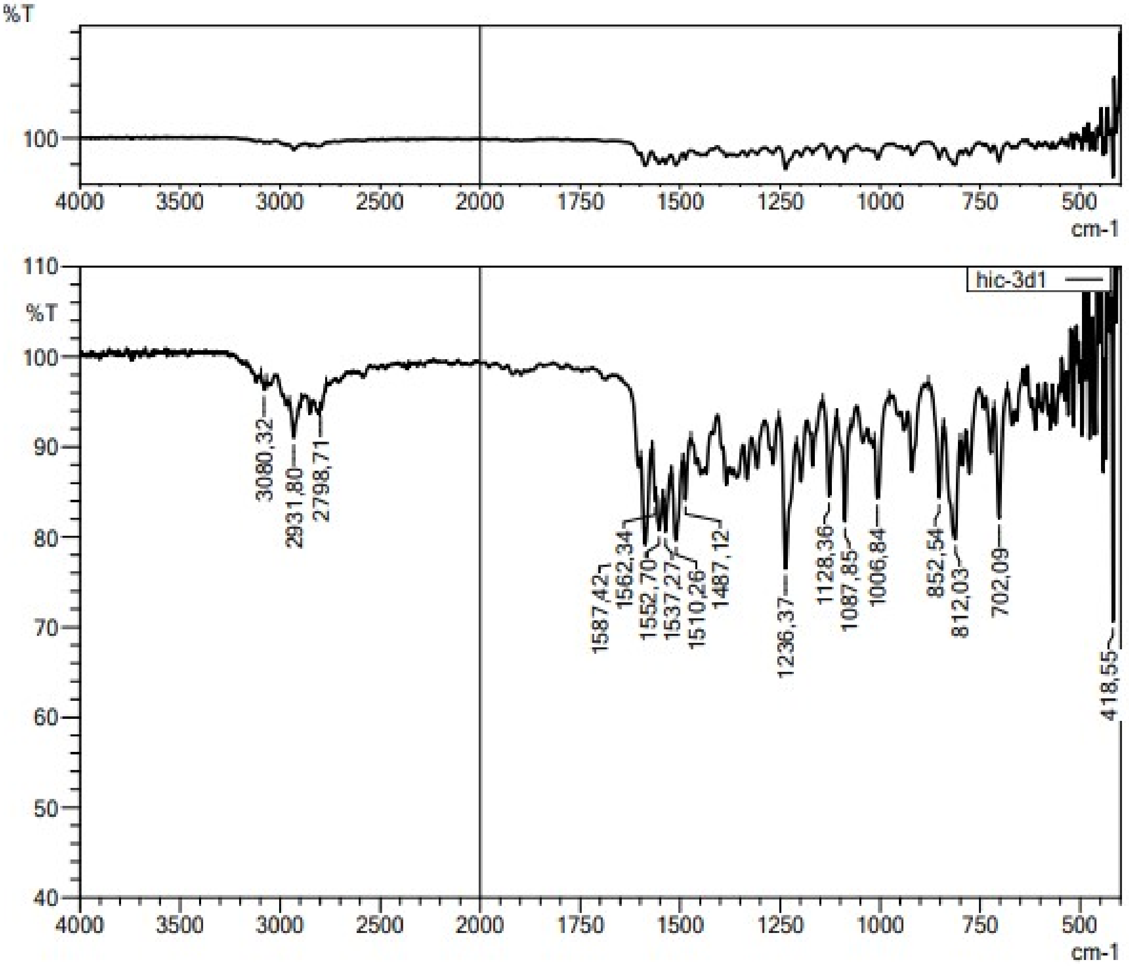

Figure 83S. IR fingerprint of **D3d**.F

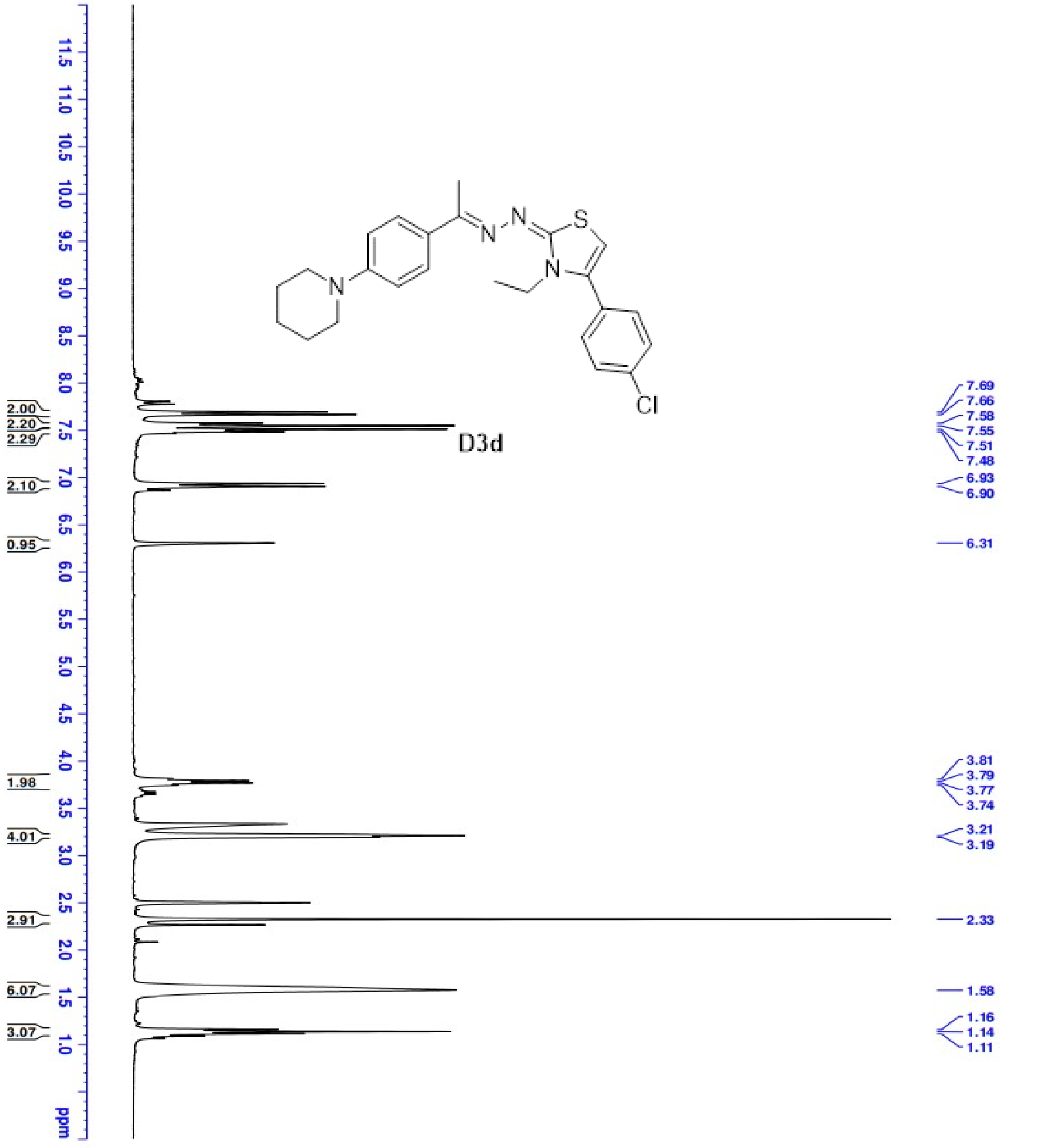

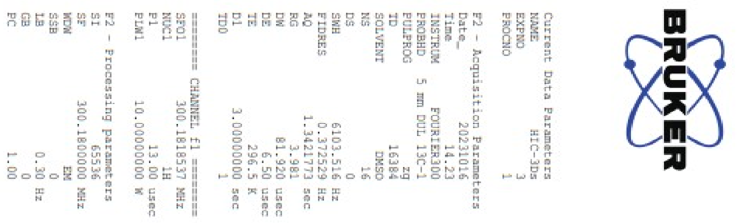

Figure 84S. ^{1}HNMR spectrum of **D3d.**

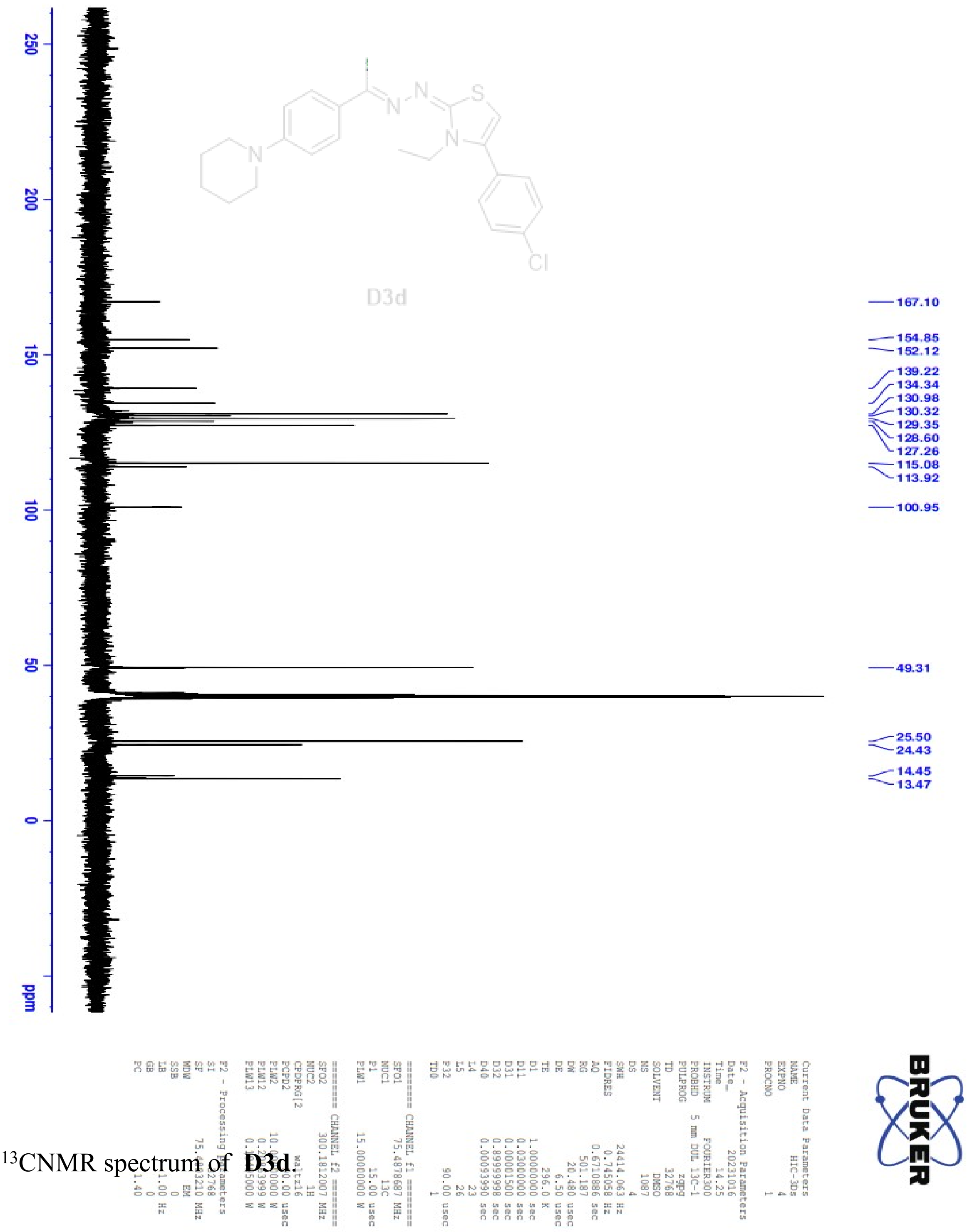

Figure 85S. ^{13}CNMR spectrum of **D3d.**

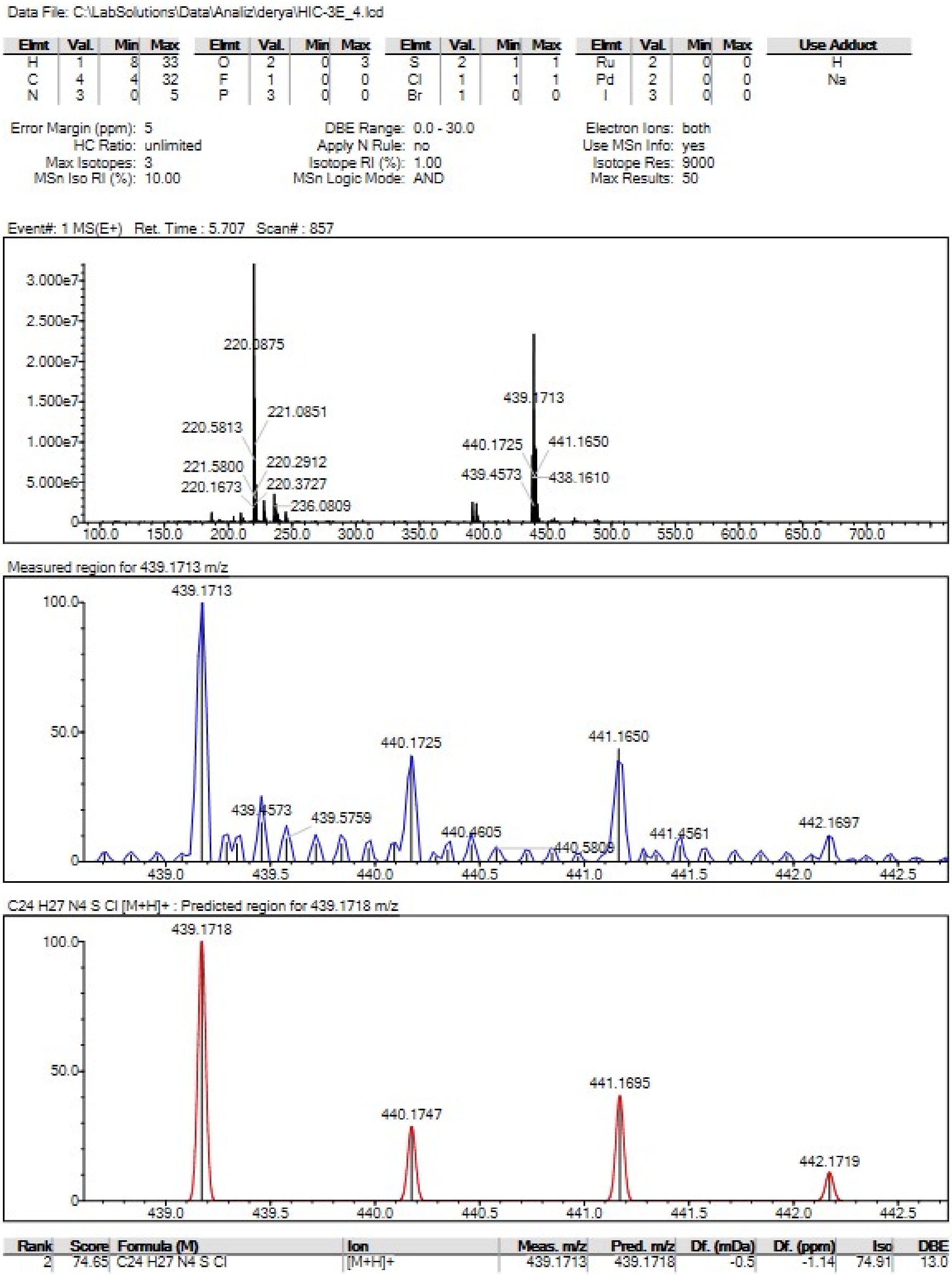

Figure 86S. HRMS spectrogram of **D3d**.

Item	Value
Acquired Date&Time	3.05.2024 11:38:53
Acquired by	System Administrator
Filename	C:\Users\dopnalab\Desktop\MASAÜSTÜ\sazan\hic\hic-3e1.ispd
Spectrum name	hic-3e1
Sample name	hic-3e
Sample ID	
Option	
Comment	
No. of Scans	30
Resolution	4 [cm-1]
Apodization	Happ-Genzel

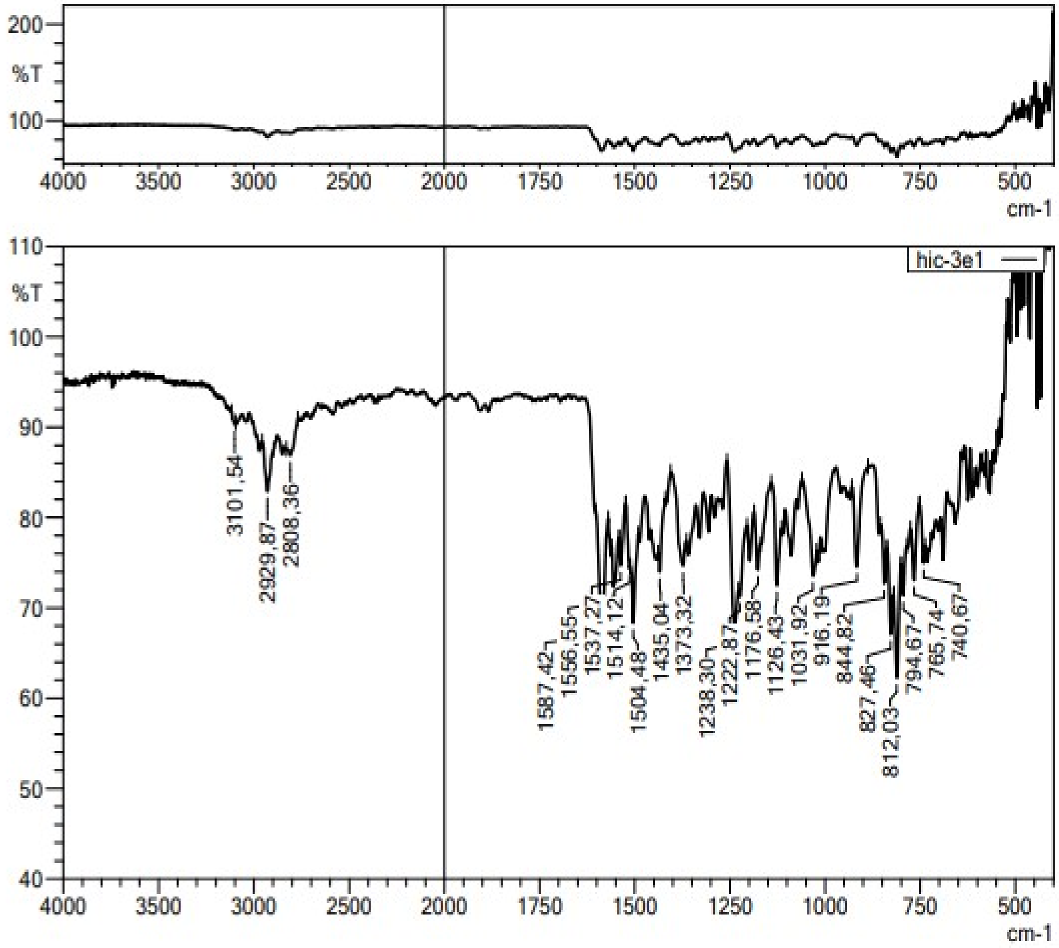

Figure 87S. IR fingerprint of **D3e**.

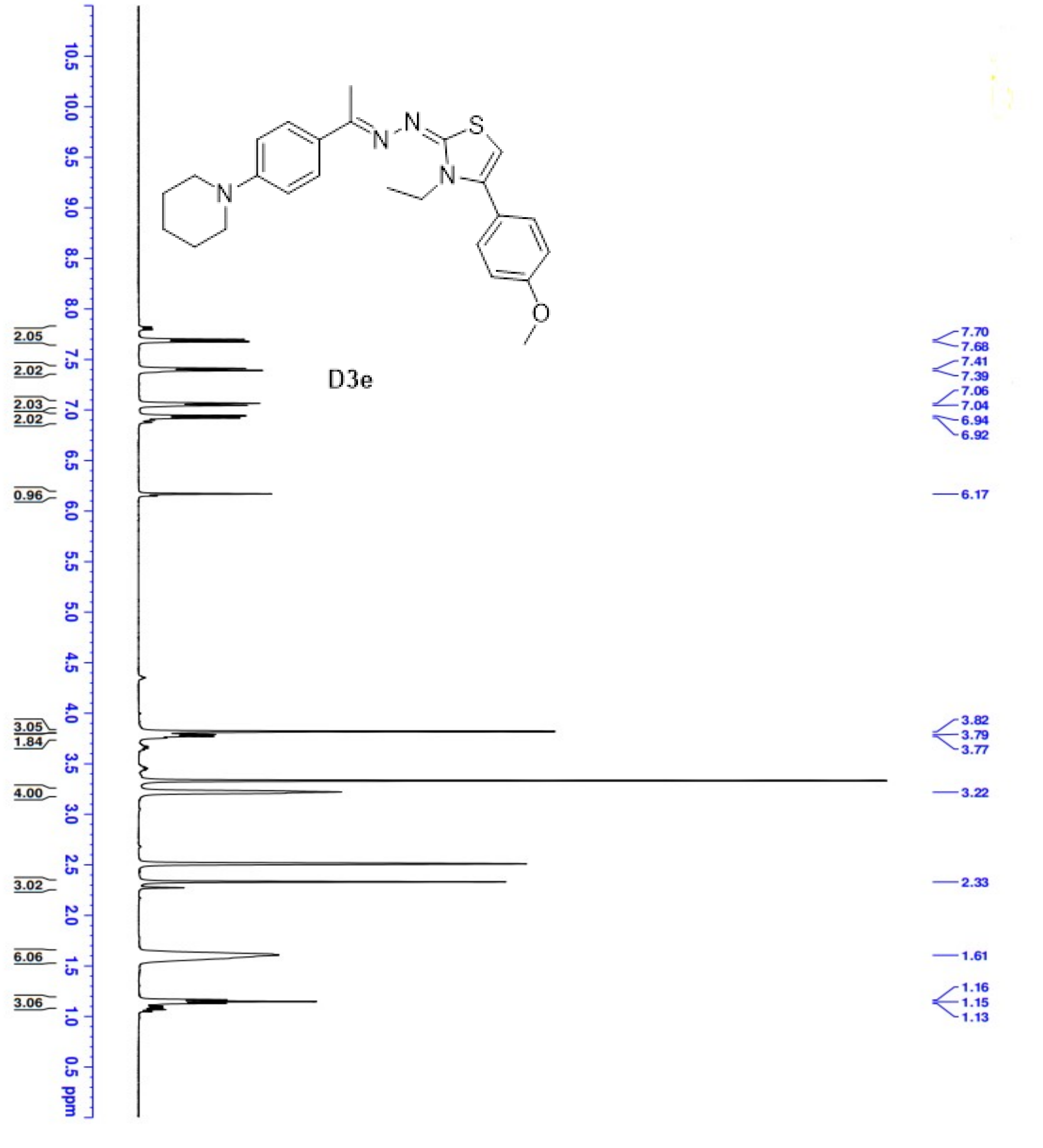

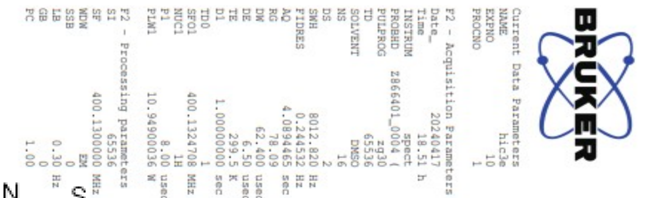

Figure 88S.^{1}HNMR spectrum of **D3e**.

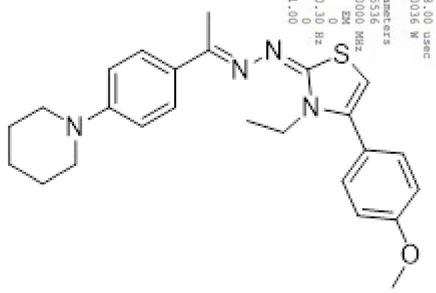

D3e

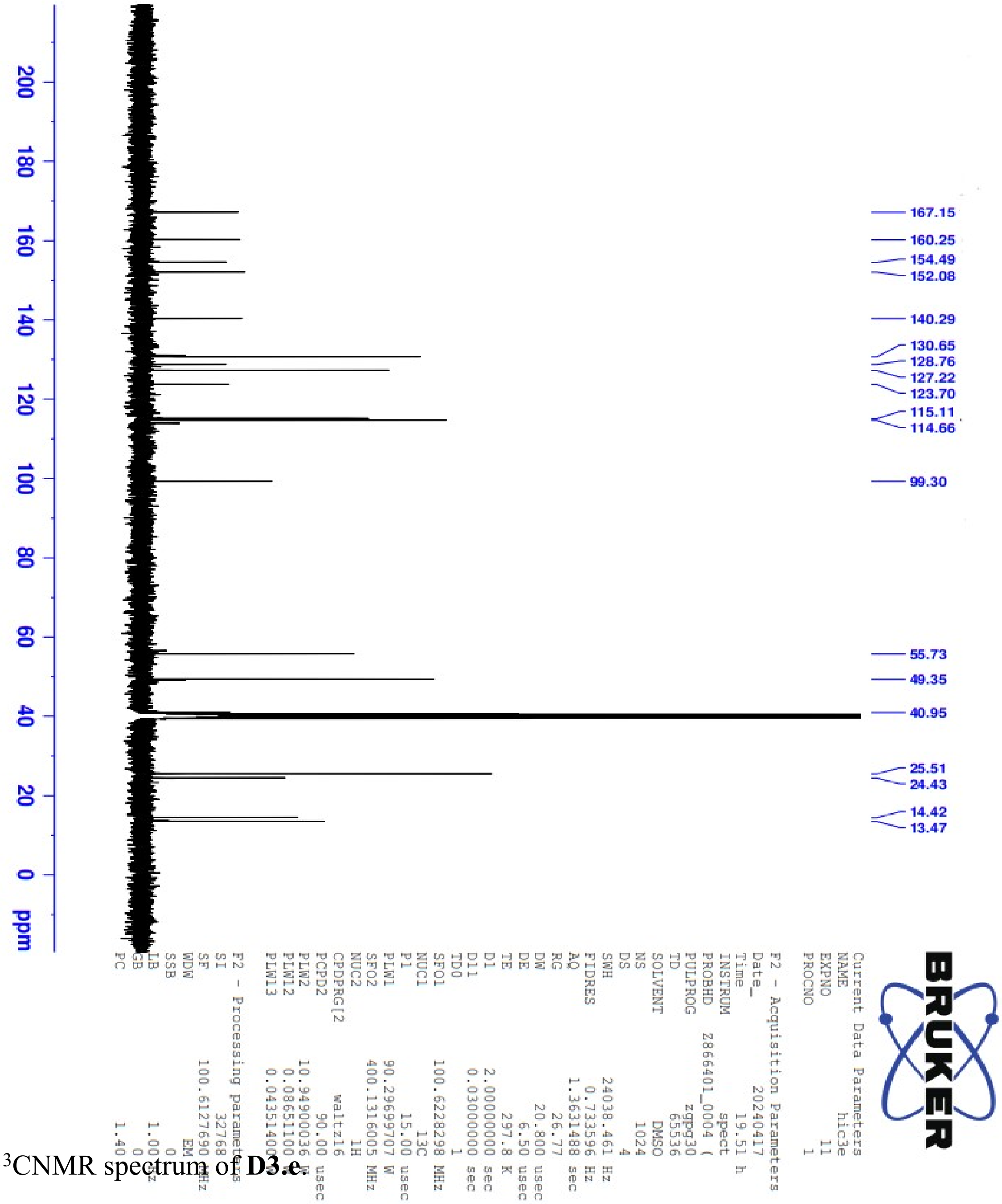

Figure 89S.^{13}CNMR spectrum of **D3.e.**

Data File: C:\LabSolutions\Data\Analiz\derya\HIC-3E_4.lcd

Elmt	Val.	Min	Max	Elmt	Val.	Min	Max	Elmt	Val.	Min	Max	Elmt	Val.	Min	Max	Use Adduct
H	1	8	33	O	2	0	3	S	2	1	1	Ru	2	0	0	H
C	4	4	32	F	1	0	0	Cl	1	0	0	Pd	2	0	0	Na
N	3	0	5	P	3	0	0	Br	1	0	0	I	3	0	0	

Error Margin (ppm):	5	DBE Range:	0.0 - 30.0	Electron Ions:	both
HC Ratio:	unlimited	Apply N Rule:	no	Use MSn Info:	yes
Max Isotopes:	3	Isotope RI (%):	1.00	Isotope Res:	9000
MSn Iso RI (%):	10.00	MSn Logic Mode:	AND	Max Results:	50

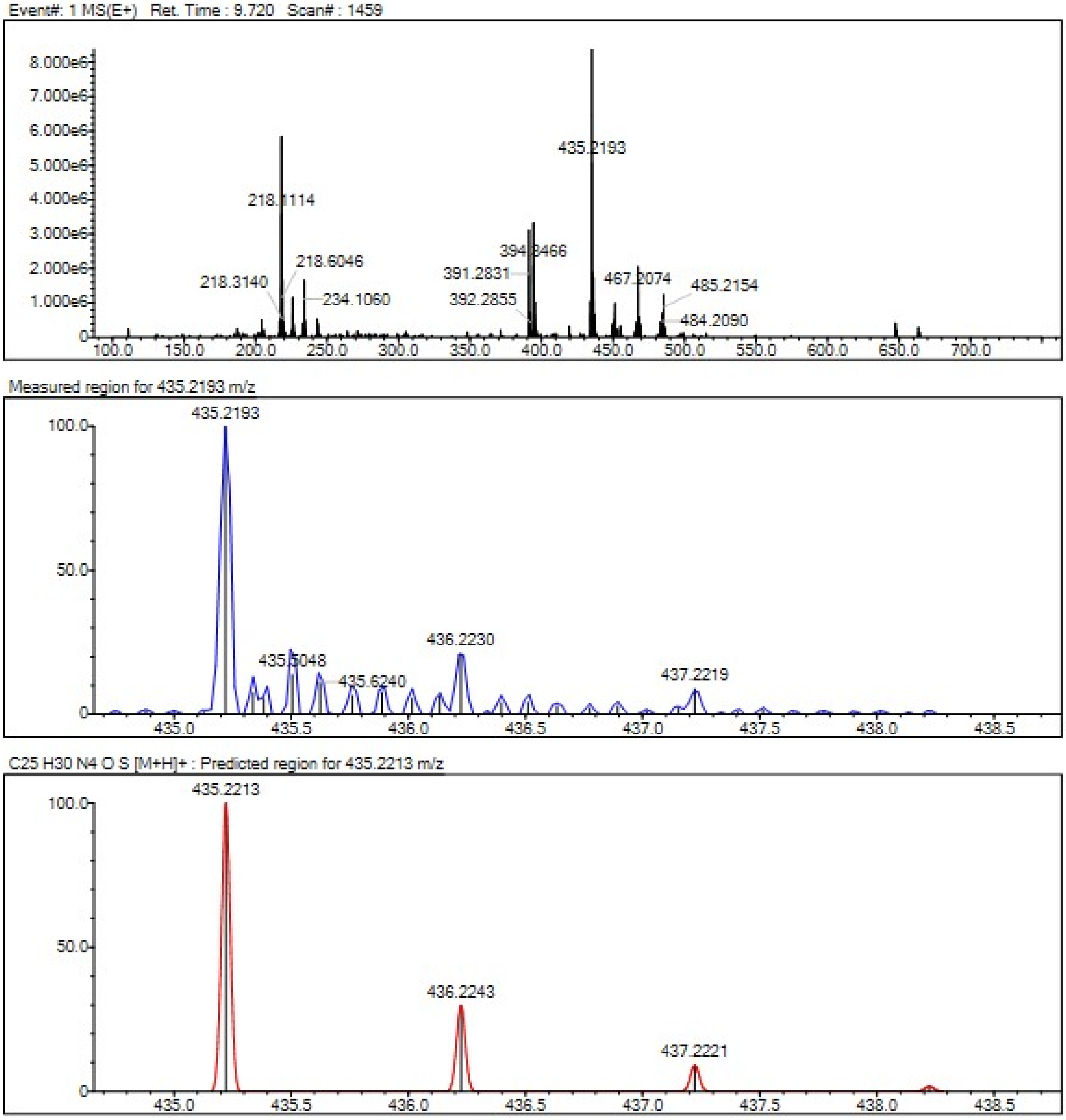

Rank	Score	Formula (M)	Ion	Meas. m/z	Pred. m/z	Df. (mDa)	Df. (ppm)	Iso	DBE
2	73.23	C25 H30 N4 O S	[M+H]+	435.2193	435.2213	-2.0	-4.60	80.48	13.0

Figure 90S. HRMS spectrogram of **D3e**.F

Item	Value
Acquired Date&Time	3.05.2024 11:42:34
Acquired by	System Administrator
Filename	C:\Users\dopnalab\Desktop\MASAÜSTÜ\sazan\hic\hic-3f1.ispd
Spectrum name	hic-3f1
Sample name	hic-3f
Sample ID	
Option	
Comment	
No. of Scans	30
Resolution	4 [cm-1]
Apodization	Happ-Genzel

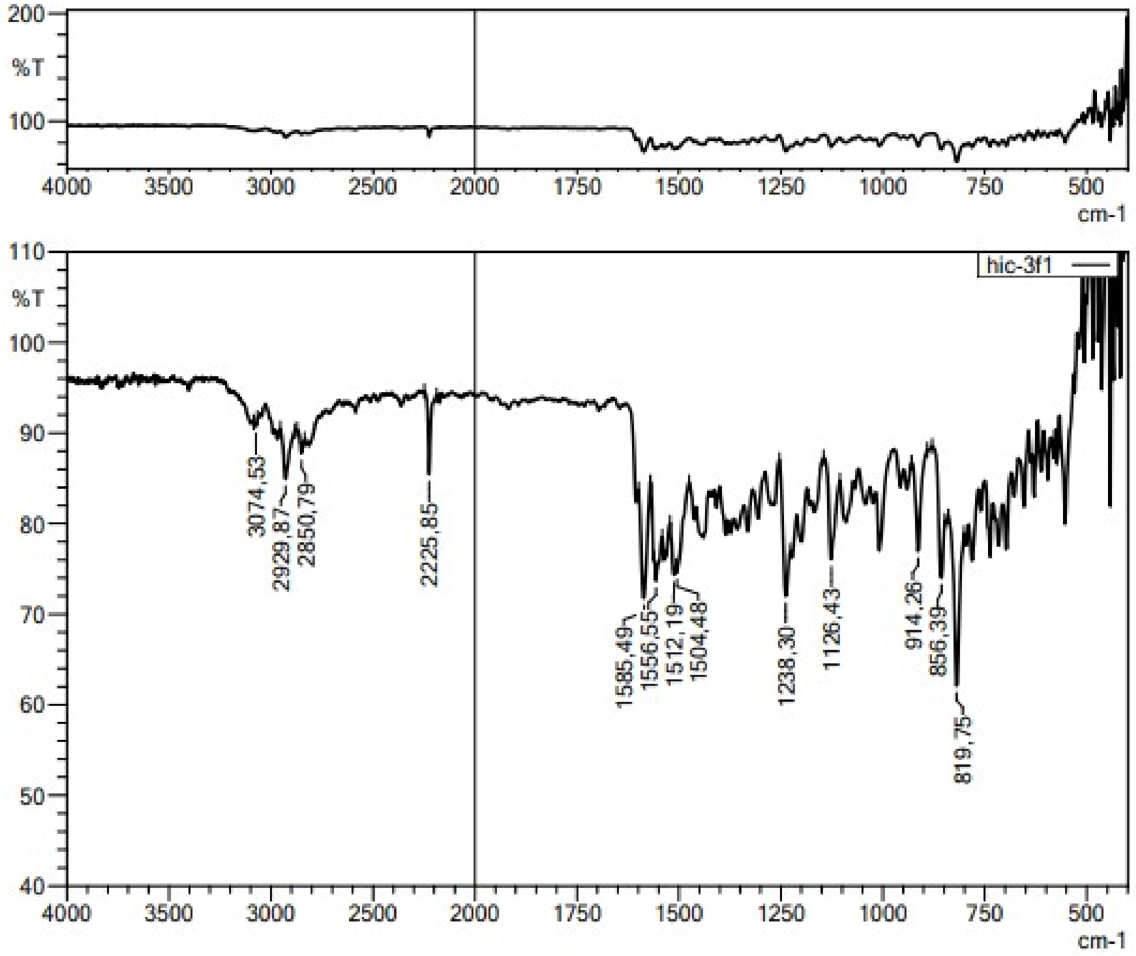

Figure 91S. IR fingerprint of **D3f.**F

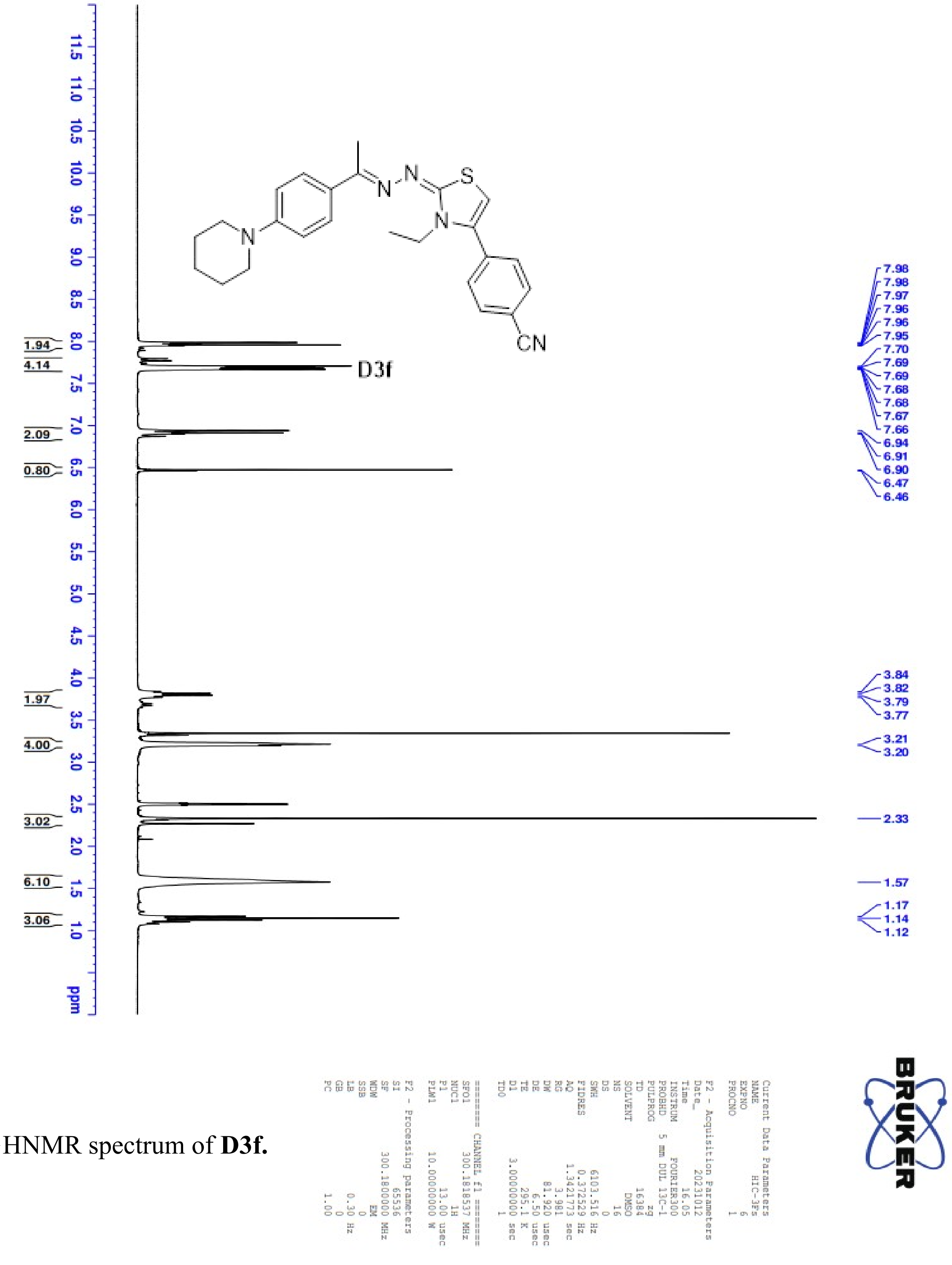

Figure 92S. ^{1}HNMR spectrum of **D3f.**

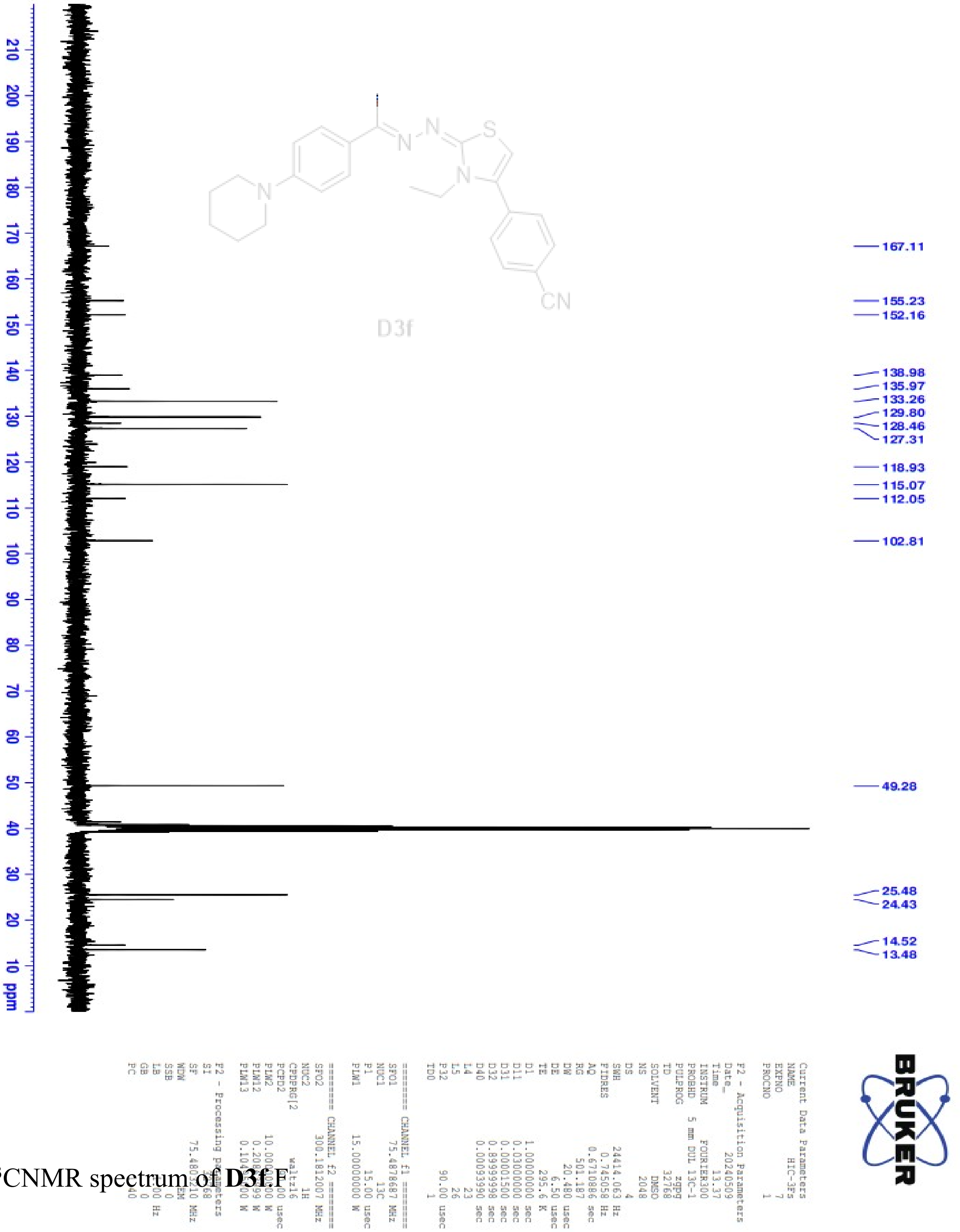

Figure 93S. ^{13}CNMR spectrum of **D3f**.F

Data File: C:\LabSolutions\Data\Analiz\derya\HIC-3F _7.lcd

Elmt	Val.	Min	Max	Elmt	Val.	Min	Max	Elmt	Val.	Min	Max	Elmt	Val.	Min	Max	Use Adduct
H	1	8	33	O	2	0	3	S	2	1	1	Ru	2	0	0	H
C	4	4	32	F	1	0	0	Cl	1	0	0	Pd	2	0	0	Na
N	3	0	5	P	3	0	0	Br	1	0	0	I	3	0	0	

Error Margin (ppm):	5	DBE Range:	0.0 - 30.0	Electron Ions:	both
HC Ratio:	unlimited	Apply N Rule:	no	Use MSn Info:	yes
Max Isotopes:	3	Isotope RI (%):	1.00	Isotope Res:	9000
MSn Iso RI (%):	10.00	MSn Logic Mode:	AND	Max Results:	50

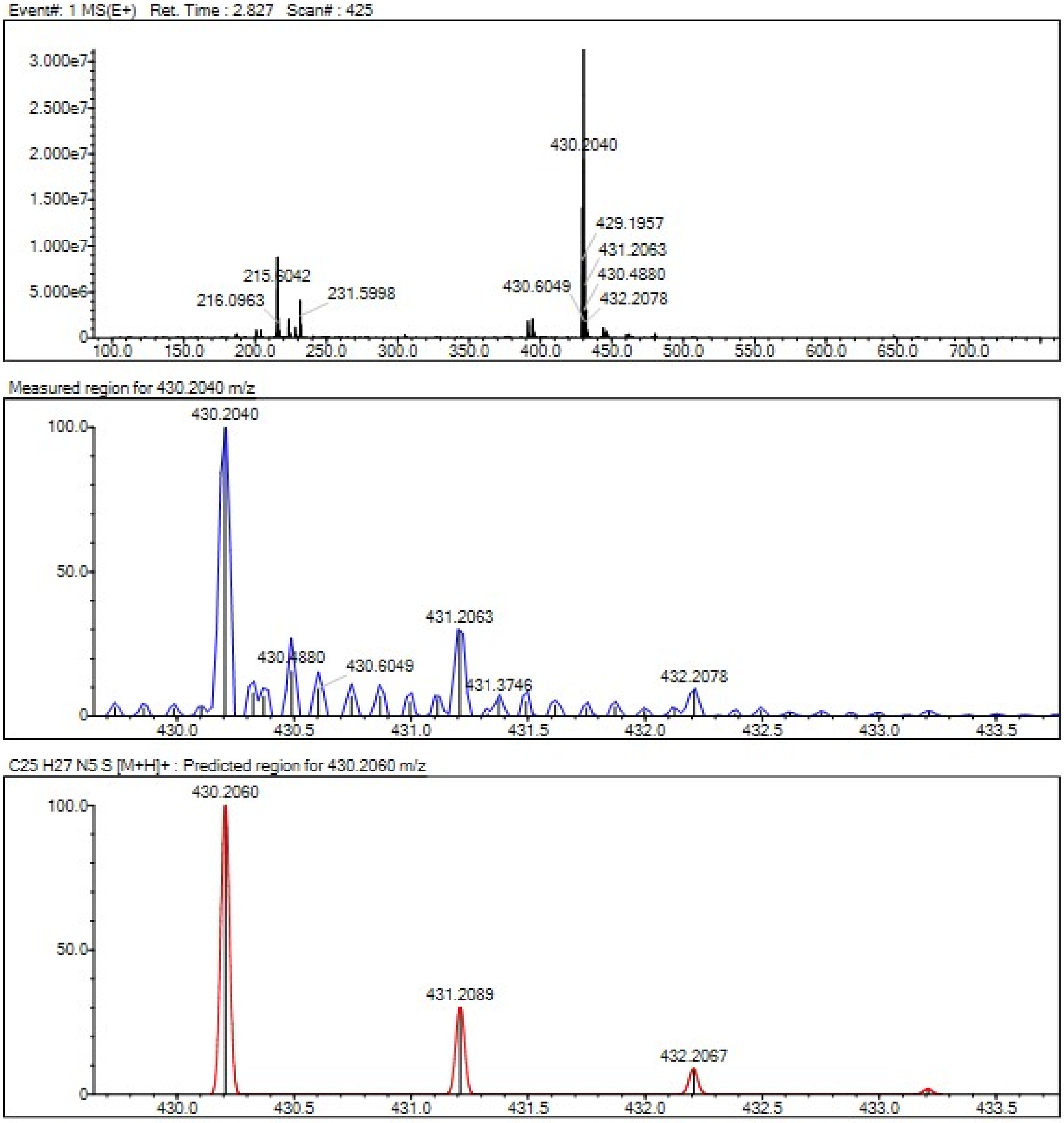

Rank	Score	Formula (M)	Ion	Meas. m/z	Pred. m/z	Df. (mDa)	Df. (ppm)	Iso	DBE
3	83.27	C25 H27 N5 S	[M+H]+	430.2040	430.2060	-2.0	-4.65	91.63	15.0

Figure 94S. HRMS spectrogram of **D3f.**

Item	Value
Acquired Date&Time	3.05.2024 11:47:28
Acquired by	System Administrator
Filename	C:\Users\dopnalab\Desktop\MASAÜSTÜ\sazan\hic\hic-3h1.ispd
Spectrum name	hic-3h1
Sample name	hic-3h
Sample ID	
Option	
Comment	
No. of Scans	30
Resolution	4 [cm-1]
Apodization	Happ-Genzel

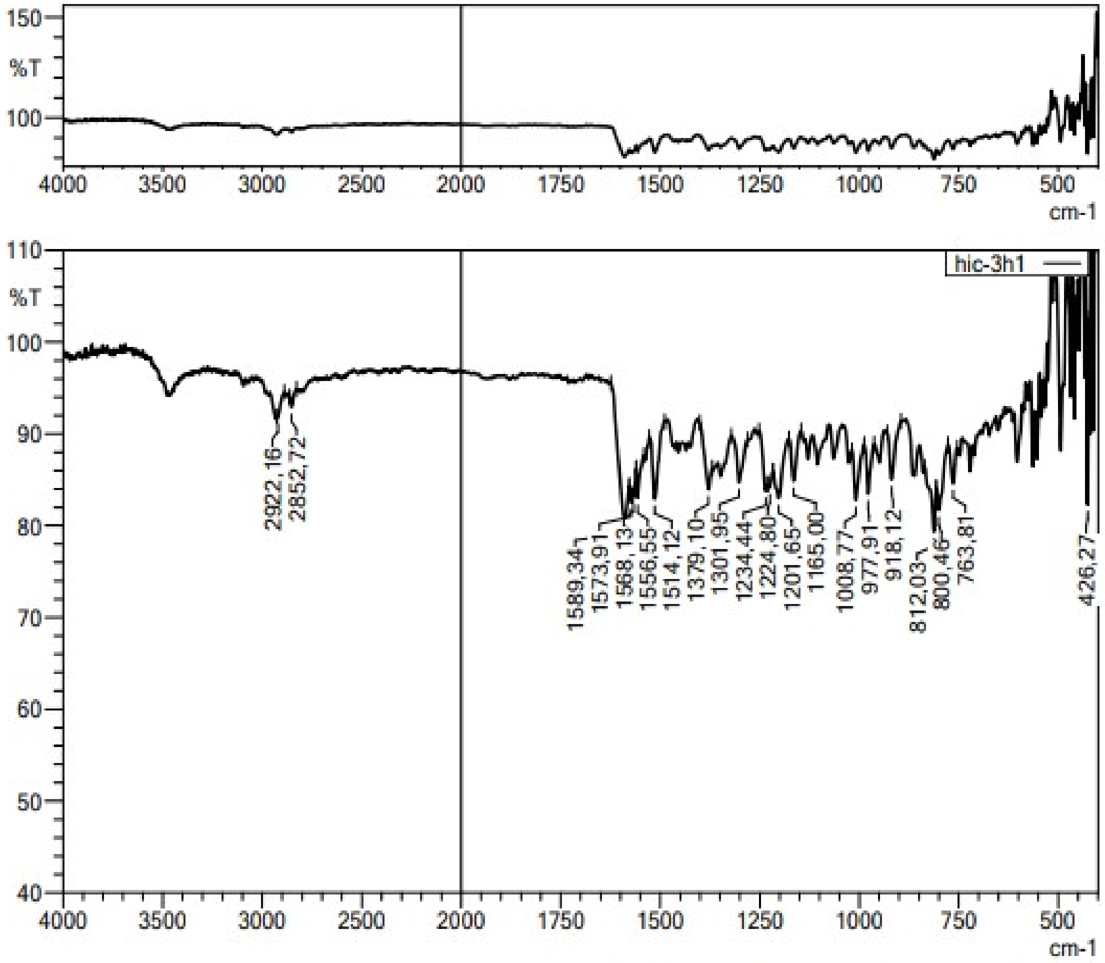

Figure 95S. IR spectrum of compound **D3g**.

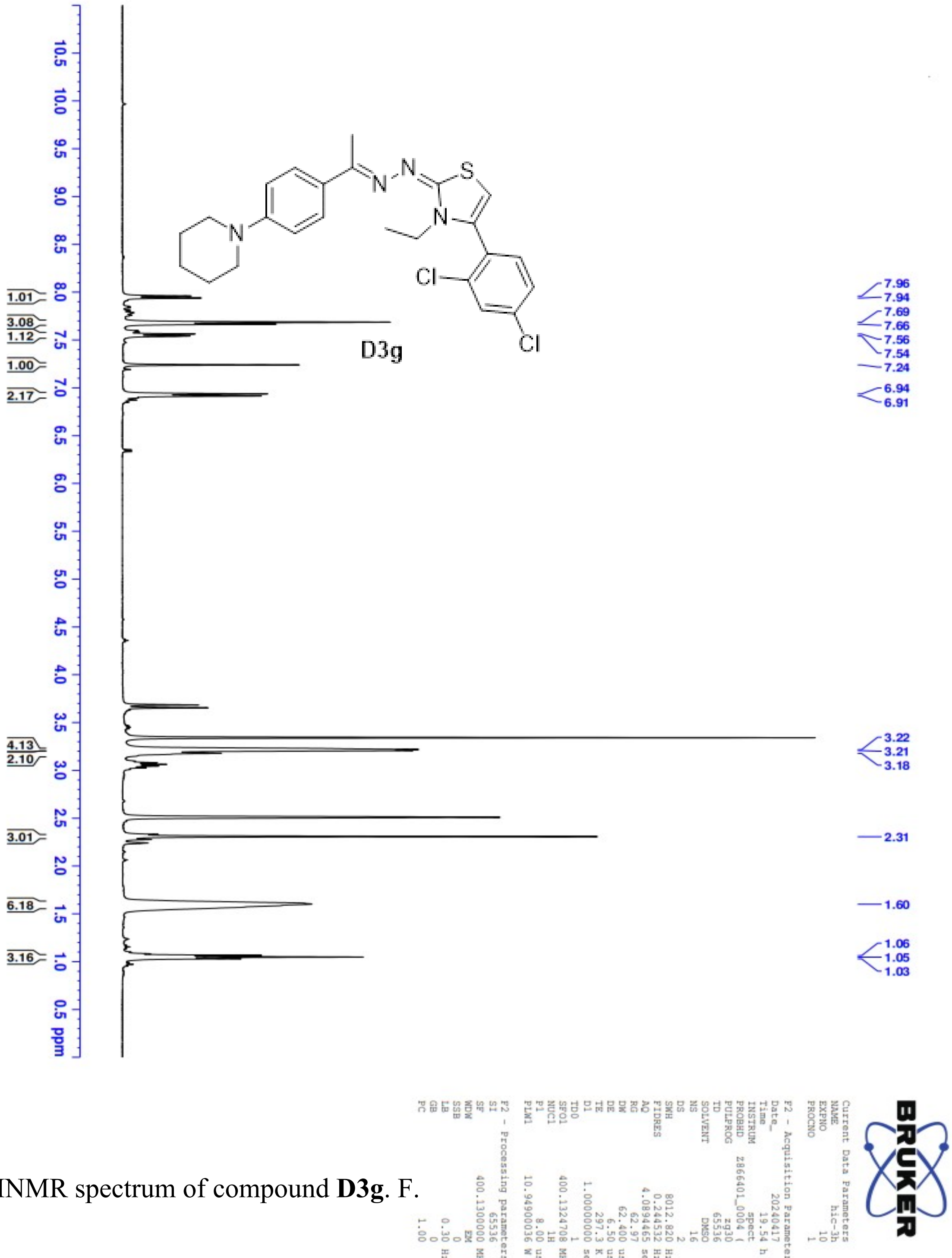

Figure 96S. ^{1}HNMR spectrum of compound **D3g**. F.

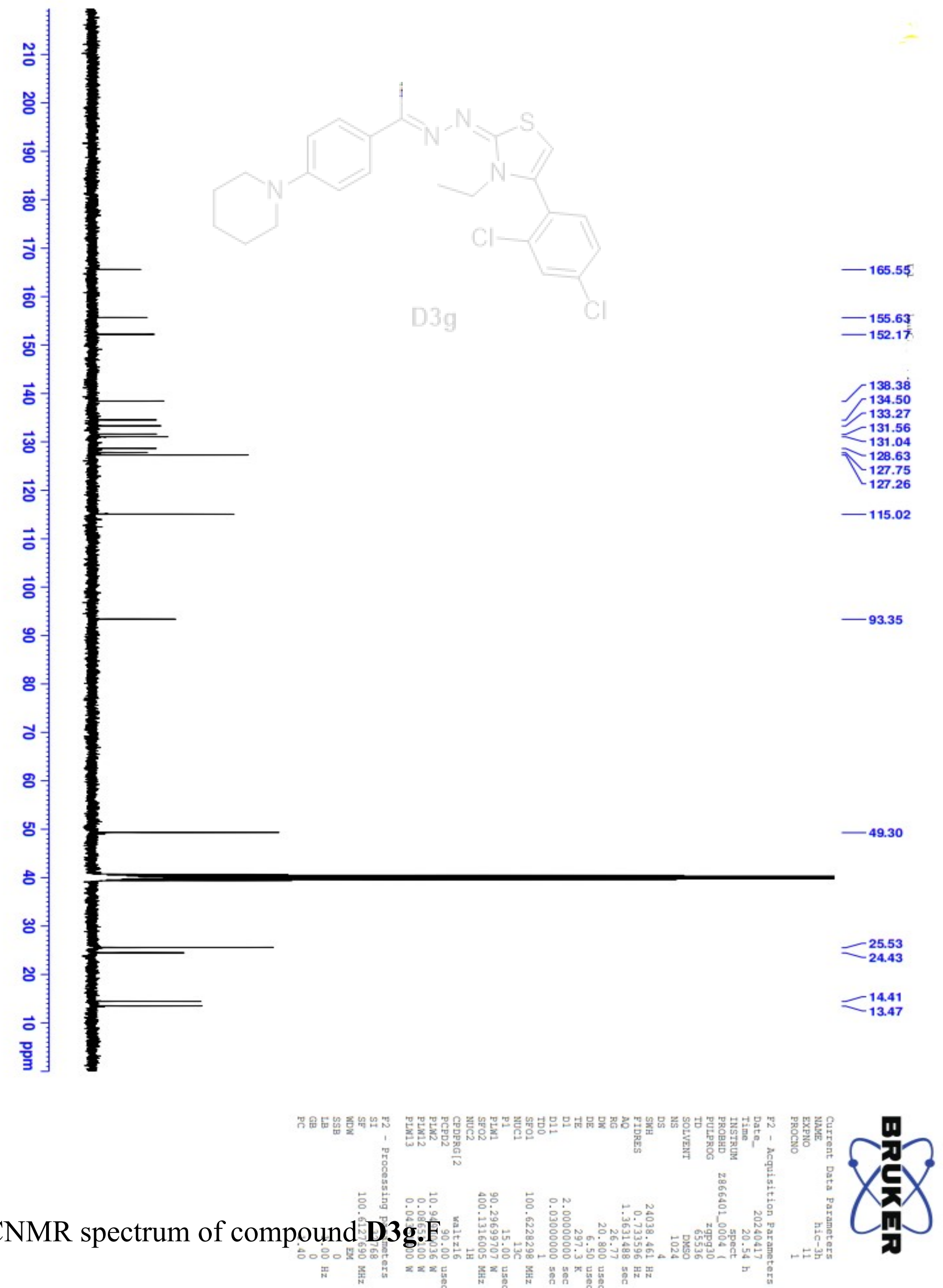

Figure 97S. ^{13}CNMR spectrum of compound **D3g.F**

Data File: C:\LabSolutions\Data\Analiz\derya\MEOH_581.lcd

Elmt	Val.	Min	Max	Elmt	Val.	Min	Max	Elmt	Val.	Min	Max	Elmt	Val.	Min	Max	Use Adduct
H	1	8	33	O	2	0	3	S	2	1	1	Ru	2	0	0	H
C	4	4	32	F	1	0	0	Cl	1	2	2	Pd	2	0	0	Na
N	3	0	5	P	3	0	0	Br	1	0	0	I	3	0	0	

Error Margin (ppm):	5	DBE Range:	0.0 - 30.0	Electron Ions:	both
HC Ratio:	unlimited	Apply N Rule:	no	Use MSn Info:	yes
Max Isotopes:	3	Isotope RI (%):	1.00	Isotope Res:	9000
MSn Iso RI (%):	10.00	MSn Logic Mode:	AND	Max Results:	50

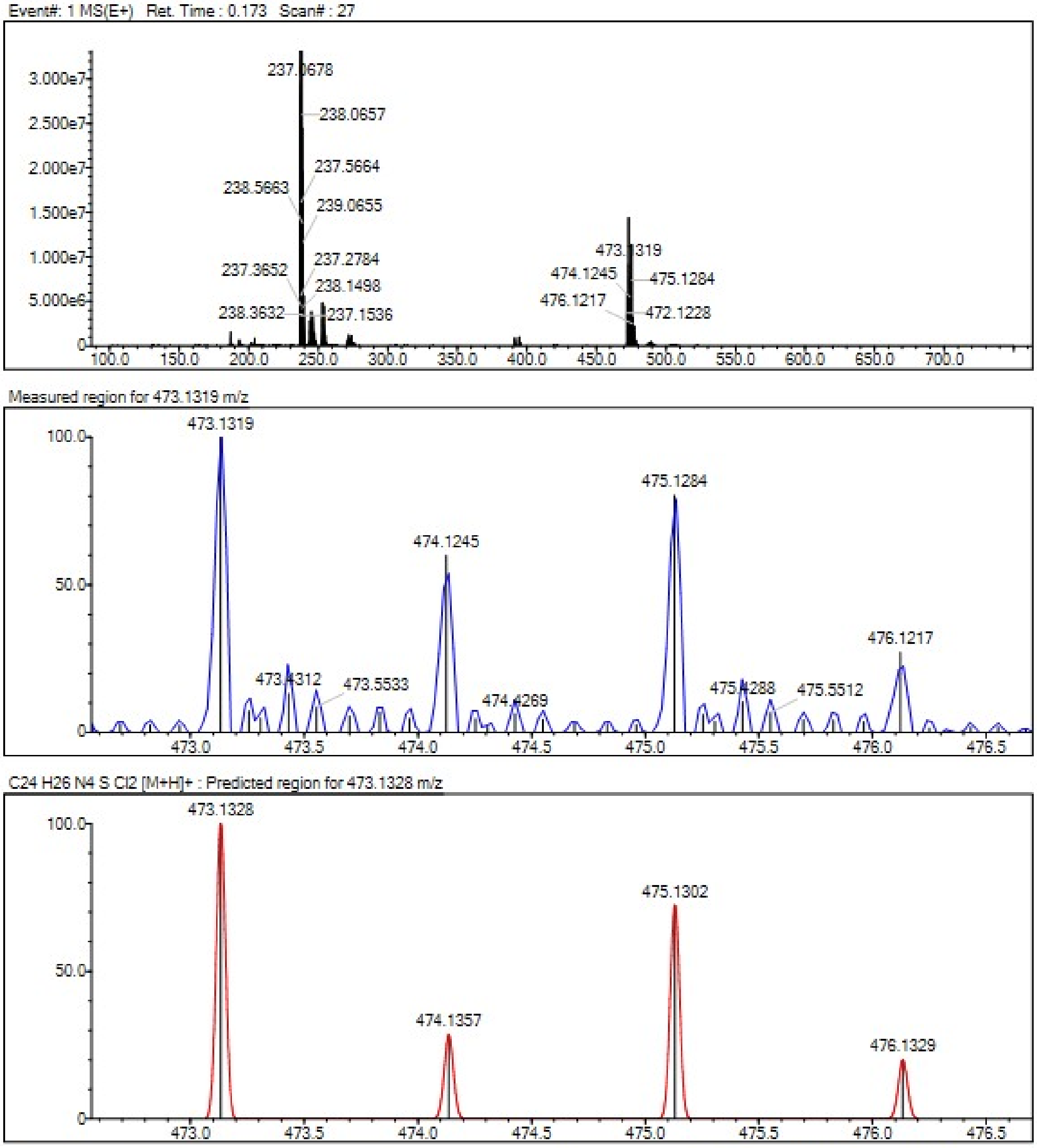

Rank	Score	Formula (M)	Ion	Meas. m/z	Pred. m/z	Df. (mDa)	Df. (ppm)	Iso	DBE
3	35.30	C24 H26 N4 S Cl2	[M+H]+	473.1319	473.1328	-0.9	-1.90	36.11	13.0

Figure 98S. HRMS spectrogram of **D3g.**F

	Item	Value
	Acquired Date&Time	3.05.2024 11:51:41
	Acquired by	System Administrator
	Filename	C:\Users\dopnalab\Desktop\MASAÜSTÜ\sazan\hic\hic-3i1.ispd
	Spectrum name	hic-3i1
	Sample name	hic-3i
	Sample ID	
	Option	
	Comment	
	No. of Scans	30
	Resolution	4 [cm-1]
	Apodization	Happ-Genzel

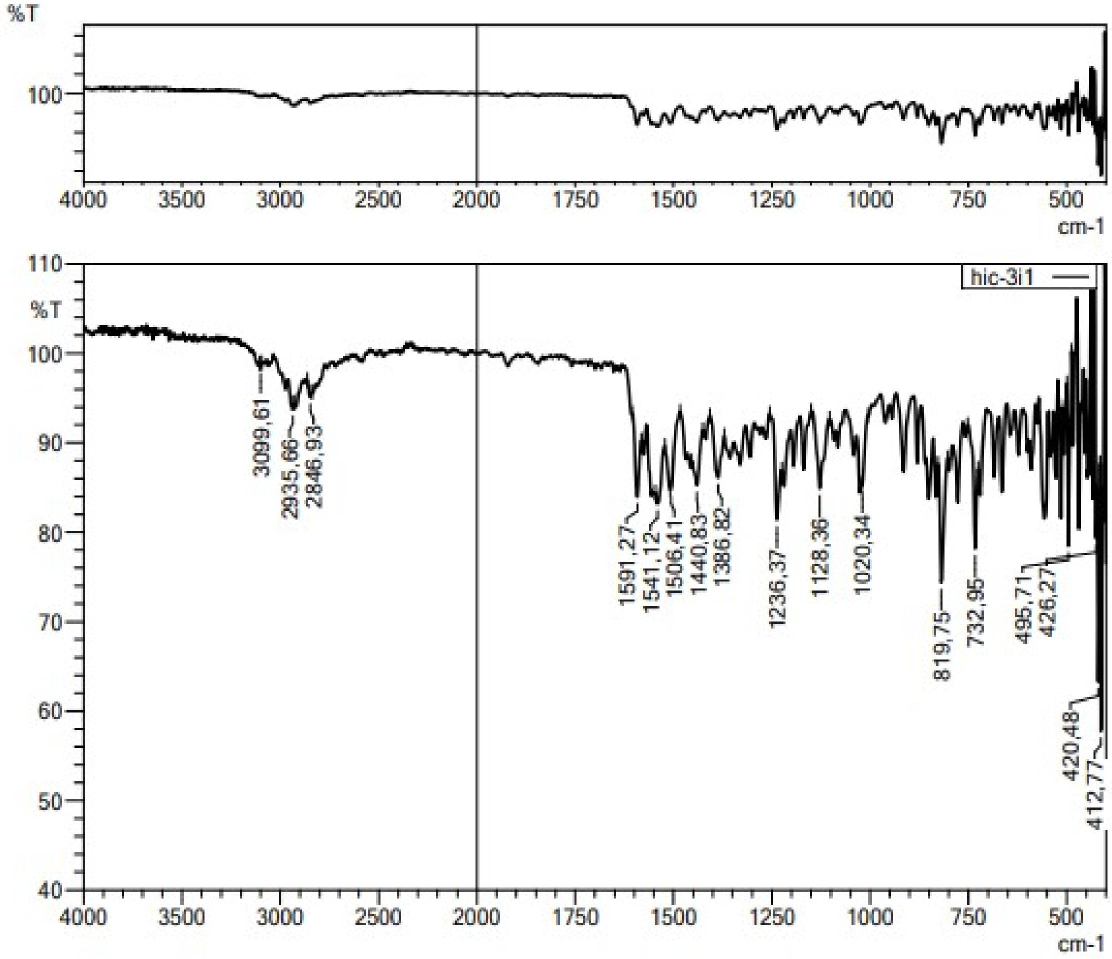

Figure 99S. IR fingerprint of **D3h.**

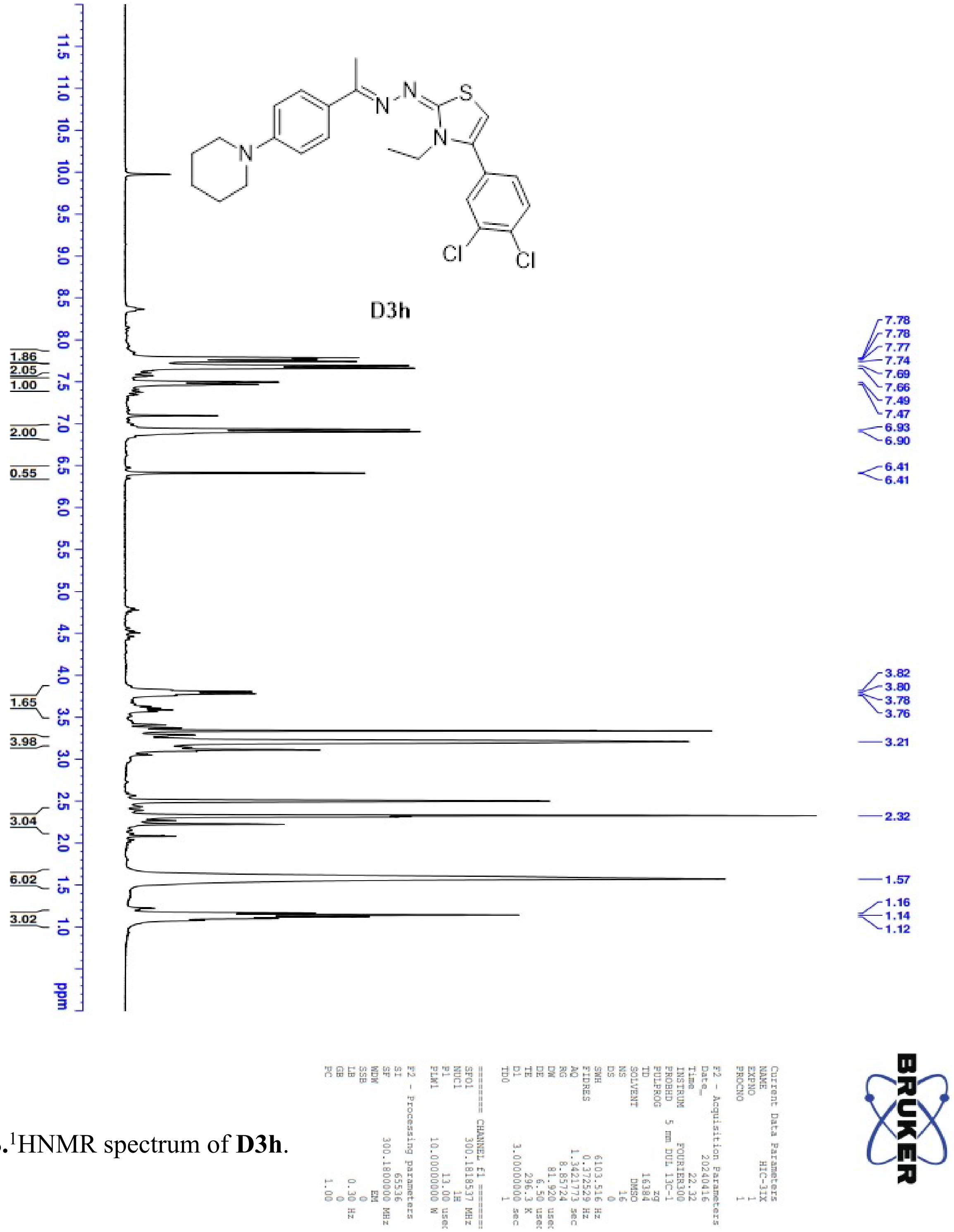

Figure 100S. ^{1}HNMR spectrum of **D3h**.

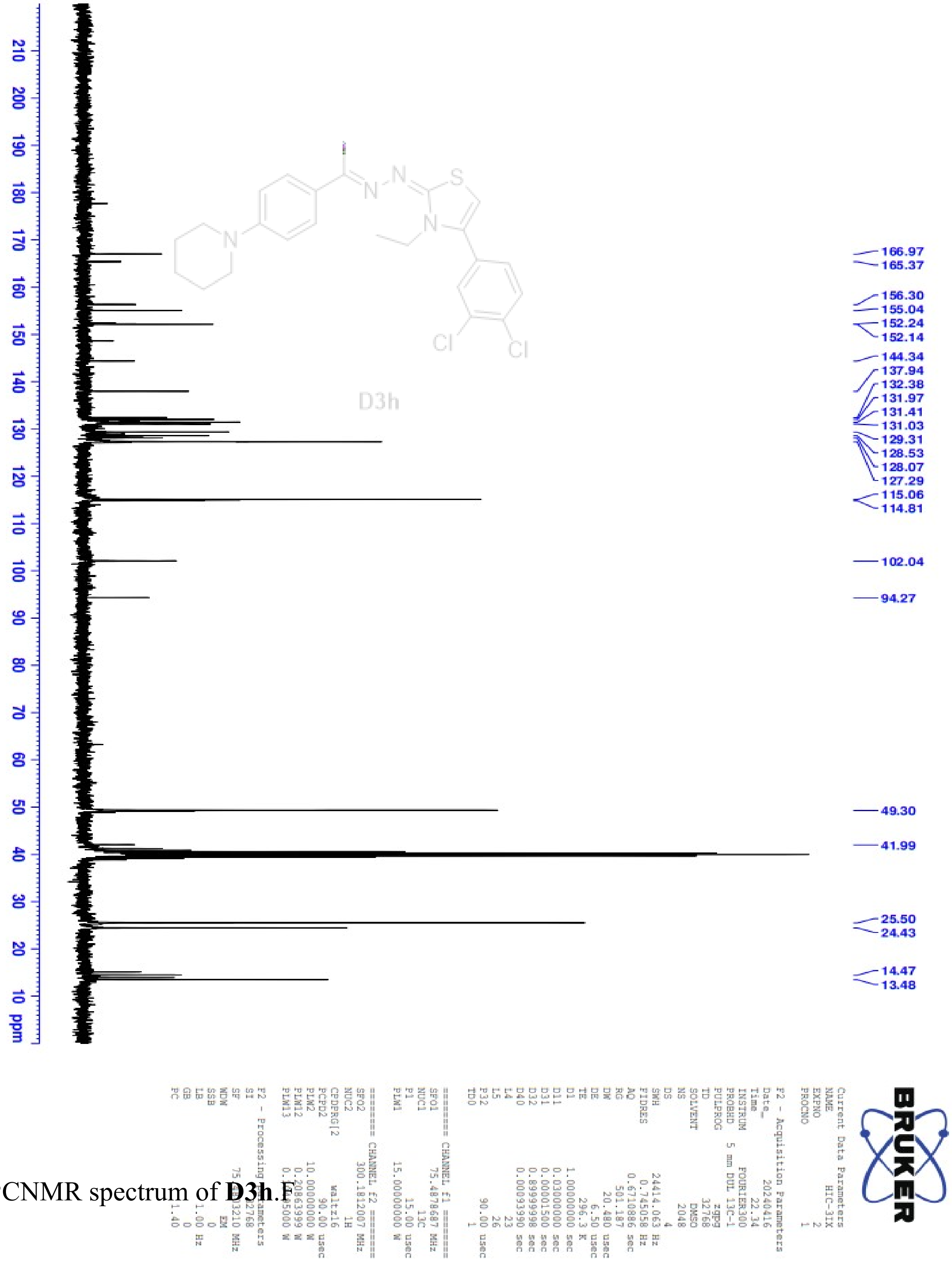

Figure 101S. ^{13}CNMR spectrum of **D3h**.F

Data File: C:\LabSolutions\Data\Analiz\derya\MEOH_632.lcd

Elmt	Val.	Min	Max	Elmt	Val.	Min	Max	Elmt	Val.	Min	Max	Elmt	Val.	Min	Max	Use Adduct
H	1	8	33	O	2	0	0	S	2	0	2	Ru	2	0	0	H
C	4	4	32	F	1	0	0	Cl	1	2	2	Pd	2	0	0	
N	3	0	5	P	3	0	0	Br	1	0	0	I	3	0	0	

Error Margin (ppm): 5
HC Ratio: unlimited
Max Isotopes: 3
MSn Iso RI (%): 10.00

DBE Range: 0.0 - 20.0
Apply N Rule: no
Isotope RI (%): 1.00
MSn Logic Mode: AND

Electron Ions: both
Use MSn Info: yes
Isotope Res: 9000
Max Results: 50

Event#: 1 MS(E+) Ret. Time : 3.467 -> 5.240 Scan# : 521 -> 787

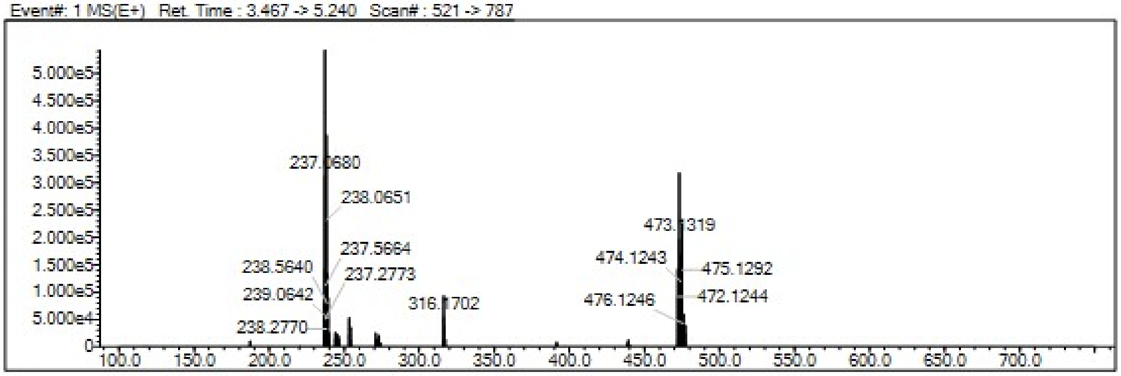

Measured region for 473.1319 m/z

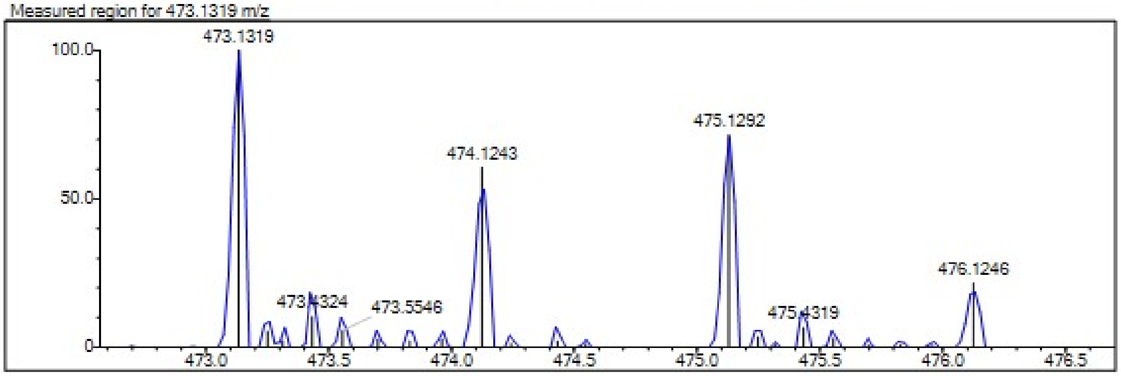

C24 H26 N4 S Cl2 [M+H]+ : Predicted region for 473.1328 m/z

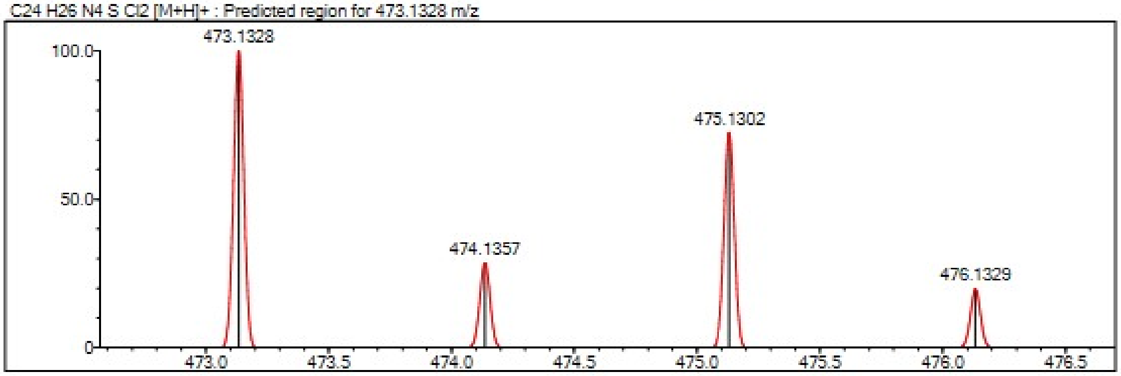

Rank	Score	Formula (M)	Ion	Meas. m/z	Pred. m/z	Df. (mDa)	Df. (ppm)	Iso	DBE
1	39.70	C24 H26 N4 S Cl2	[M+H]+	473.1319	473.1328	-0.9	-1.90	40.61	13.0

Figure 102S. HRMS spectrogram of **D3h.***F*

	Item	Value
	Acquired Date&Time	3.05.2024 11:56:07
	Acquired by	System Administrator
	Filename	C:\Users\dopnalab\Desktop\MASAÜSTÜ\sazan\hic\hic-3j1.ispd
	Spectrum name	hic-3j1
	Sample name	hic-3j
	Sample ID	
	Option	
	Comment	
	No. of Scans	30
	Resolution	4 [cm-1]
	Apodization	Happ-Genzel

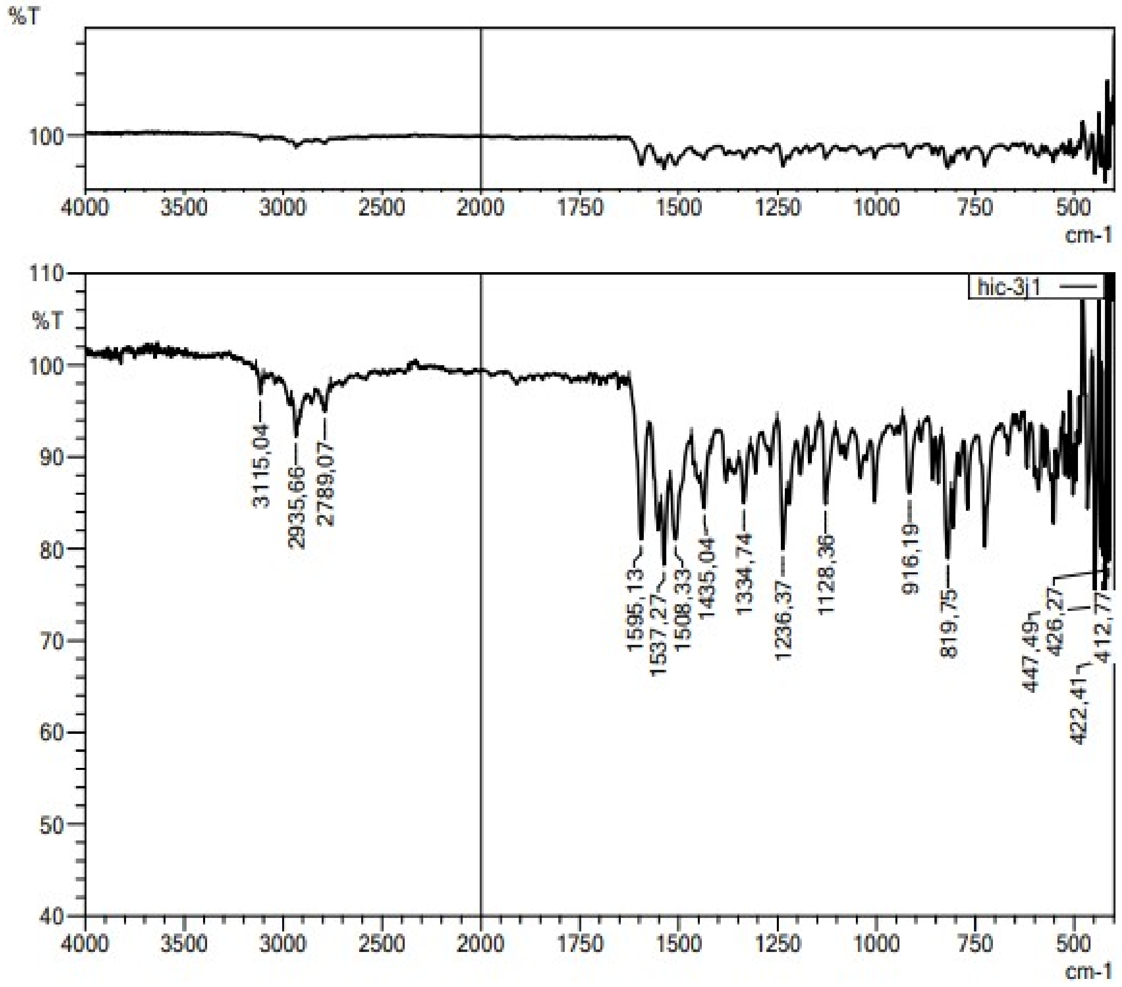

Figure 103S. IR fingerprint of **D3i**.F

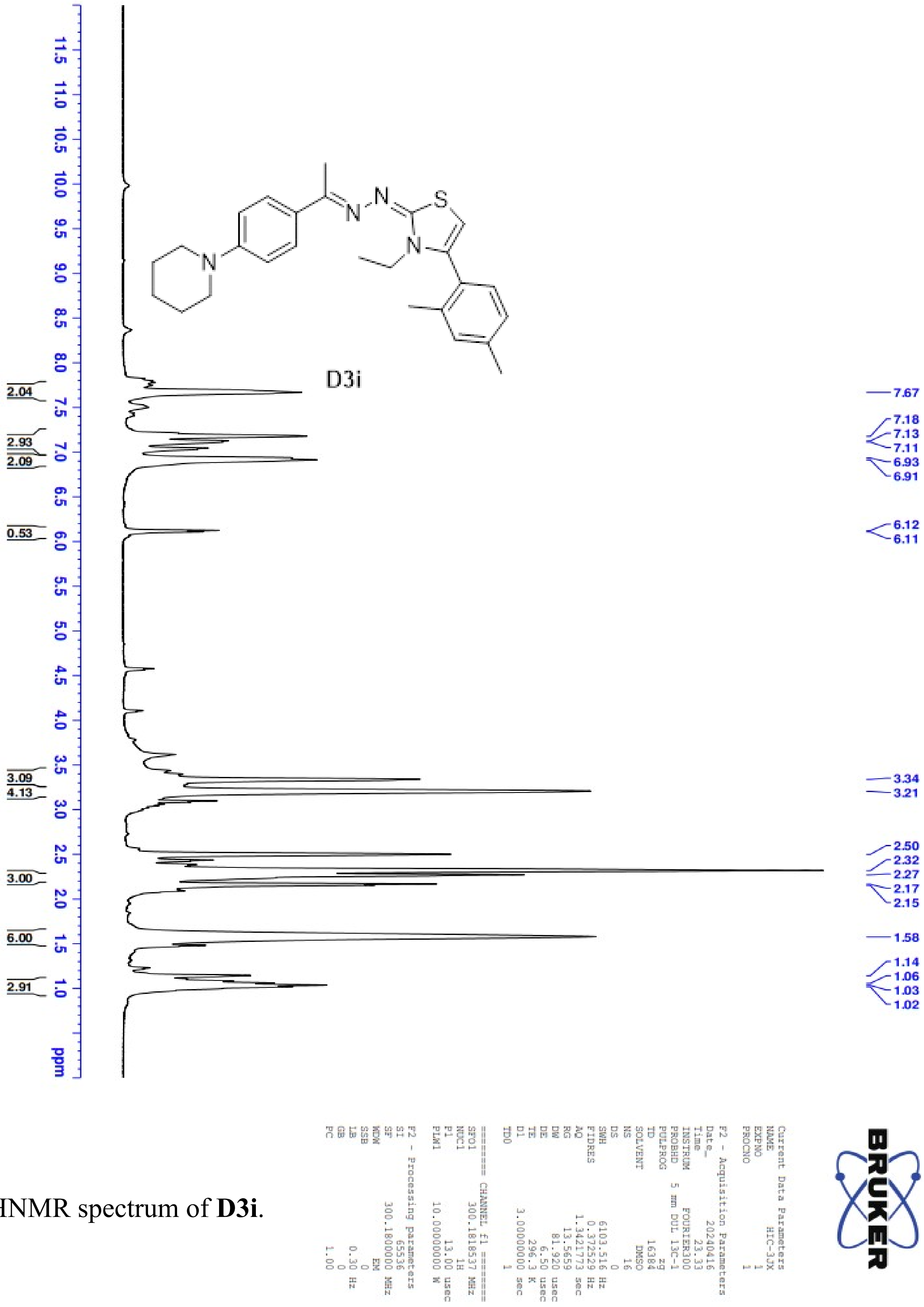

Figure 104S. ^{1}HNMR spectrum of **D3i**.

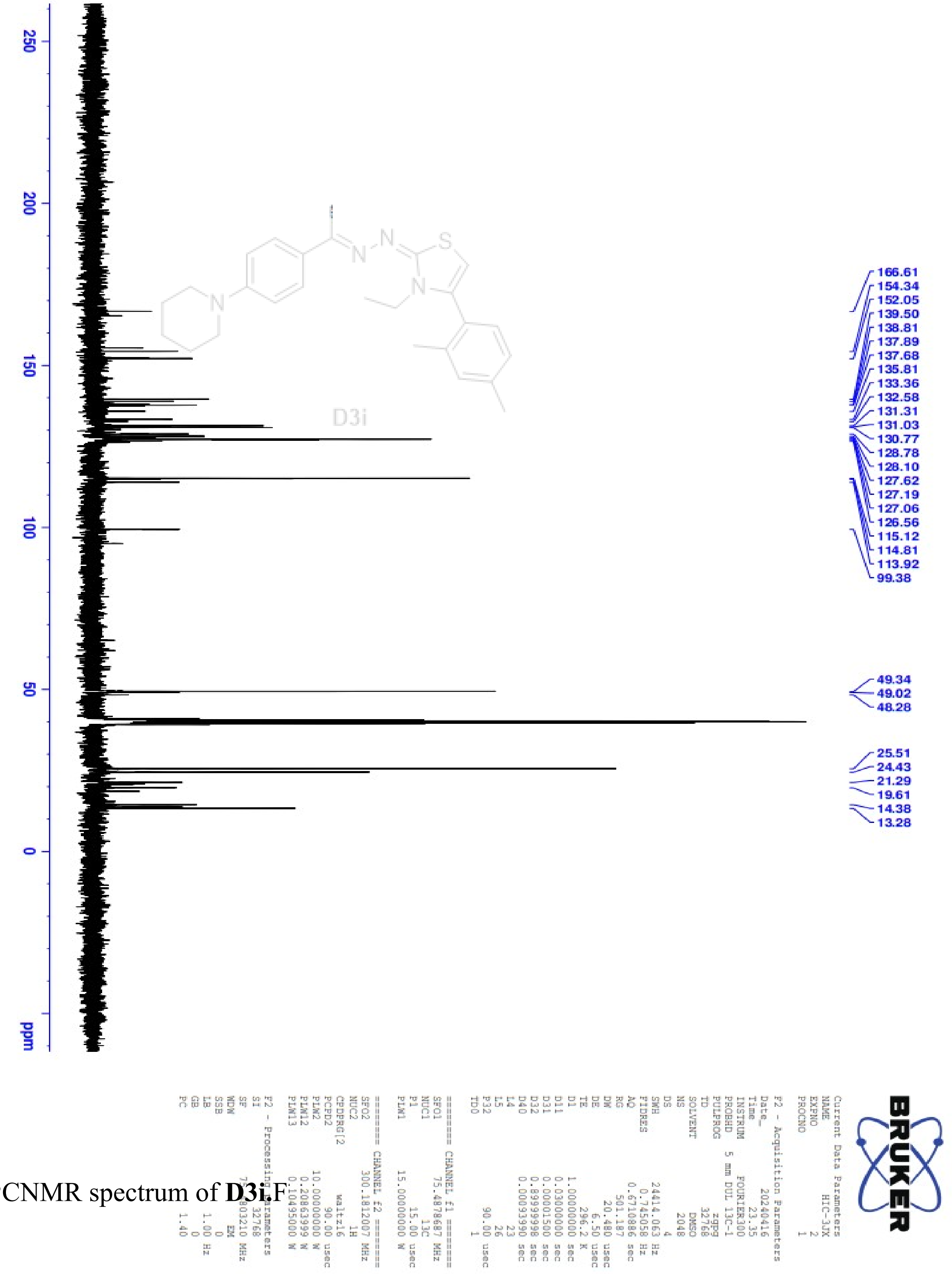

Figure 105S. ^{13}CNMR spectrum of **D3i.**

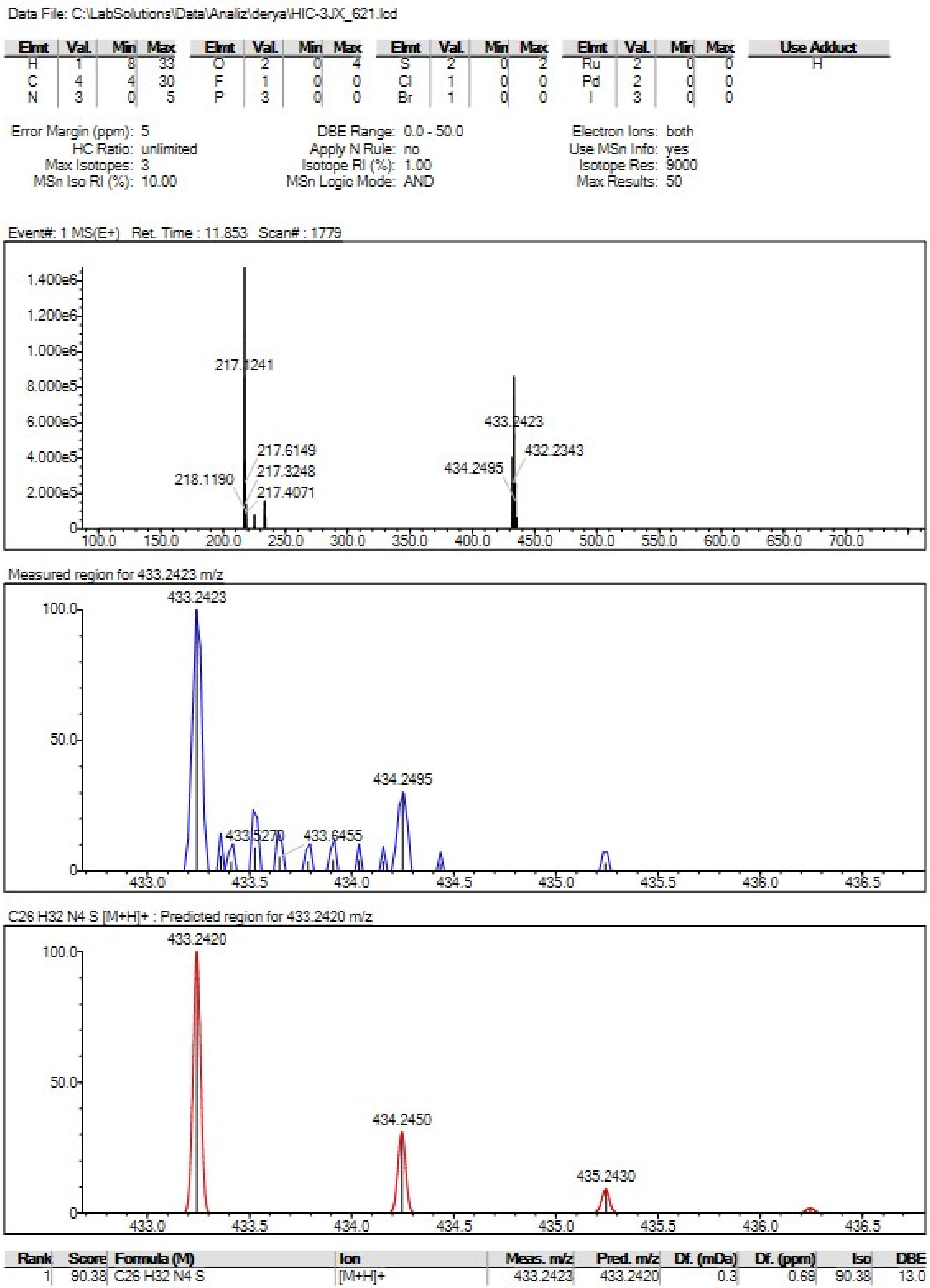

Figure 106S. HRMS spectrogram of **D3i.**F

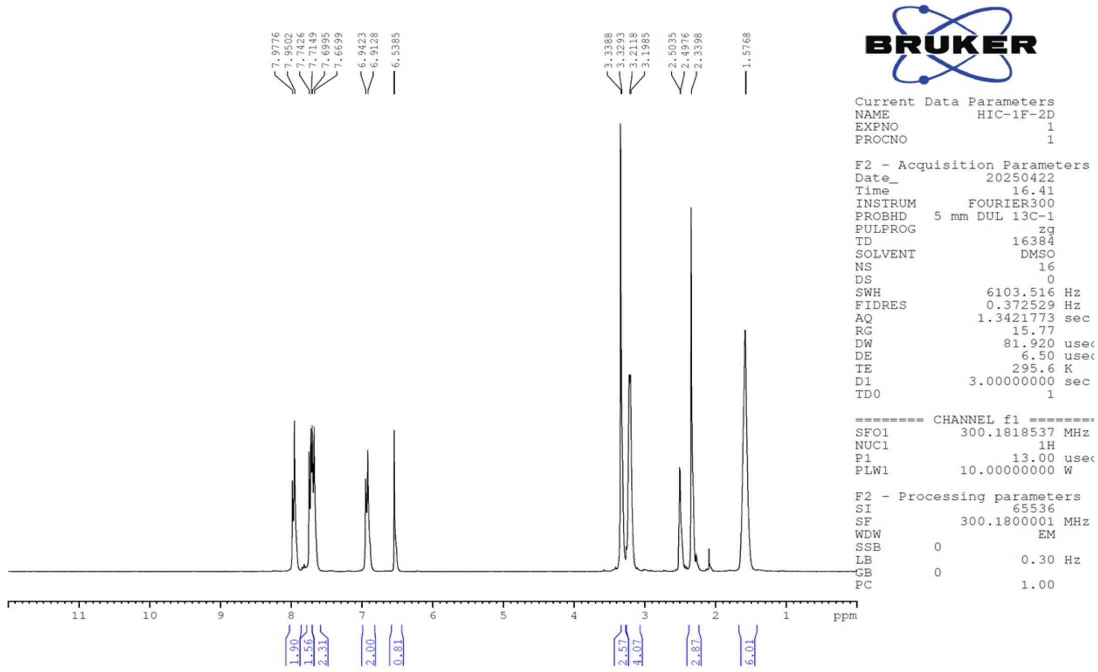

Figure 107S: 1HNMR spectra of compound **D1f** (for 2D NMR)

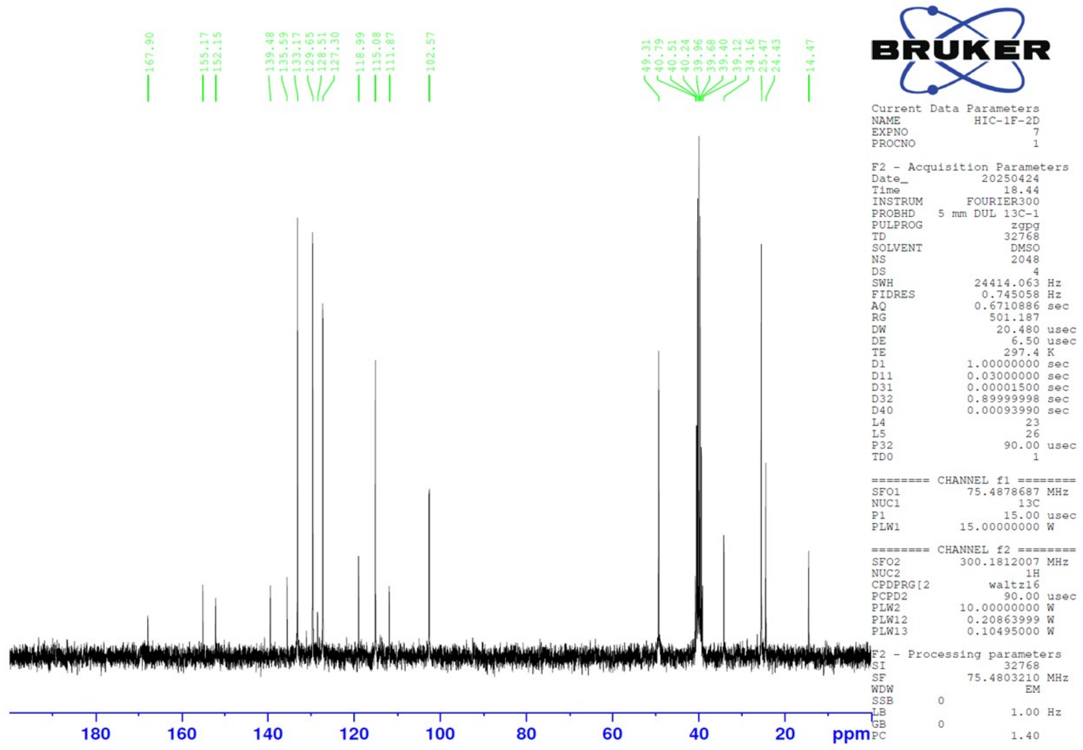

Figure 108S: ^{13}CNMR spectra of compound **D1f** (for 2D NMR)

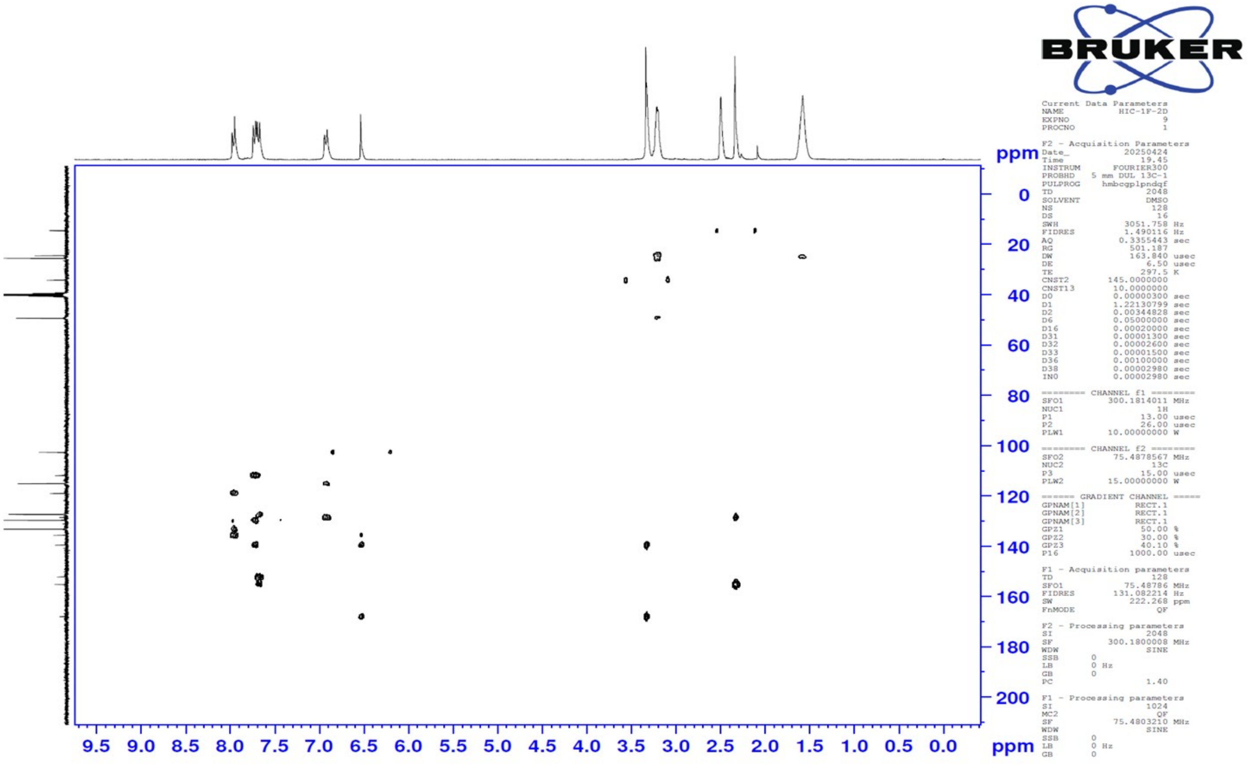

Figure 109S: HMBC spectra of compound **D1f.**

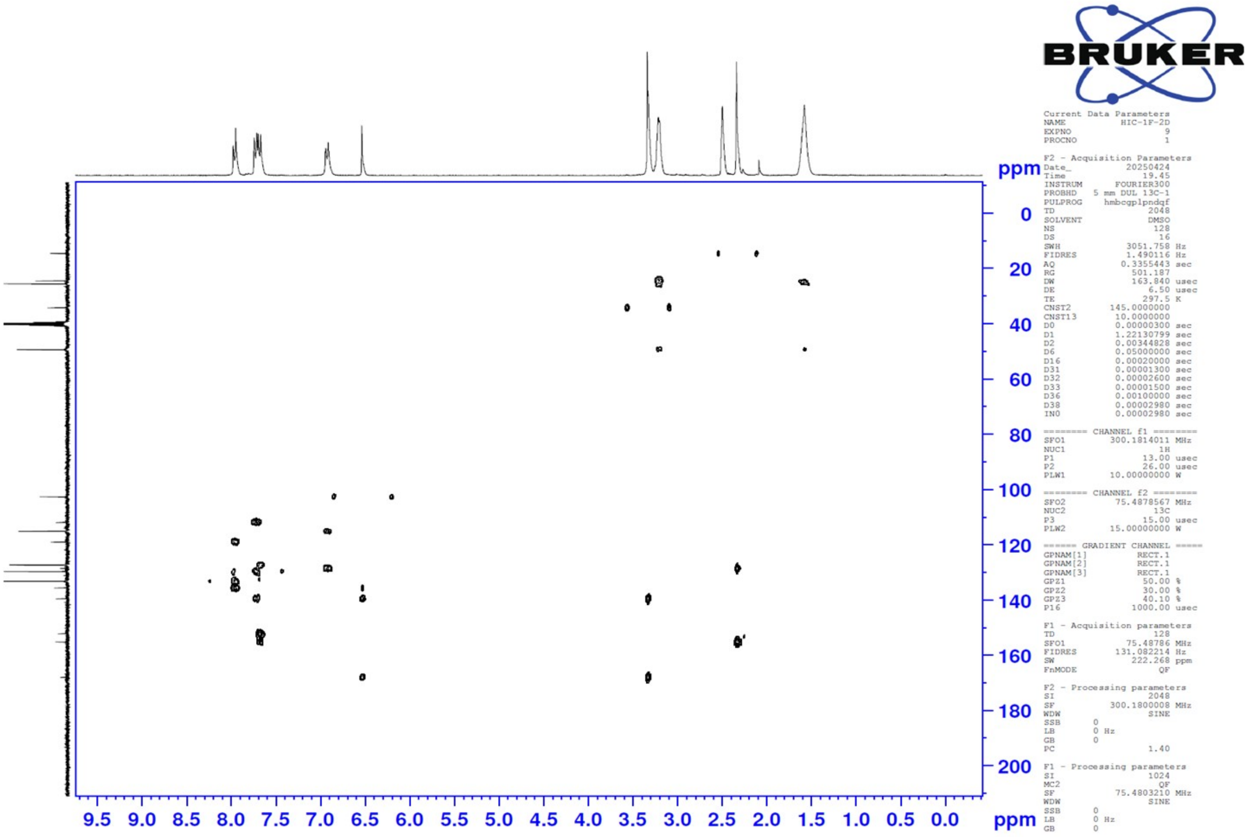

Figure 110S: HSQC spectra of compound **D1f.**

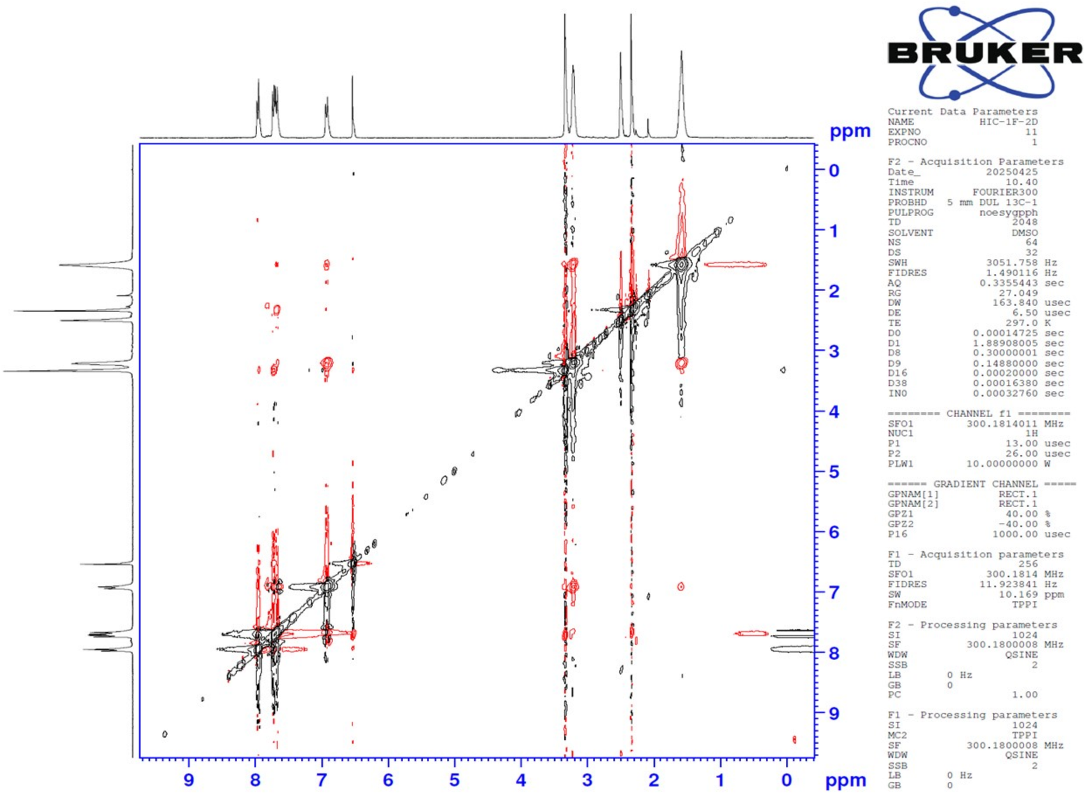

Figure 111S: NOESY spectra of compound **D1f.**
